# Systematic review of risk prediction models for arteriovenous fistula dysfunction in maintenance hemodialysis patients

**DOI:** 10.1371/journal.pone.0324004

**Published:** 2025-05-19

**Authors:** Shiyan Yao, Guannan Ma, Yongze Dong, Mengjiao Zhao, Luchen Chen, Wenhao Qi, Huajuan Shen

**Affiliations:** 1 School of Public Health and Nursing, Hangzhou Normal University, Hangzhou, China; 2 Department of Nursing, Zhejiang Provincial People’s Hospital (Affiliated People’s Hospital), Hangzhou, China; 3 School of Nursing, Zhejiang Chinese Medical University, Hangzhou, China; Coventry University, UNITED KINGDOM OF GREAT BRITAIN AND NORTHERN IRELAND

## Abstract

**Objective:**

The prevalence of arteriovenous fistula (AVF) dysfunction and its associated complications among patients undergoing maintenance hemodialysis (MHD) underscores the necessity for predictive models aimed at early diagnosis and intervention. Although various models for predicting AVF risk have emerged, a comprehensive review of their advancements and challenges is currently lacking. This study aims to systematically evaluate risk prediction models for AVF failure in MHD patients, thereby providing insights for clinical practitioners in selecting or developing more effective risk assessment tools.

**Methods:**

A systematic search was conducted across multiple databases, including PubMed, Embase, Web of Science, Cochrane Library, ClinicalTrials.gov registry platform,the China Biomedical Literature Database, CNKI, WanFang Database, and the VIP Database, focusing on studies related to risk prediction models for AVF failure in MHD patients. Google Scholar was also searched to retrieve gray literature. The search encompassed literature from the inception of these databases up to August 1, 2024. Two independent researchers performed literature screening and data extraction, utilizing the Prediction Model Risk of Bias Assessment Tool to evaluate the methodological quality of the included studies.

**Results:**

Initially, 2,052 studies were identified. After thorough screening, 11 studies were ultimately included, detailing 11 distinct risk prediction models for AVF failure in MHD patients. The sample sizes of these studies ranged from 126 to 14,892 participants. The identified models exhibited varying predictive performances, highlighting common limitations such as small sample sizes, improper handling of missing data, and a lack of external validation.

**Conclusion:**

The risk prediction models for AVF failure among MHD patients demonstrate adequate predictive performance; however, the overall quality of the research necessitates improvement. Future studies should prioritize refining research design and reporting processes, as well as validating and enhancing existing models to ascertain their effectiveness and feasibility in clinical practice.

## 1. Introduction

As the most common form of renal replacement therapy, MHD accounts for approximately 69% of all replacement therapies and 89% of all dialysis methods [1]. The vascular access used for dialysis serves as a lifeline for patients undergoing MHD. Establishing long-term, stable vascular access is essential to ensure the quality of dialysis for these patients. Although guidelines [2] recommend that the selection of vascular access be individualized based on a comprehensive assessment of the patient’s overall health and vascular status,AVF remains the preferred access type due to its low risk of fatal infection, prolonged duration of use, and fewer clinical adverse events [3]. However, AVF have notable drawbacks, particularly concerning long-term patency, with reported rates ranging from 62% to 68% after one year and declining to 38% to 56% after two years, approximately 47.5% of patients require additional interventions to maintain patency [4]. Complications such as intimal hyperplasia, vascular stenosis, thrombosis, and embolism may arise due to prolonged dialysis treatment and underlying medical conditions, potentially leading to fistula dysfunction [5]. The proportion of MHD patients requiring hospitalization due to AVF failure may reach as high as 20% to 25% [6]. Dysfunction of the AVF can result in inadequate dialysis, increased treatment burden for patients, and elevated rates of hospitalization and mortality [4]. Consequently, it is crucial to implement risk stratification management based on the risk factors associated with AVF failure in MHD patients, thereby enabling early identification of high-risk individuals and timely intervention.In recent years, the development of digital medicine has led to significant advancements in risk prediction models based on big data, providing important support for clinical decision-making. While some studies have focused on risk prediction models for AVF failure in MHD patients, the results have exhibited considerable variability, with potential biases and concerns regarding clinical applicability requiring further exploration. Furthermore, Meng et al. [7] conducted a systematic review of existing AVF-related risk prediction models; however, this study included models predicting AVF patency and maturation and only searched three English databases, potentially overlooking significant and recent research.Therefore, this study aims to comprehensively review and systematically evaluate risk prediction models for AVF dysfunction in MHD patients, with the objective of providing insights for clinicians in selecting appropriate assessment tools.

## 2. Materials and methods

### 2.1 Study design

This systematic review uses the PICOTS framework, as recommended by the Cochrane Prognosis Methodology Group, to develop an evidence-based research question

P (population): MHD patients.

I  (index prediction model): under evaluation is the AVF failure prediction model. C (comparator): No comparator model.

O (outcome): Is the incidence of AVF failure in MHD patients.

T (timing): For applying the model is either prior to dialysis or during the inter-dialytic period.

S (setting): For the model application is within hemodialysis centers.

### 2.2 Protocol and registration

This systematic review has been registered with the International Prospective Register of Systematic Reviews (PROSPERO: CRD42024589302).

### 2.3 Literature search strategy

A comprehensive computer-based search was conducted across several English databases, including PubMed, Embase, Web of Science, and the Cochrane Library, as well as Chinese biomedical literature databases, specifically the China National Knowledge Infrastructure (CNKI), WanFang Data, and VIP Database. In addition, we also searched the ClinicalTrials.gov registry platform, Google Scholar was also searched to retrieve gray literature. The search strategy incorporated both subject headings and free-text terms, tailored to the specific characteristics of each database. The search timeframe extended from the inception of each database until August 1, 2024. Key search terms included “renal dialysis” (e.g., “hemodialysis,” “maintenance hemodialysis,” and “MHD”), “arteriovenous fistula” (e.g., “AVF,” “Arteriovenous Aneurysm”), “dysfunction” (e.g., “failure to thrive,” “embolisms,” “Dysfunction,” “Failure,” “Thrombosis,” “stenosis”), and “risk prediction models” (e.g., “risk assessment,” “predict*,” and “prediction model”). The complete search strategy can be found in the supplementary materials (S1 Table in S1 Appendix).

### 2.4 Inclusion and exclusion criteria

The inclusion criteria for this study are as follows: (1) Participants must be aged 18 years or older and have undergone regular dialysis for a minimum of three months; (2) The research must focus on the development and/or validation of AVF failure risk prediction models for MHD patients, with only the most recent and comprehensive data considered in cases of duplicated publications; (3) Eligible study types include case-control studies and cohort studies. The exclusion criteria are as follows: (1) Studies from which full texts could not be obtained or that consist solely of conference abstracts; (2) Research that constructs models based on systematic reviews or meta-analysis results; (3) Models that include fewer than two predictive variables; (4) Comparative studies of models.

### 2.5 Literature screening and data extraction

The literature obtained from the search was imported into Note Express reference management software. Duplicate studies were identified and removed manually by the software. Subsequently, two trained researchers, Shiyan Yao and Luchen Chen, performed the literature screening, data extraction, and cross-verification, ensuring strict adherence to the inclusion and exclusion criteria outlined in the Cochrane Handbook. The researchers worked independently and were blinded to each other’s selections to ensure objectivity. Any unresolved discrepancies were addressed through discussion, with assistance from a third party, Yongze Dong, if necessary.

### 2.6 Quality assessment

Two trained researchers, Shiyan Yao and Luchen Chen, independently evaluated the risk of bias and applicability of the included studies using the prediction model bias risk assessment tool(PROBAST) [8]. It is a tool that includes three parts: bias risk assessment, applicability risk assessment, and overall risk assessment. It aims to rigorously evaluate research that focuses on the development, validation, or updating of personalized prediction models.

#### 2.6.1 Bias risk assessment.

The bias risk assessment comprises four domains: study population, predictive factors, outcomes, and data analysis, encompassing a total of 20 signaling questions. Each question offers the following response options: “Yes,” “Probably Yes,” “No,” “Probably No,” and “Unclear.” If all questions in a domain are answered with “Yes” or “Probably Yes,” that domain is considered to have a low risk of bias. Conversely, if any question receives a “No” or “Probably No,” that domain is deemed to have a high risk of bias. If any question is answered as “Unclear,” it indicates that the risk of bias in that domain remains uncertain due to insufficient information. When all domains are assessed as having a low risk of bias, the overall risk for the study is classified as low. If any domain presents a high risk, the overall bias risk for the study is classified as high. Additionally, if the bias risk for one domain is unclear while the other domains are classified as low, the overall bias risk for the study remains uncertain [8].

#### 2.6.2 Applicability assessment.

The applicability assessment consists of three domains: study population, predictive factors, and outcomes. Each domain can be rated as “High Applicability,” “Low Applicability,” or “Unclear Applicability.” If all domains are rated as having high applicability, the overall applicability of the study is also classified as high. However, if any domain is rated as low, the overall applicability is considered low. Furthermore, if any domain’s applicability is unclear while the others are rated high, the overall applicability is classified as unclear.

### 2.7 Data extraction

Data extraction was conducted by two independent researchers, Shiyan Yao and Luchen Chen, utilizing a data extraction form developed based on critical appraisal and data extraction for systematic reviews of prediction modelling studies(CHARMS) [9] on August 15, 2024. The extraction process involved analyzing the titles, abstracts, and full texts of the included literature. Extracted data included: study references, publication year, country of origin, study type, study population, outcome definitions, number of candidate variables, sample size, incidence rates of outcomes, methods for handling missing data, modeling methods, model performance, calibration methods, validation methods, and the final set of included predictive factors.

### 2.8 Statistical methods

Meta-analysis was performed using Stata software (version 15.0, Stata Corporation, College Station, Texas, USA). The Q test and I² statistic were used to assess the heterogeneity among the included studies. If P ≥ 0.1 and I² ≤ 50%, it indicated no significant heterogeneity between the studies, and a fixed-effects model was applied for the meta-analysis. If P < 0.1 or I² > 50%, indicating the presence of heterogeneity, a random-effects model was used, and sensitivity analysis was conducted to identify the sources of heterogeneity. Publication bias was assessed using Egger’s linear regression test. A P value > 0.05 suggested no significant publication bias, while a P value < 0.05 indicated the presence of publication bias.

### 2.9 Assessment of evidence quality

The quality of the evidence for outcome measures was assessed by two researchers (Shiyan Yao and Luchen Chen) using the Grading of Recommendations Assessment, Development, and Evaluation (GRADE) system [10]. Any disagreements were resolved through discussion with a third author (Mengjiao Zhao). The core of the GRADE methodology involves classifying evidence quality into four levels: high, moderate, low, and very low. The quality of evidence can be rated up based on three factors: large effect size, dose-response relationship, and adequate control of potential confounding factors. Evidence quality can be downgraded due to the following factors: high risk of bias, indirectness of results, inconsistency, imprecision, and publication bias. Additionally, randomized controlled trials are classified as the highest level of evidence, whereas observational studies (e.g., cohort studies, case-control studies, cross-sectional studies, etc.) are typically considered low-quality evidence by default.

## 3 Results

### 3.1 Literature search process and results

**Fig 1:**
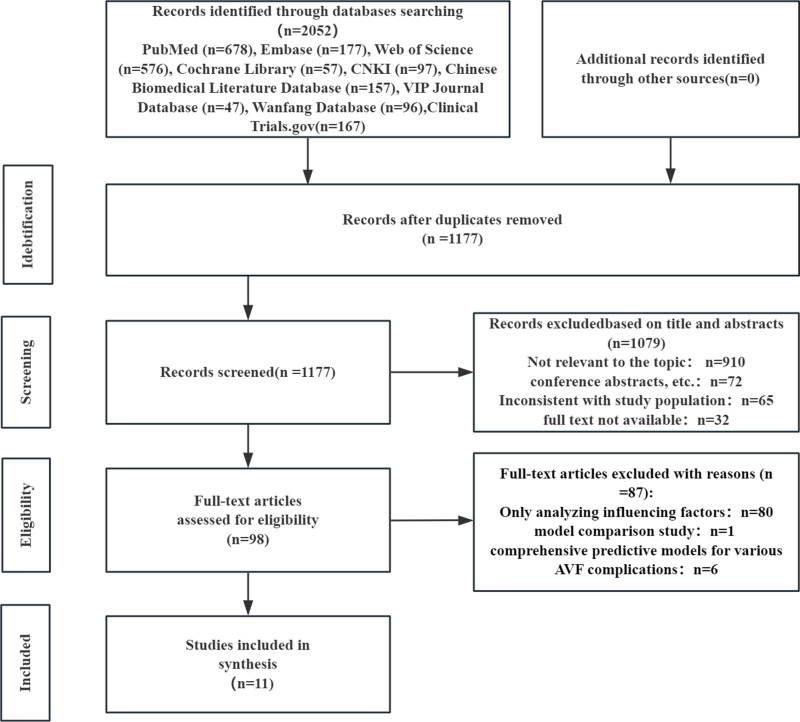
Literature screening flow chart.

Through database searches and reference tracing, a preliminary total of 2,052 relevant articles were identified. After deduplication, 1,177 articles remained. Following a systematic screening of titles, abstracts, and full texts, this study ultimately included 11 articles [Fig 1] [11–21].

### 3.2 Basic characteristics of included studies

The studies included in this review were published between 2015 and 2024. Among them, six were published in English [13,16,18–21] and five in Chinese [10,11,13,14,16]. Nine studies were conducted within the last five years, with six originating from China [11–15,17], while the remaining three were conducted in Portugal [16], Thailand [18], and the United States [19]. Of the 11 studies, five [10–12,14] were case-control studies, four [13,15,17,18] were retrospective studies, and two [19,20] were prospective studies. Specifics are outlined in Table 1.

**Table 1:** Basic characteristics of the included studies.

Study	Year	Country	Study designs	Population	Definition of end result
Weng^11^	2024	China	Case-control study	MHD patients	1,2,3,4,5
Gong^12^	2024	China	Case-control study	MHD patients	1,6
Wang^13^	2024	China	Case-control study	MHD patients, regular dialysis ≥6 months	7
Liang^14^	2022	China	Retrospective study	MHD patients	——
Che^15^	2022	China	Case-control study	MHD patients	6
Peralta^16^	2021	Portugal	Retrospective study	MHD patients	8
Wang^17^	2020	China	Case-control study	MHD patients	1, 2, 4
Wongmahisorn^18^	2020	Thailand	Retrospective study	MHD patients with regular dialysis for ≥12 months	4
Qian^19^	2020	United States	Retrospective study	MHD patients, age ≥ 67 years	9
Eslami^20^	2016	UK	Prospective cohort study	MHD patients	4
Masengu^21^	2015	UK	Prospective cohort study	MHD patients	8

Note: Definition of end result: 1 = Local stenosis of AVF > 50% of the diameter of the nearby normal vessel after 3 months of normal use; 2 = clinical manifestations include weak vascular murmur, diminished tremor, or failure to palpate; 3 = endovascular blood flow consistently <500 mL/min; 4 = inability to satisfy the required flow for dialysis; 5 = puncture difficulty; 6 = Dialysis pump-controlled blood flow less than 200 ml/min; 7 = AVF inlet blood flow consistently <600 mL/s at all times; 8 = Includes AVF thrombosis, replacement of vascular access, need for interventions to maintain patency, and hospitalisation for AVF complications; 9 = Loss of function 6 months after AVF establishmen

### 3.3 Basic features of the models

The total sample size across the 11 models ranged from 126 to 14,892 cases, with event incidence rates between 17.5% and 49.5%. The number of candidate predictive variables varied from 6 to 100. Among the 11 studies, only one reported on handling missing variables [16]; the others did not provide information regarding missing data. In terms of variable selection, three studies [13,16,17] selected variables based on univariate analysis.Specifics are outlined in Table 2.

**Table 2:** Basic characteristics of AVF dysfunction risk prediction model.

Study	Number of predictors	Variable selection method	Sample size [modelling/validation]	Conclusion incidence	Missing data treatment method
Weng^11^	15	Use VIMP method to rank the importance of variable	156	25.60%	——
Gong^12^	19	Multivariate Logistic stepwise regression analysis	126	24.60%	——
Wang^13^	18	Multifactor Logistic regression analysis followed by LASSO regression screening	150	41.30%	——
Liang^14^	18	Single-factor analysis followed by multifactor analysis	264/84	23.11%	——
Che^15^	23	LASSO	196	——	——
Peralta^16^	109	SHAP	13,369	26.40%	XGBoost
Wang^17^	17	Single-factor analysis followed by multifactor analysis	280/64	20.40%	——
Wongmahisorn^18^	8	Single-factor analysis followed by multifactor analysis	195	——	——
Qian^19^	15	——	14892	49.50%	——
Eslami^20^	17	Multifactor Logistic regression analysis	376	17.50%	——
Masengu^21^	8	Maintaining continuity Logistic regression first, followed by use of stepwise backward elimination	525	41%	——

### 3.4 Model performance and predictive factors

Nine studies applied logistic regression methods [12–15,17–21], while one study [11] utilized Cox proportional hazards regression and random forest analysis, and another study [16] employed XGBoost for model development. Among the included studies, nine [12–21] reported the AUC, which ranged from 0.53 to 0.934. Except for the studies by Qian et al. [19] and Masengu et al. [21], all other models exhibited an AUC greater than 0.7. Eight studies [11–17,20] reported calibration metrics. In the internal and external validation reports, seven studies [10–12,14,15,17,19] conducted internal validation, while three studies focused on model construction [14,17,19] performed external validation. The summary of findings revealed that the predictive models encompassed various factors, including sociodemographic factors, disease-related factors, treatment-related factors, vascular factors, and biochemical indicators, totaling between three and 28 variables. The top five frequently reported predictive variables were age (n = 8), gender (n = 4), presence of diabetes (n = 4), dialysis hypotension (n = 4), and platelet count (n = 3).Specifics are outlined in Table 3 and Table 4.

**Table 3:** Performance of models predicting.

Study	Modelling method	AUC	Calibration method	Validation method
Weng^11^	Cox proportional risk regression model, Random Fores	——	Calibration curve	Internal validation
Gong^12^	Logistic	0.792	H-L goodness-of-fit test with calibration curves	Internal validation
Wang^13^	Logistic	0.934	H-L goodness-of-fit test	Internal validation
Liang^14^	Logistic	0.799	Calibration curve, H-L goodness-of-fit test	External validation
Che^15^	Logistic	0.725	Calibration plots, decision curves	Internal validation
Peralta^16^	XGBoost	0.80	Consistency statistics and calibration plots	Internal validation
Wang^17^	Logistic	0.714	Calibration curve	External validation
Wongmahisorn^18^	Logistic	0.810	——	Internal validation
Qian^19^	Logistic	0.530	——	External validation
Eslami^20^	Logistic	0.731	H-L goodness-of-fit test	Internal validation
Masengu^21^	Logistic	0.530	——	——

**Table 4:** Inclusion of model predictors and applicable populations.

Study	Number of predictor variables	Specific variables	Applicable population and limitations
Weng^11^	6	1, 2, 3, 4, 5, 6	Applicable to MHD patients, but it is a single-centre study with a small sample size
Gong^12^	5	1, 4, 7, 8, 9	Suitable for MHD patients, but small sample size and lack of external validation
Wang^13^	7	4, 10, 11, 12, 13, 14,15	Applies to MHD patients on regular dialysis for ≥6 months, but sample size is small and external validation is lacking
Liang^14^	5	1, 4, 7, 16, 17	Applies to patients with MHD, but is a retrospective study and external validation of the small sample size included (only 84 cases), which may reduce the predictive efficacy of the model
Che^15^	7	1, 3, 7, 9, 18, 19, 20	Applies to MHD patients but small sample size and lack of external validation
Peralta^16^	28	4, 5, 7, 11, 10, 18, 21, 22, 23, 24, 25, 26, 27, 28, 29, 30, 31, 32, 33, 34, 35, 36, 37, 38, 39, 40, 41, 42, 43	Applicable to MHD patients, but lacks external validation
Wang^17^	4	4, 16, 17, 28	Applies to MHD patients, but sample size is small
Wongmahisorn^18^	3	7, 42, 43	Applies to MHD patients on regular dialysis for ≥12 months, but sample size is small and external validation is lacking
Qian^19^	7	4, 10, 43,44, 42, 45	Applies to older MHD patients aged ≥67 years and has poor model predictive performance
Eslami^20^	6	11, 46, 47, 48, 49	Applies to MHD patients but lacks external validation
Masengu^21^	6	4, 10, 43, 44, 48, 50	Poor model performance and lack of internal and external validation

Note: Influencing factors:1 = Platelet count; 2 = Triglycerides; 3 = Fibrinogen; 4 = Age; 5 = Albumin; 6 = Calcium; 7 = Comorbid diabetes; 8 = Hypotension on dialysis; 9 = Calcium-phosphorus product; 10 = Gender; 11 = Diabetes; 12 = Hyperlipidaemia; 13 = Anastomotic diameter; 14 = Blood phosphorus; 15 = D-dimer; 16 = Hypotension; 17 = Low-density lipoprotein; 18 = C-reactive protein; 19 = Puncture method; 20 = Post-dialysis hypotensive status; 21 = Temperature; 22 = Age on dialysis; 23 = Duration of AVF use; 24 = Ferritin; 25 = Glucose; 26 = PTH; 27 = Duration of dialysis treatment; 28 = Ultrafiltration volume; 29 = Effective blood flow; 30 = Effective exchanged blood volume; 31 = Kt/V; 32 = Recirculation rate; 33 = Number of days of vascular access use; 34 = Duration of AVF establishment; 35 = Number of vascular accesses established in the last six months; 36 = Number of times dialysis was performed with an AVF in the last six months; 37 = Venous pressure; 38 = Ambulatory arterial pressure; 39 = Number of previous AVF failures; 40 = Time since last AVF failure; 41 = Number of previous thrombosis; 42 = History of other vascular access; 43 = History of vascular access complications;43 = Primary disease; 44 = Comorbidities; 45 = Nursing care; 46 = Hypertension; 47 = Comorbid COPD; 48 = AVF location; 49 = Vein diameter; 50 = History of anticoagulant use.

### 3.5 Quality assessment of the models

All 11 included studies were assessed as having a “high risk of bias,” with two studies classified as having “good applicability.” Specifics are outlined in Table 5.

**Table 5:** Risk of bias and applicability evaluation of included studies.

Study	Risk of bias				Risk of applicability			Overall risk	
	Participants	Predictors	Outcome	Analysis	Participants	Predictors	Outcome	Risk of Bias	Applicability
Weng^11^	-	?	?	-	+	+	+	-	+
Gong^12^	-	?	?	-	+	+	+	-	+
Wang^13^	-	?	?	-	-	+	+	-	-
Liang^14^	-	?	-	-	+	+	+	-	+
Che^15^	-	-	?	-	+	+	+	-	+
Peralta^16^	-	-	-	-	+	-	+	-	+
Wang^17^	-	?	?	-	+	+	+	-	+
Wongmahisorn^18^	-	?	-	-	-	-	+	-	-
Qian^19^	-	-	-	-	-	-	+	-	-
Eslami^20^	+	+	?	-	+	+	+	-	+
Masengu^21^	+	+	?	-	+	+	+	-	+

Note: + : low risk of bias/high applicability; -: high risk of bias/low applicability;?: risk of bias unclear/applicability unclear.

#### 3.5.1 Bias in study population domain.

In the study population domain, nine studies [10,11,13,14,16–18] were rated as having a high risk of bias. This classification primarily resulted from the retrospective nature of the case analyses, which relied on existing case data.Important predictive factors for AVF failure in maintenance MHD patients may not be fully captured through medical records alone, potentially introducing recall bias and resulting in significant discrepancies between predicted and actual outcomes.

#### 3.5.2 Bias in predictive factors domain.

Within the predictive factors domain, three studies [14,15,18] were classified as having a high risk of bias, while six studies [10–13,17] had unclear bias risks, and two studies [19,20] were rated as low risk of bias. The three multi-center studies [14,15,18] did not report whether the measurement methods for predictive factors were consistent across different centers. In six retrospective studies [11–14,18], it remained unclear whether the assessments of predictive factors were conducted without knowledge of the outcome data, which could have led to interference from prior events, resulting in an “unclear” bias rating. The two prospective studies [19,20] collected data prior to the occurrence of outcomes, suggesting the potential use of blinding methods.

#### 3.5.3 Bias in outcomes domain.

Four studies [13,15,17,18] exhibited a high risk of bias in the outcomes domain, while seven studies [10–12,14,16,19,20] presented unclear bias risks. The three retrospective studies [15,17,18] may have introduced bias in defining and measuring predictive outcomes, and one study [14] did not predefine its outcome measures. Furthermore, seven studies [10–12,14,16,19,20] failed to clarify whether the outcome assessors were blinded.

#### 3.5.4 Bias in data analysis domain.

All 11 included studies were rated as having a high risk of bias in the data analysis domain. Six model development studies [11–15,17] exhibited insufficient sample sizes, with fewer than 20 events per variable, and subsequent adjustments were not made, potentially resulting in model outcomes that deviate significantly from reality and leading to overfitting. One study [19] did not report its variable selection methods. In the treatment of variables, a study by Weng et al. [11] converted some continuous variables into categorical variables, which may have resulted in a loss of information. Additionally, ten studies [11–15,17–21] did not report their methods for handling missing data, which may have resulted in an overestimation of the model’s discriminatory ability. Three studies [13,16,17] based their variable selection first on univariate analysis followed by multivariate logistic regression analysis, potentially overlooking important predictive factors, thereby introducing bias. With regard to model discrimination and calibration, only six studies [10,11,13–16] provided evaluations. One model construction study [20] did not perform internal validation.

#### 3.5.5 Applicability assessment.

Among the 11 studies included in this review, eight were assessed as having a low risk of applicability. Two studies [13,18] focused on patients undergoing regular dialysis for at least 6 months and 12 months, respectively, while one study [19] was limited to patients aged 67 years and older, contributing to a higher applicability risk.

### 3.6 Statistical analysis results

Due to insufficient data, the studies by Weng [11] and Eslami [20] were excluded, leaving a total of 9 studies included in the meta-analysis. The original models had AUC values ranging from 0.53 to 0.934. The studies [22] indicated that models with AUC values below 0.60 exhibited weak predictive power and may not be suitable for identifying high-risk patients in clinical practice. Models with AUC values between 0.60 and 0.75 demonstrate moderate predictive capability and could be useful in some clinical decision-making scenarios. However, these models should be used in conjunction with other clinical data. Models with AUC values greater than 0.75 are considered to have higher clinical value.In this study, the AUC estimate from the random-effects model was 0.74 (95% CI: 0.62–0.86), indicating that there is still potential for improvement in predictive accuracy.

**Fig 2:**
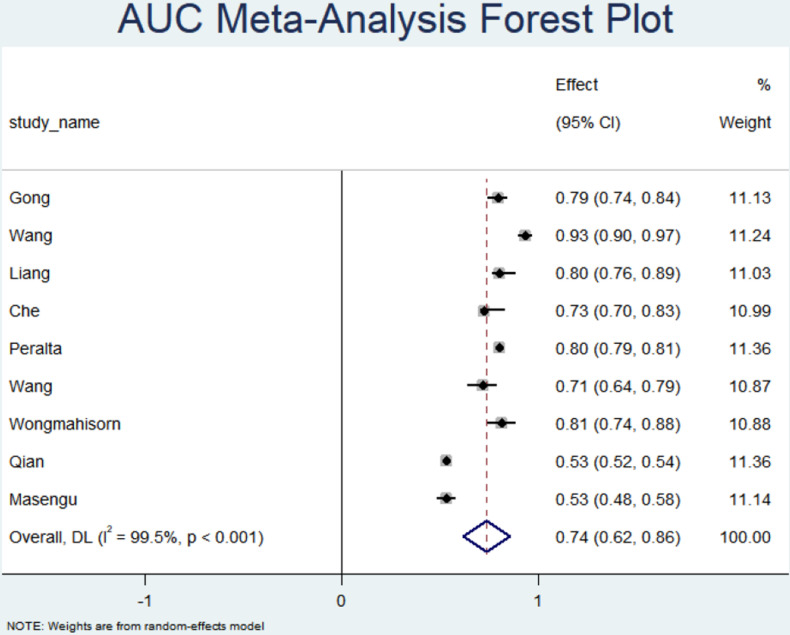
Model AUC forest plot.

**Fig 3:**
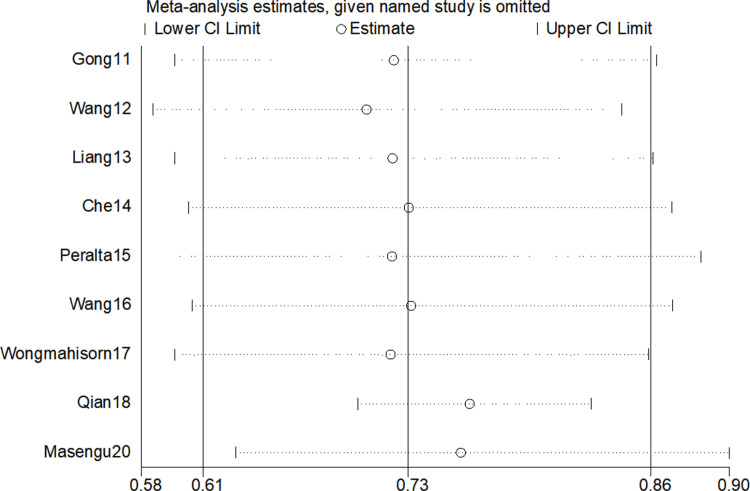
Sensitivity analysis.

Moreover, the meta-analysis revealed high heterogeneity among the study results (I² = 99.5%, P > 0.05), and Egger’s test suggested publication bias between the studies (P = 0.019). Sensitivity analysis results showed an overall combined AUC of 0.72 (95% CI: 0.713–0.727). After excluding the study by Peralta et al. [16], the AUC decreased to 0.616 (95% CI: 0.606–0.628), which was a significant reduction, likely due to the large sample size of the study (n = 13,369), the machine learning modeling method (XGBoost), and the relatively high original AUC (AUC = 0.80). Conversely, after excluding the study by Qian et al. [19], the AUC increased to 0.803 (95% CI: 0.795–0.812), a significant improvement. This may be attributed to the fact that this study was an external validation, and its original AUC was relatively low (AUC = 0.53), which had pulled down the overall combined result. (Meta-analysis and sensitivity analysis results are presented in Figs 2 and 3).

### 3.7 GRADE evidence quality assessment

The outcome indicators were evaluated for quality based on the GRADE system. Observational studies were initially classified as low-quality evidence according to GRADE guidelines. However, evidence could be upgraded when substantial effect sizes were demonstrated (e.g., AUC ≥ 0.8). Among the included studies, three models (Wang et al. [13], Peralta et al. [16], and Wongmahisorn et al. [18]) were upgraded by one level due to their exceptional predictive performance (AUC ≥ 0.8). Ultimately, only studies with rigorous methodology and validated performance, such as Peralta et al. [16], maintained a moderate-quality rating, while the majority of evidence was downgraded to low or very low quality due to limitations in bias control, imprecision, or applicability concerns. Detailed results are provided in Table 6.

**Table 6:** GRADE Grading Results.

Study	Estimation of Quality					Upgrade Factors	Quality of Evidence
	Risk of Bias	lmprecision	Inconsistency	Indirectness	Publication Bias		
Weng^11^	−1^a^	−1^b^	0	−1^d^	−1^e^	None	Very low
Gong^12^	−1^a^	−1^b^	0	−1^d^	−1^e^	None	Very low
Wang^13^	−1^a^	−1^b^	0	0	−1^e^	Yes	low
Liang^14^	−1^a^	0	0	0	−1^e^	None	low
Che^15^	−1^a^	−1^b^	0	−1^d^	−1^e^	None	Very low
Peralta^16^	0	0	0	0	−1^e^	Yes	moderate
Wang^17^	−1^a^	−1^b^	0	0	−1^e^	None	Very low
Wongmahisorn^18^	−1^a^	−1^b^	0	0	−1^e^	Yes	low
Qian^19^	−1^a^	0	−1^c^	0	−1^e^	None	Very low
Eslami^20^	−1^a^	0	0	−1^d^	−1^e^	None	Very low
Masengu^21^	−1^a^	0	0	−1^d^	−1^e^	None	Very low

Note: a. Single-center or retrospective study; b. Sample size too small; c. Direction of results is opposite or the effect size differs significantly (AUC < 0.6); d. Study population differs from the target clinical population (e.g., age, disease stage, comorbidities); e. Publication bias not discussed, assumed to be present.

## 4 Discussion

### 4.1 High risk of bias in AVF failure prediction models for MHD patients

This study evaluated eleven prediction models, yielding area under the curve (AUC) values ranging from 0.53 to 0.934. While nine of these models demonstrated AUCs greater than 0.7, indicating relatively strong predictive performance, the overall risk of bias across the studies was notably high, particularly within the analytical domain. The primary reasons for this are outlined below.Insufficient Sample Size: To prevent overfitting during model development, the Prediction Model Risk of Bias Assessment Tool (PROBAST) [8] recommends that the number of outcome events should be at least twenty times the number of candidate predictors. This implies that each variable should have more than 20 corresponding events. However, six studies included in this research [11–15,17] did not meet this criterion. Future model development studies should ensure adequate sample sizes to improve robustness.Inadequate Handling of Missing Data: One study [23] highlighted that improper handling of missing data can lead to the loss of valuable information, resulting in biased model outcomes. Among the eleven models analyzed, only one study [16] reported the methodology employed for managing missing data. Future research should adopt appropriate techniques for handling missing values, taking into account the missing data mechanism, distribution of values, and correlations among variables, as these factors significantly influence the performance of imputation methods. Common approaches for addressing missing values include statistical imputation techniques such as mean, median, or mode imputation; linear regression imputation; and multiple imputation, as well as machine learning-based methods like decision trees, random forests, k-nearest neighbors, support vector machines, XGBoost, and artificial neural networks [24]. Guo et al. [25] found that employing random forests for data imputation yielded the best overall performance in constructing Cox proportional hazards models. Additionally, the PROBAST guidelines recommend utilizing multiple imputation methods for missing data.Lack of Reporting on Model Fit: Five studies [13,18–21] did not report calibration results for their models, thereby hindering the assessment of the accuracy and reliability of the predictive models. This lack of reporting complicates subsequent research aimed at refining these models.Incomplete Model Performance Evaluation: One model-building study [21] did not conduct internal or external validation, while six studies [10,12,14,15,17,19] failed to perform external validation. These omissions potentially affect the generalizability and applicability of the models.To mitigate these issues in future research, we recommend the following: 1.Conducting more prospective, multicenter studies with larger sample sizes. 2.Selecting appropriate methods for handling missing data while emphasizing the importance of reporting calibration results. 3.Adhering to the PROBAST guidelines and standards for individual prognosis and diagnostic prediction model studies [26] when developing AVF failure prediction models. This approach should include both internal and external validation to enhance the accuracy and reliability of the models, thereby minimizing the risk of bias

### 4.2 Differences and commonalities in the selection of predictive factors for AVF failure risk in MHD patients

In clinical practice, the availability of predictive factors and the ease of model application are critical concerns for healthcare professionals. This study examined 11 predictive models that included between 8 and 109 candidate predictive factors, ultimately resulting in models comprising 3–28 variables. These variables encompassed demographic factors, disease-related factors, treatment-related factors, vascular factors, and biochemical indicators.The findings revealed variability in the predictive factors included across different AVF failure risk prediction models. However, upon summarizing the data, the five most frequently identified predictive factors were age, gender, comorbid diabetes, dialysis hypotension, and platelet count. These results suggest that healthcare providers should pay special attention to elderly patients, women, individuals with diabetes, those prone to dialysis-induced hypotension, and patients with elevated platelet counts.End-stage renal disease is a chronic condition characterized by a complex etiology and prolonged treatment duration. Alongside long-term MHD, patients often present with comorbid conditions that can interact and complicate treatment. Several studies have highlighted that MHD patients with comorbid diabetes face a significantly increased risk of AVF failure [12–14]. This risk is primarily attributed to the accumulation of glycation end-products, endothelial damage, decreased vascular elasticity, sluggish blood flow, and increased platelet aggregation, which can lead to thrombosis within the vessels [27]. Elevated platelet counts may contribute to AVF failure by increasing blood viscosity and exacerbating atherosclerosis, thereby heightening the risk of thrombosis [10,11,13]. Additionally, Chen et al. [28] identified dialysis hypotension as an independent risk factor for AVF failure. Following episodes of hypotension, reduced wall tension in the vessels, substantial decreases in local blood flow, and even stagnation can lead to thrombus formation, resulting in AVF stenosis or occlusion.Thus, although the mechanisms by which these three predictive factors lead to AVF failure differ, they ultimately converge on the outcome of thrombus formation. Consequently, healthcare professionals should design individualized intervention strategies targeting modifiable risk factors: 1.Patient Education: Enhance education for patients at high risk of AVF failure to help them recognize the importance of controllable factors, thereby improving self-management and treatment adherence. 2.Diabetes Management: Strengthen blood glucose monitoring for patients with diabetes, ensuring that glucose levels are maintained within target ranges, and implement effective dietary and nutritional management tailored to their needs. 3.Hypotension Prevention: Implement early measures to prevent dialysis-induced hypotension through timely assessment, enhanced blood pressure monitoring, and the proactive development of risk management plans. 4.Biochemical Monitoring: Increase the frequency of biochemical testing and standardize anticoagulant usage. Tailor management and adjust medication regimens based on individual patient circumstances to mitigate the hypercoagulable state of the blood.By adopting these strategies, healthcare providers can better manage the risk factors associated with AVF failure in MHD patients, ultimately enhancing patient outcomes.

### 4.3 Risk prediction model for AVF failure in MHD patients requires further optimization

Research on risk prediction models for AVF failure in patients undergoing MHD has emerged relatively recently, and the clinical effectiveness of these models has been suboptimal. This shortcoming may be attributed to several factors, including a lack of standardized outcome definitions, model complexity, and insufficient external validation.Firstly, the definitions and criteria for AVF failure vary across different studies, making it challenging to compare constructed models and limiting their applicability in clinical settings. The KDOQI guidelines introduced the concept of “access blood flow dysfunction,” which refers to clinically significant abnormalities in AVF blood flow or patency caused by stenosis, thrombosis, or related pathological changes, while excluding non-stenotic or thrombotic lesions such as aneurysmal dilation. Additionally, the emergence of puncture difficulties has been identified as a clinical indicator of AVF dysfunction [29]. However, this definition does not clearly specify the parameters for vascular diameter or natural blood flow through the fistula. Furthermore, the studies included in this research did not reach a consensus on the criteria for assessment. To achieve consistency in clinical diagnosis and to provide a unified benchmark for the development, validation, application, and interpretation of associated risk prediction models, future efforts should focus on clarifying the definition of AVF failure and establishing standard criteria.Secondly, most existing risk prediction models for AVF failure are based on single-center and small-sample studies. Although there have been multi-center, large-sample prospective studies, the applicability of these models to different countries and regions remains to be validated due to ethnic and cultural differences. Moreover, many of the predictive models analyzed in this study have not undergone external validation, which limits their predictive capabilities and scope of application [29]. It is recommended that future research on AVF failure prediction models include multi-center external validation to enhance the generalizability and applicability of the models.Lastly, with advancements in artificial intelligence (AI) within the medical field, various machine learning algorithms—such as decision trees, random forests, support vector machines, and neural networks—have been widely utilized in constructing prediction models for other populations and diseases. It is well-established that risk prediction models developed using AI algorithms often outperform traditional logistic regression models [30]. However, among the eleven models included in this study, most still primarily rely on logistic regression, with relatively few applications of machine learning techniques in predicting AVF failure risk in MHD patients. Therefore, to ensure the rigor and applicability of AVF failure risk prediction models, it is suggested that future studies adopt a multi-center, large-sample, prospective design that simplifies model structures and feature selection while employing more optimal statistical and modeling methods tailored to local conditions.

## 5. Limitations

To minimize publication bias, this study conducted a comprehensive search of both Chinese and English databases; however, certain limitations are unavoidable. Firstly, the number of included studies is limited, with the majority being conducted in China, potentially introducing publication bias,which could affect the external validity of our findings. Secondly, the exclusion of conference abstracts may impact the findings of this study. Conference abstracts, although sometimes less rigorously reviewed, may contain valuable preliminary data or emerging research that could contribute to a more comprehensive understanding of risk prediction models. Lastly, while several models addressing thrombosis formation and maturation prediction in MHD patients have emerged, this research specifically reports on models related to failure risk prediction to facilitate a more in-depth analysis of these results.While the focus on failure risk prediction provides a more in-depth analysis of this aspect, it is important to acknowledge that models predicting thrombosis formation and maturation are also crucial for the management of AVF in MHD patients.Future studies should incorporate more diverse populations from various geographical regions, systematically review conference proceedings to minimize publication bias and update information, and include a broader range of models addressing different stages of AVF dysfunction to enhance the generalizability and comprehensiveness of risk prediction for AVF failure.

## 6. Conclusion

The findings of this systematic review indicate that while the eleven risk prediction models for AVF failure MHD patients demonstrate reasonably good predictive performance, they are associated with a significant overall risk of bias, indicating that the quality of these models requires substantial enhancement. Furthermore, the majority of the models included in this study lack external validation. This absence, coupled with cultural and demographic variations across different countries, limits their generalizability and clinical applicability.Future researchers are encouraged to adhere to the TRIPOD statement as a guideline to standardize the design and reporting processes in model development. Additionally, they should consider integrating machine learning algorithms to optimize model performance. Furthermore, conducting multicenter, large-sample external validations will enhance the generalizability of these models, ultimately providing reliable support for clinical decision-making. Such approaches aim to reduce patient suffering, improve quality of life, and enhance overall prognosis.

## Supporting information

S1 Checklist PRISMA checklist. (DOCX)

S1 Appendix Search strategy. (DOCX)

S2 Appendix All studies identified in the literature search. (DOCX)
