# Supplementary material for: Systematic review of risk prediction models for arteriovenous fistula dysfunction in maintenance hemodialysis patients
Source: PLoS One. 2025 May 19;20(5):e0324004. doi: 10.1371/journal.pone.0324004 (PMC12087999; doi:10.1371/journal.pone.0324004)
Supplement: S1 Appendix — (DOCX) [file pone.0324004.s002.docx]

# **S1 Appendix**

# **S1 Table:** Search strategy

| **PubMed 678 (Initial search Aug 2024, updated Jan 2025)** |
| --- |
| ((((((((((renal dialysis[MeSH Terms]) OR (Dialyses, Renal[Title/Abstract])) OR (Renal Dialyses[Title/Abstract])) OR (hemodialysis[MeSH Terms])) OR (maintenance hemodialysis[Title])) OR (MHD[Title])) OR (chronic kidney failure[MeSH Terms])) OR (Renal Replacement Therapy[Title/Abstract])) AND (((((arteriovenous fistula[MeSH Terms]) OR (Arteriovenous Fistulas[Title/Abstract])) OR (Arteriovenous Aneurysm[Title/Abstract])) OR (autogenous arteriovenous fistula[Title/Abstract])) OR (AVF[Title/Abstract]))) AND ((((((((Dysfunction[Title/Abstract]) OR (Failure[Title/Abstract])) OR (failure to thrive[MeSH Terms])) OR (Thrombosis[MeSH Terms])) OR (thrombus*[Title/Abstract])) OR (embolisms[MeSH Terms])) OR (Venous Thromboembolism[Title/Abstract])) OR (stenosis[Title/Abstract]))) AND (((risk assessment[MeSH Terms] OR risk factors[MeSH Terms]) OR (predict*[Title/Abstract] OR"prediction model"[Title/Abstract] OR "risk prediction"[Title/Abstract] OR "risk assessment"[Title/Abstract] OR "risk factors"[Title/Abstract]))) 678 |
| **Embase 177 (Initial search Aug 2024, updated Jan 2025)** |
| (dialysis:ab,ti OR hemodialysis:ab,ti OR 'renal replacement therapy':ab,ti OR 'maintenanc hemodialysis':ab,ti OR hemodiafiltration:ab,ti) AND ('arteriovenous fistula':ab,ti OR 'autogenous arteriovenous fistula':ab,ti OR avf:ab,ti OR 'arteriovenous access':ab,ti) AND (dysfunction:ab,ti OR failure:ab,ti OR 'failure to thrive':ab,ti OR thrombosis:ab,ti OR ('stenosis, occlusion':ab,ti AND obstruction:ab,ti)) AND ('risk assessment':ab,ti OR 'prediction model':ab,ti OR 'risk prediction model':ab,ti OR 'prediction':ab,ti) |
| **Web of Science Core Collection 576 (Initial search Aug 2024, updated Jan 2025)** |
| #1 (((((TS=(dialysis )) OR TS=(renal dialysis)) OR TS=(hemofiltration)) OR TS=(maintenance hemodialysis)) OR TS=(MHD)) OR TS=(Renal Replacement Therapy)  #2 ((((TS=("Arteriovenous Fistula")) OR TS=("Arteriovenous Aneurysm")) OR TS=("autogenous arteriovenous fistula")) OR TS=(AVF))  #3 ((((((((TS=(Dysfunction)) OR TS=(Failure)) OR TS=(Failure to Thrive)) OR TS=(Thrombosis)) OR TS=(Bloiod Clot)) OR TS=(Venous Thrombosis)) OR TS=(thrombus*)) OR TS=(microthrombus)) OR TS=(stenosis)  #4 (((TS=("risk assessment")) OR TS=("prediction model")) OR TS=("risk prediction")) OR TS=(predict*)  #1 AND #2 AND #3 AND #4 |
| **The Cochrane Library 57 (Initial search Aug 2024, updated Jan 2025)** |
| (Dialysis):ti,ab,kw OR (renal dialysis):ti,ab,kw OR (Renal Replacement Therapy):ti,ab,kw OR (hemodialysis):ti,ab,kw OR (maintenanc hemodialysis):ti,ab,kw OR (hemodiafiltration):ti,ab,kw OR (hemofiltration):ti,ab,kw OR (Extracorporeal Dialysis):ti,ab,kw AND (Arteriovenous Fistula):ti,ab,kw OR (Arteriovenous Aneurysm):ti.ab,kw OR (Ateriovenous Shunt, Surgical.t.ab,kw AND (anteriovenous acces ).ti ab .kw OR(vascular next access):ti,ab,kw OR ("Fistularia"):ti,ab,kw OR (autogenous arteriovenousfistula):ti,ab,kw OR (AVF):ti,ab,kw AND ("dysfunction"):ti,ab,kw OR (Failure):ti,ab,kw OR (Failure to Thrive):ti,ab,kw OR (thrombus* ):ti, ab,kw OR (stenosis): ti.ab.kw OR 'vascular access thromboses" .ti ab kw OR (Bloiod Clotl;ti ab kw OR "embolism";ti ab kw OR i"thromboembolism" ti ab kw OR (vascular access stenosis):ti,ab,kw AND ("risk assessment"):ti,ab,kw OR ("prediction model"):ti,ab,kw OR (risk prediction model):ti,ab,kw OR (predict):ti,ab,kw |
| **CNKI 97 (Initial search Aug 2024, updated Jan 2025)** |
| (SU = 透析 + 血液透析 + 维持性血液透析) AND (SU = 血管通路 + 内瘘 + 动静脉内瘘 + 自体动静脉内瘘 ) AND (SU = 功能不良 + 功能不全 + 功能障碍 + 失功 + 栓塞 + 狭窄 + 血栓 + 闭塞) AND (SU=预测 + 风险预测 + 预测模型 + 风险评估 + 风险评分 + 模型 + 风险预测模型 + 预测模型 + 诊断模型 + 临床预测模型 + 风险因素 + 列线图) |
| **Chinese Biomedical Literature Database 157 (Initial search Aug 2024, updated Jan 2025)** |
| (透析 OR 血液透析 OR 维持性血液透析) AND (动静脉内瘘 OR 自体动静脉内瘘) AND (功能不良 OR 功能障碍 OR 失功 OR 栓塞 OR 狭窄 OR 血栓) AND (预测 OR 风险预测 OR 预测模型 OR 风险评估 OR 风险评分 OR 模型 OR 风险预测模型 OR 预测模型 OR 诊断模型 OR 临床预测模型 OR 风险因素 OR 列线图) |
| **Wanfang database 96 (Initial search Aug 2024, updated Jan 2025)** |
| 主题:(（透析 OR 血液透析 OR 维持性血液透析）) and 主题:((动静脉内瘘 OR 自体动静脉内瘘) ) and 主题:((功能不良 OR 功能障碍 OR 失功 OR 栓塞 OR 狭窄 OR 血栓) ) and 主题:(((预测 OR 风险预测 OR 预测模型 OR 风险评估 OR 风险评分 OR 模型 OR 风险预测模型 OR 预测模型 OR 诊断模型 OR 临床预测模型 OR 风险因素 OR 列线图))) |
| **Vip Joural database 47 (Initial search Aug 2024, updated Jan 2025)** |
| (((((题名或关键词=血液透析 OR 题名或关键词=血透) OR 题名或关键词=MHD) AND (((题名或关键词=血管通路 OR 题名或关键词=内瘘) OR 题名或关键词=动静脉内瘘) OR 题名或关键词=自体动静脉内瘘)) AND (((((((题名或关键词=功能不良 OR 题名或关键词=功能不全) OR 题名或关键词=功能障碍) OR 题名或关键词=失功) OR 题名或关键词=栓塞) OR 题名或关键词=狭窄) OR 题名或关键词=血栓) OR 题名或关键词=闭塞)) AND ((((((((((题名或关键词=预测 OR 题名或关键词=风险预测OR 预测模型) OR 题名或关键词=风险评估) OR 题名或关键词=风险评分) OR 题名或关键词=模型) OR 题名或关键词=风险预测模型) OR 题名或关键词=预测模型) OR 题名或关键词=诊断模型) OR 题名或关键词=临床预测模型) OR 题名或关键词=风险因素) OR 题名或关键词=列线图)) |
| **Clinical Trials.gov 167 (Initial search Jan 2025)** |
| （arteriovenous fistula OR AVF OR Arteriovenous Aneurysm) AND ((Dysfunction OR Failure OR stenosis) AND (risk assessment OR risk factors OR predict) |

**S2 Table: Data for forest plot**

| **Study** | **AUC** | **lower_ci** | **upper_ci** |
| --- | --- | --- | --- |
| Gong^11^ | 0.792 | 0.741 | 0.843 |
| Wang^12^ | 0.934 | 0.897 | 0.971 |
| Liang^13^ | 0.799 | 0.762 | 0.886 |
| Che^14^ | 0.725 | 0.703 | 0.833 |
| Peralta^15^ | 0.800 | 0.790 | 0.810 |
| Wang^16^ | 0.714 | 0.639 | 0.789 |
| Wongmahisorn^17^ | 0.810 | 0.736 | 0.885 |
| Qian^18^ | 0.530 | 0.520 | 0.540 |
| Masengu^20^ | 0.530 | 0.480 | 0.580 |
