# Supplementary material for: Systematic review of risk prediction models for arteriovenous fistula dysfunction in maintenance hemodialysis patients
Source: PLoS One. 2025 May 19;20(5):e0324004. doi: 10.1371/journal.pone.0324004 (PMC12087999; doi:10.1371/journal.pone.0324004)
Supplement: S2 Appendix — (DOCX) [file pone.0324004.s003.docx]

**Table S1 Records excluded based on title and abstracts (n = 1079)**

| No. | Reference | Reason of exclusion |
| --- | --- | --- |
|  | Hsin CH, Yang HT, Feng PC, Su TW, Yu SY, Ko PJ. Drug-coated balloon for early recurrent arteriovenous fistula dysfunction. J Vasc Access. 2024 Sep;25(5):1560-1566. doi: 10.1177/11297298231166426. | Not relevant to the topic |
|  | DU Shutong,MA Weihua,BAI Weiwei,et al. Relationship between serum C1q/tumor necrosis factor-related protein 9 and vascular hemophilic factor levels and thrombosis of arteriovenous endovascular fistulae in hemodialysis patients[J]. Journal of Translational Medicine,2024,13(06):857-862. | Inconsistent with study population |
|  | Wang, Bingyue. Study of shear force on KLF2, Cav-1 and their signaling pathways in venous endothelial cells of arteriovenous fistula[D]. Tianjin Medical University,2020.DOI:10.27366/d.cnki.gtyku.2020.000170. | Not relevant to the topic |
|  | YANG Jiemin. Efficacy of balloon angioplasty for the treatment of peri-anastomotic stenosis of autologous arteriovenous endovascular fistula based on different accesses[D]. Guilin Medical College,2024.DOI:10.27806/d.cnki.gglyx.2024.000086. | Not relevant to the topic |
|  | LIU Juan,YANG Mingzheng,CHEN Zhengfang,WU Xixi. Correlation between arteriovenous endovascular fistula stenosis and neutrophil elastase level in hemodialysis patients[J]. Journal of Clinical and Experimental Medicine,2023,22(8):821-824.DOI:10.3969/j.issn.1671-4695.2023.08.010. | Not relevant to the topic |
|  | ZHANG Yue,ZHANG Xiaogong,MI Aihong,et al. Predictive value of plasma ADAMTS-13 combined with Gas6 on AVF thrombosis in uremic patients on maintenance hemodialysis[J]. Journal of Clinical Psychosomatic Diseases,2024,30(03):46-50. | Inconsistent with study population |
|  | Yao Yao.Predictive value of Caprini thrombosis risk assessment scale for thrombotic events after AVG surgery in hemodialysis patients[J]. China Blood Purification,2024,23(05):382-386. | Inconsistent with study population |
|  | Dong Jinxiu.Study on the correlation between NLR, PLR and stenosis of arteriovenous endovascular fistula in maintenance hemodialysis patients[D]. Shanghai Jiao Tong University,2016. | Not relevant to the topic |
|  | Wang YF. A retrospective study on the comparison of tunneled hemodialysis catheter application in right external jugular vein and left internal jugular vein[D]. Zhengzhou University,2016. | Not relevant to the topic |
|  | ZHANG Zhoucang. Study on the relationship between dialysis blood flow rate and clinical prognosis of maintenance hemodialysis patients[D]. Hebei Medical University,2024.DOI:10.27111/d.cnki.ghyku.2024.000080. | Not relevant to the topic |
|  | CHEN Min,ZHANG Shanshan,ZHANG Guoxin,et al. Study on the relationship of serum Irisin and MBL with inflammatory factors and arteriovenous endovascular fistula thrombosis in maintenance hemodialysis patients[J]. Advances in Modern Biomedicine,2024,24(06):1067-1071+1037.DOI:10.13241/j.cnki.pmb.2024.06.011. | Inconsistent with study population |
|  | ZHANG Feng, JIANG Hongzhen. Progress of prevention and treatment of autologous arteriovenous endovascular fistula dysfunction[J]. Journal of Practical Medicine,2024,40(13):1767-1770 | Not relevant to the topic |
|  | ZHOU Li. Relationship between water loading parameters and stenosis of autologous arteriovenous endovascular fistula in patients on maintenance hemodialysis[J]. Journal of Hebei North College (Natural Science Edition),2024,40(12):13-16. | Not relevant to the topic |
|  | HE Ping,XU Rui,HUA Qiu Ju,et al. Predictive analysis of erythrocyte distribution width and platelet ratio on arteriovenous endovascular fistulae failure in maintenance hemodialysis patients with end-stage renal disease[J]. Chinese Journal of Continuing Medical Education,2024,47(11):988-994. DOI:10.3760/cma.j.cn115455-20240322-00264. | Not relevant to the topic |
|  | Ouyang Fenjing,Luo Hongmei,Zhu Jianqiang. Factors contributing to the occurrence of AVF loss of function in CRF patients treated with MHD and strategies for prevention[J]. China Health Standard Management,2024,15(21):117-120,124. DOI:10.3969/j.issn.1674-9316.2024.21.027. | Not relevant to the topic |
|  | WANG Mingbo,NI Xiaona,ZHU Lin,et al. Predictive value of serum Krüppel-like factor 2 and angiotensin II receptor-like 1 endogenous ligand 13 for stenosis of autologous arteriovenous endovascular fistula in hemodialysis patients[J]. Chinese medicine,2024,19(01):79-83. | Not relevant to the topic |
|  | WANG Huiying,YU Hangying,FANG Lihong. Analysis of the predictive role of arteriovenous pressure monitoring on AVF stenosis in maintenance hemodialysis patients[J]. Zhejiang Trauma Surgery,2023,28(12):2338-2340. | Not relevant to the topic |
|  | FENG Qing. Analysis of the causes and prevention of internal fistula occlusion in maintenance hemodialysis patients[C]. Chinese Society of Integrative Medicine, Renal Disease Committee. Compendium of abstracts from the 2018 Annual Academic Conference of the Renal Diseases Specialized Committee of the Chinese Society of Integrative Medicine and Western Medicine. The First Hospital of Dalian Medical University;,2018:1. | Conference abstracts,etc |
|  | Shen J,Luo ML. Causes analysis and nursing countermeasures of arteriovenous endovascular fistula stenosis and thrombosis in hemodialysis[C]. Renal Disease Specialized Committee of the Chinese Society of Integrative Medicine and Western Medicine. Compendium of abstracts from the 2018 Annual Academic Conference of the Renal Diseases Specialized Committee of the Chinese Society of Integrative Medicine. Zhejiang Provincial Tongde Hospital;,2018:1. | Conference abstracts,etc |
|  | Yu JJ,Zhao LM,Yu T,et al. Risk factor analysis and risk model construction of internal fistula loss in maintenance hemodialysis patients[J]. Huaihai Medicine,2023,41(05):463-466+471.DOI:10.14126/j.cnki.1008-7044.2023.05.006. | Full text not available |
|  | WANG Yan,ZHANG Aixia,WANG Zihui,et al. Relationship between basic fibroblast growth factor, serum and glucocorticoid-induced protein kinase 3 expression and arteriovenous endovascular fistulae failure in hemodialysis patients[J]. Journal of Xinxiang Medical College,2023,40(09):851-855. | Not relevant to the topic |
|  | ZHANG Haiyan. Evaluation value of ultrasound hemodynamic parameters on thrombus and stenosis of arteriovenous endovascular fistula in hemodialysis patients[J]. Imaging Research and Medical Application,2023,7(13):56-59. | Inconsistent with study population |
|  | HUANG Zhenxia,ZHANG Yuxiang,WANG Can,et al. Predictive value of serum chitinase-3-like protein 1 and laboratory indices for autologous arteriovenous endovascular fistulae failure in maintenance hemodialysis patients[J]. International Journal of Laboratory Medicine,2023,44(12):1468-1472+1478. | Not relevant to the topic |
|  | QIN Yu,DU Jing,ZHAO Zhanyun. Application of small animal models in the study of hemodialysis vascular access[J]. China Blood Purification,2023,22(6):446-449.DOI:10.3969/j.issn.1671-4091.2023.06.010. | Inconsistent with study population |
|  | XU Yanhua,ZHANG Manzhi,HUANG Zhanrong. Predictive role of thromboelastography and coagulation function tests in endovascular fistula blockage in maintenance hemodialysis patients[J]. Tibetan Medicine,2023,44(03):59-60. | Full text not available |
|  | LUO Xu-yang, YANG Wei, KANG Yu-wei, MA Shi-jie, FAN Yi, DENG Fei. Artery-to-fistula diameter ratio of the arteriovenous fistula as a predictor for Artery-to-fistula diameter ratio of the arteriovenous fistula as a predictor for vascular access patency after primary percutaneous transluminal angioplasty (PTA) [J]. Chinese Journal of Blood Purification, 2024, 23(11): 854-858. doi: 10.3969/j.issn.1671-4091.2024.11.011 | Not relevant to the topic |
|  | Du J. Research on the role mechanism of C-kit receptor in neoplastic venous intimal hyperplasia of autologous arteriovenous endovascular fistulae in uremic patients[D]. Shandong University,2023.DOI:10.27272/d.cnki.gshdu.2023.000661. | Not relevant to the topic |
|  | Li Shenyu,Wu Jixin,Huang Lijuan,Deng Qingmei,Su Yongxin. Evaluation of color Doppler ultrasound for the establishment of dialysis vascular access in patients with renal failure and its clinical value[J]. Imaging Research and Medical Applications,2024,8(22):97-99.DOI:10.3969/j.issn.2096-3807.2024.22.032. | Not relevant to the topic |
|  | Zhang YM. Effect of ultrasound-guided intraluminal thrombolysis combined with percutaneous transluminal angioplasty in the treatment of thrombosis of autologous arteriovenous endovascular fistula[D]. Gannan Medical College,2023.DOI:10.27959/d.cnki.ggnyx.2023.000032. | Inconsistent with study population |
|  | Wang Zhaoyu. Analysis of debilitating status and its correlation with loss of function in patients with autologous arteriovenous endovascular fistula[D]. Gannan Medical College,2023.DOI:10.27959/d.cnki.ggnyx.2023.000039. | Not relevant to the topic |
|  | YE Xilan, QIU Bihui, NING Zhifang, XIA Yunfeng, SONG Li, LI Zhilian, LIANG Xinling, FU Xia. Analysis of common clinical complications of different vascular accesses in maintenance hemodialysis patients[J]. Journal of Nursing Management,2012,12(2):129-130 | Not relevant to the topic |
|  | LUO Jing. Influencing factors of hemodialysis artificial vascular endovascular fistula dysfunction and its predictive value of endovascular dysfunction[D]. Guangzhou Medical University,2023.DOI:10.27043/d.cnki.ggzyc.2023.000234. | Inconsistent with study population |
|  | YUAN Weiwei,ZHAO Zhengxing,BIAN Hu,et al. Analysis of risk factors and treatment countermeasures for the occurrence of autologous arteriovenous endovascular fistula failure in hemodialysis patients with chronic renal failure[J]. Modern Medicine and Health Research Electronic Journal,2023,7(06):22-24. | Not relevant to the topic |
|  | Dai En,He Xiaoqin,Yang Min,et al. Effect of ultrasound-guided percutaneous transluminal angioplasty for the treatment of stenosis of autologous arteriovenous fistula[J]. Journal of Interventional Radiology,2023,32(03):258-261. | Not relevant to the topic |
|  | LU Yangui,ZHU Xiaojing,ZHANG Aihong,JIA Lina,LU Wenlu,YI Jinjin,XU Wanting. Relationship between serum VASH-1 and SFRP4 levels and stenosis of autologous arteriovenous fistula in hemodialysis patients[J]. Health Care Medicine Research and Practice,2024,21(7):40-45 | Not relevant to the topic |
|  | HE Chuan'e,RAO Yifeng,SONG Zhixia,et al. Relationship between serum SCUBE-1 and EndoCan levels and thrombosis of arteriovenous fistula in hemodialysis patients[J]. Western medicine,2023,35(02):227-231. | Inconsistent with study population |
|  | Geng Tingting. Preparation of thrombosis risk assessment table for autologous arteriovenous endovascular fistula and its reliability and validity test[D]. Dalian Medical University,2023.DOI:10.26994/d.cnki.gdlyu.2023.000161. | Inconsistent with study population |
|  | Li L,Xue L,Wu MH. Coagulation function plasma hemoglobin and D-D levels in patients with diabetic nephropathy and their relationship with hemodialysis arteriovenous fistula thrombosis[J]. Hebei Medicine,2023,29(01):107-112. | Inconsistent with study population |
|  | SHI Kai, MA Liao, LI Rui, et al. Prediction and application of the risk of arteriovenous endovascular fistula loss in hemodialysis patients[J]. International Journal of Nursing,2024,43(05):826-831.DOI:10.3760/cma.j.cn221370-20210417-00195 | Not relevant to the topic |
|  | Zhuang F,Xian Suli,Wang Yingdeng. Predictive value of preoperative abdominal aortic calcification score for new arteriovenous endovascular fistula loss[J]. China Blood Purification,2022,21(09):676-680. | Not relevant to the topic |
|  | LING Hua-Xiu,QIU Chun-Yan,DENG Chuan-Yan. Predictive value of coagulation-fibrinolysis imbalance for vascular access embolism in maintenance hemodialysis patients[J]. Medical Theory and Practice,2022,35(17):3001-3003.DOI:10.19381/j.issn.1001-7585.2022.17.052. | Not relevant to the topic |
|  | Chen S. Analysis of risk factors for late loss of function in autologous arteriovenous endovascular fistula[C]. Renal Disease Specialized Committee of Chinese Society of Integrative Medicine and Western Medicine. Compendium of abstracts from the 2018 Annual Academic Conference of the Renal Diseases Specialized Committee of the Chinese Society of Integrative Medicine and Western Medicine. Three Gorges Center Hospital;,2018:1. | Conference abstracts,etc |
|  | Ye Qincheng,Wang Sanying,Liu Qianying,et al. Research progress on the prediction model of risk of loss of function of autologous arteriovenous endovascular fistula in maintenance hemodialysis patients[J]. China Nursing Management,2022,22(07):1102-1105. | Not relevant to the topic |
|  | NIGATI ABDUZHEHEMAN,PAZHETANMU TOHUTI. Predictive value of calcium-phosphorus product for endovascular fistula loss after balloon dilatation of arteriovenous endovascular fistula in hemodialysis patients[J]. China Medical Journal,2022,57(07):750-753. | Not relevant to the topic |
|  | Wang Y. Correlation study of preoperative influencing factors and risk assessment of arteriovenous endovascular fistula stenosis[D]. Guilin Medical College,2022.DOI:10.27806/d.cnki.gglyx.2022.000334. | Not relevant to the topic |
|  | Cai, M.. Construction and validation of a prediction model for unplanned extubation risk in patients with indwelling temporary dialysis catheters[D]. Hunan University of Traditional Chinese Medicine,2022.DOI:10.27138/d.cnki.ghuzc.2022.000499. | Inconsistent with study population |
|  | KONG Man-li,LI Ruohe,WANG Su-hong,et al. Risk factors for acute thrombosis of arteriovenous endovascular fistulae in patients on maintenance hemodialysis and the application value of column line drawing[J]. Chinese Journal of Integrative Nephrology,2022,23(05):432-435. | Inconsistent with study population |
|  | HE Xin,ZHAN Ya,ZHANG Hong,et al. Prediction and assessment of the risk of hypotension in 3906 hemodialysis patients on dialysis by the column-line diagram model[J]. China Blood Purification,2022,21(05):350-355. | Inconsistent with study population |
|  | LIN Dongwen. Inhibition of vascular endothelial cell YAP signaling and intervention of arteriovenous fistula stenosis by photodisintegrated drug-carrying granular hydrogels[D]. South China University of Technology,2022.DOI:10.27151/d.cnki.ghnlu.2022.001849. | Not relevant to the topic |
|  | LIU Guojiang,PAN Le,JIANG Zeliang,et al. ROC analysis of ultrasonographic determination of hemodynamic parameters for assessing the value of hemodialysis arteriovenous endovascular fistula function and predicting thrombosis[J]. China Blood Purification,2022,21(04):264-268. | Inconsistent with study population |
|  | PAN Zhongjian. Clinical assessment of the prognostic value of mean erythrocyte width for arteriovenous endovascular fistula surgery[D]. Shandong University,2022.DOI:10.27272/d.cnki.gshdu.2022.005702. | Not relevant to the topic |
|  | SUN Chunyan,SONG Li,TIAN Xiulan,et al. A study on the prediction of the function of autologous arteriovenous endovascular fistulae by the absolute value of the ratio of prepump arterial pressure to pump-controlled blood flow rate[J]. Nursing Research,2022,36(06):952-958. | Not relevant to the topic |
|  | Zhang M. Study on the correlation between postoperative maturation and early puncture of arteriovenous endovascular fistula assessed by ultrasound[D]. Dalian Medical University,2022.DOI:10.26994/d.cnki.gdlyu.2022.000523. | Not relevant to the topic |
|  | ZHOU Qingyi,XU Yuxiang,WANG Kefeng,et al. Expression and mechanism of angiotensin II receptor-like 1 receptor endogenous ligand 13 and angiotensin II in stenosis of autologous arteriovenous endovascular fistula[J]. China Blood Purification,2022,21(02):107-110. | Not relevant to the topic |
|  | ZHANG Yuhan. Construction of thrombosis risk assessment index system of autologous arteriovenous fistula[D]. Dalian Medical University,2022.DOI:10.26994/d.cnki.gdlyu.2022.000836. | Inconsistent with study population |
|  | Zongxiang Zhou,Hong Zhang. Correlation of plasma COMP expression with arteriovenous endovascular fistula loss and prognosis in hemodialysis patients with end-stage renal disease[J]. Journal of Clinical Nephrology,2022,22(01):22-27. | Not relevant to the topic |
|  | YANG Yinan,LI Zhengsheng,XIE Juan,et al. Reflections on the etiology and pathogenesis of autologous arteriovenous endovascular fistula malfunction in Chinese medicine[C]. Guizhou Medical Association, Guizhou Medical Association Nephrology Branch, Guizhou Medical Association Blood Purification Branch. Proceedings of the annual meeting of Guizhou Medical Association Nephrology Branch and Blood Purification Branch. Department of Nephrology, The Second Affiliated Hospital of Guiyang College of Traditional Chinese Medicine;,2018:1. | Conference abstracts,etc |
|  | Lin P,Zhang Y,Chen J,et al. Effect of Chinese medicine fumigation combined with moxibustion on the function of arteriovenous endovascular fistula and analysis of factors influencing the poor function of arteriovenous endovascular fistula[J]. Shanghai Journal of Acupuncture and Moxibustion,2021,40(11):1330-1335.DOI:10.13460/j.issn.1005-0957.2021.11.1330. | Not relevant to the topic |
|  | Wang T, Li YF, Nigati Abdurehman, et al. Relationship between serum soluble late glycosylation end-product receptor, endogenous secretory late glycosylation end-product receptor levels and thrombosis of arteriovenous fistulae in hemodialysis patients[J]. Chinese Journal of Frontiers of Medicine(Electronic Edition),2021,13(10):94-98. | Inconsistent with study population |
|  | ZHANG Zhijian,CHEN Hanzhi,LIU Bin,et al. Predictive role of serum fibroblast growth factor 23 in radial artery calcification and arteriovenous endovascular fistula failure[J]. China Blood Purification,2021,20(06):410-414. | Not relevant to the topic |
|  | Sun Chunyan. Predictive study of pre-pump arterial pressure and pump-controlled blood flow rate ratio on the function of autologous arteriovenous endovascular fistula[D]. Southern Medical University,2021.DOI:10.27003/d.cnki.gojyu.2021.001141. | Not relevant to the topic |
|  | Huang WY. Factors influencing the dysfunction of artificial vascular graft endovascular fistulae in hemodialysis patients and the predictive value of platelet distribution width on the dysfunction of artificial vascular graft endovascular fistulae[D]. Southern Medical University,2021.DOI:10.27003/d.cnki.gojyu.2021.000505. | Not relevant to the topic |
|  | Jiao SW,Zhu GZ,Lu Y,et al. Value of thrombin-antithrombin complex in predicting restenosis after percutaneous transluminal angioplasty in maintenance hemodialysis patients with stenotic arteriovenous fistulae[J]. Chinese Journal of Practical Diagnosis and Therapy,2021,35(05):469-472.DOI:10.13507/j.issn.1674-3474.2021.05.009. | Not relevant to the topic |
|  | Huang, B.F.. Research on simulation model and noninvasive detection technology of arteriovenous fistula[D]. Fujian College of Engineering,2021.DOI:10.27865/d.cnki.gfgxy.2021.000060. | Inconsistent with study population |
|  | Au Yunyao. The role of vascular endothelial cell function in arteriovenous fistula stenosis based on YAP/TAZ signaling pathway[D]. Guangzhou Medical University,2021.DOI:10.27043/d.cnki.ggzyc.2021.000164. | Not relevant to the topic |
|  | Yang J. Analysis of causes and countermeasures of early thrombosis after artificial vascular arteriovenous endovascular fistula[C]. Chinese Society of Integrative Medicine and Western Medicine, Renal Disease Committee. Compilation of Abstracts of the 2018 Annual Academic Conference of the Renal Disease Specialized Committee of the Chinese Society of Integrative Medicine and Western Medicine. Daping Hospital, Army Military Medical University;,2018:1. | Conference abstracts,etc |
|  | Ma PY, Wu YD, Huang FZ, et al. Factors affecting late loss of function of arteriovenous endovascular fistula and its predictive efficacy in hemodialysis patients[J]. Shandong Medicine,2021,61(12):81-84. | Not relevant to the topic |
|  | ZHAN Shen,ZHAO Bin,ZHANG Lihong,WANG Yuzhu. Exploration of risk factors associated with restenosis of endovascular fistula after percutaneous transluminal angioplasty[J]. Journal of Clinical Nephrology,2024,24(3):200-208.DOI:10.3969/j.issn.1671-2390.2024.03.004. | Not relevant to the topic |
|  | Xiao Jian,Ma Liang. Influencing factors of arteriovenous endovascular fistula function in hemodialysis and establishment of prediction model[J]. Chinese Journal of Integrative Nephrology,2021,22(03):242-245. | Full text not available |
|  | CHEN Kai. Factors affecting the outcome of standardized autologous arteriovenous endovascular fistuloplasty and the predictive value of preoperative quantitative rating scale[D]. Chengde Medical College,2021.DOI:10.27691/d.cnki.gcdyx.2021.000249. | Not relevant to the topic |
|  | Jiao, Sweetie. Predictive value of thrombin-antithrombin complex in restenosis after percutaneous transluminal angioplasty of arteriovenous endovascular fistula[D]. Zhengzhou University,2021.DOI:10.27466/d.cnki.gzzdu.2021.002762. | Not relevant to the topic |
|  | Li Jamei. Color Doppler ultrasound assessment of maturation of autologous arteriovenous endovascular fistula and the timing of first puncture[D]. Dalian Medical University,2021.DOI:10.26994/d.cnki.gdlyu.2021.000111. | Not relevant to the topic |
|  | JIA Xiu-Fen,LIU Jin-Jin,ZHENG Duan-Fei.Clinical significance of CT angiography for detecting hemodynamic parameters after autologous arteriovenous endovascular fistuloplasty in hemodialysis patients[J]. China Medicine Herald,2021,18(01):160-163.DOI:10.20047/j.issn1673-7210.2021.01.039. | Not relevant to the topic |
|  | Kong, Xianglei. Expression and mechanism of chitosanase-3-like protein 1 in autologous arteriovenous endovascular fistulae with loss of function in end-stage renal disease[D]. Shandong University,2020.DOI:10.27272/d.cnki.gshdu.2020.006448. | Not relevant to the topic |
|  | Wang Y,Bai Ruoyao. Application value of coagulation-fibrinolytic imbalance evaluation in predicting vascular access embolism in maintenance hemodialysis patients[J]. Shaanxi Medical Journal,2020,49(11):1448-1452. | Not relevant to the topic |
|  | Wang Yi-Ying. Initial patency of endovascular fistula after autologous arteriovenous endovascular fistuloplasty in hemodialysis patients and factors affecting it[J]. Medicine Frontier,2024,14(09):119-121. | Not relevant to the topic |
|  | FANG Wan. Study on the effect of Renkang Wan on endothelial proliferation of rat jugular arteriovenous fistula based on inflammatory factors[D]. Hunan University of Traditional Chinese Medicine,2020.DOI:10.27138/d.cnki.ghuzc.2020.000482. | Inconsistent with study population |
|  | Liu W. Research on the predictive value of static pressure ratio at the venous end on the stenosis of artificial blood vessel arteriovenous endovascular fistula[D]. Nanchang University,2020.DOI:10.27232/d.cnki.gnchu.2020.000493. | Not relevant to the topic |
|  | Tong Jingmaxi. Acoustic detection of stenosis in arteriovenous vascular access[D]. Zhejiang University,2020.DOI:10.27461/d.cnki.gzjdx.2020.002644. | Not relevant to the topic |
|  | WU Fang,LI Yan,XUE Rui. Research progress of predictive modeling of the risk of loss of function of autologous arteriovenous endovascular fistula based on different research protocols[J]. Chinese Journal of Integrative Nephrology,2024,25(06):553-555. | Full text not available |
|  | Chen J. Mechanism of up-regulation of Jagged1 expression in vascular endothelial cells involved in intimal hyperplasia of arteriovenous fistula[D]. Guangzhou Medical University,2020.DOI:10.27043/d.cnki.ggzyc.2020.000453. | Not relevant to the topic |
|  | Deng H. Clinical study of early postoperative ultrasonography to predict the maturation of arteriovenous endovascular fistula[D]. Nanhua University,2020.DOI:10.27234/d.cnki.gnhuu.2020.000658. | Not relevant to the topic |
|  | ZHANG Lirong,HOU Yi,SUN Xiuli,YAN Yonghong,LI Guangyi,FENG Guozheng,YIN Na,CHEN Aizhen,HOU Guocun. Clinical application value of preoperative vascular ultrasonography for wrist autologous arteriovenous endovascular fistula[J]. China Blood Purification,2020,19(1):29-32,36.DOI:10.3969/j.issn.1671-4091.2020.01.008. | Not relevant to the topic |
|  | XU Tao,NING Chunping,ZHOU Maoping,et al. Diagnostic value of brachial artery Doppler ultrasound for hemodialysis autologous arteriovenous endovascular fistula[J]. Journal of Clinical Ultrasound Medicine,2019,21(12):891-894.DOI:10.16245/j.cnki.issn1008-6978.2019.12.004. | Not relevant to the topic |
|  | Wei H,Wang JL,Yang YX. Correlation between serum irisin levels and arteriovenous endovascular fistula thrombosis in elderly maintenance hemodialysis patients[J]. Chinese Journal of Gerontology,2019,39(22):5553-5556. | Inconsistent with study population |
|  | LIU Lu,BIAN Baohua,ZOU Jinhua. Guidance significance of arteriovenous endovascular fistula occlusion risk assessment form for hemodialysis patients[J]. Jilin medicine,2019,40(04):904-906. | Not relevant to the topic |
|  | LIU Xiu,YAN Jianjun,LI Jiangbo,et al. The value of dynamic venous pressure monitoring in predicting arteriovenous endovascular fistula stenosis in hemodialysis patients[J]. Nursing Research,2018,32(18):2986-2988. | Not relevant to the topic |
|  | Wang Zhe. Fluid shear force mediates the expression of monocyte chemotactic protein 1 and its signaling pathway in vascular endothelial cells[D]. Tianjin Medical University,2018. | Not relevant to the topic |
|  | LU Yanyan,LIN Ruya,FAN Qianqian,et al. Study on the application of vascular access pressure monitoring and analysis in predicting arteriovenous endovascular fistula stenosis in hemodialysis patients[J]. Nursing and Rehabilitation,2017,16(02):149-151. | Not relevant to the topic |
|  | Yu HB. Effect of shear force on vascular endothelial cells of arteriovenous endovascular fistula[D]. Tianjin Medical University,2016. | Not relevant to the topic |
|  | Niu Jiyuan. Experimental study on the inhibition of endothelial proliferation of endovascular fistulae by local epicardial slow-release sirolimus[D]. Taishan Medical College,2016.DOI:10.27353/d.cnki.gtsyc.2016.000019. | Not relevant to the topic |
|  | Wang B. Establishment of canine arteriovenous endovascular fistula model and preliminary experimental study[D]. Tianjin Medical University,2014. | Inconsistent with study population |
|  | Lu Z. Establishment and observation of animal model of central venous catheter for hemodialysis[D]. Tianjin Medical University,2014. | Inconsistent with study population |
|  | ZHANG Qian,ZHANG Lihong,WANG Baoxing. Progress of clinical research on maturation of autologous arteriovenous endovascular fistula[J]. China Blood Purification,2011,10(09):507-511. | Not relevant to the topic |
|  | PENG Han Guo. The effect of Tongve Oral Liquid on the function of endovascular fistula after autologous arteriovenous endovascular fistula surgery[D]. Guangzhou University of Traditional Chinese Medicine,2012. | Not relevant to the topic |
|  | WANG Tao, WANG Yunruo, WANG Huixin, et al. Analysis of risk factors for early AVF loss in maintenance hemodialysis patients[J]. International Journal of Urology, 2024, 44(1) : 121-125. DOI: 10.3760/cma.j.cn431460-20220613-00028. | Not relevant to the topic |
|  | Liu Jiali , Hu Shenling , Zhou Peiru , Huang Jiewei , Zhou Ge , Yu Huixia , Hu Bo. Current status and hotspots of hemodialysis arteriovenous fistula research: a visual analysis based on VOSviewer[J]. Chinese Journal of Nephrology, 2023, 39(12): 947-950. DOI: 10.3760/cma.j.cn441217-20230327-00337. | Not relevant to the topic |
|  | Kailai Lau , Hu Shenling , Zhou Peiru , Huang Jiewei , Zhou Ge , Yu Huixia , Hu Bo. Research status and hotspots of arteriovenous fistula in hemodialysis. Research status and hotspots of arteriovenous fistula in hemodialysis: visualization analysis based on VOSviewer[J]. Chinese Journal of Nephrology, 2023, 39(12): 947-950. DOI: 10.3760/cma.j.cn441217-20230327-00337 | Inconsistent with study population |
|  | GAO Liangyun,ZHU Xingyu,WANG Yuelin,et al. Logistic regression analysis of factors ineffective in Doppler ultrasound-guided urokinase treatment of acute arteriovenous endovascular fistula thrombosis[J]. Chinese Journal of Integrative Nephrology,2023,24(9):795-799. DOI:10.3969/j.issn.1009-587X.2023.09.016. | Not relevant to the topic |
|  | MA Wei-hua, DU Shu-tong, BAI Wei-wei, WANG Ya-jing, WANG Na. The correlation between abdominal aortic calcification and arteriovenous fistula patency period following percutaneous transluminal angioplasty[J]. Chinese Journal of Blood Purification, 2023, 22(11): 866-870. |  |
|  | LIU Wenjing,WANG Yufei,ZHANG Beihao,et al. Analysis of epidemiological characteristics, intervention effect and influencing factors of graft arteriovenous fistula thrombosis[J]. Chinese Journal of Nephrology,2024,40(7):526-532. DOI:10.3760/cma.j.cn441217-20231218-01230. | Inconsistent with study population |
|  | ZHANG Cui,LIU Lulu,CHEN Shaoxia. Focused solution model combined with supportive psychological care in patients undergoing balloon dilatation for arteriovenous endovascular fistula stenosis[J]. Qilu Nursing Journal,2024,30(12):145-147. | Not relevant to the topic |
|  | Wang Zi-qiang, Wei Ze-feng, Zheng Jin-hua, Zhu Yong-jun, Cheng Ying, Lyu Xiao-yang. Expression and significance of mitogen-activated protein kinase/ extracellular signal-regulated kinase pathway in vascular tissue at site of intimal hyperplasia after arteriovenous fistula and myofibroblasts in the uremic sera[J]. Journal of Clinical Nephrology, 2024, 24(7): 536-542. DOI: 10.3969/j.issn.1671-2390.2024.07.002 | Not relevant to the topic |
|  | WU Guiqun, LI Yuanyuan, CHEN Bo, HU Ruguangyu. Multiple regressivity analysis of factors influencing the loss of function of autologous arteriovenous endovascular fistulae in maintenance hemodialysis patients[J]. Modern Medicine and Health Research(Electronic Version),2023,7(17):99-102.DOI:10.3969/j.issn.2096-3718.2023.17.032. | Not relevant to the topic |
|  | CHEN Jianping,YANG Yang,FANG Mengjie,CHEN Rong,JIN Zhe,LIAO Aineng. Correlation between serum Endocan-1 and Lp-PLA2 levels and thrombosis of autologous arteriovenous endovascular fistulae in patients on maintenance hemodialysis[J]. Medical Theory and Practice,2024,37(10):1730-1733.DOI:10.19381/j.issn.1001-7585.2024.10.039. | Inconsistent with study population |
|  | HU Haiyang,WU Weidong,YANG Jian. Analysis of factors affecting 1-year loss of function of autologous arteriovenous endovascular fistula in hemodialysis patients[J]. China Medical Innovation,2024,21(29):145-149.DOI:10.3969/j.issn.1674-4985.2024.29.033. | Not relevant to the topic |
|  | XU Yilin,LIU Jun,ZHANG Wenying,WEI Min,LI Hailun. Individualized column chart modeling for predicting the risk of arteriovenous fistula embolism in maintenance hemodialysis[J]. Chinese Electronic Journal of Nephrology Research,2023,12(2):81-86.DOI:10.3877/cma.j.issn.2095-3216.2023.02.004. | Full text not available |
|  | Cuihong Chen. Study on the influencing factors of autologous arteriovenous endovascular fistula failure in maintenance hemodialysis patients[J]. World Digest of Recent Medical Information (Continuous Electronic Journal),2024,24(7):19-23.DOI:10.3969/j.issn.1671-3141.2024.007.004. | Conference abstracts,etc |
|  | YANG Jianxin,FENG Yifan,GUO Qiangqiang. Study on the relationship between loss of function and related clinical indicators after arteriovenous endovascular fistuloplasty in patients on maintenance hemodialysis[J]. Zhejiang Trauma Surgery,2024,29(1):90-93.DOI:10.3969/j.issn.1009-7147.2024.01.027. | Not relevant to the topic |
|  | Lin WP,Wu Y. Analysis of risk factors for AVF loss in hemodialysis patients with end-stage renal disease and construction of risk prediction model[J]. Health Friends,2024,(10):130-132. | Full text not available |
|  | GUO Xiulan,JI Meiyu,LIAO Qing,CHEN Liqing. Survey on the current status of arteriovenous endovascular fistula loss in maintenance hemodialysis patients and analysis of influencing factors[J]. Combined Chinese and Western Medicine Nursing (in Chinese and English),2024,10(3):118-120.DOI:10.11997/nitcwm.202403035. | Not relevant to the topic |
|  | Wang H. Comparison of therapeutic effects of applying different autologous arteriovenous endovascular fistulae in maintenance hemodialysis patients[J]. Chinese Journal of Modern Medicine,2022,24(10):50-53.DOI:10.3969/j.issn.1672-9463.2022.10.012. | Not relevant to the topic |
|  | Liang Y,Sun N,Wang F,Shen Y. Analysis of factors affecting the success rate of autologous arteriovenous endovascular fistulae for maintenance hemodialysis in children with end-stage chronic kidney disease[J]. Chinese Journal of Pediatrics,2015,53(9):660-664.DOI:10.3760/cma.j.issn.0578-1310.2015.09.006. | Inconsistent with study population |
|  | Yang K,Luo Laiyue,Liu Y. Construction of a model of risk factors for autologous arteriovenous endovascular fistulae failure in maintenance hemodialysis patients and strategies for prevention and treatment[J]. Chinese Journal of Continuing Medical Education,2023,46(5):444-448.DOI:10.3760/cma.j.cn115455-20221112-00983. | Full text not available |
|  | Liang Y,Fu QY. Analysis of adverse factors that may affect the function of arteriovenous endovascular fistula in maintenance hemodialysis patients and nursing countermeasures[J]. Guizhou Medicine,2020,44(12):1979-1980. | Not relevant to the topic |
|  | Ruan Qiang,Li Zhaohui,Huang Qiang,Huang Zhiyong,Guo Weichang.Fogarty catheter thrombolysis combined with high-pressure balloon endoluminal plasty in autologous arteriovenous endovascular fistula stenosis with thrombosis in elderly hemodialysis patients[J]. Chinese Journal of Geriatric Multiorgan Diseases,2021,20(12):903-907.DOI:10.11915/j.issn.1671-5403.2021.12.190. | Inconsistent with study population |
|  | Fu DJ,Zhao H. Logistic analysis and intervention of factors affecting initial loss of function in the use of autologous arteriovenous endovascular fistula in dialysis patients[J]. Heilongjiang Medicine,2020,44(1):15-16.DOI:10.3969/j.issn.1004-5775.2020.01.005. | Not relevant to the topic |
|  | Wu JH, Wang F, Zhang YD, Dai HY, Huang XZ, Chen XL. Study on the correlation between malnutrition-inflammation-atherosclerosis syndrome and loss of function of autologous arteriovenous endovascular fistula[J]. Journal of Nantong University (Medical Edition),2017,37(3):262-265. | Not relevant to the topic |
|  | Deng Wei. Clinical application research on blood routine related indexes in autologous arteriovenous endovascular fistula dysfunction in hemodialysis patients[D]. Guangdong Medical University,2023. | Not relevant to the topic |
|  | LI Bin,DANG Xugao,LI Jin,LIU Hengjian. Relationship between plasma growth arrest-specific protein 6 levels and arteriovenous fistula thrombosis in maintenance hemodialysis patients[J]. Journal of Clinical Nephrology,2022,22(9):737-741.DOI:10.3969/j.issn.1671-2390.2022.09.006. | Inconsistent with study population |
|  | WANG Qin,WANG Ying,BU Limei,CHEN Yuyu. Analysis of the causes of occlusion of autologous arteriovenous endovascular fistula in maintenance hemodialysis patients[J]. Baotou medicine,2016,40(3):138-139. | Not relevant to the topic |
|  | [69]Deng Ting. Study on factors related to stenosis of hemodialysis autologous arteriovenous endovascular fistula[J]. Medical Food Therapy and Health,2019,(14):272,278. | Not relevant to the topic |
|  | ZENG Haihong,WU Xiuhong,QIAN Yinling,FENG Biyi,HUANG Jing. Analysis of the causes of arteriovenous endovascular fistula failure in maintenance hemodialysis patients and discussion of countermeasures[J]. China Medical Science,2020,10(16):84-87.DOI:10.3969/j.issn.2095-0616.2020.16.022. | Not relevant to the topic |
|  | Cui, Liye. Analysis of the causes of thrombosis of autologous arteriovenous endovascular fistulae in patients on maintenance hemodialysis[J]. Electronic Journal of Clinical Medicine Literature,2022,9(21):30-32. | Inconsistent with study population |
|  | XIONG Liangwei,ZHANG Xianggui. Risk factors associated with autologous arteriovenous endovascular fistula loss and preventive measures[J]. Medical Review,2017,23(24):4910-4915.DOI:10.3969/j.issn.1006-2084.2017.24.024. | Not relevant to the topic |
|  | Liao L. Study on the mechanism of stenosis of autologous arteriovenous endovascular fistula in maintenance hemodialysis patients based on metabolomics analysis[D]. Department of Medicine, Nanchang University, 2023. | Not relevant to the topic |
|  | WEI Weiguang,TANG Xining,CHEN Jiayi,GUO Rongjin,LEI Qiulian,HUANG Jiantian. Progress of postoperative maturation of autologous arteriovenous endovascular fistula in hemodialysis patients[J]. Chinese Medical Science,2022,12(6):37-40,77.DOI:10.3969/j.issn.2095-0616.2022.06.010. | Not relevant to the topic |
|  | Li J,Chen WD,Zhang Y,Zhang JQ. Analysis of factors affecting calcification of fistula opening of autologous arteriovenous endovascular fistula in patients on maintenance hemodialysis[J]. China Blood Purification,2014,13(11):793-796.DOI:10.3969/j.issn.1671-4091.2014.11.014. | Not relevant to the topic |
|  | Liu Yao, Ding Yanming, Li Jing, Su Li, Liu Tianjiao, Tao Zhenhui, Yu Chongyan, Cao Liyun, Deng Jun, Liu Xia, Wang Zhijian. Quality evaluation and content analysis of guidelines related to dysfunctional monitoring of autologous arteriovenous endovascular fistula[J]. Chinese Journal of Modern Nursing,2019,25(36):4719-4723.DOI:10.3760/cma.j.issn.1674-2907.2019.36.008. | Not relevant to the topic |
|  | Liu S.L. Effect of ultrasound-guided balloon dilatation plasty for arteriovenous endovascular fistula in patients with autologous arteriovenous endovascular stenosis[J]. Chinese and Foreign Medical Research,2023,21(25):63-66.DOI:10.14033/j.cnki.cfmr.2023.25.016 | Not relevant to the topic |
|  | ZHANG Qian,LU Wenwen,TAN Jinyun,WANG Yong,ZHANG Minmin,HUANG Bihong. Coronary artery calcification and assessment of restenosis risk after percutaneous transluminal angioplasty of arteriovenous endovascular fistulae in hemodialysis patients[J]. Journal of Kidney Disease and Dialysis Kidney Transplantation,2023,32(1):8-14.DOI:10.3969/j.issn.1006-298X.2023.01.002. | Not relevant to the topic |
|  | WANG Bo,WEI Fang,SUN Guijiang,CHEN Haiyan,LU Zhi,JIA Lan,JIANG Elie. Study on the duration of autologous arteriovenous endovascular fistula use and its influencing factors in maintenance hemodialysis patients[J]. Chinese Family Medicine,2018,21(29):3577-3581.DOI:10.12114/j.issn.1007-9572.2018.00.097. | Not relevant to the topic |
|  | Ding Jiaxiang,Zhang Dongliang,Dai Wendi,Ji Danying,Zhang Li,Liu Wenhu. Analysis of risk factors for thrombosis of autologous arteriovenous endovascular fistula[J]. Journal of Capital Medical University,2010,31(1):113-116.DOI:10.3969/j.issn.1006-7795.2010.01.026. | Inconsistent with study population |
|  | Xiong LW,Zhang XG. Study on chronic risk factors for failure of mature autologous arteriovenous endovascular fistula[J]. China Blood Purification,2018,17(5):329-334.DOI:10.3969/j.issn.1671-4091.2018.05.010. | Not relevant to the topic |
|  | Deng YX, Wu Y, Huang LB, Liu HH, Huang YP. Current status of color Doppler ultrasound in autologous arteriovenous endovascular fistula[J]. Modern Medicine and Health Research (Electronic Edition),2023,7(8):133-135.DOI:10.3969/j.issn.2096-3718.2023.08.043. | Not relevant to the topic |
|  | SIN Jianzhen,LIN Yuequn,LIAO Binrong,LI Fengjuan,HUANG Tingting. Analysis of factors influencing the formation of arteriovenous fistula embolism in maintenance hemodialysis patients[J]. Nursing Practice and Research,2021,18(17):2557-2560.DOI:10.3969/j.issn.1672-9676.2021.17.007. | Not relevant to the topic |
|  | FU Minghui. Study on clinical complications of different vascular accesses in maintenance hemodialysis patients[J]. Intelligent Health,2024,10(20):47-49.DOI:10.19335/j.cnki.2096-1219.2024.20.015. | Not relevant to the topic |
|  | Tang YQ,Zhang ZL.Significance of TGF-β1 and Smad3 expression in autologous arteriovenous endovascular fistulae of uremic patients[C]. Renal Disease Specialized Committee of Guizhou Society of Integrative Chinese and Western Medicine.Proceedings of the 2018 Guizhou Continuing Education Learning Class of Traditional Chinese Medicine and the Academic Annual Meeting of Renal Disease Specialized Committee of Guizhou Society of Integrative Chinese and Western Medicine. Affiliated Hospital of Guizhou Medical University;,2018:1.DOI:10.26914/c.cnkihy.2018.007409. | Conference abstracts,etc |
|  | Mi Xuhua,Tang Wanxin,Fu Ping,Su Baihai,Zeng Wentong. Analysis of early loss of function of autologous arteriovenous endovascular fistula and its influencing factors[J]. China Blood Purification,2009,8(7):365-368.DOI:10.3969/j.issn.1671-4091.2009.07.006. | Full text not available |
|  | XU Cuiping,YANG Zengdi,CHENG Jinjin,LI Jun,WANG Jun,TONG Qingping. Diagnostic value of brachial artery flow parameters assessed by color Doppler ultrasound for stenosis of cephalic venous anastomosis complicated by autologous arteriovenous endovascular fistula[J]. China Blood Purification,2022,21(10):766-769.DOI:10.3969/j.issn.1671-4091.2022.10.014. | Not relevant to the topic |
|  | Su Shanna,He Da. Effect of risk-graded care on arteriovenous endovascular fistula function in maintenance hemodialysis patients[J]. Nursing Research,2019,33(19):3445-3447.DOI:10.12102/j.issn.1009-6493.2019.19.034. | Not relevant to the topic |
|  | Ou Yuexiu,Qin Shuguang,Lin Changping,Wang Turnzhen. Logistic regression analysis of risk factors for autologous arteriovenous endovascular fistula malfunction in hemodialysis patients[J]. Anhui Medicine,2019,23(1):72-75.DOI:10.3969/j.issn.1009-6469.2019.01.019. | Not relevant to the topic |
|  | Gui Qiaoqiao,He Yun,Wu Ying,Cao Dan,Shen Zhaonan. Analysis of the correlation between the dose of recombinant human erythropoietin used and late loss of function of autologous arteriovenous endovascular fistula[J]. Clinical Blood Transfusion and Laboratory,2021,23(6):770-773.DOI:10.3969/j.issn.1671-2587.2021.06.021. | Not relevant to the topic |
|  | FAN YP,ZHU YH,ZHAO PING,CAO XJ,CHEN T,SHEN LIANGLAN,HUANG SHU. Changes of high mobility group protein B1 in thrombosis in hemodialysis autologous arteriovenous endovascular fistula patients with chronic renal failure[J]. Chinese Electronic Journal of Clinical Laboratory Management,2016,4(3):179-182.DOI:10.3877/cma.j.issn.2095-5820.2016.03.011. | Inconsistent with study population |
|  | YANJJ,ZHANGZHONGHUA,YANHE,LICHUN,WANGHUI. Effect of continuous monitoring of autologous arteriovenous endovascular fistula flow and recirculation rate on hemodialysis patients[J]. Nursing Research,2015,(33):4127-4130.DOI:10.3969/j.issn.1009-6493.2015.33.010. | Not relevant to the topic |
|  | Hur Yunxia,Hur Yuexia. Analysis of the causes of arteriovenous endovascular fistula failure and nursing care in maintenance hemodialysis patients[C]. Nanjing Society of Rehabilitation Medicine. Proceedings of the First National Academic Exchange Conference on Rehabilitation and Clinical Pharmacy (III). Ningxia Hospital of Traditional Chinese Medicine & Institute of Traditional Chinese Medicine;,2022:6.DOI:10.26914/c.cnkihy.2022.085347. | Conference abstracts,etc |
|  | Liu H, Zhang W, Zhu Q, Wang XY, Liu ZM, Kan XL. Correlation analysis between blood flow of autologous arteriovenous endovascular fistula and occurrence of endovascular thrombosis[J]. Hebei Medicine,2011,33(19):2928-2929.DOI:10.3969/j.issn.1002-7386.2011.19.022. | Inconsistent with study population |
|  | Zhou QY,Xu YX,Wang KF. Correlation between restenosis of autologous arteriovenous fistula and calcium and phosphorus metabolism disorders after percutaneous transluminal angioplasty[J]. Chinese Drugs and Clinics,2021,21(11):1841-1844.DOI:10.11655/zgywylc2021.11.001. | Not relevant to the topic |
|  | ZHU Wei,JIANG Jun,PENG Li,WANG Peng,ZHU Chengxiu,LAN Lei. Survival analysis of graft arteriovenous endovascular fistula in maintenance hemodialysis patients[J]. China Blood Purification,2023,22(12):939-943.DOI:10.3969/j.issn.1671-4091.2023.12.012. | Not relevant to the topic |
|  | HE Ping,XU Rui,HUA Qiuju,JIANG Lu,WANG Weiping. Predictive analysis of erythrocyte distribution width and platelet ratio for arteriovenous endovascular fistulae failure in maintenance hemodialysis patients with end-stage renal disease[J]. Chinese Journal of Continuing Medical Education,2024,47(11):988-994.DOI:10.3760/cma.j.cn115455-20240322-00264. | Not relevant to the topic |
|  | HUANG Yanyan,YANG Xiaoyan,PAN Xianxu. Impact of cascade care based on risk assessment on patients with arteriovenous endovascular fistula on maintenance hemodialysis[J]. Weekly Digest - Elderly Weekly,2024,(16):239-241. | Not relevant to the topic |
|  | Li G. Meta-analysis of factors influencing the loss of function of autologous arteriovenous endovascular fistulae in maintenance hemodialysis patients[D]. Huzhou Normal College,2020. | Not relevant to the topic |
|  | TIAN Guanyuan,ZHENG Ling,ZHONG Xiaoze,WU Yiqiang,WU Wuzhong. Factors affecting early loss of function and changes in blood pressure rhythm and blood pressure variability of arteriovenous endovascular fistula in hemodialysis patients[J]. Modern Medicine and Health Research (Electronic Edition),2022,6(8):36-40. | Not relevant to the topic |
|  | ZHANG Yanzi,SUI Xiaolu,XU Yunpeng,ZHANG Aisha,XIE Tingfei,CHEN Jihong. Correlation between endovascular pathologic changes and vascular shear stress during arteriovenous fistula loss in rats[J]. China Blood Purification,2022,21(5):356-360.DOI:10.3969/j.issn.1671-4091.2022.05.012. | Inconsistent with study population |
|  | LIU Yao, CHENG Xuyang, ZHENG Xizi, DUAN Xiufang, LIU Xiaohui, HAN Dongsheng, TAO Zhenhui, CHEN Yuqing, WANG Zhijian. Research progress on methods for monitoring dysfunction of autologous arteriovenous endovascular fistula[J]. China Blood Purification,2019,18(10):722-724.DOI:10.3969/j.issn.1671-4091.2019.10.017. | Not relevant to the topic |
|  | Xue Na,Zhou Mengni,Shi Juan. Impact of intensive care based on WeChat platform on quality of life and arteriovenous endovascular fistula function of hemodialysis patients[J]. Clinical Medicine Research and Practice,2024,9(20):128-131.DOI:10.19347/j.cnki.2096-1413.202420032. | Not relevant to the topic |
|  | Zhang Zhaoxing. Causes of thrombosis of arteriovenous endovascular fistulae in renal failure on maintenance hemodialysis[J]. China Health Standard Management,2020,11(7):68-71.DOI:10.3969/j.issn.1674-9316.2020.07.025. | Inconsistent with study population |
|  | WEI Wei, CHU Zhi-Hua, SHU Xing, ZHANG Li-Fang, SU Wen-Xiang, HAN Si-Sheng, XU Yan-Qiu, WANG Yi, YAO Tian-Wen. Risk factors for thrombosis of autologous arteriovenous fistula and current status of Chinese and Western medicine treatment[J]. Chinese Journal of Integrative Nephrology,2023,24(1):61-63.DOI:10.3969/j.issn.1009-587X.2023.01.017. | Inconsistent with study population |
|  | WU Lu-Wei, YE Yong-Ling, JI Qing, HUANG Gong-Cai, ZHANG De-Zhong. The value of uric acid, D-dimer and homocysteine in predicting restenosis of arteriovenous endovascular fistula after PTA in hemodialysis patients[J]. Zhejiang Clinical Medicine,2021,23(12):1814-1815. | Not relevant to the topic |
|  | Shan Fanhua. Causes and clinical characteristics of arteriovenous endovascular fistula thrombosis in patients with renal failure on maintenance hemodialysis[J]. Frontiers of medicine,2019,9(13):120. | Inconsistent with study population |
|  | HUANG Jianyu,DUAN Liangliang. Predictive modeling of arteriovenous endovascular fistula malfunction in hemodialysis patients based on the algorithm of synthetic oversampling technique for a few types of samples[J]. Journal of Clinical Nephrology,2024,24(05):378-384. | Full text not available |
|  | Cao HH. Analysis of the causes of thrombosis of autologous arteriovenous endovascular fistulae in patients on maintenance hemodialysis[C]. Proceedings of the 17th East China Nephrology Forum and Shandong Province Nephrology Annual Conference.2017:669-669. | Conference abstracts,etc |
|  | Wen Mowan. Multicenter survival analysis of autologous arteriovenous endovascular fistula in patients with chronic nephritis on maintenance hemodialysis[C].Proceedings of the 2018 Annual Meeting of the Chinese Hospital Association of Hemodialysis Center Management Branch and the 10th China Hemodialysis Forum, the 3rd Asia-Pacific Dialysis Access Conference & 2017 Henan Provincial Medical Association Hemodialysis Academic Annual Conference.2018:1-1. | Conference abstracts,etc |
|  | Li N,Peng KF. Analysis of risk factors for arteriovenous endovascular fistula embolism in maintenance hemodialysis patients[J]. Journal of Bengbu Medical College,2017,42(11):1510-1512,1516.DOI:10.13898/j.cnki.issn.1000-2200.2017.11.023. | Not relevant to the topic |
|  | Liu Xinwu,Liao Shuangqing,Wang Runxiu. Progress of research on the application of Doppler ultrasound in the establishment and maintenance of autologous arteriovenous endovascular fistula[J]. Journal of Gannan Medical College,2021,41(3):288-292,306.DOI:10.3969/j.issn.1001-5779.2021.03.014. | Not relevant to the topic |
|  | Luo Xianglan, Song Li, Fu Xia, Quan Zilin, Zhao Liyan, Cui Dongmei, Peng Yinyan, Chen Cheng, Feng Zhonglin, Tao Yiming, Ye Zhiming, Liang Xinling. Evaluation of 2-year follow-up after percutaneous endovascular treatment for arteriovenous fistula malfunction[J]. Nursing Research,2021,35(23):4144-4150.DOI:10.12102/j.issn.1009-6493.2021.23.002. | Not relevant to the topic |
|  | Zhao Yina. Study on the effect of physical activity on arteriovenous endovascular fistula thrombosis in maintenance hemodialysis patients[D]. Shantou University,2023. | Inconsistent with study population |
|  | SHAO Yanqiu,KONG Min,WANG Qian,WU Xiaorong. Application research on pretreatment system of risk factors for arteriovenous endovascular fistula loss in hemodialysis patients[J]. Chinese Journal of Modern Nursing,2016,22(35):5083-5086,5087.DOI:10.3760/cma.j.issn.1674-2907.2016.35.014. | Not relevant to the topic |
|  | Luo G,Li D. Causes of obstruction and early management of autologous arteriovenous endovascular fistula in hemodialysis patients[J]. China Medical Guide,2010,08(25):99-100.DOI:10.3969/j.issn.1671-8194.2010.25.067. | Not relevant to the topic |
|  | YAN Ai-Hong,BAO Xiu-Qin,ZHANG Qiao-Gen. Risk factors for loss of function of arteriovenous endovascular fistula and its relationship with aortic arch calcification[J]. China Blood Purification,2023,22(2):86-89.DOI:10.3969/j.issn.1671-4091.2023.02.002. | Not relevant to the topic |
|  | FU Ping,CUI Tianlei. Management of thrombus in autologous arteriovenous endovascular fistula[J]. Journal of Nephrology and Dialysis Kidney Transplantation,2013,22(2):139-140.DOI:10.3969/j.issn.1006-298X.2013.02.008. | Inconsistent with study population |
|  | Liu Yamin. Analysis of risk factors for dialysis catheter-related bloodstream infections and risk prediction model[D]. Zhengzhou University,2021. | Inconsistent with study population |
|  | LIU Haiyan,ZHAO Naxin,XIANG Pan,REN Wenwen,ZENG Zhili,DONG Qinghua. Effect of nutritional control status on late loss of function of arteriovenous endovascular fistula in patients with end-stage renal disease[J]. China Blood Purification,2023,22(7):542-545,556.DOI:10.3969/j.issn.1671-4091.2023.07.015. | Not relevant to the topic |
|  | Chen J. Discussion on clinical monitoring and experimental strategy of vascular access failure in maintenance hemodialysis[J]. China Health Nutrition,2018,28(30):121.DOI:10.3969/j.issn.1004-7484.2018.30.104. | Not relevant to the topic |
|  | Gao Yanjing,Yuan Zhongmin,Wang Jun,Liu Mingkang. The assessment value of hemodynamic parameters on the function of arteriovenous endovascular fistula in hemodialysis patients[J]. Journal of Vascular and Endoluminal Vascular Surgery,2023,9(7):876-880.DOI:10.19418/j.cnki.issn2096-0646.2023.07.23. | Not relevant to the topic |
|  | Hu T,Zhou JF. Progress of technical research on the enabling phase of hemodialysis autologous arteriovenous endovascular fistula[J]. China Blood Purification,2021,20(6):395-397.DOI:10.3969/j.issn.1671-4091.2021.06.009. | Not relevant to the topic |
|  | Zhou Ye. Analysis of adverse factors that may affect the function of arteriovenous endovascular fistula in maintenance hemodialysis patients and nursing countermeasures[J]. Family with pregnant babies,2021,3(11):286. | Not relevant to the topic |
|  | YANG Yuanyuan. Analysis of the distribution and influence factors of traditional Chinese medicine of stenosis of autologous arteriovenous fistula[D]. Hubei University of Traditional Chinese Medicine,2023. | Not relevant to the topic |
|  | SHAO Danni, XIONG Fei, ZHU Qing. Analysis of factors influencing the failure of arteriovenous endovascular fistulae in hemodialysis patients[J]. Journal of Emergency and Critical Care in Internal Medicine,2013,19(1):30-32.DOI:10.11768/nkjwzzzz20130112. | Not relevant to the topic |
|  | Feng, Yuanhao. Analysis of the efficacy of percutaneous transluminal angioplasty for the treatment of stenosis or occlusion of autologous arteriovenous endovascular fistulae and postoperative endovascular fistulae loss[D]. Guangzhou Medical University,2021. | Not relevant to the topic |
|  | TIAN Xin, MU Jing, HE Yanbo, ZHANG Lixin. Relationship between hyperhomocysteinemia and arteriovenous endovascular fistula loss[J]. Chinese Community Physician,2014,(21):105-106,108.DOI:10.3969/j.issn.1007-614x.2014.21.66. | Not relevant to the topic |
|  | Zhu YL, Ding H, Fan PL, Gu QL, Teng J, Zhuang Z, Wang WP. Observation and evaluation of color Doppler ultrasound on preoperative vascularization of autologous arteriovenous endovascular fistula molding[J]. China Blood Purification,2015,14(4):246-249.DOI:10.3969/j.issn.1671-4091.2015.04.017. | Not relevant to the topic |
|  | Huang YJ,Wu JQ,Huang L,Huang JL,Chen YP. Relationship between the degree of vascular calcification (VC) and arteriovenous fistula (AVF) loss of function in hemodialysis (HD) patients[J]. China Modern Physician,2019,57(27):25-28. | Not relevant to the topic |
|  | Chen Hongmei. Application effect of all-point docking nursing in the care of arteriovenous endovascular fistula in hemodialysis patients[J]. Dialysis and Artificial Organs,2023,34(2):50-54.DOI:10.3969/j.issn.1005-0809.2023.02.013. | Not relevant to the topic |
|  | Yang Qiong,Feng Jin. Effect of anticipatory care on the complication rate of arteriovenous endovascular fistula in maintenance hemodialysis patients[J]. Chinese Drugs and Clinics,2020,20(19):3306-3308.DOI:10.11655/zgywylc2020.19.065. | Not relevant to the topic |
|  | Mo Wenqi,Xu Juling,Jiang Hua,Zhu Wenya,Yang Dandan,Yuan Jing. Progress of non-pharmacologic intervention for maturation of autologous arteriovenous endovascular fistula[J]. China Blood Purification,2021,20(6):401-404.DOI:10.3969/j.issn.1671-4091.2021.06.011. | Not relevant to the topic |
|  | Liu L. Study on the relationship between vascular calcification and peripheral blood FGF-23 and Fetuin-A levels in undialyzed patients with chronic kidney disease stage 5[D]. Inner Mongolia Medical University,2016. | Not relevant to the topic |
|  | Wang, Xiaoyun. Complications of chronic vascular access[C]. Symposium on the Sixth National Academic Conference of the Chinese Medical Association Nephrology Branch. Beijing:Chinese Medical Association,2002:172-175. | Conference abstracts,etc |
|  | Ye Ruizhong. Observation and evaluation of preoperative vascularization of forearm endovascular fistula by vascular ultrasound[C].2011 Zhejiang Provincial Ultrasound Medicine Academic Annual Conference Compendium of Papers.2011:. | Conference abstracts,etc |
|  | XIAN Xinyang,XIAO Qingxuan,XIE Yingye,CAO Ting. Analysis of the preventive effect of an arteriovenous endovascular fistula care program on autologous arteriovenous endovascular fistula failure based on evidence-based medicine[J]. Electronic Journal of Practical Clinical Nursing,2023,8(22):109-111. | Not relevant to the topic |
|  | AN Huiwoo,QI Xingmin,WANG Rui,JIAO Xia,WANG Shijie.Analysis of the effects and prognostic factors of Gracz arteriovenous endovascular fistula and deep venous catheter indwelling on hemodialysis patients[J]. China Blood Purification,2020,19(7):471-475.DOI:10.3969/j.issn.1671-4091.2020.07.010. | Not relevant to the topic |
|  | Wang Gelao Hao. Analysis of risk factors for late functional failure of arteriovenous endovascular fistula in maintenance hemodialysis patients[C]. Proceedings of the 2019 Academic Annual Meeting (CNA2019) of the Chinese Physicians Association Nephrologists Branch.2019:1-1. | Conference abstracts,etc |
|  | Xiong LW. Study on chronic risk factors for failure of mature autologous arteriovenous endovascular fistula[D]. Zunyi Medical College,2018. | Not relevant to the topic |
|  | ZOU Zhaohua,QING Wei,TANG Liqun,ZHANG Zhen,ZHU Maocai. Construction of a simple model for grading arteriovenous endovascular fistula in maintenance hemodialysis patients[J]. Chinese Journal of Practical Nursing,2023,39(5):374-378.DOI:10.3760/cma.j.cn211501-20220129-00289. | Full text not available |
|  | Fang Ye. Clinical application of ultrasound-guided PTA in the treatment of arteriovenous endovascular fistula stenosis[C]. Zhejiang Provincial Medical Association,Zhejiang Provincial Medical Association Ultrasound Medicine Branch.2019 Compilation of Papers from the Academic Conference on Ultrasound Medicine of Zhejiang Provincial Medical Association. The Second Hospital of Yinzhou District, Ningbo City;,2019:1.DOI:10.26914/c.cnkihy.2019.099329. | Conference abstracts,etc |
|  | ZHENG Duanfei,ZHANG Zhoucang,XU Liyang,CHEN Bicheng,LIU Yi,HUANG Chaoxing,ZHENG Jingchen.Establishment of an autologous arteriovenous endovascular fistula model in 5/6 nephrectomized rats[J]. Chinese Journal of Nephrology,2012,28(4):331-332.DOI:10.3760/cma.j.issn.1001-7097.2012.04.018. | Inconsistent with study population |
|  | Chen W.H., Wang Y.K., Liao F.Q., Wang Y.G.. Independent risk factors for AVF longevity in MHD uremic patients based on COX regression analysis[J]. Guangzhou Medicine,2023,54(7):93-98.DOI:10.3969/j.issn.1000-8535.2023.07.017. | Not relevant to the topic |
|  | KONG Man-li,LI Ruohe,WANG Su-hong,LU Shi-kai,SHI Guo-chan. Analysis of factors associated with the occurrence of arteriovenous endovascular fistula thrombosis and evidence-based medical care[J]. Modern Practical Medicine,2021,33(6):806-807,814.DOI:10.3969/j.issn.1671-0800.2021.06.050. | Inconsistent with study population |
|  | WANG X, WANG N, WU GZ, LIU W, SHI J, HUANG SZ, CAI JJ, HAN JH. Effect of atorvastatin on arteriovenous endovascular fistula function in end-stage renal disease patients with diabetes mellitus[J]. Anhui Medicine,2023,27(9):1877-1881,cover 3.DOI:10.3969/j.issn.1009-6469.2023.09.041. | Not relevant to the topic |
|  | Xue C,Tan Jazhen,Kong Yuanyuan,Xiang Dongmei. Analysis of factors affecting the time to primary patency and short-term patency rate after arteriovenous endovascular fistula surgery for upper extremity grafts[J]. Journal of Clinical Nephrology,2023,23(8):621-627.DOI:10.3969/j.issn.1671-2390.2023.08.002. | Not relevant to the topic |
|  | HU Menghong,ZHAO Guangben,YU Changqing,CHEN Jilin,LIN Hongli. Research progress of vascular calcification in arteriovenous endovascular fistula[J]. China Blood Purification,2022,21(11):823-826.DOI:10.3969/j.issn.1671-4091.2022.11.008. | Not relevant to the topic |
|  | Cui XY. Study on the effects of different vascular access choices on cardiac function and prognostic influencing factors in maintenance hemodialysis patients[J]. Electronic Journal of Clinical Medicine Literature,2017,4(13):2443-2444.DOI:10.3877/j.issn.2095-8242.2017.13.043. | Not relevant to the topic |
|  | Hu, Yi-Min. Application value of ultrasonography combined with Murray's law in assessing autologous arteriovenous endovascular fistulae loss of function[D]. Guangdong Medical University,2023. | Not relevant to the topic |
|  | Kang Qiyu. Evaluation of the efficacy of stepped balloon dilatation in the treatment of stenosis of autologous arteriovenous endovascular fistula[D]. Shanxi Medical University,2023. | Not relevant to the topic |
|  | KONG Xianglei. Expression and mechanism of chitosanase-3-like protein 1 in autologous arteriovenous fistulae in end-stage renal disease[D]. Shandong University,2020. | Not relevant to the topic |
|  | CHI YANQING,LI YAN,GUO ZANGHU,WANG BAOXING.The effect of PTA balloon dilatation pressure on the patency rate of arteriovenous endovascular fistula stenosis after PTA and its risk factor analysis[J]. PLA Medical Journal,2021,46(11):1085-1091.DOI:10.11855/j.issn.0577-7402.2021.11.04. | Not relevant to the topic |
|  | Zhang, Li-Rong. Study on the correlation between preoperative cephalic vein lesions and initial patency rate of arteriovenous endovascular fistula[D]. Inner Mongolia Medical University,2020. | Not relevant to the topic |
|  | SHA Yabin,REN Wanjun,KANG Na,XU Lin. Effect of vascular access type on inflammation in new hemodialysis patients[J]. Chinese Journal of Endemic Disease Control,2013,28(4):308-311. | Not relevant to the topic |
|  | WEI Toujiao. Clinical study on the safety and efficacy of PIRRT using arteriovenous endovascular fistula in maintenance hemodialysis patients[D]. Xi'an Medical College,2023. | Not relevant to the topic |
|  | WANG LH, WEI F, CHEN Haiyan, SUN Guijiang, BI Xueqing, JIANG Elli. Study on the effects of different vascular access choices on cardiac function and prognostic influencing factors in maintenance hemodialysis patients[J]. Chinese Family Medicine,2016,19(30):3681-3684,3690.DOI:10.3969/j.issn.1007-9572.2016.30.008. | Not relevant to the topic |
|  | Cao W.H.. Evaluation of the application of comprehensive nursing care in the preventive care of arteriovenous endovascular fistula failure[J]. Health must read,2020,(27):92. | Not relevant to the topic |
|  | XU Yao. Correlation between klotho gene polymorphism and autologous arteriovenous fistula dysfunction in dialysis patients[D]. Yangzhou University,2020. | Not relevant to the topic |
|  | Du J. Study on the mechanism of C--kit receptor in the neoplastic venous endothelial proliferation of autologous arteriovenous endocardial fistula in uremic patients[D]. Shandong University,2023. | Not relevant to the topic |
|  | Weng Li-Na,Cui Yi-Hong,Xu Wei. Analysis of nutritional status of maintenance hemodialysis patients and its influencing factors[J]. Hainan Medicine,2018,29(6):773-777.DOI:10.3969/j.issn.1003-6350.2018.06.011. | Not relevant to the topic |
|  | Xu Shuqi,Tang Lijun,Cao Wei,Liang Liming,Wang Zunsong,Kong Xianglei. A retrospective cohort study of factors influencing the primary patency rate of radial artery-cephalic vein endovascular fistula in patients with end-stage renal disease[J]. Journal of Nephrology and Dialysis Kidney Transplantation,2023,32(1):15-20.DOI:10.3969/j.issn.1006-298X.2023.01.003. | Not relevant to the topic |
|  | GUO Yue,NING Yunfeng,WANG Xiaoyi,HAN Rongrong. Current status and progress of research on self-care of arteriovenous endovascular fistula in hemodialysis patients[J]. Combined Chinese and Western Medicine Nursing (in Chinese and English),2020,6(7):245-247.DOI:10.11997/nitcwm.202007054. | Not relevant to the topic |
|  | Gan Z, Chen T, Feng YM, Zhou L, Guan J. Percutaneous transluminal angioplasty for the treatment of artificial vascularized arteriovenous endovascular fistulae failure in patients on maintenance hemodialysis[J]. Jiangsu Medicine,2020,46(2):162-166.DOI:10.19460/j.cnki.0253-3685.2020.02.016. | Not relevant to the topic |
|  | Dai Lu, Tian Maolu, Cha Yan, Liu Lu, Li Zhengsheng, Huang Chengbiao, Yuan Jing. Relationship between lean tissue index and loss of function of arteriovenous endovascular fistula in maintenance hemodialysis patients[J]. Chinese Journal of Nephrology,2023,39(1):32-35.DOI:10.3760/cma.j.cn441217-20220621-00633. | Not relevant to the topic |
|  | Ma Lijie,Zhao Sumei,Sun Fang,Sun Qianmei. Potential predictive value of mean platelet volume and mean platelet volume/platelet count ratio for vascular access failure in hemodialysis patients[J]. Chinese Electronic Journal of Nephrology Research,2024,13(2):61-67.DOI:10.3877/cma.j.issn.2095-3216.2024.02.001. | Not relevant to the topic |
|  | Lin X. Effects of statins and antiplatelet drugs on patency of autologous arteriovenous endovascular fistula[D]. Chongqing Medical University,2021. | Not relevant to the topic |
|  | Chen Yinggui. Effect of blood pressure variability in dialysis on the function of arteriovenous endovascular fistula in maintenance hemodialysis patients[D]. Southern Medical University,2022. | Not relevant to the topic |
|  | MA Wei-Hua,DU Shu-Tong,BAI Wei-Wei,WANG Ya-Jing,WANG Na. Correlation analysis of abdominal aortic calcification and time of arteriovenous endovascular fistula patency after percutaneous angioplasty[J]. China Blood Purification,2023,22(11):866-870.DOI:10.3969/j.issn.1671-4091.2023.11.015. | Not relevant to the topic |
|  | Huang, F.C.. Effect of zero-pressure modified puncture method on the incidence of puncture point blood leakage in patients with new arteriovenous endovascular fistula maintenance hemodialysis in forearm[J]. Qinghai Medical Journal,2022,52(1):19-21. | Not relevant to the topic |
|  | Xie Chunhe.Role of p38MAPK and PCNA in the pathogenesis of autologous arteriovenous endovascular fistula stenosis[D]. Hebei Medical University,2022. | Not relevant to the topic |
|  | LIU Shuting,FENG Ji,YANG Yaqin. Analysis of the predictive value of peripheral blood tissue protease level on arteriovenous fistula stenosis and the treatment effect of urokinase combined with argatroban in patients undergoing maintenance hemodialysis[J]. Chinese Journal of Continuing Medical Education,2023,46(3):225-229.DOI:10.3760/cma.j.cn115455-20220727-00667. | Not relevant to the topic |
|  | YUAN Yuan,WANG Wenjing,ZHAI Panpan. Effect of risk-graded care on the function and complications of arteriovenous endovascular fistulae in maintenance hemodialysis patients[J]. Health Home,2021,(16):31-32. | Not relevant to the topic |
|  | QIAN Yongmei. Risk factors and nursing care for thrombosis of arteriovenous endovascular fistula[J]. Integrative Nursing in Chinese and Western Medicine (in Chinese and English),2017,3(9):191-194.DOI:10.11997/nitcwm.201709060. | Inconsistent with study population |
|  | PENG Fei,HUANG Dejian,ZHANG Xiaosheng.Application of 64-row spiral CT angiography in hemodialysis forearm arteriovenous fistula[J]. Jiangsu Medicine,2011,37(20):2448-2449. | Not relevant to the topic |
|  | Application of 64-row spiral CT angiography in arteriovenous fistula[C]. Proceedings of the Frontiers of Nephrology Forum of the Chinese Academy of Engineering, Department of Medicine and Health, and the 13th North China and Beijing Nephrology Annual Conference.2012:1-2. | Conference abstracts,etc |
|  | ZHONG Xiaoyan,YANG Feng,ZHONG Xiaorong,WANG Mingzhe.Study on the relationship between CD62P,CD63,mean platelet volume and thrombosis of arteriovenous endovascular fistula[J]. Chinese and Foreign Medicine,2021,40(29):31-34.DOI:10.16662/j.cnki.1674-0742.2021.29.031. | Inconsistent with study population |
|  | ZHENG Duanfei, SUN Wenwen, JIA Xiufen, MU Kekai, PAN Huijun, LIU Yi, HUANG Chaoxing. Application of percutaneous transluminal angioplasty in arteriovenous endovascular stenosis in hemodialysis patients[J]. Journal of Wenzhou Medical University,2015,(6):426-429.DOI:10.3969/j.issn.2095-9400.2015.06.008. | Not relevant to the topic |
|  | Lee, Yi-Shrimp. A case of arteriovenous endovascular fistula loss due to high radial artery variant[J]. Fujian Medical Journal,2021,43(2):176-177.DOI:10.3969/j.issn.1002-2600.2021.02.069. | Not relevant to the topic |
|  | Dai, T.-S.. Effect of blunt needle grommet puncture technique in hemodialysis patients with autologous arteriovenous endovascular fistula[J]. Health Care Guide,2022,(1):59-62. | Not relevant to the topic |
|  | WANG Mengfan. Fibroblast growth factor 23 in the prediction of poor maturation of autologous arteriovenous fistula[D]. Zhengzhou University,2022. | Not relevant to the topic |
|  | LI Zhui,ZHANG Mao,ZHAO Yu,REN Wei,CHENG Jun. Observation on the effect of hyperbaric oxygen on the blood flow of arteriovenous endovascular fistula for dialysis[J]. Sichuan Animal,2013,32(1):112-115.DOI:10.3969/j.issn.1000-7083.2013.01.022. | Inconsistent with study population |
|  | DUAN Shuzong,DONG Qiaorong,WANG Jingfu,ZHANG Baohong. Hemoglobin and erythrocyte distribution width for predicting late thrombosis in arteriovenous endovascular fistula[J]. Journal of Clinical and Experimental Medicine,2015,(17):1438-1439.DOI:10.3969/j.issn.1671-4695.2015.017.014. | Inconsistent with study population |
|  | SUN Xiuli,ZHANG Lirong,ZHOU Dake,ZHAO Jishan,HOU Yi,YAN Yonghong,LEI Zhenkun,HOU Guocun. Correlation between pathological changes of cephalic vein and its maturation before arteriovenous endovascular fistula[J]. Journal of Clinical Nephrology,2021,21(4):281-287.DOI:10.3969/j.issn.1671-2390.w20-190. | Not relevant to the topic |
|  | Li Meixiu,Li Xiaoqin,Wu Jing. Clinical significance of arteriovenous pressure monitoring in predicting endovascular fistula stenosis in hemodialysis patients[J]. General Practice Nursing,2017,15(28):3483-3485.DOI:10.3969/j.issn.1674-4748.2017.28.011. | Not relevant to the topic |
|  | Hua Sen. Clinical monitoring and experimental study of vascular access failure in maintenance hemodialysis[D]. Second Military Medical University,2008. | Not relevant to the topic |
|  | LIU Fenghua,WANG Zhaoxing,XU Yan,LIU Changchun. Significance of erythrocyte distribution width in the functional abnormalities of different vascular pathways in hemodialysis patients[J]. Journal of Clinical and Experimental Medicine,2022,21(7):722-726.DOI:10.3969/j.issn.1671-4695.2022.07.014. | Not relevant to the topic |
|  | XU Yan,HUANG Wen,ZHAI Yanling. Relationship between arteriovenous fistula thrombosis and soluble endothelial cell protein C receptor[J]. Chinese Journal of Nephrology,2010,26(9):714-715.DOI:10.3760/cma.j.issn.1001-7097.2010.09.015. | Inconsistent with study population |
|  | CHEN Zhenwei, ZENG Haiou, HUANG Fengqin, FU Qianli, LU Minhong, WU Qiang, YANG Ticheng. Effect of radial artery calcification at the anastomosis of arteriovenous endovascular fistula on endovascular fistula and survival of patients with end-stage renal disease[J]. Chinese Journal of Nephrology,2018,34(11):822-830.DOI:10.3760/cma.j.issn.1001-7097.2018.11.004. | Not relevant to the topic |
|  | Ye Yuan,Xu ZP,Jiang XY,Chen XM,Wan LF,Feng T. Effect of risk-graded care model on endovascular fistula function in maintenance hemodialysis patients[J]. Chinese Journal of Integrative Nephrology,2014,15(3):255-257. | Not relevant to the topic |
|  | Xiong Lin. Effects of mineral metabolism disorders on the function of autologous arteriovenous endovascular fistula in uremic patients[D]. Chongqing Medical University,2021. | Not relevant to the topic |
|  | YUAN Yimeng,HAO Yan.Predictive value of the correlation between PLR and microinflammatory status on the function of endovascular fistula in patients on maintenance hemodialysis[J]. Medical Food Therapy and Health,2022,20(32):30-31. | Not relevant to the topic |
|  | GAN Danhui,JIN Jinxiao,SHAODuan,ZHU Chiling,ZHOU Huijun. Study on the predictive value of arteriovenous pressure monitoring on AVF stenosis in patients[J]. Zhejiang Trauma Surgery,2021,26(6):1070-1071.DOI:10.3969/j.issn.1009-7147.2021.06.027. | Not relevant to the topic |
|  | Chen K,Guo YC,Li XD. Study on the factors affecting the success rate of standard autologous arteriovenous endovascular fistula surgery[J]. World Digest of Recent Medical Information,2021,21(38):29-31.DOI:10.3969/j.issn.1671-3141.2021.38.015 . | Conference abstracts,etc |
|  | Wu J. Logistic regression analysis of risk factors for early loss of function of arteriovenous endovascular fistula in patients with type 2 diabetes mellitus[J]. Chinese Journal of Integrative Nephrology,2018,19(10):907-909.DOI:10.3969/j.issn.1009-587X.2018.10.022. | Not relevant to the topic |
|  | TANG Anna,TANG Bin.Observation on the effect of 50% magnesium sulfate combined with 75% alcohol wet compress in the treatment of hematoma of arteriovenous endovascular fistula[J]. Frontiers of medicine,2021,11(21):8-9,12. | Not relevant to the topic |
|  | XIE Jiaping,XU Weixi,YANG Jieke,et al. Clinical study on factors affecting autologous arteriovenous endovascular fistulae failure and prediction model in hemodialysis diabetic patients[J]. Shaanxi Medical Journal,2024,53(05):641-645. | Full text not available |
|  | LU Hao,HE Ying,LI Xiurong. Fine vascular arteriovenous endovascular fistuloplasty technique and preoperative and postoperative observation[J]. Chinese Journal of Continuing Medical Education,2018,41(3):219-222.DOI:10.3760/cma.j.issn.1673-4904.2018.03.008. | Not relevant to the topic |
|  | Gan Z. Clinical application study of percutaneous endoluminal angioplasty in hemodialysis patients with arteriovenous endovascular fistula loss [D]. Southeast University,2022. | Not relevant to the topic |
|  | GONG Jianhua,XU Qian,YUAN Hai. Relationship between long-term internal jugular vein cannulation thrombosis and platelet count and its activation in hemodialysis patients[J]. Chinese Journal of Gerontology,2012,32(22):4990-4992.DOI:10.3969/j.issn.1005-9202.2012.22.066. | Inconsistent with study population |
|  | WANG Tong,HE Da,XIE Nanxi,WAN Sheng,MA Wei,ZHAO Lan. Correlation analysis of normal heparin and low molecular heparin anticoagulation on arteriovenous endovascular fistula failure[J]. China Blood Purification,2012,11(4):226-227.DOI:10.3969/j.issn.1671-4091.2012.04.013. | Not relevant to the topic |
|  | Wang L-T,Wang Q. Risk factors for arteriovenous endovascular fistula thrombosis in hemodialysis patients and progress in preventive care[J]. Pharmacy Weekly,2021,30(33):142-143. | Inconsistent with study population |
|  | HE Li-Chang. Clinical analysis of arteriovenous fistula thrombosis leading to loss of function and distribution of body mass in traditional Chinese medicine[D]. Guangzhou University of Traditional Chinese Medicine,2013. | Inconsistent with study population |
|  | LIANG Cuiyun,YAN Biyan,MAI CuiFang. Effect of risk-graded management on the function of arteriovenous endovascular fistula in hemodialysis patients[J]. Maternal and Child Nursing,2023,3(1):189-192.DOI:10.3969/j.issn.2097-0838.2023.01.060. | Not relevant to the topic |
|  | Li JJ. Study on the correlation between preoperative and 1-day postoperative ultrasound detection indexes of autologous arteriovenous endovascular fistula and endovascular fistula maturation[D]. Zhengzhou University,2020. | Not relevant to the topic |
|  | WEI Shizhuo. Meta-analysis of the correlation between arteriovenous endovascular fistula malfunction and blood lipids in hemodialysis patients[D]. Jilin University,2022. | Not relevant to the topic |
|  | ZHANG Li-Na,LIU Ju-Hong,SHEN Ying-Jiao. Safety and efficacy of sodium bicarbonate combined with heparin sealing of hemodialysis semi-permanent catheter[J]. Chinese Journal of Integrative Nephrology,2023,24(12):1108-1111.DOI:10.3969/j.issn.1009-587X.2023.12.024. | Not relevant to the topic |
|  | WANG Li-Hua,JIANG Elie,LIU Xue-Ling,WEI Fang. Correlation analysis of arteriovenous endovascular fistula function and left ventricular function in maintenance hemodialysis patients[J]. Journal of Clinical Cardiovascular Disease,2014,30(8):709-711.DOI:10.13201/j.issn.1001-1439.2014.08.017. | Not relevant to the topic |
|  | Zhang Liangqin. The role of quality nursing intervention in the care of hemodialysis patients after balloon dilatation for stenosis of arteriovenous endovascular fistula[J]. Health Advice,2022,16(23):117-119. | Not relevant to the topic |
|  | ZHENG Xizi,DUAN Xiufang,WANG Jinwei,LIU Yao,MENG Li,YIN Yanqi,JIN Qizhuang. Correlation between blood flow and loss of function of arteriovenous endovascular fistula in hemodialysis patients monitored by ultrasonic dilution method and color Doppler ultrasound[J]. Chinese Journal of Nephrology,2022,38(12):1025-1031.DOI:10.3760/cma.j.cn441217-20220830-00150. | Not relevant to the topic |
|  | CHEN Dongping,YE Chaoyang,HUANG Di. Animal models for hemodialysis vascular access study[J]. Chinese Journal of Nephrology,2018,34(4):305-309.DOI:10.3760/cma.j.issn.1001-7097.2018.04.013. | Inconsistent with study population |
|  | Fu Yunchao. Risk factors for arteriovenous endovascular fistula thrombosis and their relationship with serum HMGB1, IL-1β, and IL-10 levels[J]. Pharmaceutical Biotechnology,2021,28(6):611-614.DOI:10.19526/j.cnki.1005-8915.20210612. | Inconsistent with study population |
|  | Liu Zhen. Effect of parathyroidectomy on the function of arteriovenous endovascular fistula in hemodialysis patients[D]. Chengde Medical College,2023. | Not relevant to the topic |
|  | Liu M. Study on factors affecting early loss of function of arteriovenous endocardial fistula in rural hemodialysis patients[D]. Zhengzhou University,2019. | Not relevant to the topic |
|  | Hsiao S.Y.,Liu J.H.. Role of color Doppler ultrasound technology in hemodialysis arteriovenous fistula[J]. Guangzhou Medicine,2009,40(4):38-40.DOI:10.3969/j.issn.1000-8535.2009.04.020. | Not relevant to the topic |
|  | YAN Yan,ZOU Menglin,HUANG Mingjun,XIAO Jun,ZHANG Li,ZENG Yan,YANG Yujuan,CHEN Qinkai. Factors affecting the service life of autologous arteriovenous endovascular fistula[J]. Chinese Journal of Nephrology,2020,36(4):300-305.DOI:10.3760/cma.j.cn441217-20191007-00057. | Not relevant to the topic |
|  | Fu, Qianli. Relationship between fibroblast growth factor 23 and Klotho protein and arteriovenous endovascular fistula loss in hemodialysis patients[D]. Guangdong Medical University,2019. | Not relevant to the topic |
|  | CHA Xiajing,NI Zhaohui,YAN Yucheng,GU Leyi,FANG Yan,WANG Yongmei,QIAN Jiaqi. Role of endovascular blood flow in hemodialysis patients in predicting endovascular prognosis[J]. China Blood Purification,2009,8(12):669-672.DOI:10.3969/j.issn.1671-4091.2009.12.010. | Not relevant to the topic |
|  | Zhan S,Zhao B,Wang H,Zhang LH,Wang YZ. Efficacy of peripheral cutting balloon in the treatment of long-segment stenosis of arteriovenous endovascular fistula and factors affecting restenosis after treatment[J]. Chinese Journal of Nephrology,2024,40(7):533-539.DOI:10.3760/cma.j.cn441217-20231120-01120. | Not relevant to the topic |
|  | WANG Xiaoyan,Lv Ping,Ge Zhilan,Jia Wei,Qian Muzhou. Application of modified blunt needle grommet method in hemodialysis endovascular fistula puncture[J]. International Journal of Transplantation and Blood Purification,2014,12(4):30-33.DOI:10.3760/cma.j.issn.1673-4238.2014.04.008. | Not relevant to the topic |
|  | Zou, L.,Yang, L.. Comparison of the application effect of three internal fistula puncture methods in hemodialysis patients[J]. Qilu Nursing Journal,2015,(14):20-22.DOI:10.3969/j.issn.1006-7256.2015.14.010. | Not relevant to the topic |
|  | Yang, Kun-Yin. Application of medical failure mode and effect analysis in preventing arteriovenous endovascular fistula failure[J]. Health Management,2021,(30):69-70,82. | Not relevant to the topic |
|  | Jiao, Sweetie. Predictive value of thrombin-antithrombin complex in restenosis after percutaneous transluminal angioplasty of arteriovenous fistula[D]. Zhengzhou University,2021. | Not relevant to the topic |
|  | Li Xing. Embolization of arteriovenous endovascular fistula in hemodialysis patients and the factors affecting it[J]. Today's Health,2021,(10):57-58. | Not relevant to the topic |
|  | Lai Miaoyu,Lai Xiuhong,Zhu Dexia. Application of immediate + repeated feedback teaching method in self-management of internal fistula in hemodialysis patients[J]. International Journal of Nursing,2017,36(9):1275-1278.DOI:10.3760/cma.j.issn.1673-4351.2017.09.040. | Not relevant to the topic |
|  | Liang W. Comparison of three different endovascular fistula puncture methods in hemodialysis[J]. Medical theory and practice,2014,(18):2399-2401. | Not relevant to the topic |
|  | Zhang Qian. Clinical study on the maturation timing of autologous arteriovenous endovascular fistula[D]. Hebei Medical University,2012. | Not relevant to the topic |
|  | Ye Zuyang. Study on cephalic vein intima-media thickness and early loss of function of arteriovenous endovascular fistula[D]. Guangxi University of Traditional Chinese Medicine,2020. | Not relevant to the topic |
|  | HUANG Chun-Xiang,YING Bo,ZHOU Jian-Fang. Analysis of factors affecting hemodialysis with polyester sleeve central venous indwelling catheter-associated bloodstream infection[J]. Chinese Journal of Integrative Nephrology,2017,18(2):158-159.DOI:10.3969/j.issn.1009-587X.2017.02.022. | Not relevant to the topic |
|  | Wen J,Yang T,Zhan S,Wang YZ. Relationship between stenosis of venous anastomosis of artificial endovascular fistula and venous vascular conditions[J]. China Blood Purification,2018,17(11):757-760.DOI:10.3969/j.issn.1671-4091.2018.11.009. | Not relevant to the topic |
|  | Zhang WM,Lu Y,Xu JF. Risk analysis and prevention of hemodialysis[J]. Electronic Journal of Integrative Cardiovascular Disease of Chinese and Western Medicine,2019,7(11):30,32. | Not relevant to the topic |
|  | Li Qiu. Study on the effects of different vascular access on microinflammatory state and infection in maintenance hemodialysis patients[D]. Southwest Medical University,2020. | Not relevant to the topic |
|  | YUAN Xinzhu,LI Lingqin,LI Boliang,WANG Yanjiang,LIN Changwei,XIE Xisheng. Meta-analysis of the effect of mucopolysaccharide polysulfate cream combined with physical therapy on the function of internal fistula[J]. Chinese Journal of Integrative Nephrology,2021,22(8):711-714.DOI:10.3969/j.issn.1009-587X.2021.08.018. | Not relevant to the topic |
|  | Ou Yuexiu. Survival analysis of arteriovenous endovascular fistula in hemodialysis patients with diabetic nephropathy[D]. Guangzhou Medical University,2018. | Not relevant to the topic |
|  | Ye W. Meta-analysis of the association between diabetes mellitus and arteriovenous endovascular fistula loss in dialysis patients[C]. Proceedings of the 17th East China Nephrology Forum and Shandong Province Nephrology Annual Conference.2017:644-644. | Conference abstracts,etc |
|  | Wei HG. Study on the correlation between anti-PF1/H antibody and thrombosis of arteriovenous endovascular fistula in dialysis patients[D]. Shandong University,2012. | Inconsistent with study population |
|  | Cui Gongzhen. Clinical study on serum troponin T/I in patients on maintenance hemodialysis[D]. Jilin University,2013. | Not relevant to the topic |
|  | Wang Xiao. Analysis of factors related to ultrasonographic characteristics and clinical significance of arteriovenous endovascular fistula in dialysis patients[D]. Shandong First Medical University,2021. | Not relevant to the topic |
|  | Wang Qian. Analysis of factors affecting nutritional risk in maintenance hemodialysis patients[D]. North China University of Science and Technology,2016. | Not relevant to the topic |
|  | Chen Xueqin.Study on the effect of COMMD5 on blood pressure after PTx in maintenance hemodialysis patients[D]. South China University of Technology,2021. | Not relevant to the topic |
|  | Li Y. Factors affecting left ventricular function in maintenance hemodialysis patients[D]. Chongqing Medical University,2019. | Not relevant to the topic |
|  | Wu YD. Mechanism study of growth differentiation factor 8 promoting mesenchymal stem cell activation in arteriovenous endovascular fistula stenosis[D]. South China University of Technology,2023. | Not relevant to the topic |
|  | PENG Fei.Application of 64-row spiral CT angiography in hemodialysis forearm arteriovenous fistula[C]. Proceedings of the 12th National Symposium on Integrative Medicine and Western Medicine Imaging.2012:1128-1132. | Conference abstracts,etc |
|  | Wang LN. Prevention and control strategy of hepatitis B in its infectious hemodialysis patients from the serological markers of hepatitis B virus[C].Proceedings of the 2018 Annual Meeting of the China Hospital Association Blood Purification Center Management Branch and the 10th China Blood Purification Forum, the 3rd Asia-Pacific Dialysis Access Conference & 2017 Henan Provincial Medical Association Blood Purification Academic Annual Conference.2018:1-2. | Conference abstracts,etc |
|  | Fan Yun Yun. Survival analysis of single-center arteriovenous endovascular fistula and the effect of qi and blood-boosting traditional Chinese medicine [D]. Guangzhou University of Traditional Chinese Medicine,2015. | Not relevant to the topic |
|  | Ma Panyue. Construction of light-controlled “self-degradation” nanoparticle hydrogel system and blocking of YAP signaling in vascular smooth muscle cells[D]. South China University of Technology,2021. | Not relevant to the topic |
|  | GUO Yongxin,FENG Peiyun,SHEN Wenling,et al. Analysis of risk factors for acute thrombosis of arteriovenous fistula in uremic patients on maintenance hemodialysis and construction of Nomogram prediction model[J]. Journal of Xinxiang Medical College,2024,41(05):472-476. | Full text not available |
|  | Jia Lan. Hemodynamic study of arteriovenous endovascular fistula dysfunction[D]. Tianjin Medical University,2015. | Not relevant to the topic |
|  | Huang Zhe. Prognostic study of hemodialysis filtration on maintenance dialysis patients[D]. Dalian Medical University,2020. | Not relevant to the topic |
|  | Zhen Zhang,Xuemei Wang,Zhiwei Zhang,Guoqing Du,Lan Wang,Jun Yang,Zongqian Wang. Hemodynamic evaluation of chronic hemodialysis autologous arteriovenous fistula by color Doppler ultrasound[J]. China Medical Imaging Technology,2006,22(5):718-721.DOI:10.3321/j.issn:1003-3289.2006.05.025. | Not relevant to the topic |
|  | Cui TY. Correlation study of factors influencing the initial loss of function of endovascular fistula in uremic patients[D]. Shandong First Medical University,2020. | Not relevant to the topic |
|  | CHENG Yuan. Application of CT thoracic vascular three-dimensional reconstruction for screening central venous stenosis[C]. Proceedings of the Ninth National Nephrology Symposium of the Chinese Medical Association Geriatric Branch.2011:110-111. | Conference abstracts,etc |
|  | Pan CJ. Clinical evaluation of the prognostic value of mean red blood cell width in arteriovenous endovascular fistula surgery[D]. Shandong University,2022. | Not relevant to the topic |
|  | Yin X. Diagnostic value of CT angiography for hemodialysis access complications[D]. Harbin Medical University,2014. | Not relevant to the topic |
|  | Jiang Xue. Correlation study of cardiac function and radial artery augmentation index in patients with end-stage renal disease[D]. Dalian Medical University,2010. | Not relevant to the topic |
|  | Yan Huihui. Causes of blood seepage from arteriovenous endovascular fistula puncture during hemodialysis and its nursing countermeasures[J]. Famous doctors,2023,(21):156-158. | Not relevant to the topic |
|  | YU Yang. Risk factors, clinical application of vascular stents and biomechanical evaluation of hemodialysis-related central venous occlusive disease[D]. Sichuan University,2021. | Not relevant to the topic |
|  | CAO Cuiming, WANG Yan, XU Mei, WEI Ricao, QIU Qiang, LI Minman, GAO Yuwei. Perioperative risk grading and intervention analysis of 102 cases of arteriovenous endovascular fistuloplasty[J]. People's military medicine,2016,59(5):494-495. | Not relevant to the topic |
|  | Guo Wang. Predictive role of hypotension in dialysis and difference between post-dialysis weight and target weight for endovascular fistula occlusion[C]. Proceedings of the 2019 Academic Annual Meeting of the Chinese Physicians Association Nephrologists Branch (CNA2019).2019:1-1. | Conference abstracts,etc |
|  | Ye JH. Analysis of clinical characteristics of stenosis of artificial arteriovenous endovascular fistula[C]. Proceedings of the Ninth China Blood Purification Forum and 2017 Annual Meeting of China Hospital Association Blood Purification Center Management Branch.2017:1-1. | Conference abstracts,etc |
|  | Xu Han. Analysis of influencing factors related to early patency rate of arteriovenous endovascular fistula[D]. Nanchang University,2012. | Not relevant to the topic |
|  | Xie Shurong. Study on the mechanism of high glucose-mediated hemodialysis access stenosis through up-regulation of Hedgehog signaling pathway[C]. Proceedings of the 18th National Conference on Experimental Hematology of the Chinese Society of Pathophysiology.2021:1-1. | Conference abstracts,etc |
|  | Li Yan. Effect of percutaneous transluminal angioplasty balloon dilatation pressure on the patency rate of arteriovenous endovascular fistula after PTA[D]. Hebei Medical University,2021. | Not relevant to the topic |
|  | PENG Fei.Application of 64-row spiral CT angiography in hemodialysis forearm arteriovenous fistula[C]. Proceedings of the 13th National Symposium on Integrative Imaging of Chinese and Western Medicine and the 8th Symposium on Integrative Imaging of Chinese and Western Medicine in Fujian Province.2014:387-391. | Conference abstracts,etc |
|  | XU Yan,FENG Sujuan,SONG Lei,ZHANG Peixuan. Application of risk management in hemodialysis nursing management[J]. World Digest of Recent Medical Information,2021,21(89):322-323.DOI:10.3969/j.issn.1671-3141.2021.89.157. | Conference abstracts,etc |
|  | Liu GQ, Deng XianQin, Liu HY, Liu WG, Hu YT, Huang WK, Chen WC. Dual-energy single-phase phase CTV of the upper extremity in hemodialysis access derangement[J]. Journal of Clinical Radiology,2019,38(3):542-546. | Not relevant to the topic |
|  | Yang J. Effects of turbulent flow on vascular endothelial cells in arteriovenous endovascular fistula[D]. Tianjin Medical University,2017. | Not relevant to the topic |
|  | Qiao Baoru. A study on the influencing factors for the early patency of AVF for dialysis[D]. Huazhong University of Science and Technology,2016. | Not relevant to the topic |
|  | Peng X. Exploring the value of color Doppler ultrasound in the detection of arteriovenous endovascular fistula[D]. Chongqing Medical University,2015. | Not relevant to the topic |
|  | Ren Wanjun. bFGF regulates the mechanism of arteriovenous fistula stenosis through TGF--β1/Smad3 signaling pathway[D]. Shandong University,2019. | Not relevant to the topic |
|  | Zheng FY. A preliminary study on the use of ambulatory blood pressure monitoring to improve the survival quality of hemodialysis patients with combined hypertension[D]. Wannan Medical College,2017. | Not relevant to the topic |
|  | Chen H. Correlation between B-type natriuretic peptide combined with ICG monitoring and prognosis of hemodialysis patients[D]. Tianjin Medical University,2015. | Not relevant to the topic |
|  | Dong JX,Ke D,Hu LZ,Liu YD,Fu QN. Anatomical factors and clinical significance of anatomical compression left cephalic brachial vein stenosis assessed based on CT[J]. Chinese Journal of Anatomy and Clinics,2021,26(6):616-622.DOI:10.3760/cma.j.cn101202-20200804-00254. | Not relevant to the topic |
|  | Tang L. Platelet activation in vascular access and dialysis membrane application in hemodialysis patients[D]. Suzhou University,2006. | Not relevant to the topic |
|  | LI Bingyu. Effects of integrin β3 on autophagy in vascular endothelial cells and its mechanism of action[D]. Chongqing Medical University,2020. | Not relevant to the topic |
|  | Cao Lei. Detection of IgG-H/PF4 antibodies in hemodialysis patients and its clinical significance[D]. Southern Medical University,2009. | Not relevant to the topic |
|  | Ren Hongqi. Analysis of the use of vascular access in patients treated with hemodialysis[D]. Second Military Medical University,2016. | Not relevant to the topic |
|  | He Liting.Experience sharing of 562 hemodialysis patients without catheter infection[C]. Proceedings of the 2019 Academic Annual Meeting of the Chinese Physicians Association Nephrologists Branch (CNA2019).2019:1-1. | Conference abstracts,etc |
|  | LI Yanyan,DU Guiying,FAN Yiyun,et al. Construction of risk factors and risk prediction model for restenosis after percutaneous transluminal angioplasty in patients with stenosis of arteriovenous endovascular fistula on hemodialysis[J]. Journal of Community Medicine,2024,22(14):481486.DOI:10.19790/j.cnki.JCM.2024.14.03. | Inconsistent with study population |
|  | Aljarrah Q, Al Bakkar L, Bakkar S, Abou-Foul AK, Allouh MZ. Perioperative Vascular Access Mapping in Patients with Hemodialysis: A Comparative Study of Access Navigation and Selection in Jordan. Vasc Health Risk Manag. 2024 Sep13;20:421-434. doi: 10.2147/VHRM.S480827. PMID: 39324109 | Not relevant to the topic |
|  | Shiri P, Rezaeian S, Abdi A, Jalilian M, Khatony A. Risk factors for thrombosis in dialysis patients: A comprehensive systematic review and meta- analysis. J Vasc Nurs. 2024 Sep;42(3):165-176. doi: 10.1016/j.jvn.2024.05.002. | Not relevant to the topic |
|  | Okuhata Y, Sakai Y, Ikenouchi A, Kashiwagi T, Iwabu M. Low Serum Albumin Levels are Associated with Short-Term Recurrence of Arteriovenous Fistula Failure. J Nippon Med Sch. 2024;91(4):383-390. doi:10.1272/jnms.JNMS.2024_91-408 | Not relevant to the topic |
|  | Pala AA, Urcun YS, Guven C. Evaluation of the relationship between proximal upper-extremity arteriovenous fistula patency and atherogenic index of plasma. Int J Artif Organs. 2024 Oct;47(10):743-748. doi: 10.1177/03913988241269534. | Not relevant to the topic |
|  | Liu S, Wang Y, He X, Wang Y, Li X. Factors affecting suboptimal maturation of autogenous arteriovenous fistula in elderly patients with diabetes:A narrative review. Heliyon. 2024 Aug 3;10(15):e35766. doi:10.1016/j.heliyon.2024.e35766. | Not relevant to the topic |
|  | Whitaker L, Sherman N, Ahmed I, Etkin Y. A review of the current recommendations and practices for hemodialysis access monitoring and maintenance procedures. Semin Vasc Surg. 2024 Jun;37(2):133-149. doi:10.1053/j.semvascsurg.2024.04.007 | Not relevant to the topic |
|  | Mestres G, Fontseré N, García-Madrid C, Campelos P, Maduell F, Riambau V. Intra-operative factors predicting 1-month arteriovenous fistula thrombosis. J Vasc Access. 2012 Apr-Jun;13(2):193-7. doi: 10.5301/jva.5000021. | Not relevant to the topic |
|  | Ye Q, Zou J, Li X, Guan W, Hu C, Zhao B, Li Y, Chen M, Su Q, Wu H. Effect of no-touch versus conventional technique on arteriovenous fistula outcomes: a randomised controlled trial. J Nephrol. 2024 Sep;37(7):1921-1928. doi:10.1007/s40620-024-02025-3. | Not relevant to the topic |
|  | Ohashi Y, Protack CD, Aoyagi Y, Gonzalez L, Thaxton C, Zhang W, Kano M, Bai H, Yatsula B, Alves R, Hoshina K, Schneider EB, Long X, Perry RJ, Dardik A. Heterogeneous gene expression during early arteriovenous fistula remodeling suggests that downregulation of metabolism predicts adaptive venous remodeling. Sci Rep. 2024 Jun 10;14(1):13287. doi: 10.1038/s41598-024-64075-8. | Not relevant to the topic |
|  | Wang KM, Gelabert H, Jimenez JC, Rigberg D, Woo K. Association of Frailty with Postoperative Survival and Outcomes Following Hemodialysis Vascular Access Creation. Ann Vasc Surg. 2024 Sep;106:184-188. doi: 10.1016/j.avsg.2024.03.028. | Not relevant to the topic |
|  | Richards J, Summers D, Sidders A, et al. Doppler ultrasound surveillance of recently formed haemodialysis arteriovenous fistula: the SONAR observational cohort study. Health Technol Assess. 2024 May;28(24):1-54. doi: 10.3310/YTBT4172. | Not relevant to the topic |
|  | Malikov S, Faure EM. Editor's Choice - Outcomes of Cold Stored Saphenous Vein Allografts for Haemodialysis Vascular Access. Eur J Vasc Endovasc Surg. 2024 Sep;68(3):397-404. doi: 10.1016/j.ejvs.2024.04.042. | Not relevant to the topic |
|  | Tayebi P, Dadashi K, Asgharpour M, Moghadamnia AA, Gholinia H, Bijani A. Distal Forearm Arteriovenous Fistula Maturation in Diabetic Hemodialysis Patients. Vasc Endovascular Surg. 2024 Aug;58(6):611-616. doi: 10.1177/15385744241251653. | Not relevant to the topic |
|  | Song W, Wu L, Sun C, Kong X, Wang H. New-onset atrial fibrillation following arteriovenous fistula increases adverse clinical events in dialysis patients with end-stage renal disease. Front Cardiovasc Med. 2024 Apr 12;11:1386304. doi: 10.3389/fcvm.2024.1386304. | Not relevant to the topic |
|  | Kanchanasuttirak P, Pitaksantayothin W, Kanchanasuttirak W. Accuracy of physical examination versus Doppler ultrasonography for determining maturity in postoperative arteriovenous fistula formation. Asian J Surg. 2024 Sep;47(9):3847-3851. doi: 10.1016/j.asjsur.2024.03.176. | Not relevant to the topic |
|  | Cheun TJ, Hart JP, Davies MG. Management of depth to achieve timely arteriovenous fistula utilization. J Vasc Surg. 2024 Aug;80(2):545-553.e3. doi: 10.1016/j.jvs.2024.03.445. | Not relevant to the topic |
|  | Haricharan RN, Aprahamian CJ, Morgan TL, Harmon CM, Barnhart DC. Intermediate-term patency of upper arm arteriovenous fistulae for hemodialysis access in children. J Pediatr Surg. 2008 Jan;43(1):147-51. doi: 10.1016/j.jpedsurg.2007.09.036. | Inconsistent with study population |
|  | Bae M, Lee CW, Chung SW, Huh U, Kim J, Jeong H, Lee NH. Rejoining Veins for Forced Maturation of Small-Caliber Arteriovenous Fistula. Ann Vasc Surg. 2024 Jul;104:268-275. doi: 10.1016/j.avsg.2024.01.007. | Not relevant to the topic |
|  | Lee H, Choi H, Han E, Kim YJ. Comparison of Clinical Effectiveness and Safety of Drug-Coated Balloons versus Percutaneous Transluminal Angioplasty in Arteriovenous Fistulae: A Review of Systematic Reviews and Updated Meta-Analysis. J Vasc Interv Radiol. 2024 Jul;35(7):949-962.e13. doi: 10.1016/j.jvir.2024.03.027. | Not relevant to the topic |
|  | Alsolami E, Alobaidi S. Hemodialysis nurses' knowledge, attitude, and practices in managing vascular access: A cross-sectional study in Saudi Arabia. Medicine (Baltimore). 2024 Mar 29;103(13):e37310. doi: 10.1097/MD.0000000000037310. | Not relevant to the topic |
|  | Jiang Y, Liu Z, Liu L, Xiong Z, Chen Y, Zhang S, Su C. Differential expression of serum TM, PAF, and CD62P in patients with autologous arteriovenous fistula and the correlation with vascular access function. Immun Inflamm Dis. 2024 Mar;12(3):e1227. doi: 10.1002/iid3.1227. | Not relevant to the topic |
|  | Bergmann M, Fakhoury B, Barroso T, Prushik SG, Jaber BL, Balakrishnan VS. Early access flow rate predicts vascular access patency-related intervention in the first year: A retrospective cohort study. Hemodial Int. 2024 Jul;28(3):262-269. doi: 10.1111/hdi.13148. | Not relevant to the topic |
|  | Koudounas G, Giannopoulos S, Volteas P, Aljobeh A, Karkos C, Virvilis D. Arteriovenous Fistula Maturation in Patients with Ipsilateral Versus Contralateral Tunneled Dialysis Catheter: A Systematic Review and Meta-analysis. Ann Vasc Surg. 2024 Jun;103:14-21. doi: 10.1016/j.avsg.2023.11.048. | Not relevant to the topic |
|  | Warner ED, Corsi DR, Jimenez D, Bierowski M, Brailovsky Y, Oliveros E, Alvarez RJ, Kumar V, Bhardwaj A, Rajapreyar IN. Determinants of pulmonary hypertension in patients with end-stage kidney disease and arteriovenous access. Curr Probl Cardiol. 2024 Apr;49(4):102406. doi: 10.1016/j.cpcardiol.2024.102406. | Not relevant to the topic |
|  | Sharma A, Sindwani G, Singh D, Mathur R, Bhardwaj A. Patency Rates and Outcomes of Renal Access Arteriovenous Fistulas for Hemodialysis in Patients with Chronic Kidney Disease. Saudi J Kidney Dis Transpl. 2023 May 1;34(3):201-206. doi: 10.4103/1319-2442.393992. | Not relevant to the topic |
|  | Tian Y, Shi K, Zhang Y, Cao Y, Zhou Y. Logistic regression analysis of risk factors for hematoma after autologous arteriovenous fistula in hemodialysis patients. Medicine (Baltimore). 2024 Jan 12;103(2):e36890. doi: 10.1097/MD.0000000000036890. | Not relevant to the topic |
|  | Hu X, Ding H, Wei Q, Chen R, Zhao W, Jiang L, Wang J, Liu H, Cao J, Liu H, Wang B. Fibroblast growth factor 21 predicts arteriovenous fistula functional patency loss and mortality in patients undergoing maintenance hemodialysis. Ren Fail. 2024 Dec;46(1):2302407. doi: 10.1080/0886022X.2024.2302407. | Not relevant to the topic |
|  | Tanyeri A. Association of radiocephalic arteriovenous anatomical markers with post-angioplasty blood flow volume. Acta Radiol. 2024 May;65(5):463-469. doi: 10.1177/02841851231223006. | Not relevant to the topic |
|  | Fisher AT, Mulaney-Topkar B, Sheehan BM, Garcia-Toca M, Sorial E, Sgroi MD. Association between heart failure and arteriovenous access patency in patients with end-stage renal disease on hemodialysis. J Vasc Surg. 2024 May;79(5):1187-1194. doi: 10.1016/j.jvs.2023.12.039. | Not relevant to the topic |
|  | Yasir MB, Man RK, Gogikar A, Nanda A, Niharika Janga LS, Sambe HG, Mohamed L. A Systematic Review Exploring the Impact of Arteriovenous Fistula Ligature on High-Output Heart Failure in Renal Transplant Recipients. Ann Vasc Surg. 2024 Mar;100:67-80. doi: 10.1016/j.avsg.2023.10.010. | Not relevant to the topic |
|  | Elbarbary MR, Ahmed LA, El-Adl DA, Ezzat AA, Nassib SA. Study of Osteopontin as a Marker of Arteriovenous Shunt Stenosis in Hemodialysis Patients. Curr Vasc Pharmacol. 2024;22(1):50-57. doi: 10.2174/0115701611260120231106081701. | Not relevant to the topic |
|  | Xu S, Wang J, Tang L, Cao W, Liang L, Wei K, Wang Z, Kong X. The RADAR technique in reconstruction of failed autologous arteriovenous fistulas due to juxta-anastomotic stenosis is equivalent to that with traditional surgery in maintenance hemodialysis patients. J Vasc Access. 2023 Nov 23:11297298231212225. doi: 10.1177/11297298231212225. | Not relevant to the topic |
|  | Ghandour H, Cataneo JL, Asha A, Jaeger JK, Jacobs CE, Schwartz LB, El Khoury R. Slowly moving the needle away from Fistula First. J Vasc Surg. 2024 Feb;79(2):382-387. doi: 10.1016/j.jvs.2023.11.007. | Not relevant to the topic |
|  | Suryawanshi M, Dutta P, Ganduboina R, Rajput V, Pawar SG. Standardization of Pre-operative Evaluation to Improve the Outcome of Arteriovenous Fistula for Vascular Access of Hemodialysis: A Review of 700 Cases. Cureus. 2023 Sep 26;15(9):e45999. doi: 10.7759/cureus.45999. | Not relevant to the topic |
|  | Tanyeri A. Early inadequate venous flow volume after technically successful balloon angioplasty of radiocephalic arteriovenous fistula: causes and follow-up results. Eur Rev Med Pharmacol Sci. 2023 Oct;27(19):9076-9084. doi: 10.26355/eurrev_202310_33933. | Not relevant to the topic |
|  | Zamboli P, Punzi M, Calabria M, Capasso M, Granata A, Lomonte C. Color Doppler ultrasound evaluation of arteriovenous grafts for hemodialysis. J Vasc Access. 2024 Nov;25(6):1721-1740. doi: 10.1177/11297298231178588. | Not relevant to the topic |
|  | Forcey D, Tran D, Connor J, Ayudhya PKN, Ocampo C, Nelson C, Crikis S. Improving assessment and escalation of threatened haemodialysis access: results of a nursing-led program. BMC Nephrol. 2023 Sep 13;24(1):268. doi: 10.1186/s12882-023-03321-z. | Not relevant to the topic |
|  | Forcey D, Tran D, Connor J, Ayudhya PKN, Ocampo C, Nelson C, Crikis S. Improving assessment and escalation of threatened haemodialysis access: results of a nursing-led program. BMC Nephrol. 2023 Sep 13;24(1):268. doi: 10.1186/s12882-023-03321-z. | Not relevant to the topic |
|  | Pfister M, d'Avalos LV, Müller PC, de Rougemont O, Bonani M, Kobe A, Puippe G, Nickel F, Rössler F. Long-term patency of arteriovenous fistulas for hemodialysis: A decade's experience in a transplant unit. Hemodial Int. 2023 Oct;27(4):388-399. doi: 10.1111/hdi.13110. | Not relevant to the topic |
|  | Evans LM, Raj R. A scoping review of outcomes with routine surveillance of arterio-venous fistulas. J Vasc Access. 2024 Sep;25(5):1409-1415. doi: 10.1177/11297298231188024. | Not relevant to the topic |
|  | Xing X, Li Q, Yang Y, Wang Y, Zhan X, Zhang C, Xu G, He F. Factors affecting the primary patency of native arteriovenous fistulas after ultrasound-guided percutaneous transluminal angioplasty. Ren Fail. 2023 Dec;45(1):2233623. doi: 10.1080/0886022X.2023.2233623. | Not relevant to the topic |
|  | Marques da Silva B, Dores M, Silva O, Pereira M, Outerelo C, Fortes A, Lopes JA, Gameiro J. Planning vascular access creation: The promising role of the kidney failure risk equation. J Vasc Access. 2024 Nov;25(6):1828-1834. doi: 10.1177/11297298231186373. | Not relevant to the topic |
|  | Kaller R, Russu E, Arbănași EM, Mureșan AV, Jakab M, Ciucanu CC, Arbănași EM, Suciu BA, Hosu I, Demian L, Horváth E. Intimal CD31-Positive Relative Surfaces Are Associated with Systemic Inflammatory Markers and Maturation of Arteriovenous Fistula in Dialysis Patients. J Clin Med. 2023 Jun 30;12(13):4419. doi: 10.3390/jcm12134419. | Not relevant to the topic |
|  | Hu S, Wang R, Ma T, Lei Q, Yuan F, Zhang Y, Wang D, Cheng J. Association between preoperative C-reactive protein to albumin ratio and late arteriovenous fistula dysfunction in hemodialysis patients: a cohort study. Sci Rep. 2023 Jul 11;13(1):11184. doi: 10.1038/s41598-023-38202-w. | Not relevant to the topic |
|  | Hafeez MS, Chaer RA, Eslami MH, Abdul-Malak OM, Yuo TH. Surgical and endovascular assisted maturation procedures improve cannulation after arteriovenous fistula creation, but not after arteriovenous graft placement. J Vasc Access. 2024 Sep;25(5):1649-1658. doi: 10.1177/11297298231185793. | Not relevant to the topic |
|  | GR, Lee T. Effects of a More Selective Arteriovenous Fistula Strategy on Vascular Access Outcomes. J Am Soc Nephrol. 2023 Sep 1;34(9):1589-1600. doi: 10.1681/ASN.0000000000000174. | Not relevant to the topic |
|  | Katsanos K, Ho P, Tang TY, Vlachou E, Yap CJQ, Kitrou PM, Karnabatidis D. Polymer-coated paclitaxel-eluting stents for the treatment of stenosed native arteriovenous fistulas: Long-term results from the ELUDIA study. J Vasc Access. 2024 Sep;25(5):1593-1600. doi: 10.1177/11297298231174263. | Not relevant to the topic |
|  | FENG Yulin,XU Xudong. Analysis of risk factors for the occurrence of autologous arteriovenous endovascular fistula failure in hemodialysis patients of long dialysis age[J]. Zhejiang Trauma Surgery,2024,29(07):1318-1320. | Full text not available |
|  | Allsopp K, Smith L. Did stopping ultrasound surveillance during COVID-19 result in an increase of the dialysis access thrombosis rate? J Vasc Access. 2024 Sep;25(5):1539-1543. doi: 10.1177/11297298231180326. | Not relevant to the topic |
|  | Long-term efficacy and risk factors of balloon-assisted maturation for radial-cephalic arteriovenous fistula with small-caliber veins. Hemodial Int. 2023 Jul;27(3):241-248. doi: 10.1111/hdi.13091. | Not relevant to the topic |
|  | Nguyen B, Duong MC, Diem Tran HN, Do KQ, Nguyen KTT. Arteriovenous fistula creation by nephrologist and its outcomes: a prospective cohort study from Vietnam. BMC Nephrol. 2023 Apr 4;24(1):88. doi: 10.1186/s12882-023-03123-3. | Not relevant to the topic |
|  | Chen H, Song N, Li AM, Dai Q, Liu YT, Yang SK. Association of vitamin D metabolites with arteriovenous fistula function in hemodialysis patients: A single center study. Hemodial Int. 2023 Jul;27(3):231-240. doi: 10.1111/hdi.13080. | Not relevant to the topic |
|  | Wei S, Liu N, Fu Y, Sun M. Novel insights into modifiable risk factors for arteriovenous fistula failure and the importance of CKD lipid profile: A meta-analysis. J Vasc Access. 2024 Sep;25(5):1416-1431. doi: 10.1177/11297298221115557. | Not relevant to the topic |
|  | Wei S, Jiao J, Yu Y, Tian X, Yang X, Feng S, Li Y, Zhang P, Bai M. Long-term arteriovenous fistula prognosis for maintenance hemodialysis patients who accepted PIRRT by using arteriovenous fistula. Int J Artif Organs. 2023 Apr;46(4):195-201. doi: 10.1177/03913988231162384. | Not relevant to the topic |
|  | Badak TO, Ada S. Malnutrition: A risk factor for vascular access problems. J Vasc Access. 2024 Jul;25(4):1279-1284. doi: 10.1177/11297298231154631. | Not relevant to the topic |
|  | Mendes D, Almeida P, Sousa C, Loureiro L, Teixeira S, Rego D, Norton-de-Matos A. Outflow Rescue of Elbow-Blockade Forearm Arteriovenous Fistulas: Outcomes of Open Surgical Techniques. Ann Vasc Surg. 2023 Aug;94:280-288. doi: 10.1016/j.avsg.2023.02.028. | Not relevant to the topic |
|  | Gunawardena T, Sharma H, Mehra S. Outcomes of operative intervention for ulcers over hemodialysis arteriovenous access. Hemodial Int. 2023 Apr;27(2):112-116. doi: 10.1111/hdi.13075. | Not relevant to the topic |
|  | Huang XM, Zhang Y, Du M, Gu LQ, Fu HL, Yu F, Xu L, Li JJ, Wang Y, Sun XF. Prognosis of Heart Valve Calcification on Cardiovascular Events in Hemodialysis Patients without Central Venous Catheters. Cardiorenal Med. 2023;13(1):38-45. doi: 10.1159/000529136. | Not relevant to the topic |
|  | Corr M, Pachchigar A, O'Neill M, Higgins R, O'Neill S, Hanko J, Masengu A. A decade of arteriovenous fistula creations in the ⩾75 years population: Equal opportunity or sub-optimal use of resources. J Vasc Access. 2024 Jul;25(4):1093-1099. doi: 10.1177/11297298221147571 | Not relevant to the topic |
|  | Yii E, Lee L, Polkinghorne K, Thwaites S, Saunder A, Yii MK. Optimal flow volume measurements in forearm versus arm arteriovenous fistulas. Nephrology (Carlton). 2023 Mar;28(3):175-180. doi: 10.1111/nep.14142. | Not relevant to the topic |
|  | Li D, Hao M, Sheng H, Ge H, Zhu Y. Lumen diameter is associated with the patency after percutaneous angioplasty of arteriovenous fistulas. J Vasc Access. 2024 Jul;25(4):1087-1092. doi: 10.1177/11297298221112531. | Not relevant to the topic |
|  | Hou G, Fu M, Wang X, Liu Z, Zhang Y, Zhu D, Pang H, Li R, Shen L. Modified no-touch technique for radio-cephalic arteriovenous fistula increases primary patency and decreases juxta-anastomotic stenosis. J Vasc Access. 2024 May;25(3):904-913. doi: 10.1177/11297298221139339. | Not relevant to the topic |
|  | Gubensek J. Doppler ultrasound assessment of calcified radial arteries prior to radio-cephalic arterio-venous fistula placement: an observational study. J Vasc Access. 2024 May;25(3):897-903. doi: 10.1177/11297298221143598. | Not relevant to the topic |
|  | Deogaonkar G, Thulasidasan N, Phulambrikar R, Diamantopoulos A, Sran K, Ahmed I, Loukopoulos I, Karunanithy N. Endovascular salvage of thrombosed haemodialysis vascular access. Vasa. 2023 Jan;52(1):63-70. doi: 10.1024/0301-1526/a001043. | Not relevant to the topic |
|  | Kim SM, Park PJ, Kim HK. Comparison between radiocephalic and brachiocephalic arteriovenous fistula in octogenarians: A retrospective single center study. J Vasc Access. 2024 May;25(3):849-853. doi: 10.1177/11297298221139055. | Not relevant to the topic |
|  | Luo F, Huang C, Yao G, Zhou J, Lu X. Comparison of percutaneous transluminal angioplasty and surgical revision after intraoperative dilatation with biliary tract probes for arteriovenous fistula stenosis at juxta-anastomosis. Vascular. 2024 Apr;32(2):467-474. doi: 10.1177/17085381221140179. | Not relevant to the topic |
|  | Chang R, Alabi O, Mahajan A, Miller JS, Bhat KR, Mize BM, Khader MA, Teodorescu V. Arteriovenous fistula aneurysmorrhaphy is associated with improved patency and decreased vascular access abandonment. J Vasc Surg. 2023 Mar;77(3):891-898.e1. doi: 10.1016/j.jvs.2022.10.054. | Not relevant to the topic |
|  | Taurisano M, Mancini A, D'elia F. CEUS-guided PTA on stenotic AVF: Morphological and functional point of view. J Vasc Access. 2024 Mar;25(2):576-583. doi: 10.1177/11297298221126289. | Not relevant to the topic |
|  | Salikhova TY, Pushin DM, Nesterenko IV, Biryukova LS, Guria GT. Patient specific approach to analysis of shear-induced platelet activation in haemodialysis arteriovenous fistula. PLoS One. 2022 Oct 3;17(10):e0272342. doi: 10.1371/journal.pone.0272342. | Not relevant to the topic |
|  | Park JH, Park I, Han K, Yoon J, Sim Y, Kim SJ, Won JY, Lee S, Kwon JH, Moon S, Kim GM, Kim MD. Feasibility of Deep Learning-Based Analysis of Auscultation for Screening Significant Stenosis of Native Arteriovenous Fistula for Hemodialysis Requiring Angioplasty. Korean J Radiol. 2022 Oct;23(10):949-958. doi: 10.3348/kjr.2022.0364. | Not relevant to the topic |
|  | Lazar AN, Johnson AP, Morrissey NJ. Association of insurance status with timing of hemodialysis access placement. J Vasc Surg. 2021 Oct;74(4):1309-1316.e2. doi: 10.1016/j.jvs.2021.05.063. | Conference abstracts,etc |
|  | Rozenberg I, Benchetrit S, Raigorodetsky M, Fajer S, Shnaker A, Nacasch N, Einbinder Y, Zitman-Gal T, Cohen-Hagai K. Clinical Outcomes of Vascular Accesses in Hemodialysis Patients. Isr Med Assoc J. 2022 Aug;24(8):514-519. | Not relevant to the topic |
|  | Martinez LI, Esteve V, Yeste M, Artigas V, Llagostera S. Clinical Utility of a New Predicting Score for Radiocephalic Arteriovenous Fistula Survival. Ann Vasc Surg. 2017 May;41:56-61. doi: 10.1016/j.avsg.2016.09.022. | Conference abstracts,etc |
|  | Cahalane AM, Abboud SE, Kawai T, Yeh H, Dageford LA, Kimura S, Steele DJR, Kalva SP, Irani Z, Cui J. Stent Diameter, Not Cephalic Arch Anatomy, Predicts Stent Graft Patency in Cephalic Arch Stenosis. J Vasc Interv Radiol. 2022 Nov;33(11):1321-1328.e1. doi: 10.1016/j.jvir.2022.07.014. | Not relevant to the topic |
|  | Calabrese V, Micali C, Russotto Y, Laganà N, Gullotta C, Pisano A, Santoro D, Nunnari G, Venanzi Rullo E. Arteriovenous fistula thrombosis in hemodialysis patients with COVID-19: epiphenomenon or marker of severe clinical disease? G Ital Nefrol. 2022 Jun 20;39(3):2022-vol3. | Not relevant to the topic |
|  | Brastauskas IM, Patel N, German Z, Davis RP, Stafford JM, Edwards M, Murea M, Goldman MP. A Single-Center Experience with Forearm Arteriovenous Loop Grafts for Hemodialysis. Ann Vasc Surg. 2022 Nov;87:286-294. doi: 10.1016/j.avsg.2022.06.003. | Not relevant to the topic |
|  | Kamath N, Naik N, Iyengar A. Clinical profile and outcome of arteriovenous fistulae in children on maintenance hemodialysis from a low-resource setting. J Vasc Surg. 2022 Dec;76(6):1699-1703. doi: 10.1016/j.jvs.2022.06.098. | Inconsistent with study population |
|  | Mokhtari S, Besancenot A, Beaumont M, Leroux F, Rinckenbach S, Salomon Du Mont L. Snuff-Box Versus Wrist Radiocephalic Arteriovenous Fistulas for Hemodialysis: Maturation Tend and its Affecting Factors. Ann Vasc Surg. 2022 Nov;87:495-501. doi: 10.1016/j.avsg.2022.05.032. | Not relevant to the topic |
|  | Tang QH, Yang H, Chen J, Lin QN, Qin Z, Hu M, Qin X. Comparison between transposed arteriovenous fistulas and arteriovenous graft for the hemodialysis patients: A meta-analysis and systematic review. J Vasc Access. 2024 Mar;25(2):369-389. doi: 10.1177/11297298221102875. | Not relevant to the topic |
|  | Liu J, Zhang D, Brahmandam A, Matsubara Y, Gao M, Tian J, Liu B, Shu C, Dardik A. Bioinformatics identifies predictors of arteriovenous fistula maturation. J Vasc Access. 2024 Jan;25(1):172-186. doi: 10.1177/11297298221102298. | Not relevant to the topic |
|  | Garza B, Geer J, Swartz SJ, Srivaths P, Huynh TTT, Brewer ED. Good outcomes for arteriovenous fistula with buttonhole cannulation for chronic hemodialysis in children and adolescents. Pediatr Nephrol. 2023 Feb;38(2):509-517. doi: 10.1007/s00467-022-05580-8. | Inconsistent with study population |
|  | Luo Q, Liu H, Yang Q. Analysis of Factors Influencing Restenosis after Percutaneous Transluminal Angioplasty. Blood Purif. 2022;51(12):1031-1038. doi: 10.1159/000524159. | Not relevant to the topic |
|  | Stavert B, Monaro S, Naganathan V, Aitken S. Frailty predicts increased risk of reintervention in the 2 years after arteriovenous fistula creation. J Vasc Access. 2023 Nov;24(6):1428-1437. doi: 10.1177/11297298221088756. | Not relevant to the topic |
|  | Kuningas K, Stringer S, Cockwell P, Khawaja A, Inston N. Is there a role of the kidney failure risk equation in optimizing timing of vascular access creation in pre-dialysis patients? J Vasc Access. 2023 Nov;24(6):1305-1313. doi: 10.1177/11297298221084799. | Not relevant to the topic |
|  | Ge L, Fang Y, Rao S. A Retrospective Case-Control Study on Late Failure of Arteriovenous Fistula in Hemodialysis Patients and Prediction of Risk Factors. Comput Math Methods Med. 2022 Mar 8;2022:8110289. doi: 10.1155/2022/8110289. Retraction in: Comput Math Methods Med. 2023 Jul 19;2023:9873820. doi: 10.1155/2023/9873820. | Not relevant to the topic |
|  | Sharif Nia H, Kohestani D, Froelicher ES, Ibrahim FM, Ibrahim MM, Bayat Shahparast F, Goudarzian AH. The Relationship Between Self-Care Behavior and Concerns About Body Image in Patients Undergoing Hemodialysis in Iran. Front Public Health. 2022 Mar 4;10:825415. doi: 10.3389/fpubh.2022.825415. | Not relevant to the topic |
|  | Wang Q, Lin J, Han H, Wu D, Zhou Y, Zhao B. Preoperative Cardiac Index as a Predictor of Maturation and Primary Patency of Radiocephalic Arteriovenous Fistula in Hemodialysis. Blood Purif. 2022;51(11):932-942. doi: 10.1159/000521956. | Not relevant to the topic |
|  | Calotta NA, Astor BC, Ross JR. Automated 3D ultrasound enables novice users to measure arteriovenous fistula maturation parameters with comparable accuracy to conventional duplex by trained sonographers: Results of a benchtop study. J Vasc Access. 2023 Nov;24(6):1398-1406. doi: 10.1177/11297298221074462. | Not relevant to the topic |
|  | Luo CM, Hsieh MY, Cheng CH, Chen CH, Liao MT, Chuang SY, Wu CC. Association of Frailty With Thrombosis of Hemodialysis Vascular Access: A Prospective Taiwanese Cohort Study. Am J Kidney Dis. 2022 Sep;80(3):353-363.e1. doi: 10.1053/j.ajkd.2021.12.017. | Not relevant to the topic |
|  | Shembekar SN, Zodpe DB, Padole PM. Prediction of the anastomosis angle of arteriovenous fistula in hemodialysis to standardize the surgical technique. Biomed Mater Eng. 2022;33(5):423-436. doi: 10.3233/BME-211389. | Not relevant to the topic |
|  | Hakim AJ, Brooke BS, Beckstrom JL, Sarfati MR, Kraiss LW. Rules of 6 criteria predict dialysis fistula maturation but not all rules are equal. J Vasc Surg. 2022 Jul;76(1):232-238.e2. doi: 10.1016/j.jvs.2022.02.018. | Not relevant to the topic |
|  | G. Bhatia, M. Wagle, N. Jethnani, J. Bhagtani and A. Chandak, "Machine Learning for Prediction of Life of Arteriovenous Fistula," 2018 3rd International Conference for Convergence in Technology (I2CT), Pune, India, 2018, pp. 1-6, doi: 10.1109/I2CT.2018.8529386. | Conference abstracts,etc |
|  | Chuang YC, Chen JB, Yang LC, Kuo CY. Significance of platelet activation in vascular access survival of haemodialysis patients. Nephrol Dial Transplant. 2003 May;18(5):947-54. doi: 10.1093/ndt/gfg056. | Conference abstracts,etc |
|  | Mo H, Ahn S, Han A, Min S, Min SK, Jung IM. Outcome after early thrombotic occlusion of arteriovenous fistulas. J Vasc Access. 2023 Nov;24(6):1268-1274. doi: 10.1177/11297298221076582. | Not relevant to the topic |
|  | Soo Hoo AJ, Scully RE, Sharma G, Patterson S, Walsh J, Voiculescu A, Belkin M, Menard M, Keith Ozaki C, Hentschel DM. Contemporary outcomes of precision banding for high flow hemodialysis access. J Vasc Access. 2023 Nov;24(6):1260-1267. doi: 10.1177/11297298221076581. | Not relevant to the topic |
|  | de Castro-Santos G, Shiomatsu GY, Oliveira RMDS, Procópio RJ, Navarro TP. Intraoperative vascular Doppler ultrasound blood flow and peak systolic velocity predict early patency in hemodialysis arteriovenous fistula. J Vasc Bras. 2022 Jan 7;20:e20210098. doi: 10.1590/1677-5449.210098. | Not relevant to the topic |
|  | Anapalli SR, N HD, Sarma P, Srikanth L, V SK. Thrombophilic risk factors and ABO blood group profile for arteriovenous access failure in end stage kidney disease patients: a single-center experience. Ren Fail. 2022 Dec;44(1):34-42. doi: 10.1080/0886022X.2021.2011746. | Not relevant to the topic |
|  | Heggen BD, Ramspek CL, van der Bogt KEA, de Haan MW, Hemmelder MH, Hiligsmann MJC, van Loon MM, Rotmans JI, Tordoir JHM, Dekker FW, Schurink GWH, Snoeijs MGJ; OASIS study group. Optimising Access Surgery in Senior Haemodialysis Patients (OASIS): study protocol for a multicentre randomised controlled trial. BMJ Open. 2022 Feb 3;12(2):e053108. doi: 10.1136/bmjopen-2021-053108. | Not relevant to the topic |
|  | Soma Y, Murakami M, Nakatani E, Sato Y, Tanaka S, Mori K, Sugawara A. Brachial artery transposition versus catheters as tertiary vascular access for maintenance hemodialysis: a single-center retrospective study. Sci Rep. 2022 Jan 10;12(1):306. doi: 10.1038/s41598-021-03860-1. | Not relevant to the topic |
|  | Carleton J, Chang J, Richard Pu Q, Rhee R. Internal jugular to internal jugular vein bypass of symptomatic central vein obstruction. J Vasc Access. 2022 Jan 10:11297298211070703. doi: 10.1177/11297298211070703. | Not relevant to the topic |
|  | Riding AM, Al-Nowfal A, Ramanarayanan S, Swift O, Mathavakkannan S, Jeevaratnam P, Steiner K. A single-centre, retrospective analysis of mortality over 80 months comparing paclitaxel-coated balloon versus standard balloon angioplasty in the treatment of dysfunctional arteriovenous access. J Vasc Access. 2021 Dec 30:11297298211066749. doi: 10.1177/11297298211066749. | Not relevant to the topic |
|  | Woo K, Gascue L, Norris K, Lin E. Patient Frailty and Functional Use of Hemodialysis Vascular Access: A Retrospective Study of the US Renal Data System. Am J Kidney Dis. 2022 Jul;80(1):30-45. doi: 10.1053/j.ajkd.2021.10.011. | Not relevant to the topic |
|  | Li M, Sun C, Du X. Application Value and Relevance Analysis of the Risk Evaluation System for Arteriovenous Fistula Puncture in Thrombosis after Puncture. J Healthc Eng. 2021 Dec 2;2021:6919979. doi: 10.1155/2021/6919979. | Not relevant to the topic |
|  | Plumb TJ, Adelson AB, Groggel GC, Johanning JM, Lynch TG, Lund B. Obesity and hemodialysis vascular access failure. Am J Kidney Dis. 2007 Sep;50(3):450-4. doi: 10.1053/j.ajkd.2007.06.010. | Not relevant to the topic |
|  | Yii E, Yii MK, Thwaites S, Zhu J, Chong T, Tong L, Nair S. Receiver operating characteristic curve analysis of arteriovenous dialysis access flow using ultrasound. ANZ J Surg. 2022 Mar;92(3):461-465. doi: 10.1111/ans.17378. | Not relevant to the topic |
|  | Ticala M, Rusu CC, Moldovan D, Potra AR, Tirinescu DC, Coman AL, Bondor CI, Budisan L, Kacsó IM. Hemodialysis Patients with Higher Serum Levels of Soluble Receptor for Advanced Glycation End Products Have an Increased Risk for Arteriovenous Fistula Failure. Blood Purif. 2022;51(9):764-771. doi: 10.1159/000519879. | Not relevant to the topic |
|  | Gan W, Shao D, Xu L, Tuo Y, Mao H, Wang W, Xiao W, Xu F, Huang X, Chen W, Zeng X. Maturation and survival of arteriovenous fistula: The challenge starts from the preoperative assessment stage. Semin Dial. 2022 May;35(3):228-235. doi: 10.1111/sdi.13036. | Not relevant to the topic |
|  | Christian Israel Alfaro Sánchez, Antonio José Marín Franco, Mª Paz Sorribes, Shaira Martínez-Vaquera, #2833 Charlson comorbidity index predicts early failure and overall survival of vascular access among diabetic dialysis patients., Nephrology Dialysis Transplantation, Volume 39, Issue Supplement_1, May 2024, gfae069–1552–2833, https://doi.org/10.1093/ndt/gfae069.1552 | Conference abstracts,etc |
|  | Lynn KL, Buttimore AL, Wells JE, Inkster JA, Roake JA, Morton JB. Long-term survival of arteriovenous fistulas in home hemodialysis patients. Kidney Int. 2004 May;65(5):1890-6. doi: 10.1111/j.1523-1755.2004.00597.x. | Conference abstracts,etc |
|  | Murali S, Shenoy SV, Prabhu RA, Nagaraju SP. Hypertensive emergency and seizures during haemodialysis. BMJ Case Rep. 2021 Sep 21;14(9):e242471. doi: 10.1136/bcr-2021-242471. | Conference abstracts,etc |
|  | Dixon BS, Novak L, Fangman J. Hemodialysis vascular access survival: upper-arm native arteriovenous fistula. Am J Kidney Dis. 2002 Jan;39(1):92-101. doi: 10.1053/ajkd.2002.29886. | Conference abstracts,etc |
|  | Lindhard K, Hansen D, Lindegaard Pedersen B, Rix M, Hansen HP, Jensen BL, Heaf J. Stable incidence and survival of arteriovenous fistulas over 39 years: A long-term national cohort study. J Vasc Access. 2023 Jul;24(4):620-629. doi: 10.1177/11297298211046102. | Not relevant to the topic |
|  | Etkin Y, Talathi S, Rao A, Akerman M, Lesser M, Mussa FF, Landis GS. The Role of Duplex Ultrasound in Assessing AVF Maturation. Ann Vasc Surg. 2021 Apr;72:315-320. doi: 10.1016/j.avsg.2020.10.006. | Conference abstracts,etc |
|  | Caputo BC, Leong B, Sibona A, Jhajj S, Kohne C, Gabel J, Shih W, Abou Zamzam A, Bianchi C, Teruya T. Arteriovenous fistula maturation: Physical exam versus flow study. Ann Vasc Surg. 2021 Nov;77:16-24. doi: 10.1016/j.avsg.2021.05.022. | Not relevant to the topic |
|  | Khanfar O, Aydi R, Saada S, Shehada M, Hamdan Z, Sawalmeh O, Hassan M, Hammoudi A, Nazzal Z. Mid-term cumulative patency of fistula and PTFE grafts among hemodialysis patients: A retrospective, single-center study from Palestine. J Vasc Access. 2023 Jul;24(4):559-567. doi: 10.1177/11297298211040988. | Not relevant to the topic |
|  | Zhu F, Yao Y, Ci H, Shawuti A. Predictive value of neutrophil-to-lymphocyte ratio and platelet-to-lymphocyte ratio for primary patency of percutaneous transluminal angioplasty in hemodialysis arteriovenous fistula stenosis. Vascular. 2022 Oct;30(5):920-927. doi: 10.1177/17085381211039672. | Not relevant to the topic |
|  | Chen L, Zhang W, Tan J, Hu M, Shi W, Zhang M, Wang Y, Yu B, Chen J. Morphological Lesion Types Are Associated with Primary and Secondary Patency Rates after High-Pressure Balloon Angioplasty for Dysfunctional Arteriovenous Fistulas. Blood Purif. 2022;51(5):425-434. doi: 10.1159/000516883. | Not relevant to the topic |
|  | Hammes M, Moya-Rodriguez A, Bernstein C, Nathan S, Navuluri R, Basu A. Computational modeling of the cephalic arch predicts hemodynamic profiles in patients with brachiocephalic fistula access receiving hemodialysis. PLoS One. 2021 Jul 14;16(7):e0254016. doi: 10.1371/journal.pone.0254016. | Not relevant to the topic |
|  | Mauro R, Rocchi C, Vasuri F, Pini A, Croci Chiocchini AL, Ciavarella C, La Manna G, Pasquinelli G, Faggioli G, Gargiulo M. Tissue Ki67 proliferative index expression and pathological changes in hemodialysis arteriovenous fistulae: Preliminary single-center results. J Vasc Access. 2023 May;24(3):391-396. doi: 10.1177/11297298211015495 | Not relevant to the topic |
|  | Staaf K, Fernström A, Uhlin F. Cannulation technique and complications in arteriovenous fistulas: a Swedish Renal Registry-based cohort study. BMC Nephrol. 2021 Jul 7;22(1):256. doi: 10.1186/s12882-021-02458-z. | Not relevant to the topic |
|  | Nishida H, Fukuhara H, Nawano T, Kanno H, Yagi M, Yamagishi A, Sakurai T, Naito S, Kato T, Kudo K, Ichikawa K, Tsuchiya N. Adjuvant heparinization before manipulation of artery reduces early failure in primary arteriovenous fistula for end-stage renal disease patients. Clin Exp Nephrol. 2021 Dec;25(12):1346-1353. doi: 10.1007/s10157-021-02109-5. | Not relevant to the topic |
|  | Nishida H, Fukuhara H, Nawano T, Kanno H, Yagi M, Yamagishi A, Sakurai T, Naito S, Kato T, Kudo K, Ichikawa K, Tsuchiya N. Adjuvant heparinization before manipulation of artery reduces early failure in primary arteriovenous fistula for end-stage renal disease patients. Clin Exp Nephrol. 2021 Dec;25(12):1346-1353. doi: 10.1007/s10157-021-02109-5. | Not relevant to the topic |
|  | Chen FY, Chen CF, Tan AC, Chan CH, Chen FA, Liu WS, Chen TH, Ou SM, Li SY, Tsai MT, Chen YT, Lin CC. Long-term prognosis of vascular access in hemodialysis patients with systemic lupus erythematosus: a retrospective cohort study. Sci Rep. 2021 Jun 15;11(1):12519. doi: 10.1038/s41598-021-92005-5. | Not relevant to the topic |
|  | Li C, Li Q, Ou J, Li W, Guan B, Lu Y, Yun C, Hocher B, Hu B, Yin L. Relationship between Monocytes and Stenosis-Related Autologous Arteriovenous Fistula Dysfunction. Blood Purif. 2022;51(3):226-232. doi: 10.1159/000514059. | Not relevant to the topic |
|  | Güven C, Pala AA, Urcun YS. Effects of plasma atherogenic index and plasma osmolality on arteriovenous fistula patency in hemodialysis patients. J Vasc Access. 2023 Jan;24(1):64-70. doi: 10.1177/11297298211011864. | Not relevant to the topic |
|  | Williams D, Leuthardt EC, Genin GM, Zayed M. Tailoring of arteriovenous graft-to-vein anastomosis angle to attenuate pathological flow fields. Sci Rep. 2021 Jun 9;11(1):12153. doi: 10.1038/s41598-021-90813-3. | Not relevant to the topic |
|  | Copeland TP, Hye RJ, Lawrence PF, Woo K. Association of Race and Ethnicity with Vascular Access Type Selection and Outcomes. Ann Vasc Surg. 2020 Jan;62:142-147. doi: 10.1016/j.avsg.2019.08.068. | Conference abstracts,etc |
|  | Weaver ML, Holscher CM, Sorber RA, Lum YW, Reifsnyder T. Redo Hemodialysis Access in Elderly Patients has Acceptable Outcomes With Similar Patency of Arteriovenous Fistulas as Compared to Grafts. Ann Vasc Surg. 2021 Oct;76:128-133. doi: 10.1016/j.avsg.2021.04.028. | Not relevant to the topic |
|  | Lazarides MK, Christaina E, Antoniou GA, Argyriou C, Trypsianis G, Georgiadis GS. Plain versus paclitaxel-coated balloon angioplasty in arteriovenous fistula and graft stenosis: An umbrella review. J Vasc Access. 2022 Nov;23(6):981-988. doi: 10.1177/11297298211005290. | Not relevant to the topic |
|  | Weaver ML, Holscher CM, Sorber RA, Lum YW, Reifsnyder T. Redo Hemodialysis Access in Elderly Patients has Acceptable Outcomes With Similar Patency of Arteriovenous Fistulas as Compared to Grafts. Ann Vasc Surg. 2021 Oct;76:128-133. doi: 10.1016/j.avsg.2021.04.028. | Not relevant to the topic |
|  | Taurisano M, Mancini A, D'Elia F. [Parameters predicting arterious-venous fistula maturation in pre surgery vascular mapping]. G Ital Nefrol. 2021 Apr 14;38(2):2021-vol2. Italian. | Not relevant to the topic |
|  | Kim HK, Han A, Ahn S, Ko H, Chung CTY, Choi KW, Min S, Ha J, Min SK. Better Efficacy of Balloon Assisted Maturation in Radial-Cephalic Arteriovenous Fistula for Hemodialysis. Vasc Specialist Int. 2021 Mar 31;37(1):29-36. doi: 10.5758/vsi.210003. | Not relevant to the topic |
|  | Spatola L, Rivera RF, Mugnai G. Cardiovascular implantable electronic devices and native arteriovenous fistula in hemodialysis patients: novel perspectives. Int Urol Nephrol. 2021 Dec;53(12):2541-2548. doi: 10.1007/s11255-021-02830-w. | Not relevant to the topic |
|  | Ghosh NK, Bhattacharjee HK, Prajapati O, Krishna A, Kumar A, Mahajan S, Bansal VK. Impact of clinical parameters and vascular haemodynamics on arterio-venous fistula maturation in patients with end stage renal disease: A prospective study on Indian patients. J Vasc Access. 2022 Jul;23(4):508-514. doi: 10.1177/11297298211001158. | Not relevant to the topic |
|  | Heindel P, Dieffenbach BV, Sharma G, Belkin M, Ozaki CK, Hentschel DM. Contemporary outcomes of a "snuffbox first" hemodialysis access approach in the United States. J Vasc Surg. 2021 Sep;74(3):947-956. doi: 10.1016/j.jvs.2021.01.069. | Not relevant to the topic |
|  | Yap YS, Chi WC, Lin CH, Liu YC, Wu YW. Association of early failure of arteriovenous fistula with mortality in hemodialysis patients. Sci Rep. 2021 Mar 11;11(1):5699. doi: 10.1038/s41598-021-85267-6. | Not relevant to the topic |
|  | Liu CT, Hsu SC, Hsieh HL, Chen CH, Chen CY, Sue YM, Lin FY, Shih CM, Shiu YT, Huang PH. Parathyroid Hormone Induces Transition of Myofibroblasts in Arteriovenous Fistula and Increases Maturation Failure. Endocrinology. 2021 Jul 1;162(7):bqab044. doi: 10.1210/endocr/bqab044. | Not relevant to the topic |
|  | Wu Y, Wang F, Wang T, Zheng Y, You L, Xue J. Association of Retinol-Binding Protein 4 with Arteriovenous Fistula Dysfunction in Hemodialysis Patients. Blood Purif. 2021;50(6):906-913. doi: 10.1159/000513418. | Not relevant to the topic |
|  | Tsuboi M, Suzuki H, Kawai H, Ejima T, Mitsuishi F. Vascular sound visualization system is useful for monitoring and surveillance of vascular access. J Vasc Access. 2022 May;23(3):390-397. doi: 10.1177/1129729821993984. | Not relevant to the topic |
|  | Zahra SA, Choudhury RY, Basharat K, Tran T, Begum M, Abotabekh A, Hedayat F, Rimmer L, Harky A, Bashir M. Translational Sciences in Cardiac Failure Secondary to Arteriovenous Fistula in Hemodialysis Patients. Ann Vasc Surg. 2021 Jul;74:431-449. doi: 10.1016/j.avsg.2021.01.071. | Not relevant to the topic |
|  | Karnabatidis D, Kitrou PM, Ponce P, Chong TT, Pietura R, Pegis JD, Ko PJ, Lin CH; Lutonix AV Global Registry Investigators. A Multicenter Global Registry of Paclitaxel Drug-Coated Balloon in Dysfunctional Arteriovenous Fistulae and Grafts: 6-Month Results. J Vasc Interv Radiol. 2021 Mar;32(3):360-368.e2. doi: 10.1016/j.jvir.2020.11.018. | Not relevant to the topic |
|  | Chan MR, Bedi S, Sanchez RJ, Young HN, Becker YT, Kellerman PS, Yevzlin AS. Stent placement versus angioplasty improves patency of arteriovenous grafts and blood flow of arteriovenous fistulae. Clin J Am Soc Nephrol. 2008 May;3(3):699-705. doi: 10.2215/CJN.04831107. | Conference abstracts,etc |
|  | Liang L, Chen Q, Cao W, Tang L, Wei Y, Ding N, Kong X, Xu D. Chitinase-3-like protein 1 is an independent risk factor for the early failure of forearm autologous arteriovenous fistulas in uremic patients. Ther Apher Dial. 2021 Dec;25(6):939-946. doi: 10.1111/1744-9987.13623. | Not relevant to the topic |
|  | MacDonald CJ, Ross R, Houston JG. Assessment of brachial artery mechanics using velocity vector imaging does not predict arteriovenous fistula failure; a feasibility study. Biomed Phys Eng Express. 2020 Apr 9;6(3):035022. doi: 10.1088/2057-1976/ab7549. | Not relevant to the topic |
|  | Gorsane I, Chermiti M, Mechri E, Elloumi Z. Evolutive profile of hemodialysis vascular accesses Hemodialysis accesses. Tunis Med. 2021 Mai;99(5):575-581. | Not relevant to the topic |
|  | Letachowicz K, Bardowska K, Królicki T, Kamińska D, Banasik M, Zajdel K, Mazanowska O, Madziarska K, Janczak D, Krajewska M. The impact of location and patency of the arteriovenous fistula on quality of life of kidney transplant recipients. Ren Fail. 2021 Dec;43(1):113-122. doi: 10.1080/0886022X.2020.1865171. | Not relevant to the topic |
|  | Yadav R, Gerrickens MWM, Teijink JAW, Scheltinga MRM. Abnormal preoperative digital brachial index is associated with lower 2-year arteriovenous fistula access patency. J Vasc Surg. 2021 Jul;74(1):237-245. doi: 10.1016/j.jvs.2020.12.075. | Not relevant to the topic |
|  | Dupuis MÈ, Laurin LP, Goupil R, Bénard V, Pichette M, Lafrance JP, Elftouh N, Pichette V, Nadeau-Fredette AC. Arteriovenous Fistula Creation and Estimated Glomerular Filtration Rate Decline in Advanced CKD: A Matched Cohort Study. Kidney360. 2020 Nov 19;2(1):42-49. doi: 10.34067/KID.0005072020. | Not relevant to the topic |
|  | Ticala M, Rusu CC, Moldovan D, Potra AR, Tirinescu DC, Coman AL, Bondor CI, Budisan L, Kacso IM. Relationship between vascular cell adhesion molecule-1 (VCAM-1), soluble receptor for advanced glycation end products (sRAGE) and functional hemodynamic parameters of arteriovenous fistula. J Vasc Access. 2022 Jan;23(1):67-74. doi: 10.1177/1129729820976264. | Not relevant to the topic |
|  | Abreo K, Sachdeva B, Abreo AP. To ligate or not to ligate hemodialysis arteriovenous fistulas in kidney transplant patients. J Vasc Access. 2021 Nov;22(6):942-946. doi: 10.1177/1129729820970786. | Not relevant to the topic |
|  | Aljarrah Q, Allouh M, Hallak AH, Alghezawi SE, Al-Omari M, Elheis M, Al-Jarrah M, Bakkar S, Aleshawi AJ, Al-Jarrah H, Ibrahim KS, Al Shishani JM, Almukhtar A. Lesion Type Analysis of Hemodialysis Patients Who Underwent Endovascular Management for Symptomatic Central Venous Disease. Vasc Health Risk Manag. 2020 Oct 9;16:419-427. doi: 10.2147/VHRM.S273450. | Not relevant to the topic |
|  | Stolić RV, Bukumiric Z, Mitrovic V, Sipic M, Krdzic B, Relic G, Nikolic G, Sovtic S, Suljkovic NE. Are There Differences in Arteriovenous Fistulae Created for Hemodialysis between Nephrologists and Vascular Surgeons? Med Princ Pract. 2021;30(2):178-184. doi: 10.1159/000512632. | Not relevant to the topic |
|  | He Y, Northrup H, Roy-Chaudhury P, Cheung AK, Berceli SA, Shiu YT. Analyses of hemodialysis arteriovenous fistula geometric configuration and its associations with maturation and reintervention. J Vasc Surg. 2021 May;73(5):1778-1786.e1. doi: 10.1016/j.jvs.2020.09.033. | Not relevant to the topic |
|  | Bodington R, Greenley S, Bhandari S. Getting the basics right: the monitoring of arteriovenous fistulae, a review of the evidence. Curr Opin Nephrol Hypertens. 2020 Nov;29(6):564-571. doi: 10.1097/MNH.0000000000000644. | Not relevant to the topic |
|  | See YP, Cho Y, Pascoe EM, Cass A, Irish A, Voss D, Polkinghorne KR, Hooi LS, Ong LM, Paul-Brent PA, Kerr PG, Mori TA, Hawley CM, Johnson DW, Viecelli AK. Predictors of Arteriovenous Fistula Failure: A *Post Hoc* Analysis of the FAVOURED Study. Kidney360. 2020 Sep 14;1(11):1259-1269. doi: 10.34067/KID.0002732020. | Not relevant to the topic |
|  | Paketci S. Exploring why female sex is a risk factor for arteriovenous fistula failure. Ther Apher Dial. 2021 Jun;25(3):352-353. doi: 10.1111/1744-9987.13582. | Conference abstracts,etc |
|  | Gumus F. Patency Rates After Successful Arteriovenous Fistula Thrombectomy: Relevance of the Flow/d-Dimer Ratio in the Decision-Making. Vasc Endovascular Surg. 2020 Nov;54(8):670-675. doi: 10.1177/1538574420945064. | Not relevant to the topic |
|  | Yap YS, Chi WC, Lin CH, Liu YC, Wu YW, Yang HY. Factors affecting patency of arteriovenous fistula following first percutaneous transluminal angioplasty. Clin Exp Nephrol. 2021 Jan;25(1):80-86. doi: 10.1007/s10157-020-01958-w. | Not relevant to the topic |
|  | Granata A, Maccarrone R, Di Lullo L, Morale W, Battaglia GG, Di Nicolò P, Bellasi A, Pesce F, Khater E, Gesualdo L, Basile A, Gallieni M. Feasibility of routine ultrasound-guided percutaneous transluminal angioplasty in the treatment of native arteriovenous fistula dysfunction. J Vasc Access. 2021 Sep;22(5):739-743. doi: 10.1177/1129729820943076. | Not relevant to the topic |
|  | Locham S, Naazie I, Canner J, Siracuse J, Al-Nouri O, Malas M. Incidence and risk factors of sepsis in hemodialysis patients in the United States. J Vasc Surg. 2021 Mar;73(3):1016-1021.e3. doi: 10.1016/j.jvs.2020.06.126. Epub 2020 Jul 21. | Not relevant to the topic |
|  | Chen MC, Weng MJ, Chang BC, Lai HC, Wu MY, Fu CY, Liu YC, Chi WC. Quantification of the severity of outflow stenosis of hemodialysis fistulas with a pulse- and thrill-based scoring system. BMC Nephrol. 2020 Jul 25;21(1):304. doi: 10.1186/s12882-020-01968-6. | Not relevant to the topic |
|  | Gomes AP, Germano A, Sousa M, Martins R, Coelho C, Ferreira MJ, Rocha E, Nunes V. Preoperative color Doppler ultrasound parameters for surgical decision-making in upper arm arteriovenous fistula maturation. J Vasc Surg. 2021 Mar;73(3):1022-1030. doi: 10.1016/j.jvs.2020.07.063. | Not relevant to the topic |
|  | Eroglu E, Kocyiğit I, Karakukcu C, Tuncay A, Zararsiz G, Eren D, Kahriman G, Hayri Sipahioglu M, Tokgoz B, Tasdemir K, Oymak O. Hypoxia-inducible factors in arteriovenous fistula maturation: A prospective cohort study. Eur J Clin Invest. 2020 Dec;50(12):e13350. doi: 10.1111/eci.13350. | Not relevant to the topic |
|  | Stoumpos S, Tan A, Hall Barrientos P, Stevenson K, Thomson PC, Kasthuri R, Radjenovic A, Kingsmore DB, Roditi G, Mark PB. Ferumoxytol MR Angiography versus Duplex US for Vascular Mapping before Arteriovenous Fistula Surgery for Hemodialysis. Radiology. 2020 Oct;297(1):214-222. doi: 10.1148/radiol.2020200069. | Not relevant to the topic |
|  | Bulbul E, Enc N. Construction and validation of a scale for assessing arteriovenous fistulas. J Vasc Access. 2021 Mar;22(2):178-183. doi: 10.1177/1129729820933753. | Not relevant to the topic |
|  | Cyrek AE, Bernheim J, Juntermanns B, Husen P, Pacha A, Hoffmann JN. Intraoperative use of transit time flow measurement improves patency of newly created radiocephalic arteriovenous fistulas in patients requiring hemodialysis. J Vasc Access. 2020 Nov;21(6):990-996. doi: 10.1177/1129729820916561. | Not relevant to the topic |
|  | Blanchard V, Courtellemont C, Cariou E, Fournier P, Lavie-Badie Y, Pascal P, Galinier M, Kamar N, Carrié D, Lairez O. Cardiac impact of arteriovenous fistulas: what tools to assess? Heart Vessels. 2020 Nov;35(11):1583-1593. doi: 10.1007/s00380-020-01630-z. | Not relevant to the topic |
|  | Obeidat KA, Saadeh RA, Hammouri HM, Obeidat MA, Tawalbeh RA. Outcomes of arteriovenous fistula creation: A Jordanian experience. J Vasc Access. 2020 Nov;21(6):977-982. doi: 10.1177/1129729820920140. | Not relevant to the topic |
|  | Hou G, Hou Y, Sun X, Yin N, Feng G, Yan Y, Guangyi L. Venous distensibility is more important than venous diameter in primary survival of autogenous radiocephalic arteriovenous fistulas. J Vasc Access. 2020 Nov;21(6):963-968. doi: 10.1177/1129729820920103. | Not relevant to the topic |
|  | Zonnebeld N, Tordoir JHM, van Loon MM, de Smet AAEA, Huisman LC, Cuypers PWM, Schlösser FJV, Lemson S, Heinen SGH, Bouwman LH, Toorop RJ, Huberts W, Delhaas T; Shunt Simulation Study Group. Pre-operative Patient Specific Flow Predictions to Improve Haemodialysis Arteriovenous Fistula Maturation (Shunt Simulation Study): A Randomised Controlled Trial. Eur J Vasc Endovasc Surg. 2020 Jul;60(1):98-106. doi: 10.1016/j.ejvs.2020.03.005. | Not relevant to the topic |
|  | Lopes JRA, Marques ALB, Correa JA. The influence of a doppler ultrasound in arteriovenous fistula for dialysis failure related to some risk factors. J Bras Nefrol. 2020 Apr 27;42(2):147-152. doi: 10.1590/2175-8239-JBN-2019-0080. | Not relevant to the topic |
|  | Waheed A, Masengu A, Skala T, Li G, Jastrzebski J, Zalunardo N. A prospective cohort study of predictors of upper extremity arteriovenous fistula maturation. J Vasc Access. 2020 Sep;21(5):746-752. doi: 10.1177/1129729820907471. | Not relevant to the topic |
|  | Png CYM, Korayem A, Finlay DJ. Post-General Anesthesia Ultrasound-Guided Venous Mapping Increases Autogenous Access Placement Rates. Ann Vasc Surg. 2018 Aug;51:132-140. doi: 10.1016/j.avsg.2018.02.015. | Not relevant to the topic |
|  | Xiong Y, Yu Y, Zhang C, Morris E, Wang L, Deng Y, Li Y, Fu P. The role of echocardiography in prognosis for dysfunction and abandonment of radiocephalic arteriovenous fistula in elderly Chinese patients on hemodialysis. Semin Dial. 2020 Jul;33(4):309-315. doi: 10.1111/sdi.12871. | Not relevant to the topic |
|  | Lee HS, Park MJ, Yoon SY, Joo N, Song YR, Kim HJ, Kim SG, Nizet V, Kim JK. Role of peribrachial fat as a key determinant of brachial artery dilatation for successful arteriovenous fistula maturation in hemodialysis patients. Sci Rep. 2020 Mar 2;10(1):3841. doi: 10.1038/s41598-020-60734-8. | Not relevant to the topic |
|  | Maresca B, Filice FB, Orlando S, Ciavarella GM, Scrivano J, Volpe M, Pirozzi N. Early echocardiographic modifications after flow reduction by proximal radial artery ligation in patients with high-output heart failure due to high-flow forearm arteriovenous fistula. J Vasc Access. 2020 Sep;21(5):753-759. doi: 10.1177/1129729820907249. | Not relevant to the topic |
|  | Veríssimo, R.; De Sousa, L. L.; Carvalho, T. J.; Mendes, A. P. Predictors of Vascular Access Thrombosis in Maintenance Hemodialysis Patients: An Historic Cohort Study: PO1036. Journal of the American Society of Nephrology 2021, 32 (10S), 347–347. https://doi.org/10.1681/ASN.20213210S1347b | Conference abstracts,etc |
|  | Cao Z, Li J, Zhang T, Zhao K, Zhao J, Yang Y, Jiang C, Zhu R, Li Z, Wu W. Comparative Effectiveness of Drug-Coated Balloon vs Balloon Angioplasty for the Treatment of Arteriovenous Fistula Stenosis: A Meta-analysis. J Endovasc Ther. 2020 Apr;27(2):266-275. doi: 10.1177/1526602820902757. | Not relevant to the topic |
|  | Drouven JW, de Bruin C, van Roon AM, Oldenziel J, Bokkers RPH, Zeebregts CJ. Outcomes after endovascular mechanical thrombectomy in occluded vascular access used for dialysis purposes. Catheter Cardiovasc Interv. 2020 Mar 1;95(4):758-764. doi: 10.1002/ccd.28730. | Not relevant to the topic |
|  | Lee TL, Chen CF, Tan AC, Chan CH, Ou SM, Chen FY, Yu KW, Chen YT, Lin CC. Prognosis of Vascular Access in Haemodialysis Patients with Autosomal Dominant Polycystic Kidney Disease. Sci Rep. 2020 Feb 6;10(1):1985. doi: 10.1038/s41598-020-58441-5. | Not relevant to the topic |
|  | Çakıcı EK, Çakıcı M, Gümüş F, Tan Kürklü TS, Yazılıtaş F, Örün UA, Bülbül M. Effects of hemodialysis access type on right heart geometry in adolescents. J Vasc Access. 2020 Sep;21(5):658-664. doi: 10.1177/1129729819897454. | Not relevant to the topic |
|  | Colucci M, Torreggiani M, Bernardi I, Stangalino S, Catucci D, Esposito V, Sileno G, Esposito C. Smart Flow for the evaluation of the hemodialysis arteriovenous fistula. J Vasc Access. 2020 Sep;21(5):652-657. doi: 10.1177/1129729819897171. | Not relevant to the topic |
|  | Drouven JW, de Bruin C, van Roon AM, Bokkers RPH, El Moumni M, Zeebregts CJ. Vascular access creation in octogenarians: The effect of age on outcomes. J Vasc Surg. 2020 Jul;72(1):171-179. doi: 10.1016/j.jvs.2019.09.047. | Not relevant to the topic |
|  | Colucci M, Torreggiani M, Bernardi I, Stangalino S, Catucci D, Esposito V, Sileno G, Esposito C. Smart Flow for the evaluation of the hemodialysis arteriovenous fistula. J Vasc Access. 2020 Sep;21(5):652-657. doi: 10.1177/1129729819897171 | Not relevant to the topic |
|  | Szarnecka-Sojda A, Jacheć W, Polewczyk M, Łętek A, Miszczuk J, Polewczyk A. Risk of Complications and Survival of Patients Dialyzed with Permanent Catheters. Medicina (Kaunas). 2019 Dec 19;56(1):2. doi: 10.3390/medicina56010002. | Not relevant to the topic |
|  | Kocaaslan C, Oztekin A, Bademci MS, Denli Yalvac ES, Bulut N, Aydin E. A retrospective comparison analysis of results of drug-coated balloon versus plain balloon angioplasty in treatment of juxta-anastomotic de novo stenosis of radiocephalic arteriovenous fistulas. J Vasc Access. 2020 Sep;21(5):596-601. doi: 10.1177/1129729819893205. | Not relevant to the topic |
|  | Mo YW, Song L, Huang JY, Sun CY, Zhou LF, Zheng SQ, Zhuang TT, Chen YG, Chen YH, Liu SX, Liang XL, Fu X. Can the fistula arm be used to lift heavy items? Six-pound dumbbells versus handgrip exercise in a 6-month follow-up secondary analysis of a randomized controlled trial. J Vasc Access. 2020 Sep;21(5):602-608. doi: 10.1177/1129729819894090. | Not relevant to the topic |
|  | Jaberi A, Schwartz D, Marticorena R, Dacouris N, Prabhudesai V, McFarlane P, Donnelly S. Risk factors for the development of cephalic arch stenosis. J Vasc Access. 2007 Oct-Dec;8(4):287-95. | Not relevant to the topic |
|  | Kong X, Tang L, Liang L, Cao W, Zhang L, Yong W, Ding N, Li W, Wang Z, Xu D. Clinical outcomes following the surgery of new autologous arteriovenous fistulas proximal to the failed ones in end-stage renal disease patients: a retrospective cohort study. Ren Fail. 2019 Nov;41(1):1036-1044. doi: 10.1080/0886022X.2019.1696210. | Not relevant to the topic |
|  | Gardezi AI, Karim MS, Rosenberg JE, Scialla JJ, Banerjee T, Powe NR, Shafi T, Parekh RS, Yevzlin AS, Astor BC. Markers of mineral metabolism and vascular access complications: The Choices for Healthy Outcomes in Caring for ESRD (CHOICE) study. Hemodial Int. 2020 Jan;24(1):43-51. doi: 10.1111/hdi.12798. | Not relevant to the topic |
|  | Stirbu O, Gadalean F, Pitea IV, Ciobanu G, Schiller A, Grosu I, Nes A, Bratescu R, Olariu N, Timar B, Tandrau MC. C-reactive protein as a prognostic risk factor for loss of arteriovenous fistula patency in hemodialyzed patients. J Vasc Surg. 2019 Jul;70(1):208-215. doi: 10.1016/j.jvs.2018.10.100. | Conference abstracts,etc |
|  | Onder AM, Flynn JT, Billings AA, Deng F, DeFreitas M, Katsoufis C, Grinsell MM, Patterson L, Jetton J, Fathallah-Shaykh S, Ranch D, Aviles D, Copelovitch L, Ellis E, Chadha V, Elmaghrabi A, Lin JJ, Butani L, Haddad M, Marsenic O, Brakeman P, Quigley R, Shin HS, Garro R, Liu H, Rahimikollu J, Raina R, Langman CB, Wood E; Midwest Pediatric Nephrology Consortium. Predictors of time to first cannulation for arteriovenous fistula in pediatric hemodialysis patients: Midwest Pediatric Nephrology Consortium study. Pediatr Nephrol. 2020 Feb;35(2):287-295. doi: 10.1007/s00467-019-04396-3. | Not relevant to the topic |
|  | Yuan Y, Cheng W, Lu H. Drug-Eluting Balloon Versus Plain Balloon Angioplasty For The Treatment of Failing Hemodialysis Access: A Systematic Review and Meta-analysis. Ann Vasc Surg. 2020 Apr;64:389-396. doi: 10.1016/j.avsg.2019.10.062. | Not relevant to the topic |
|  | Kong X, Du J, Su H, Chen Q, Tang L, Zhang L, Wang Z, Liang L, Xu D. Serum levels of the endothelial glycocalyx constituents and the early failure of forearm autologous arteriovenous fistulas in end-stage renal disease patients: a prospective cohort study. Int Urol Nephrol. 2020 Jan;52(1):169-177. doi: 10.1007/s11255-019-02317-9. | Not relevant to the topic |
|  | Duque JC, Martinez L, Tabbara M, Parikh P, Paez A, Selman G, Salman LH, Velazquez OC, Vazquez-Padron RI. Vascularization of the arteriovenous fistula wall and association with maturation outcomes. J Vasc Access. 2020 Mar;21(2):161-168. doi: 10.1177/1129729819863584. | Not relevant to the topic |
|  | Hou G, Yan Y, Li G, Hou Y, Sun X, Yin N, Feng G. Preoperative cephalic vein diameter and diabetes do not limit the choice of wrist radio-cephalic arteriovenous fistula. J Vasc Access. 2020 May;21(3):366-371. doi: 10.1177/1129729819879320. | Not relevant to the topic |
|  | Cheng HS, Chang TI, Chen CH, Hsu SC, Hsieh HL, Chen CY, Huang WC, Sue YM, Lin FY, Shih CM, Chen JW, Lin SJ, Huang PH, Liu CT. Study protocol for a prospective observational study to investigate the role of luminal pressure on arteriovenous fistula maturation. Medicine (Baltimore). 2019 Oct;98(40):e17238. doi: 10.1097/MD.0000000000017238. | Not relevant to the topic |
|  | Yu Y, Xiong Y, Zhang C, Fu M, Li Y, Fu P. Vascular Access Type Was Not Associated with Mortality and the Predictors for Cardiovascular Death in Elderly Chinese Patients on Hemodialysis. Blood Purif. 2020;49(1-2):63-70. doi: 10.1159/000502941. | Not relevant to the topic |
|  | Mallios, A.; Bourquelot, P.; Franco, G.; Hebibi, H.; Fonkoua, H.; Allouache, M.; Costanzo, A.; De Blic, R.; Harika, G.; Boura, B.; Jennings, W. C. Midterm Results of Percutaneous Arteriovenous Fistula Creation with the Ellipsys Vascular Access System, Technical Recommendations, and an Algorithm for Maintenance. J. Vasc. Surg. 2020, 72 (6), 2097–2106. https://doi.org/10.1016/j.jvs.2020.02.048. | Conference abstracts,etc |
|  | Hall D, Shaughnessy D, Drawz P, Akkina S, Esten A, Foley RN, Reule S. Time to thrombectomy is associated with increased risk for dialysis catheter placement. J Ren Care. 2019 Dec;45(4):232-238. doi: 10.1111/jorc.12295. | Not relevant to the topic |
|  | Yan Wee IJ, Yap HY, Hsien Ts'ung LT, Lee Qingwei S, Tan CS, Tang TY, Chong TT. A systematic review and meta-analysis of drug-coated balloon versus conventional balloon angioplasty for dialysis access stenosis. J Vasc Surg. 2019 Sep;70(3):970-979.e3. doi: 10.1016/j.jvs.2019.01.082. | Not relevant to the topic |
|  | Jorgensen MS, Farres H, James BLW, Li Z, Almerey T, Sheikh-Ali R, Clendenen S, Robards C, Erben Y, Oldenburg WA, Hakaim AG. The Role of Regional versus General Anesthesia on Arteriovenous Fistula and Graft Outcomes: A Single-Institution Experience and Literature Review. Ann Vasc Surg. 2020 Jan;62:287-294. doi: 10.1016/j.avsg.2019.05.016. | Not relevant to the topic |
|  | Szewczyk D, Bojakowski K, Kasprzak D, Kaźmierczak S, Piasecki A, Andziak P. Creation of Arteriovenous Fistulas and Grafts Concomitantly with Endovascular Correction of Outflow Veins: A Hybrid Procedure. Ann Vasc Surg. 2019 Nov;61:356-362. doi: 10.1016/j.avsg.2019.04.047. | Not relevant to the topic |
|  | Salem KM, Abou Ali AN, Sue E, Mohapatra A, Eid RE, Kormos RL, Chaer RA, Avgerinos ED. Maturation of arteriovenous fistulas in patients with ventricular assist devices. J Vasc Access. 2020 Mar;21(2):176-179. doi: 10.1177/1129729819865706. | Not relevant to the topic |
|  | Pike SL, Farber A, Arinze N, Levin S, Cheng TW, Jones DW, Tan TW, Malas M, Rybin D, Siracuse JJ. Patients with lower extremity dialysis access have poor primary patency and survival. J Vasc Surg. 2019 Dec;70(6):1913-1918. doi: 10.1016/j.jvs.2019.03.037. | Not relevant to the topic |
|  | Tozzi M, Franchin M, Savio D, Comelli S, Di Maggio L, Carbonari L, Ebrahimi R, Fontana F, Piacentino F, Cervarolo MC, Palermo V, Piffaretti G. Drug-coated balloon angioplasty in failing haemodialysis arteriovenous shunts: 12-month outcomes in 200 patients from the Aperto Italian registry. J Vasc Access. 2019 Nov;20(6):733-739. doi: 10.1177/1129729819848609. | Not relevant to the topic |
|  | Copeland T, Lawrence P, Woo K. Outcomes of initial hemodialysis vascular access in patients initiating dialysis with a tunneled catheter. J Vasc Surg. 2019 Oct;70(4):1235-1241. doi: 10.1016/j.jvs.2019.02.036. | Not relevant to the topic |
|  | So YH, Choi YH, Oh S, Jung IM, Chung JK, Lucatelli P. Thrombosed native hemodialysis fistulas: Technical and clinical outcomes of endovascular recanalization and factors influencing patency. J Vasc Access. 2019 Nov;20(6):725-732. doi: 10.1177/1129729819848931. | Not relevant to the topic |
|  | Manou-Stathopoulou S, Robinson EJ, Harvey JJ, Karunanithy N, Calder F, Robson MG. Factors associated with outcome after successful radiological intervention in arteriovenous fistulas: A retrospective cohort. J Vasc Access. 2019 Nov;20(6):716-724. doi: 10.1177/1129729819845991. | Not relevant to the topic |
|  | Wang S, Wang MS. Intraoperative pneumatic tourniquet use may improve the clinical outcomes of arteriovenous fistula creations. J Vasc Access. 2019 Nov;20(6):706-715. doi: 10.1177/1129729819845968. | Not relevant to the topic |
|  | Alsheekh A, Hingorani A, Aurshina A, Kibrik P, Chait J, Ascher E. Early Results of Duplex-Guided Transradial Artery Fistuloplasties. Ann Vasc Surg. 2019 Oct;60:178-181. doi: 10.1016/j.avsg.2019.02.036. | Not relevant to the topic |
|  | Siga EL, Ibalo N, Benegas MR, Laura F, Luna C, Aiziczon DH, Demicheli E. Relevance of a skilled vascular surgeon and optimized facility practices in the long-term patency of arteriovenous fistulas: a prospective study. J Bras Nefrol. 2019 Jul-Sep;41(3):330-335. doi: 10.1590/2175-8239-JBN-2018-0125. | Not relevant to the topic |
|  | Borzych-Duzalka D, Shroff R, Ariceta G, Yap YC, Paglialonga F, Xu H, Kang HG, Thumfart J, Aysun KB, Stefanidis CJ, Fila M, Sever L, Vondrak K, Szabo AJ, Szczepanska M, Ranchin B, Holtta T, Zaloszyc A, Bilge I, Warady BA, Schaefer F, Schmitt CP. Vascular Access Choice, Complications, and Outcomes in Children on Maintenance Hemodialysis: Findings From the International Pediatric Hemodialysis Network (IPHN) Registry. Am J Kidney Dis. 2019 Aug;74(2):193-202. doi: 10.1053/j.ajkd.2019.02.014. | Inconsistent with study population |
|  | Lee ES, Shen Q, Pitts RL, Guo M, Wu MH, Sun SC, Yuan SY. Serum metalloproteinases MMP-2, MMP-9, and metalloproteinase tissue inhibitors in patients are associated with arteriovenous fistula maturation. J Vasc Surg. 2011 Aug;54(2):454-9; discussion 459-60. doi: 10.1016/j.jvs.2011.02.056. | Conference abstracts,etc |
|  | Thomas SD, Peden S, Crowe P, Varcoe RL. Interwoven Nitinol Stents to Treat Radiocephalic Anastomotic Arteriovenous Fistula Stenosis. J Endovasc Ther. 2019 Jun;26(3):394-401. doi: 10.1177/1526602819842851. | Not relevant to the topic |
|  | Gulcu A, Sarioglu O, Peker A, Alatas O. Drug-Eluting Balloon Angioplasty for Juxta-Anastomotic Stenoses in Distal Radiocephalic Hemodialysis Fistulas: Long-Term Patency Results. Cardiovasc Intervent Radiol. 2019 Jun;42(6):835-840. doi: 10.1007/s00270-019-02213-w. | Not relevant to the topic |
|  | Cho M, Kim JS, Cho S, Cho WP, Choi C, Ahn S, Min SI, Ha J, Min SK. Baseline characteristics of arm vessels by preoperative duplex ultrasonography in Korean patients for hemodialysis vascular access. J Vasc Access. 2019 Nov;20(6):646-651. doi: 10.1177/1129729819838168. | Not relevant to the topic |
|  | Kennedy SA, Mafeld S, Baerlocher MO, Jaberi A, Rajan DK. Drug-Coated Balloon Angioplasty in Hemodialysis Circuits: A Systematic Review and Meta-Analysis. J Vasc Interv Radiol. 2019 Apr;30(4):483-494.e1. doi: 10.1016/j.jvir.2019.01.012. | Not relevant to the topic |
|  | Fumagalli G, Trovato F, Migliori M, Panichi V, De Pietro S. The forearm arteriovenous graft between the brachial artery and the brachial vein as a reliable dialysis vascular access for patients with inadequate superficial veins. J Vasc Surg. 2019 Jul;70(1):199-207.e4. doi: 10.1016/j.jvs.2018.10.101. | Not relevant to the topic |
|  | Hicks CW, Bae S, Pozo ME, DiBrito SR, Abularrage CJ, Segev DL, Garonzik-Wang J, Reifsnyder T. Practice patterns in arteriovenous fistula ligation among kidney transplant recipients in the United States Renal Data Systems. J Vasc Surg. 2019 Sep;70(3):842-852.e1. doi: 10.1016/j.jvs.2018.11.048. | Not relevant to the topic |
|  | Dua A, Rothenberg KA, Mikkineni K, Sgroi MD, Sorial E, Toca MG. Secondary interventions in patients with implantable cardiac devices and ipsilateral arteriovenous access. J Vasc Surg. 2019 Oct;70(4):1242-1246. doi: 10.1016/j.jvs.2018.12.029. | Not relevant to the topic |
|  | Wan Z, Lai Q, Zhou Y, Chen L, Tu B. Partial aneurysmectomy for treatment of autologous hemodialysis fistula aneurysm is safe and effective. J Vasc Surg. 2019 Aug;70(2):547-553. doi: 10.1016/j.jvs.2018.10.119. | Not relevant to the topic |
|  | Martinez L, Tabbara M, Duque JC, Selman G, Falcon NS, Paez A, Griswold AJ, Ramos-Echazabal G, Hernandez DR, Velazquez OC, Salman LH, Vazquez-Padron RI. Transcriptomics of Human Arteriovenous Fistula Failure: Genes Associated With Nonmaturation. Am J Kidney Dis. 2019 Jul;74(1):73-81. doi: 10.1053/j.ajkd.2018.12.035. | Not relevant to the topic |
|  | Beaulieu RJ, Locham S, Nejim B, Dakour-Aridi H, Woo K, Malas MB. General anesthesia is associated with reduced early failure among patients undergoing hemodialysis access. J Vasc Surg. 2019 Mar;69(3):890-897.e5. doi: 10.1016/j.jvs.2018.05.247. | Not relevant to the topic |
|  | Prasad N, Thammishetti V, Bhadauria DS, Kaul A, Sharma RK, Srivastava A, Gupta A. Outcomes of radiocephalic fistula created by nephrologists. J Vasc Access. 2019 Nov;20(6):615-620. doi: 10.1177/1129729819830361. | Not relevant to the topic |
|  | Shiu YT, Rotmans JI, Geelhoed WJ, Pike DB, Lee T. Arteriovenous conduits for hemodialysis: how to better modulate the pathophysiological vascular response to optimize vascular access durability. Am J Physiol Renal Physiol. 2019 May 1;316(5):F794-F806. doi: 10.1152/ajprenal.00440.2018. | Not relevant to the topic |
|  | Yildiz I. The Efficacy of Paclitaxel Drug-Eluting Balloon Angioplasty Versus Standard Balloon Angioplasty in Stenosis of Native Hemodialysis Arteriovenous Fistulas: An Analysis of Clinical Success, Primary Patency and Risk Factors for Recurrent Dysfunction. Cardiovasc Intervent Radiol. 2019 May;42(5):685-692. doi: 10.1007/s00270-019-02171-3. | Not relevant to the topic |
|  | Okamuro L, Gray K, Korn A, Parrish A, Kaji A, Howell EC, Bowens N, de Virgilio C. Careful Patient Selection Achieves High Radiocephalic Arteriovenous Fistula Patency in Diabetic and Female Patients. Ann Vasc Surg. 2019 May;57:16-21. doi: 10.1016/j.avsg.2018.12.057. | Not relevant to the topic |
|  | Lin YC, Huang YY, Lin MY, Chiu YW, Lim LM, Hsieh CC. Long-term outcomes of lateral tunneled transposed brachiobasilic arteriovenous fistulas in elderly hemodialysis patient. J Vasc Access. 2019 Sep;20(5):557-562. doi: 10.1177/1129729818820184. | Not relevant to the topic |
|  | Rezapour M, Sepehri MM, Khavanin Zadeh M, Alborzi M. A new method to determine anastomosis angle configuration for arteriovenous fistula maturation. Med J Islam Repub Iran. 2018 Jul 25;32:62. doi: 10.14196/mjiri.32.62. | Not relevant to the topic |
|  | Bruns SD, Jennings WC. Proximal radial artery as inflow site for native arteriovenous fistula. J Am Coll Surg. 2003 Jul;197(1):58-63. doi: 10.1016/S1072-7515(03)00142-X. | Conference abstracts,etc |
|  | Guo-Cun H, Yong-Hong Y, Xiu-Li S, Yi H, Na Y, Guo-Zhen F, Ai-Zhen C. Two weeks post-operative ultrasound examination of radio-cephalic arteriovenous fistulae to predict maturity in a Chinese population. J Vasc Access. 2019 Jul;20(4):417-422. doi: 10.1177/1129729818821620. | Not relevant to the topic |
|  | Tan RY, Pang SC, Teh SP, Lee KG, Chong TT, Gogna A, Tan CS. Comparison of alteplase and urokinase for pharmacomechanical thrombolysis of clotted hemodialysis access. J Vasc Access. 2019 Sep;20(5):501-506. doi: 10.1177/1129729818819735. | Not relevant to the topic |
|  | Kim JH, Cho SB, Kim YH, Chung HH, Lee SH, Sung DJ. Transjugular percutaneous endovascular treatment of dysfunctional hemodialysis access. J Vasc Access. 2019 Sep;20(5):488-494. doi: 10.1177/1129729818815327. | Not relevant to the topic |
|  | Hwang D, Park S, Kim HK, Huh S. Comparative outcomes of vascular access in patients older than 70 years with end-stage renal disease. J Vasc Surg. 2019 Apr;69(4):1196-1206.e5. doi: 10.1016/j.jvs.2018.07.061. | Not relevant to the topic |
|  | D'cruz RT, Leong SW, Syn N, Tiwari A, Sannasi VV, Singh Sidhu HR, Tang TY. Endovascular treatment of cephalic arch stenosis in brachiocephalic arteriovenous fistulas: A systematic review and meta-analysis. J Vasc Access. 2019 Jul;20(4):345-355. doi: 10.1177/1129729818814466. | Not relevant to the topic |
|  | Kumbar L, Peterson E, Zaborowicz M, Besarab A, Yee J, Zasuwa G. Sentinel vascular access monitoring after endovascular intervention predicts access outcome. J Vasc Access. 2019 Jul;20(4):409-416. doi: 10.1177/1129729818812729. | Not relevant to the topic |
|  | Saeed F, Arrigain S, Schold JD, Nally JV Jr, Navaneethan SD. What are the Risk Factors for One-Year Mortality in Older Patients with Chronic Kidney Disease? An Analysis of the Cleveland Clinic CKD Registry. Nephron. 2019;141(2):98-104. doi: 10.1159/000494298. | Not relevant to the topic |
|  | Jamil M, Usman R, Ali K. Proximal Arterio Venous Fistula Creation - A Gold Standard In Chronic Renal Failure Patients With Multiple Comorbidities. J Ayub Med Coll Abbottabad. 2018 Jul-Sep;30(3):439-442. | Not relevant to the topic |
|  | Ozpak B, Yilmaz Y. Arteriovenous fistulas ipsilateral to internal jugular catheters for hemodialysis have decreased patency rates. Vascular. 2019 Jun;27(3):270-276. doi: 10.1177/1708538118811483. | Not relevant to the topic |
|  | Zhou M, Lu FP. [Effect of hyperphosphatemia on patency rate of arteriovenous fistula of patients with late fistula dysfunction/failure after reoperation]. Zhonghua Yi Xue Za Zhi. 2018 Nov 13;98(42):3406-3410. Chinese. doi: 10.3760/cma.j.issn.0376-2491. | Not relevant to the topic |
|  | Lyu B, Banerjee T, Scialla JJ, Shafi T, Yevzlin AS, Powe NR, Parekh RS, Astor BC. Vascular Calcification Markers and Hemodialysis Vascular Access Complications. Am J Nephrol. 2018;48(5):330-338. doi: 10.1159/000493549. | Not relevant to the topic |
|  | Majerus SJA, Knauss T, Mandal S, Vince G, Damaser MS. Bruit-enhancing phonoangiogram filter using sub-band autoregressive linear predictive coding. Annu Int Conf IEEE Eng Med Biol Soc. 2018 Jul;2018:1416-1419. doi: 10.1109/EMBC.2018.8512588. | Not relevant to the topic |
|  | Choi YJ, Lee YK, Park HC, Kim EY, Cho A, Han C, Choi SR, Kim H, Kim EJ, Yoon JW, Noh JW. Prediction of vascular access stenosis: Blood temperature monitoring with the Twister versus static intra-access pressure ratio. PLoS One. 2018 Oct 29;13(10):e0204630. doi: 10.1371/journal.pone.0204630. | Not relevant to the topic |
|  | Samarendra P, Ramkumar M, Sharma V, Kumari S. Cardiorenal syndrome in renal transplant recipients - It's the fistula at fault: A case series. Clin Transplant. 2018 Nov;32(11):e13417. doi: 10.1111/ctr.13417. | Not relevant to the topic |
|  | obbin ML, Greene T, Allon M, Dember LM, Imrey PB, Cheung AK, Himmelfarb J, Huber TS, Kaufman JS, Radeva MK, Roy-Chaudhury P, Shiu YT, Vazquez MA, Umphrey HR, Alexander L, Abts C, Beck GJ, Kusek JW, Feldman HI; Hemodialysis Fistula Maturation Study Group. Prediction of Arteriovenous Fistula Clinical Maturation from Postoperative Ultrasound Measurements: Findings from the Hemodialysis Fistula Maturation Study. J Am Soc Nephrol. 2018 Nov;29(11):2735-2744. doi: 10.1681/ASN.2017111225. Epub 2018 Oct 11. Erratum in: J Am Soc Nephrol. 2020 Mar;31(3):665. doi: 10.1681/ASN.2020010067. | Not relevant to the topic |
|  | Hsu YH, Yen YC, Lin YC, Sung LC. Antiplatelet agents maintain arteriovenous fistula and graft function in patients receiving hemodialysis: A nationwide case-control study. PLoS One. 2018 Oct 18;13(10):e0206011. doi: 10.1371/journal.pone.0206011. Erratum in: PLoS One. 2019 Apr 17;14(4):e0215546. doi: 10.1371/journal.pone.0215546. | Not relevant to the topic |
|  | Higashiura W, Takara H, Kitamura R, Yasutani T, Miyasato H, Tengan T. Factors Associated with Secondary Functional Patency After Percutaneous Transluminal Angioplasty of the Early Failing or Immature Hemodialysis Arteriovenous Fistula. Cardiovasc Intervent Radiol. 2019 Jan;42(1):34-40. doi: 10.1007/s00270-018-2083-0. | Not relevant to the topic |
|  | Onder AM, Flynn JT, Billings AA, Deng F, DeFreitas M, Katsoufis C, Grinsell MM, Patterson LT, Jetton J, Fathallah-Shaykh S, Ranch D, Aviles D, Copelovitch L, Ellis E, Chanda V, Elmaghrabi A, Lin JJ, Butani L, Haddad M, Couloures OM, Brakeman P, Quigley R, Stella Shin H, Garro R, Liu H, Rahimikollu J, Raina R, Langman CB, Wood EG; Midwest Pediatric Nephrology Consortium. Predictors of patency for arteriovenous fistulae and grafts in pediatric hemodialysis patients. Pediatr Nephrol. 2019 Feb;34(2):329-339. doi: 10.1007/s00467-018-4082-4. | Not relevant to the topic |
|  | Patanè D, Failla G, Coniglio G, Russo G, Morale W, Seminara G, Calcara G, Bisceglie P, Malfa P. Treatment of juxta-anastomotic stenoses for failing distal radiocephalic arteriovenous fistulas: Drug-coated balloons versus angioplasty. J Vasc Access. 2019 Mar;20(2):209-216. doi: 10.1177/1129729818793102. | Not relevant to the topic |
|  | Farrington CA, Robbin ML, Lee T, Barker-Finkel J, Allon M. Postoperative Ultrasound, Unassisted Maturation, and Subsequent Primary Patency of Arteriovenous Fistulas. Clin J Am Soc Nephrol. 2018 Sep 7;13(9):1364-1372. doi: 10.2215/CJN.02230218. | Not relevant to the topic |
|  | Rodrigues AT, Colugnati FAB, Bastos MG. Evaluation of variables associated with the patency of arteriovenous fistulas for hemodialysis created by a nephrologist. J Bras Nefrol. 2018 Oct-Dec;40(4):326-332. doi: 10.1590/2175-8239-jbn-2017-0014. | Not relevant to the topic |
|  | Hallioglu O, Keceli M, Bozlu G, Delibas A, Karpuz D, Selvi H. Evaluation of T-wave alternans in pediatric patients with chronic renal failure. J Electrocardiol. 2018 Jul-Aug;51(4):622-627. doi: 10.1016/j.jelectrocard.2018.04.013. | Not relevant to the topic |
|  | Hwang SD, Lee JH, Lee SW, Kim JK, Kim MJ, Song JH. Comparison of ultrasound scan blood flow measurement versus other forms of surveillance in the thrombosis rate of hemodialysis access: A systemic review and meta-analysis. Medicine (Baltimore). 2018 Jul;97(30):e11194. doi: 10.1097/MD.0000000000011194. | Not relevant to the topic |
|  | Zouaghi MK, Lammouchi MA, Hassan M, Rais L, Krid M, Smaoui W, Jebali H, Kheder R, Hamida FB, Moussa FB, Fatma LB, Beji S. Determinants of patency of arteriovenous fistula in hemodialysis patients. Saudi J Kidney Dis Transpl. 2018 May-Jun;29(3):615-622. doi: 10.4103/1319-2442.235183. | Not relevant to the topic |
|  | Yan Y, Su X, Zheng J, Zhang L, Yang L, Jiang Q, Chen Q. Association of Preoperative Mean Arterial Pressure With the Primary Failure of Brescia-Cimino Arteriovenous Fistula Within the First 7 Days Following Surgery in Hemodialysis Patients. Ther Apher Dial. 2018 Oct;22(5):539-543. doi: 10.1111/1744-9987. | Not relevant to the topic |
|  | Jun Yan Wee I, Mohamed IH, Patel A, Choong AMTL. A systematic review and meta-analysis of one-stage versus two-stage brachiobasilic arteriovenous fistula creation. J Vasc Surg. 2018 Jul;68(1):285-297. doi: 10.1016/j.jvs.2018.03.428. | Not relevant to the topic |
|  | Wärme A, Hadimeri U, Hadimeri H, Nasic S, Stegmayr B. High doses of erythropoietin stimulating agents may be a risk factor for AV-fistula stenosis. Clin Hemorheol Microcirc. 2019;71(1):53-57. doi: 10.3233/CH-180381. | Not relevant to the topic |
|  | Fraser CD 3rd, Grimm JC, Liu RH, Wesson RN, Azar F, Beaulieu RJ, Reifsnyder T. Removal of Noninfected Arteriovenous Fistulae after Kidney Transplantation is a Safe and Beneficial Management Strategy for Unused Dialysis Access. Ann Vasc Surg. 2018 Nov;53:128-132. doi: 10.1016/j.avsg.2018.04.020. | Not relevant to the topic |
|  | Said K, Hassan M, Farouk M, Baligh E, Zayed B. Right Ventricular Function After Creation of an Atriovenous Fistula in Patients With End Stage Renal Disease. Heart Lung Circ. 2019 Jun;28(6):884-892. doi: 10.1016/j.hlc.2018.04.282. | Not relevant to the topic |
|  | Spatola L, Finazzi S, Calvetta A, Angelini C, Badalamenti S. Subjective Global Assessment-Dialysis Malnutrition Score and arteriovenous fistula outcome: A comparison with Charlson Comorbidity Index. J Vasc Access. 2019 Jan;20(1):70-78. doi: 10.1177/1129729818779550. | Not relevant to the topic |
|  | Marcelin C, D'Souza S, Le Bras Y, Petitpierre F, Grenier N, van den Berg JC, Huasen B. Mechanical Thrombectomy in Acute Thrombosis of Dialysis Arteriovenous Fistulae and Grafts Using a Vacuum-Assisted Thrombectomy Catheter: A Multicenter Study. J Vasc Interv Radiol. 2018 Jul;29(7):993-997. doi: 10.1016/j.jvir.2018.02.030. Epub 2018 May 30. Erratum in: J Vasc Interv Radiol. 2018 Nov;29(11):1637. doi: 10.1016/j.jvir.2018.10.001. | Not relevant to the topic |
|  | Tan RY, Manning M, Spurway J, Jegatheesan T, Bertram M, Phipps L, Swinnen J. Improving haemodialysis fistula maturation following early ultrasound vascular mapping: 'The Venous Preservation Scan'. Nephrology (Carlton). 2019 May;24(5):550-556. doi: 10.1111/nep.13403. | Not relevant to the topic |
|  | Conkbayir I, Celtikci P, Ergun O, Durmaz HA. Value of duplex Doppler ultrasonography for the evaluation of dysfunctional hemodialysis access arteriovenous fistulas prior to endovascular interventions. J Clin Ultrasound. 2018 Oct;46(8):503-511. doi: 10.1002/jcu.22601. | Not relevant to the topic |
|  | Yan Y, Ye D, Yang L, Ye W, Zhan D, Zhang L, Xiao J, Zeng Y, Chen Q. A meta-analysis of the association between diabetic patients and AVF failure in dialysis. Ren Fail. 2018 Nov;40(1):379-383. doi: 10.1080/0886022X.2018. | Not relevant to the topic |
|  | Thamer M, Lee TC, Wasse H, Glickman MH, Qian J, Gottlieb D, Toner S, Pflederer TA. Medicare Costs Associated With Arteriovenous Fistulas Among US Hemodialysis Patients. Am J Kidney Dis. 2018 Jul;72(1):10-18. doi: 10.1053/j.ajkd.2018.01.034. | Not relevant to the topic |
|  | Okawa T, Murakami M, Yamada R, Tanaka S, Mori K, Mori N. One-stage operation for superficialization of native radio-cephalic fistula in obese patients. J Vasc Access. 2019 May;20(1_suppl):45-49. doi: 10.1177/1129729818762994. | Not relevant to the topic |
|  | Wakamoto K, Doi S, Nakashima A, Kawai T, Kyuden Y, Naito T, Asai M, Takahashi S, Murakami M, Masaki T. Comparing the 12-month patency of low- versus high-pressure dilation in failing arteriovenous fistulae: A prospective multicenter trial (YOROI study). J Vasc Access. 2018 Sep;19(5):477-483. doi: 10.1177/1129729818760976. | Not relevant to the topic |
|  | Kusztal M, Nowak K. Cardiac implantable electronic device and vascular access: Strategies to overcome problems. J Vasc Access. 2018 Nov;19(6):521-527. doi: 10.1177/1129729818762981. | Not relevant to the topic |
|  | Abreo K, Buffington M, Sachdeva B. Angioplasty to promote arteriovenous fistula maturation and maintenance. J Vasc Access. 2018 Jul;19(4):337-340. doi: 10.1177/1129729818760979. | Not relevant to the topic |
|  | Roca-Tey R, Ibeas J, Moreno T, Gruss E, Merino JL, Vallespín J, Hernán D, Arribas P; Spanish Multidisciplinary Vascular Access Group (GEMAV). Dialysis arteriovenous access monitoring and surveillance according to the 2017 Spanish Guidelines. J Vasc Access. 2018 Sep;19(5):422-429. doi: 10.1177/1129729818761307. | Not relevant to the topic |
|  | Ghaffarian AA, Al-Dulaimi R, Kraiss LW, Sarfati M, Griffin CL, Smith BK, Donald G, Brooke BS. Clinical effectiveness of open thrombectomy for thrombosed autogenous arteriovenous fistulas and grafts. J Vasc Surg. 2018 Jul;68(1):189-196. doi: 10.1016/j.jvs.2017.12.050. | Not relevant to the topic |
|  | Pillado E, Behdad M, Williams R, Wilson SE. Flow Rates at Thirty Days after Construction of Radiocephalic Arteriovenous Fistula Predict Hemodialysis Function. Ann Vasc Surg. 2018 May;49:268-272. doi: 10.1016/j.avsg.2018.01.068. | Not relevant to the topic |
|  | Korn A, Alipour H, Zane J, Shahverdiani A, Ryan TJ, Kaji A, Bowens N, de Virgilio C. Factors Associated with Early Thrombosis after Arteriovenous Fistula Creation. Ann Vasc Surg. 2018 May;49:281-284. doi: 10.1016/j.avsg.2018.02.003. | Not relevant to the topic |
|  | Gray K, Korn A, Zane J, Gonzalez G, Kaji A, Bowens N, de Virgilio C. Ultrasound Vein and Artery Mapping by General Surgery Residents During Initial Consult Can Decrease Time to Dialysis Access Creation. Ann Vasc Surg. 2018 May;49:285-288. doi: 10.1016/j.avsg.2018.02.006. | Conference abstracts,etc |
|  | Zamboli P, Lucà S, Borrelli S, Garofalo C, Liberti ME, Pacilio M, Lucà S, Palladino G, Punzi M. High-flow arteriovenous fistula and heart failure: could the indexation of blood flow rate and echocardiography have a role in the identification of patients at higher risk? J Nephrol. 2018 Dec;31(6):975-983. doi: 10.1007/s40620-018-0472-8. | Not relevant to the topic |
|  | Martinez L, Duque JC, Tabbara M, Paez A, Selman G, Hernandez DR, Sundberg CA, Tey JCS, Shiu YT, Cheung AK, Allon M, Velazquez OC, Salman LH, Vazquez-Padron RI. Fibrotic Venous Remodeling and Nonmaturation of Arteriovenous Fistulas. J Am Soc Nephrol. 2018 Mar;29(3):1030-1040. doi: 10.1681/ASN.2017050559. | Not relevant to the topic |
|  | Aala A, Sharif S, Parikh L, Gordon PC, Hu SL. High-Output Cardiac Failure and Coronary Steal With an Arteriovenous Fistula. Am J Kidney Dis. 2018 Jun;71(6):896-903. doi: 10.1053/j.ajkd.2017.10.012. | Conference abstracts,etc |
|  | Suemitsu K, Shiraki T, Iida O, Kobayashi H, Matsuoka Y, Izumi M, Nakanishi T. Impact of Lesion Morphology on Durability After Angioplasty of Failed Arteriovenous Fistulas in Hemodialysis Patients. J Endovasc Ther. 2018 Oct;25(5):649-654. doi: 10.1177/1526602817748316. | Not relevant to the topic |
|  | Sun X, He Y, Ma Y, He H, Zhang L, de Seabra Rodrigues Dias IR, Liu Y. A Single-Center Retrospective Analysis of the Efficacy of a New Balloon Catheter in Autogenous Arteriovenous Fistula Dysfunction Resistant to Conventional Balloon Angioplasty. Ann Vasc Surg. 2018 Apr;48:79-88. doi: 10.1016/j.avsg.2017.09.025. | Not relevant to the topic |
|  | Karava V, Jehanno P, Kwon T, Deschênes G, Macher MA, Bourquelot P. Autologous arteriovenous fistulas for hemodialysis using microsurgery techniques in children weighing less than 20 kg. Pediatr Nephrol. 2018 May;33(5):855-862. doi: 10.1007/s00467-017-3854-6. | Inconsistent with study population |
|  | Glass C, Porter J, Singh M, Gillespie D, Young K, Illig K. A large-scale study of the upper arm basilic transposition for hemodialysis. Ann Vasc Surg. 2010 Jan;24(1):85-91. doi: 10.1016/j.avsg.2009.05.006. | Conference abstracts,etc |
|  | Viecelli AK, Mori TA, Roy-Chaudhury P, Polkinghorne KR, Hawley CM, Johnson DW, Pascoe EM, Irish AB. The pathogenesis of hemodialysis vascular access failure and systemic therapies for its prevention: Optimism unfulfilled. Semin Dial. 2018 May;31(3):244-257. doi: 10.1111/sdi.12658. | Not relevant to the topic |
|  | Troisi N, Frosini P, Somma C, Romano E, Guidotti A, Dattolo PC, Ferro G, Chisci E, Michelagnoli S. Drug-coated balloons reduce the risk of recurrent restenosis in arteriovenous fistulas and prosthetic grafts for hemodialysis. Int Angiol. 2018 Feb;37(1):59-63. doi: 10.23736/S0392-9590.17.03886-X. | Not relevant to the topic |
|  | McGrogan DG, Stringer S, Cockwell P, Jesky M, Ferro C, Maxwell AP, Inston NG. Arterial stiffness alone does not explain arteriovenous fistula outcomes. J Vasc Access. 2018 Jan;19(1):63-68. doi: 10.5301/jva.5000791. | Not relevant to the topic |
|  | Hsieh MY, Lin L, Chen TY, Chen DM, Lee MH, Shen YF, Yang CW, Chuang SY, Wu CC, Hung KY. Timely thrombectomy can improve patency of hemodialysis arteriovenous fistulas. J Vasc Surg. 2018 Apr;67(4):1217-1226. doi: 10.1016/j.jvs.2017.08.072. | Not relevant to the topic |
|  | Alfano G, Fontana F, Iannaccone M, Noussan P, Cappelli G. Preoperative management of arteriovenous fistula (AVF) for hemodialysis. J Vasc Access. 2017 Nov 17;18(6):451-463. doi: 10.5301/jva.5000771. | Not relevant to the topic |
|  | Basile C, Lomonte C. The complex relationship among arteriovenous access, heart, and circulation. Semin Dial. 2018 Jan;31(1):15-20. doi: 10.1111/sdi.12652. | Not relevant to the topic |
|  | Chaudry MS, Carlson N, Gislason GH, Kamper AL, Rix M, Fowler VG Jr, Torp-Pedersen C, Bruun NE. Risk of Infective Endocarditis in Patients with End Stage Renal Disease. Clin J Am Soc Nephrol. 2017 Nov 7;12(11):1814-1822. doi: 10.2215/CJN.02320317. | Not relevant to the topic |
|  | Ghaffarian AA, Griffin CL, Kraiss LW, Sarfati MR, Brooke BS. Comparative effectiveness of one-stage versus two-stage basilic vein transposition arteriovenous fistulas. J Vasc Surg. 2018 Feb;67(2):529-535.e1. doi: 10.1016/j.jvs.2017.07.115. | Not relevant to the topic |
|  | Murea M, Brown WM, Divers J, Moossavi S, Robinson TW, Bagwell B, Burkart JM, Freedman BI. Vascular Access Placement Order and Outcomes in Hemodialysis Patients: A Longitudinal Study. Am J Nephrol. 2017;46(4):268-275. doi: 10.1159/000481313. | Not relevant to the topic |
|  | Kim SM, Ko HK, Noh M, Ko GY, Kim MJ, Kwon TW, Kim HJ, Cho YP. Factors Affecting Patency following Successful Percutaneous Intervention for Dysfunctional Hemodialysis Vascular Access. Ann Vasc Surg. 2018 Feb;47:54-61. doi: 10.1016/j.avsg.2017.08.003. | Not relevant to the topic |
|  | Hicks CW, Bronsert M, Hammermeister KE, Henderson WG, Gibula DR, Black JH 3rd, Glebova NO. Temporal Trends, Determinants, and Outcomes of Inpatient versus Outpatient Arteriovenous Fistula Operations. Ann Vasc Surg. 2018 Jan;46:65-74.e1. doi: 10.1016/j.avsg.2017.07.032. | Not relevant to the topic |
|  | Tirinescu DC, Tomuleasa C, Pop L, Bondor CI, Vlăduţiu DŞ, Paţiu IM, Rusu CC, Moldovan DT, Potra A, Kacsó IM. Matrix-Metalloproteinase-2 Predicts Arteriovenous Fistula Failure in Hemodialysis Patients. Ther Apher Dial. 2017 Dec;21(6):586-591. doi: 10.1111/1744-9987.12584 | Not relevant to the topic |
|  | Ghahremani-Ghajar M, Jin A, Borghei P, Chen JLT. An unusual case of an irretrievable hemodialysis catheter in a patient with end stage renal disease. Hemodial Int. 2018 Jan;22(1):E1-E5. doi: 10.1111/hdi.12584. | Conference abstracts,etc |
|  | Chen LX, Josephson MA, Hedeker D, Campbell KH, Stankus N, Saunders MR. A Clinical Prediction Score to Guide Referral of Elderly Dialysis Patients for Kidney Transplant Evaluation. Kidney Int Rep. 2017 Jul;2(4):645-653. doi: 10.1016/j.ekir.2017.02.014. | Not relevant to the topic |
|  | Lee JY, Kim YO. Pre-existing arterial pathologic changes affecting arteriovenous fistula patency and cardiovascular mortality in hemodialysis patients. Korean J Intern Med. 2017 Sep;32(5):790-797. doi: 10.3904/kjim.2017.268. | Not relevant to the topic |
|  | Davies MG, Hicks TD, Haidar GM, El-Sayed HF. Outcomes of intervention for cephalic arch stenosis in brachiocephalic arteriovenous fistulas. J Vasc Surg. 2017 Nov;66(5):1504-1510. doi: 10.1016/j.jvs.2017.05.116. | Not relevant to the topic |
|  | Boitet A, Massy ZA, Goeau-Brissonniere O, Javerliat I, Coggia M, Coscas R. Drug-coated balloon angioplasty for dialysis access fistula stenosis. Semin Vasc Surg. 2016 Dec;29(4):178-185. doi: 10.1053/j.semvascsurg.2016.08.002. | Not relevant to the topic |
|  | Hu H, Patel S, Hanisch JJ, Santana JM, Hashimoto T, Bai H, Kudze T, Foster TR, Guo J, Yatsula B, Tsui J, Dardik A. Future research directions to improve fistula maturation and reduce access failure. Semin Vasc Surg. 2016 Dec;29(4):153-171. doi: 10.1053/j.semvascsurg.2016.08.005. | Not relevant to the topic |
|  | Gołębiowski T, Weyde W, Letachowicz K, Kusztal M, Augustyniak-Bartosik H, Penar J, Madziarska K, Zmonarski S, Krajewska M, Klinger M. The sleeve method for creation of radiocephalic arteriovenous fistulas in patients with calcified vessels. J Vasc Access. 2017 Sep 11;18(5):384-389. doi: 10.5301/jva.5000761. | Not relevant to the topic |
|  | Chisci E, Harris LM, Menici F, Frosini P, Romano E, Troisi N, Ercolini L, Michelagnoli S. Outcomes of three types of native arteriovenous fistula in a single center. J Vasc Access. 2017 Sep 11;18(5):379-383. doi: 10.5301/jva.5000742. | Not relevant to the topic |
|  | Shintaku S, Kawanishi H, Moriishi M, Ago R, Banshodani M, Hashimoto S, Tsuchiya S. Postoperative day 1 access blood flow and resistive index can predict patency in distal forearm arteriovenous fistula. J Vasc Access. 2017 Sep 11;18(5):371-378. doi: 10.5301/jva.5000777. | Not relevant to the topic |
|  | Arnaoutakis DJ, Deroo EP, McGlynn P, Coll MD, Belkin M, Hentschel DM, Ozaki CK. Improved outcomes with proximal radial-cephalic arteriovenous fistulas compared with brachial-cephalic arteriovenous fistulas. J Vasc Surg. 2017 Nov;66(5):1497-1503. doi: 10.1016/j.jvs.2017.04.075. | Not relevant to the topic |
|  | Diandra JC, Lo ZJ, Ang WW, Feng JF, Narayanan S, Tan GWL, Chandrasekar S. A Review of Arteriovenous Fistulae Creation in Octogenarians. Ann Vasc Surg. 2018 Jan;46:331-336. doi: 10.1016/j.avsg.2017.07.021. | Not relevant to the topic |
|  | Nassar GM, Beathard G, Rhee E, Khan AJ, Nguyen B. Management of transposed arteriovenous fistula swing point stenosis at the basilic vein angle of transposition by stent grafts. J Vasc Access. 2017 Nov 17;18(6):482-487. doi: 10.5301/jva.5000770. | Not relevant to the topic |
|  | Wang B, Rao A, Pappas K, Silpe J, Garlapati A, Talathi S, Mussa F, Landis GS, Etkin Y. Maturation Rates of Arteriovenous Fistulas Using Small Veins in the Era of Endovascular Interventions. Ann Vasc Surg. 2021 Feb;71:208-214. doi: 10.1016/j.avsg.2020.08.109. | Conference abstracts,etc |
|  | Roccatello D, Sciascia S, Rossi D, Naretto C, Bazzan M, Solfietti L, Baldovino S, Menegatti E. Outpatient percutaneous native renal biopsy: safety profile in a large monocentric cohort. BMJ Open. 2017 Jun 21;7(6):e015243. doi: 10.1136/bmjopen-2016-015243. | Not relevant to the topic |
|  | Fadrowski JJ, Hwang W, Neu AM, Fivush BA, Furth SL. Patterns of use of vascular catheters for hemodialysis in children in the United States. Am J Kidney Dis. 2009 Jan;53(1):91-8. doi: 10.1053/j.ajkd.2008.08.011. | Inconsistent with study population |
|  | Cunnane CV, Cunnane EM, Walsh MT. A Review of the Hemodynamic Factors Believed to Contribute to Vascular Access Dysfunction. Cardiovasc Eng Technol. 2017 Sep;8(3):280-294. doi: 10.1007/s13239-017-0307-0. | Not relevant to the topic |
|  | Beaulieu MC, Dumaine CS, Romann A, Kiaii M. Advanced age is not a barrier to creating a functional arteriovenous fistula: a retrospective study. J Vasc Access. 2017 Jul 14;18(4):307-312. doi: 10.5301/jva.5000710. | Not relevant to the topic |
|  | Senthoor D, Thant KZ, Ng TK, Ho P. Clinical Course of Hemodialysis Access After Initial Endovascular Intervention for Stenosis in Asian Renal Failure Patients. Vasc Endovascular Surg. 2017 Aug;51(6):363-367. doi: 10.1177/1538574417706639. | Not relevant to the topic |
|  | Al Shakarchi J, Hodson J, Field M, Inston N. Novel use of infrared thermal imaging to predict arteriovenous fistula patency and maturation. J Vasc Access. 2017 Jul 14;18(4):313-318. doi: 10.5301/jva.5000729. | Not relevant to the topic |
|  | Ismail A, Abushouk AI, Bekhet AH, Abunar O, Hassan O, Khamis AA, Al-Sayed M, Elgebaly A. Regional versus local anesthesia for arteriovenous fistula creation in end-stage renal disease: a systematic review and meta-analysis. J Vasc Access. 2017 May 15;18(3):177-184. doi: 10.5301/jva.5000683. | Not relevant to the topic |
|  | Thomas M, Nesbitt C, Ghouri M, Hansrani M. Maintenance of Hemodialysis Vascular Access and Prevention of Access Dysfunction: A Review. Ann Vasc Surg. 2017 Aug;43:318-327. doi: 10.1016/j.avsg.2017.02.014. | Not relevant to the topic |
|  | Aragoncillo I, Abad S, Caldés S, Amézquita Y, Vega A, Cirugeda A, Moratilla C, Ibeas J, Roca-Tey R, Fernández C, Macías N, Quiroga B, Blanco A, Villaverde M, Ruiz C, Martín B, Ruiz AM, Ampuero J, de Alvaro F, López-Gómez JM. Adding access blood flow surveillance reduces thrombosis and improves arteriovenous fistula patency: a randomized controlled trial. J Vasc Access. 2017 Jul 14;18(4):352-358. doi: 10.5301/jva.5000700. | Not relevant to the topic |
|  | Cho S, Lee YJ, Kim SR. Value of Doppler evaluation of physically abnormal fistula: hemodynamic guidelines and access outcomes. Korean J Intern Med. 2019 Jan;34(1):137-145. doi: 10.3904/kjim.2016.299. | Not relevant to the topic |
|  | Lerner B, Desrochers S, Tangri N. Risk Prediction Models in CKD. Semin Nephrol. 2017 Mar;37(2):144-150. doi: 10.1016/j.semnephrol. | Not relevant to the topic |
|  | Ben Ahmed S, Hadj-Abdelkader M, Benezit M, Deteix P, Heng AE, Rosset E. Predictors of Autogenous Arteriovenous Hemodialysis Access Thrombosis after Renal Transplantation. Ann Vasc Surg. 2017 Jul;42:231-237. doi: 10.1016/j.avsg.2017.01.008. | Not relevant to the topic |
|  | Chipde SS, Agrawal S, Kalathia J, Mishra U, Agrawal R. Basilic vein transposition: A viable alternative for multiple failed arteriovenous fistulas - A single center experience. Saudi J Kidney Dis Transpl. 2017 Mar-Apr;28(2):336-340. doi: 10.4103/1319-2442.202781. | Not relevant to the topic |
|  | Manne V, Vaddi SP, Reddy VB, Dayapule S. Factors influencing patency of Brescia-Cimino arteriovenous fistulas in hemodialysis patients. Saudi J Kidney Dis Transpl. 2017 Mar-Apr;28(2):313-317. doi: 10.4103/1319-2442.202759. | Not relevant to the topic |
|  | Salimi F, Shahabi S, Talebzadeh H, Keshavarzian A, Pourfakharan M, Safaei M. Evaluation of Diagnostic Values of Clinical Assessment in Determining the Maturation of Arteriovenous Fistulas for Satisfactory Hemodialysis. Adv Biomed Res. 2017 Mar 1;6:18. doi: 10.4103/2277-9175.201330. | Not relevant to the topic |
|  | Greenberg J, Jayarajan S, Reddy S, Schmieder FA, Roberts AB, van Bemmelen PS, Lee J, Choi ET. Long-Term Outcomes of Fistula First Initiative in an Urban University Hospital-Is It Still Relevant? Vasc Endovascular Surg. 2017 Apr;51(3):125-130. doi: 10.1177/1538574417692454. | Not relevant to the topic |
|  | Miyamoto M, Kurita N, Suemitsu K, Murakami M. Fistula and Survival Outcomes after Fistula Creation among Predialysis Chronic Kidney Disease Stage 5 Patients. Am J Nephrol. 2017;45(4):356-364. doi: 10.1159/000466707. | Not relevant to the topic |
|  | Baktiroglu S, Yanar F, Yuksel S, Celik B, Kilic HG. Elbow AVF configurations and indications. J Vasc Access. 2017 Mar 6;18(Suppl. 1):98-103. doi: 10.5301/jva.5000682. | Not relevant to the topic |
|  | Wilmink T, Hollingworth L, Stevenson T, Powers S. Is early cannulation of an arteriovenous fistula associated with early failure of the fistula? J Vasc Access. 2017 Mar 6;18(Suppl. 1):92-97. doi: 10.5301/jva.5000674. | Not relevant to the topic |
|  | Masengu A, Hanko J. Patient factors and haemodialysis arteriovenous fistula outcomes. J Vasc Access. 2017 Mar 6;18(Suppl. 1):19-23. doi: 10.5301/jva.5000665. | Conference abstracts,etc |
|  | Remuzzi A, Bozzetto M, Brambilla P. Is shear stress the key factor for AVF maturation? J Vasc Access. 2017 Mar 6;18(Suppl. 1):10-14. doi: 10.5301/jva.5000686. | Not relevant to the topic |
|  | Shenoy S. Surgical technique determines the outcome of the Brescia/Cimino AVF. J Vasc Access. 2017 Mar 6;18(Suppl. 1):1-4. doi: 10.5301/jva.5000698. | Not relevant to the topic |
|  | Bozzetto M, Rota S, Vigo V, Casucci F, Lomonte C, Morale W, Senatore M, Tazza L, Lodi M, Remuzzi G, Remuzzi A. Clinical use of computational modeling for surgical planning of arteriovenous fistula for hemodialysis. BMC Med Inform Decis Mak. 2017 Mar 14;17(1):26. doi: 10.1186/s12911-017-0420-x. | Not relevant to the topic |
|  | Cerneviciute R, Sahebally SM, Ahmed K, Murphy M, Mahmood W, Walsh SR. Regional Versus Local Anaesthesia for Haemodialysis Arteriovenous Fistula Formation: A Systematic Review and Meta-Analysis. Eur J Vasc Endovasc Surg. 2017 May;53(5):734-742. doi: 10.1016/j.ejvs.2017.01.025. | Not relevant to the topic |
|  | Al-Thani H, El-Menyar A, Al-Thani N, Asim M, Hussein A, Sadek A, Sharaf A, Fares A. Characteristics, Management, and Outcomes of Surgically Treated Arteriovenous Fistula Aneurysm in Patients on Regular Hemodialysis. Ann Vasc Surg. 2017 May;41:46-55. doi: 10.1016/j.avsg.2016.08.046. | Not relevant to the topic |
|  | Woo K, Ulloa J, Allon M, Carsten CG 3rd, Chemla ES, Henry ML, Huber TS, Lawson JH, Lok CE, Peden EK, Scher L, Sidawy A, Maggard-Gibbons M, Cull D. Establishing patient-specific criteria for selecting the optimal upper extremity vascular access procedure. J Vasc Surg. 2017 Apr;65(4):1089-1103.e1. doi: 10.1016/j.jvs.2016.10.099. | Not relevant to the topic |
|  | Siracuse JJ, Shah NK, Peacock MR, Tahhan G, Kalish JA, Rybin D, Eslami MH, Farber A. Thirty-day and 90-day hospital readmission after outpatient upper extremity hemodialysis access creation. J Vasc Surg. 2017 May;65(5):1376-1382. doi: 10.1016/j.jvs.2016.11.023. | Not relevant to the topic |
|  | Duque JC, Martinez L, Tabbara M, Dvorquez D, Mehandru SK, Asif A, Vazquez-Padron RI, Salman LH. Arteriovenous fistula maturation in patients with permanent access created prior to or after hemodialysis initiation. J Vasc Access. 2017 May 15;18(3):185-191. doi: 10.5301/jva.5000662. | Not relevant to the topic |
|  | Machowska A, Alscher MD, Vanga SR, Koch M, Aarup M, Qureshi AR, Lindholm B, Rutherford P. Dialysis access, infections, and hospitalisations in unplanned dialysis start patients: results from the OPTiONS study. Int J Artif Organs. 2017 Mar 16;40(2):48-59. doi: 10.5301/ijao.5000557. | Not relevant to the topic |
|  | Kats M, Hawxby AM, Barker J, Allon M. Impact of obesity on arteriovenous fistula outcomes in dialysis patients. Kidney Int. 2007 Jan;71(1):39-43. doi: 10.1038/sj.ki.5001904. | Conference abstracts,etc |
|  | Allon M. Arteriovenous Grafts: Much Maligned But in Need of Reconsideration? Semin Dial. 2017 Mar;30(2):125-133. doi: 10.1111/sdi.12567. | Not relevant to the topic |
|  | Reque J, Garcia-Prieto A, Linares T, Vega A, Abad S, Panizo N, Quiroga B, Collado Boira EJ, López-Gómez JM. Pulmonary Hypertension Is Associated with Mortality and Cardiovascular Events in Chronic Kidney Disease Patients. Am J Nephrol. 2017;45(2):107-114. doi: 10.1159/000453047. | Not relevant to the topic |
|  | Regus S, Almási-Sperling V, Rother U, Meyer A, Lang W. Surgeon experience affects outcome of forearm arteriovenous fistulae more than outcomes of upper-arm fistulae. J Vasc Access. 2017 Mar 21;18(2):120-125. doi: 10.5301/jva.5000639. | Not relevant to the topic |
|  | Rizvi SA, Usoh F, Hingorani A, Iadgarova E, Boniscavage P, Eisenberg J, Ascher E, Marks N. The Clinical Efficacy of Balloon-Assisted Maturation of Autogenous Arteriovenous Fistulae. Ann Vasc Surg. 2017 May;41:41-45. doi: 10.1016/j.avsg.2016.08.022. | Not relevant to the topic |
|  | Maldonado-Cárceles AB, García-Medina J, Torres-Cantero AM. Performance of physical examination versus ultrasonography to detect stenosis in haemodialysis arteriovenous fistula. J Vasc Access. 2017 Jan 18;18(1):30-34. doi: 10.5301/jva.5000616. | Not relevant to the topic |
|  | Zohny SF, El-Fattah MA, Khan JA. Circulating Fibroblast Growth Factor-23 Level and Paraoxonase-1 Lactonase Activity in Chronic Hemodialysis Patients: Their Impact on the Incidence of Native AV Fistula Thrombosis. Clin Invest Med. 2016 Oct 14;39(5):E173-E181. doi: 10.25011/cim.v39i5.27149. | Not relevant to the topic |
|  | Wu CC, Chen TY, Hsieh MY, Lin L, Yang CW, Chuang SY, Tarng DC. Monocyte Chemoattractant Protein-1 Levels and Postangioplasty Restenosis of Arteriovenous Fistulas. Clin J Am Soc Nephrol. 2017 Jan 6;12(1):113-121. doi: 10.2215/CJN.04030416. | Not relevant to the topic |
|  | Lee J, Kim S, Kim SM, Song R, Kim HK, Park JS, Park SC. Assessing radiocephalic wrist arteriovenous fistulas of obtuse anastomosis using computational fluid dynamics and clinical application. J Vasc Access. 2016 Nov 2;17(6):512-520. doi: 10.5301/jva.5000607. | Not relevant to the topic |
|  | Soleymanian T, Sheikh V, Tareh F, Argani H, Ossareh S. Hemodialysis vascular access and clinical outcomes: an observational multicenter study. J Vasc Access. 2017 Jan 18;18(1):35-42. doi: 10.5301/jva.5000610. | Not relevant to the topic |
|  | Ishii T, Suzuki Y, Nakayama T, Ohmori M, Masai S, Sasagawa N, Ohyama K. Duplex ultrasound for the prediction of vascular events associated with arteriovenous fistulas in hemodialysis patients. J Vasc Access. 2016 Nov 2;17(6):499-505. doi: 10.5301/jva.5000595. | Not relevant to the topic |
|  | Regus S, Almási-Sperling V, Lang W. Pediatric patients undergoing arteriovenous fistula surgery without intraoperative heparin. J Vasc Access. 2016 Nov 2;17(6):494-498. doi: 10.5301/jva.5000598. | Not relevant to the topic |
|  | Cnossen N, Kooman JP, Konings CJ, van Dantzig JM, van der Sande FM, Leunissen K. Peritoneal dialysis in patients with congestive heart failure. Nephrol Dial Transplant. 2006 Jul;21 Suppl 2:ii63-6. doi: 10.1093/ndt/gfl193. | Conference abstracts,etc |
|  | Masengu A, Hanko JB, Maxwell AP. Arterial stiffness and arteriovenous fistula failure of maturation. J Vasc Access. 2016 Nov 2;17(6):477-482. doi: 10.5301/jva.5000599. | Not relevant to the topic |
|  | Murakami M, Sakaguchi G, Mori N. Arteriovenous fistula combined with brachial artery superficialization is effective in patients with a high risk of maturation failure. J Vasc Surg. 2017 Feb;65(2):452-458. doi: 10.1016/j.jvs.2016.08.083. | Not relevant to the topic |
|  | Tirinescu DC, Bondor CI, Vlăduțiu DŞ, Pațiu IM, Moldovan D, Orășan R, Kacsó IM. Ultrasonographic diagnosis of stenosis of native arteriovenous fistulas in haemodialysis patients. Med Ultrason. 2016 Sep;18(3):332-8. doi: 10.11152/mu.2013.2066.183.fis. | Not relevant to the topic |
|  | Kim SM, Min SK, Ahn S, Min SI, Ha J. Outcomes of Arteriovenous Fistula for Hemodialysis in Pediatric and Adolescent Patients. Vasc Specialist Int. 2016 Sep;32(3):113-118. doi: 10.5758/vsi.2016.32.3.113. | Not relevant to the topic |
|  | Ragupathi L, Johnson D, Marhefka GD. Right Ventricular Enlargement within Months of Arteriovenous Fistula Creation in 2 Hemodialysis Patients. Tex Heart Inst J. 2016 Aug 1;43(4):350-3. doi: 10.14503/THIJ-15-5353. | Conference abstracts,etc |
|  | Wo K, Morrison BJ, Harada RN. Developing Duplex Ultrasound Criteria for Diagnosis of Arteriovenous Fistula Stenosis. Ann Vasc Surg. 2017 Jan;38:99-104. doi: 10.1016/j.avsg.2016.04.013. | Conference abstracts,etc |
|  | Joseph Lo Z, Tay WM, Lee Q, Chua JL, Tan GW, Chandrasekar S, Narayanan S. Predictors of radio-cephalic arteriovenous fistulae patency in an Asian population. J Vasc Access. 2016 Sep 21;17(5):411-416. doi: 10.5301/jva.5000591. | Not relevant to the topic |
|  | Huber TS, Larive B, Imrey PB, Radeva MK, Kaufman JM, Kraiss LW, Farber AM, Berceli SA; HFM Study Group. Access-related hand ischemia and the Hemodialysis Fistula Maturation Study. J Vasc Surg. 2016 Oct;64(4):1050-1058.e1. doi: 10.1016/j.jvs.2016.03.449. | Not relevant to the topic |
|  | Domenick Sridharan N, Fish L, Yu L, Weisbord S, Jhamb M, Makaroun MS, Yuo TH. The associations of hemodialysis access type and access satisfaction with health-related quality of life. J Vasc Surg. 2018 Jan;67(1):229-235. doi: 10.1016/j.jvs.2017.05.131. | Conference abstracts,etc |
|  | Pushevski V, Dejanov P, Gerasimovska V, Petrushevska G, Oncevski A, Sikole A, Popov Z, Ivanovski N. Severe Endothelial Damage in Chronic Kidney Disease Patients Prior to Haemodialysis Vascular Access Surgery. Pril (Makedon Akad Nauk Umet Odd Med Nauki). 2015;36(3):43-9. doi: 10.1515/prilozi-2015-0077. | Not relevant to the topic |
|  | Sidhu A, Tan KT, Noel-Lamy M, Simons ME, Rajan DK. Does Technical Success of Angioplasty in Dysfunctional Hemodialysis Accesses Correlate with Access Patency? Cardiovasc Intervent Radiol. 2016 Oct;39(10):1400-6. doi: 10.1007/s00270-016-1401-7. | Not relevant to the topic |
|  | Barreto P, Almeida P, de Matos N, Queirós JA, Pinheiro J, Silva F, Carvalho T, Almeida R, Cabrita A. Preoperative vessel mapping in chronic kidney disease patients - a center experience. J Vasc Access. 2016 Jul 12;17(4):320-7. doi: 10.5301/jva.5000559. | Not relevant to the topic |
|  | Fu H, An M, Zhang H, Song Y, Zhang Y. Preventing embolus shedding from an arteriovenous fistula during hemodialysis treatment. J Vasc Access. 2016 Jul 12;17(4):e73-4. doi: 10.5301/jva.5000568. | Conference abstracts,etc |
|  | Kudlaty EA, Kendrick DE, Allemang MT, Kashyap VS, Wong VL. Upper Extremity Steal Syndrome Is Associated with Atherosclerotic Burden and Access Configuration. Ann Vasc Surg. 2016 Aug;35:82-7. doi: 10.1016/j.avsg.2016.01.058. | Not relevant to the topic |
|  | Ryu YG, Lee DK, Baek MJ, Kim H. Clinical Value of Intraoperative Transit-Time Flow Measurement for Autogenous Radiocephalic Arteriovenous Fistula in Patients with Chronic Kidney Disease. Ann Vasc Surg. 2016 Aug;35:53-9. doi: 10.1016/j.avsg.2016.02.018. | Not relevant to the topic |
|  | Wohlfahrt P, Rokosny S, Melenovsky V, Borlaug BA, Pecenkova V, Balaz P. Cardiac remodeling after reduction of high-flow arteriovenous fistulas in end-stage renal disease. Hypertens Res. 2016 Sep;39(9):654-9. doi: 10.1038/hr.2016.50. | Not relevant to the topic |
|  | Itoga NK, Ullery BW, Tran K, Lee GK, Aalami OO, Bech FR, Zhou W. Use of a proactive duplex ultrasound protocol for hemodialysis access. J Vasc Surg. 2016 Oct;64(4):1042-1049.e1. doi: 10.1016/j.jvs.2016.03.442. | Not relevant to the topic |
|  | Goh MA, Ali JM, Iype S, Pettigrew GJ. Outcomes of primary arteriovenous fistulas in patients older than 70 years. J Vasc Surg. 2016 May;63(5):1333-40. doi: 10.1016/j.jvs.2015.12.044. | Not relevant to the topic |
|  | Hammes M, Boghosian M, Cassel K, Watson S, Funaki B, Doshi T, Mahmoudzadeh Akherat SM, Hines J, Coe F. Increased Inlet Blood Flow Velocity Predicts Low Wall Shear Stress in the Cephalic Arch of Patients with Brachiocephalic Fistula Access. PLoS One. 2016 Apr 13;11(4):e0152873. doi: 10.1371/journal.pone.0152873. | Not relevant to the topic |
|  | Kim SM, Han Y, Kwon H, Hong HS, Choi JY, Park H, Kwon TW, Cho YP. Impact of a preoperative evaluation on the outcomes of an arteriovenous fistula. Ann Surg Treat Res. 2016 Apr;90(4):224-30. doi: 10.4174/astr.2016.90.4.224. | Not relevant to the topic |
|  | Eroglu E, Kocyigit I, Saraymen B, Tuncay A, Mavili E, Unal A, Avcilar H, Koker MY, Oymak O. The association of endothelial progenitor cell markers with arteriovenous fistula maturation in hemodialysis patients. Int Urol Nephrol. 2016 Jun;48(6):891-9. doi: 10.1007/s11255-016-1282-3. | Not relevant to the topic |
|  | Ladenheim ED, Lulic D, Lum C, Agrawal S, Chadwick N. First-week postoperative flow measurements are highly predictive of primary patency of radiocephalic arteriovenous fistulas. J Vasc Access. 2016 Jul 12;17(4):307-12. doi: 10.5301/jva.5000544. | Not relevant to the topic |
|  | Zhu YL, Ding H, Fan PL, Gu QL, Teng J, Wang WP. Predicting the maturity of haemodialysis arteriovenous fistulas with colour Doppler ultrasound: a single-centre study from China. Clin Radiol. 2016 Jun;71(6):576-82. doi: 10.1016/j.crad.2016.02.025. | Not relevant to the topic |
|  | Tabbara M, Duque JC, Martinez L, Escobar LA, Wu W, Pan Y, Fernandez N, Velazquez OC, Jaimes EA, Salman LH, Vazquez-Padron RI. Pre-existing and Postoperative Intimal Hyperplasia and Arteriovenous Fistula Outcomes. Am J Kidney Dis. 2016 Sep;68(3):455-64. doi: 10.1053/j.ajkd.2016.02.044. | Not relevant to the topic |
|  | Shah AS, Valdes J, Charlton-Ouw KM, Chen Z, Coogan SM, Amer HM, Estrera AL, Safi HJ, Azizzadeh A. Endovascular treatment of hemodialysis access pseudoaneurysms. J Vasc Surg. 2012 Apr;55(4):1058-62. doi: 10.1016/j.jvs.2011.10.126. | Conference abstracts,etc |
|  | Wilmink T, Hollingworth L, Dasgupta I. Access ligation in transplant patients. J Vasc Access. 2016 Mar;17 Suppl 1:S64-8. doi: 10.5301/jva.5000537. | Not relevant to the topic |
|  | Roca-Tey R. Permanent arteriovenous fistula or catheter dialysis for heart failure patients. J Vasc Access. 2016 Mar;17 Suppl 1:S23-9. doi: 10.5301/jva.5000511. | Not relevant to the topic |
|  | Mien, T. C.; Yahud, S. Assessment of an Arterial Stiffness to Demonstrate AVF Performance: Modelling Approach. J. Phys.: Conf. Ser. 2019, 1372 (1), 012057. https://doi.org/10.1088/1742-6596/1372/1/012057. | Conference abstracts,etc |
|  | Al Shakarchi J, McGrogan D, Van der Veer S, Sperrin M, Inston N. Predictive models for arteriovenous fistula maturation. J Vasc Access. 2016 May 7;17(3):229-32. doi: 10.5301/jva.5000500. | Not relevant to the topic |
|  | Smith GE, Barnes R, Fagan M, Chetter IC. The Impact of Vein Mechanical Compliance on Arteriovenous Fistula Outcomes. Ann Vasc Surg. 2016 Apr;32:9-14. doi: 10.1016/j.avsg.2015.11.002. | Not relevant to the topic |
|  | Smith GE, Barnes R, Fagan M, Chetter IC. The Impact of Vein Mechanical Compliance on Arteriovenous Fistula Outcomes. Ann Vasc Surg. 2016 Apr;32:9-14. doi: 10.1016/j.avsg.2015.11.002. | Not relevant to the topic |
|  | Masengu A, McDaid J, Maxwell AP, Hanko JB. Preoperative radial artery volume flow is predictive of arteriovenous fistula outcomes. J Vasc Surg. 2016 Feb;63(2):429-35. doi: 10.1016/j.jvs.2015.08.106. | Not relevant to the topic |
|  | Korepta LM, Watson JJ, Elder EA, Davis AT, Mansour MA, Chambers CM, Cuff RF, Wong PY. Outcomes for forearm and upper arm arteriovenous fistula creation with the transposition technique. J Vasc Surg. 2016 Mar;63(3):764-71. doi: 10.1016/j.jvs.2015.09.049. | Not relevant to the topic |
|  | Cui J, Steele D, Wenger J, Kawai T, Liu F, Elias N, Watkins MT, Irani Z. Hemodialysis arteriovenous fistula as first option not necessary in elderly patients. J Vasc Surg. 2016 May;63(5):1326-32. doi: 10.1016/j.jvs.2015.11.036. | Not relevant to the topic |
|  | Wilmink T, Hollingworth L, Powers S, Allen C, Dasgupta I. Natural History of Common Autologous Arteriovenous Fistulae: Consequences for Planning of Dialysis. Eur J Vasc Endovasc Surg. 2016 Jan;51(1):134-40. doi: 10.1016/j.ejvs.2015.10.005. | Not relevant to the topic |
|  | Liang Y, Sun N, Wang H, Shen Y. [Analysis on factors relevant to operation success rate of internal arteriovenous fistula used in pediatric patients with end stage renal disease]. Zhonghua Er Ke Za Zhi. 2015 Sep;53(9):660-4. Chinese. | Not relevant to the topic |
|  | Smith GE, Souroullos P, Cayton T, Harwood A, Carradice D, Chetter IC. A systematic review and meta-analysis of systemic intraoperative anticoagulation during arteriovenous access formation for dialysis. J Vasc Access. 2016 Jan-Feb;17(1):1-5. doi: 10.5301/jva.5000484. | Not relevant to the topic |
|  | Farber A, Imrey PB, Huber TS, Kaufman JM, Kraiss LW, Larive B, Li L, Feldman HI; HFM Study Group. Multiple preoperative and intraoperative factors predict early fistula thrombosis in the Hemodialysis Fistula Maturation Study. J Vasc Surg. 2016 Jan;63(1):163-70.e6. doi: 10.1016/j.jvs.2015.07.086. | Not relevant to the topic |
|  | Murley A, Wijewardane A, Wilmink T, Baharani J. Should patients be on antithrombotic medication for their first arteriovenous fistulae? J Vasc Access. 2016 Mar-Apr;17(2):118-23. doi: 10.5301/jva.5000491. | Not relevant to the topic |
|  | Chen CF, Lin CC. The association of genotype polymorphisms with vascular access patency in hemodialysis patients. J Vasc Access. 2019 May;20(1_suppl):24-30. doi: 10.1177/1129729818758556. | Conference abstracts,etc |
|  | Gkotsis G, Jennings WC, Malik J, Mallios A, Taubman K. Treatment of High Flow Arteriovenous Fistulas after Successful Renal Transplant Using a Simple Precision Banding Technique. Ann Vasc Surg. 2016 Feb;31:85-90. doi: 10.1016/j.avsg.2015.08.012. | Not relevant to the topic |
|  | Jadlowiec CC, Mannion EM, Lavallee M, Brown MG. Hemodialysis Access in the Elderly: Outcomes among Patients Older than Seventy. Ann Vasc Surg. 2016 Feb;31:77-84. doi: 10.1016/j.avsg.2015.08.013. | Not relevant to the topic |
|  | Malka KT, Flahive J, Csizinscky A, Aiello F, Simons JP, Schanzer A, Messina LM, Robinson WP. Results of repeated percutaneous interventions on failing arteriovenous fistulas and grafts and factors affecting outcomes. J Vasc Surg. 2016 Mar;63(3):772-7. doi: 10.1016/j.jvs.2015.09.031. | Not relevant to the topic |
|  | Jennings WC, Kindred MG, Broughan TA. Creating radiocephalic arteriovenous fistulas: technical and functional success. J Am Coll Surg. 2009 Mar;208(3):419-25. doi: 10.1016/j.jamcollsurg.2008.11.015. | Conference abstracts,etc |
|  | Cheng Y, Zhang F, Zhu J, Wang T, Wei M, Guo D, Mo L, Zhu C, Wang X. Influence of blood pressure variability on the life of arteriovenous fistulae in maintenance hemodialysis patients. Clin Hemorheol Microcirc. 2016;62(2):129-37. doi: 10.3233/CH-151959. | Not relevant to the topic |
|  | McDonnell SM, Frueh J, Blecha M, Aulivola B, Halandras PM. Health care disparities involved in establishing functional arteriovenous fistula hemodialysis access. J Vasc Surg. 2023 Sep;78(3):774-778. doi: 10.1016/j.jvs.2023.04.038. | Conference abstracts,etc |
|  | Aragoncillo I, Amézquita Y, Caldés S, Abad S, Vega A, Cirugeda A, Moratilla C, Ibeas J, Roca-Tey R, Fernández C, Quiroga B, Blanco A, Villaverde M, Ruiz C, Martín B, Ruiz AM, Ampuero J, López-Gómez JM, de Alvaro F. The impact of access blood flow surveillance on reduction of thrombosis in native arteriovenous fistula: a randomized clinical trial. J Vasc Access. 2016 Jan-Feb;17(1):13-9. doi: 10.5301/jva.5000461. | Not relevant to the topic |
|  | Perl J, Nessim SJ, Moist LM, Wald R, Na Y, Tennankore KK, Chan CT. Vascular Access Type and Patient and Technique Survival in Home Hemodialysis Patients: The Canadian Organ Replacement Register. Am J Kidney Dis. 2016 Feb;67(2):251-9. doi: 10.1053/j.ajkd.2015.07.032. | Not relevant to the topic |
|  | Kanno T, Kamijo Y, Hashimoto K, Kanno Y. Outcomes of blood flow suppression methods of treating high flow access in hemodialysis patients with arteriovenous fistula. J Vasc Access. 2015 Nov;16 Suppl 10:S28-33. doi: 10.5301/jva.5000415. | Not relevant to the topic |
|  | Sato T, Tsuboi M, Onogi T, Miwa N, Sakurai H, Ookubo K, Matsubara C, Kasuga H. Standard procedures of endovascular treatment for vascular access stenosis in our facility - clinical usefulness of ultrasonography. J Vasc Access. 2015 Nov;16 Suppl 10:S34-7. doi: 10.5301/jva.5000460. | Not relevant to the topic |
|  | Murakami M, Miyamoto M, Suemitsu K. Percutaneous transluminal angioplasty in Japan: five-center investigation. J Vasc Access. 2015 Nov;16 Suppl 10:S38-42. doi: 10.5301/jva.5000426. | Not relevant to the topic |
|  | Zhou L, Liu H, Liu F, Wu H, Zhang L, Li Z, Li J. [Survival analysis and risk factors for arteriovenous fistula  in 472 patients]. Zhong Nan Da Xue Xue Bao Yi Xue Ban. 2015 Aug;40(8):902-6. Chinese. doi: 10.11817/j.issn.1672-7347.2015.08.013. | Not relevant to the topic |
|  | Alkhouli M, Sandhu P, Boobes K, Hatahet K, Raza F, Boobes Y. Cardiac complications of arteriovenous fistulas in patients with end-stage renal disease. Nefrologia. 2015;35(3):234-45. doi: 10.1016/j.nefro.2015.03.001. | Not relevant to the topic |
|  | Barnes R, Smith GE, Chetter IC. A prospective observational study to assess the impact of operator seniority on outcome following arteriovenous fistula formation. J Vasc Access. 2015 Sep-Oct;16(5):372-6. doi: 10.5301/jva.5000401. | Not relevant to the topic |
|  | Zarkowsky DS, Hicks CW, Arhuidese I, Canner JK, Obeid T, Qazi U, Schneider E, Abularrage CJ, Black JH 3rd, Freischlag JA, Malas MB. Quality Improvement Targets for Regional Variation in Surgical End-Stage Renal Disease Care. JAMA Surg. 2015 Aug;150(8):764-70. doi: 10.1001/jamasurg.2015.1126. | Not relevant to the topic |
|  | Al Shakarchi J, Houston G, Inston N. Early cannulation grafts for haemodialysis: a systematic review. J Vasc Access. 2015 Nov-Dec;16(6):493-7. doi: 10.5301/jva.5000412. | Not relevant to the topic |
|  | Swinnen JJ, Zahid A, Burgess DC. Paclitaxel drug-eluting balloons to recurrent in-stent stenoses in autogenous dialysis fistulas: a retrospective study. J Vasc Access. 2015 Sep-Oct;16(5):388-93. doi: 10.5301/jva.5000396. | Not relevant to the topic |
|  | Verest S, Logghe P, Claes K, Kuypers D, Fourneau I. Effect of clinical examination and anatomical location on native arteriovenous fistula maturation rate in high risk patients. Acta Chir Belg. 2014 Sep-Oct;114(5):324-31. | Not relevant to the topic |
|  | Jin WT, Zhang GF, Liu HC, Zhang H, Li B, Zhu XQ. Non-contrast-enhanced MR angiography for detecting arteriovenous fistula dysfunction in haemodialysis patients. Clin Radiol. 2015 Aug;70(8):852-7. doi: 10.1016/j.crad.2015.04.005. | Not relevant to the topic |
|  | Zhang JC, Al-Jaishi A, Perl J, Garg AX, Moist LM. Hemodialysis Arteriovenous Vascular Access Creation After Kidney Transplant Failure. Am J Kidney Dis. 2015 Oct;66(4):646-54. doi: 10.1053/j.ajkd.2015.03.031. | Not relevant to the topic |
|  | Rosenberg JE, Yevzlin AS, Chan MR, Valliant AM, Astor BC. Prediction of Arteriovenous Fistula Dysfunction: Can it be Taught? Semin Dial. 2015 Sep-Oct;28(5):544-7. doi: 10.1111/sdi.12361. | Not relevant to the topic |
|  | Yigla M, Nakhoul F, Sabag A, Tov N, Gorevich B, Abassi Z, Reisner SA. Pulmonary hypertension in patients with end-stage renal disease. Chest. 2003 May;123(5):1577-82. doi: 10.1378/chest.123.5.1577. | Conference abstracts,etc |
|  | Maleux G, De Coster B, Laenen A, Vaninbroukx J, Meijers B, Claes K, Fourneau I, Heye S. Percutaneous rheolytic thrombectomy of thrombosed autogenous dialysis fistulas: technical results, clinical outcome, and factors influencing patency. J Endovasc Ther. 2015 Feb;22(1):80-6. doi: 10.1177/1526602814566378. | Not relevant to the topic |
|  | Leake AE, Yuo TH, Wu T, Fish L, Dillavou ED, Chaer RA, Leers SA, Makaroun MS. Arteriovenous grafts are associated with earlier catheter removal and fewer catheter days in the United States Renal Data System population. J Vasc Surg. 2015 Jul;62(1):123-7. doi: 10.1016/j.jvs.2015.02.018. | Not relevant to the topic |
|  | Ko SH, Bandyk DF, Hodgkiss-Harlow KD, Barleben A, Lane J 3rd. Estimation of brachial artery volume flow by duplex ultrasound imaging predicts dialysis access maturation. J Vasc Surg. 2015 Jun;61(6):1521-7. doi: 10.1016/j.jvs.2015.01.036. | Conference abstracts,etc |
|  | Patel MS, Davies MG, Nassar GM, Naoum JJ. Open repair and venous inflow plication of the arteriovenous fistula is effective in treating vascular steal syndrome. Ann Vasc Surg. 2015 Jul;29(5):927-33. doi: 10.1016/j.avsg.2014.12.042. | Not relevant to the topic |
|  | Bourquelot P, Karam L, Robert-Ebadi H, Pirozzi N. Transposition, elevation, lipectomy and V-Wing for easy needling. J Vasc Access. 2015;16 Suppl 9:S108-13. doi: 10.5301/jva.5000353. | Not relevant to the topic |
|  | Shi Y, Zhu M, Cheng J, Zhang J, Ni Z. Venous stenosis in chronic dialysis patients with a well-functioning arteriovenous fistula. Vascular. 2016 Feb;24(1):25-30. doi: 10.1177/1708538115575649. | Not relevant to the topic |
|  | Wu CC, Jiang H, Cheng J, Zhao LF, Sheng KX, Chen JH. The outcome of the proximal radial artery arteriovenous fistula. J Vasc Surg. 2015 Mar;61(3):802-8. doi: 10.1016/j.jvs.2014.08.112. | Not relevant to the topic |
|  | Allon M, Robbin ML, Umphrey HR, Young CJ, Deierhoi MH, Goodman J, Hanaway M, Lockhart ME, Barker-Finkel J, Litovsky S. Preoperative arterial microcalcification and clinical outcomes of arteriovenous fistulas for hemodialysis. Am J Kidney Dis. 2015 Jul;66(1):84-90. doi: 10.1053/j.ajkd.2014.12.015. | Not relevant to the topic |
|  | Aktas A, Bozkurt A, Aktas B, Kirbas I. Percutaneous transluminal balloon angioplasty in stenosis of native hemodialysis arteriovenous fistulas: technical success and analysis of factors affecting postprocedural fistula patency. Diagn Interv Radiol. 2015 Mar-Apr;21(2):160-6. doi: 10.5152/dir.2014.14348. | Not relevant to the topic |
|  | Lazarides M, Georgiadis G, Argyriou C. Safety issues in surgical and endovascular techniques to rescue failing or failed arteriovenous fistulas and arteriovenous grafts. Contrib Nephrol. 2015;184:153-63. doi: 10.1159/000365912. | Not relevant to the topic |
|  | King DH, Paulson WD, Al-Qaisi M, Taylor MG, Panayiotopoulos Y, Abeygunarsekara S, Chan A, Ali G, Chemla ES. Volume blood flow, static pressure ratio and venous conductance in native arterio-venous fistulae: three surveillance methods compared. J Vasc Access. 2015 May-Jun;16(3):211-7. doi: 10.5301/jva.5000324. | Not relevant to the topic |
|  | Aitken E, Kerr D, Geddes C, Berry C, Kingsmore D. Cardiovascular changes occurring with occlusion of a mature arteriovenous fistula. J Vasc Access. 2015 Nov-Dec;16(6):459-66. doi: 10.5301/jva.5000336. | Not relevant to the topic |
|  | McGrogan DG, Field MA, Maxwell AP, Marie Y, Inston NG. Patient survival following arteriovenous fistula formation. J Vasc Access. 2015 May-Jun;16(3):195-9. doi: 10.5301/jva.5000343. | Not relevant to the topic |
|  | Mufty H, Claes K, Heye S, Fourneau I. Proactive surveillance approach to guarantee a functional arteriovenous fistula at first dialysis is worth. J Vasc Access. 2015 May-Jun;16(3):183-8. doi: 10.5301/jva.5000329. | Not relevant to the topic |
|  | Erickson KF, Mell MW, Winkelmayer WC, Chertow GM, Bhattacharya J. Provider visit frequency and vascular access interventions in hemodialysis. Clin J Am Soc Nephrol. 2015 Feb 6;10(2):269-77. doi: 10.2215/CJN.05540614. | Not relevant to the topic |
|  | Lv L, Huang W, Zhang J, Shi Y, Zhang L. Altered microRNA expression in stenoses of native arteriovenous fistulas in hemodialysis patients. J Vasc Surg. 2016 Apr;63(4):1034-43.e3. doi: 10.1016/j.jvs.2014.10.099. | Not relevant to the topic |
|  | Dhamija R, Nash SK, Nguyen SV, Slack K, Tadeo J. Monitoring and Surveillance of Hemodialysis Vascular Access Using StenTec and Physical Exam. Semin Dial. 2015 May-Jun;28(3):299-304. doi: 10.1111/sdi.12311. | Not relevant to the topic |
|  | Banerjee T, Kim SJ, Astor B, Shafi T, Coresh J, Powe NR. Vascular access type, inflammatory markers, and mortality in incident hemodialysis patients: the Choices for Healthy Outcomes in Caring for End-Stage Renal Disease (CHOICE) Study. Am J Kidney Dis. 2014 Dec;64(6):954-61. doi: 10.1053/j.ajkd.2014.07.010. | Not relevant to the topic |
|  | Mauro CR, Ding K, Xue H, Tao M, Longchamp A, Belkin M, Kristal BS, Ozaki CK. Adipose phenotype predicts early human autogenous arteriovenous hemodialysis remodeling. J Vasc Surg. 2016 Jan;63(1):171-6.e1. doi: 10.1016/j.jvs.2014.06.110. | Not relevant to the topic |
|  | Yilmaz H, Bozkurt A, Cakmak M, Celik HT, Bilgic MA, Bavbek N, Akcay A. Relationship between late arteriovenous fistula (AVF) stenosis and neutrophil-lymphocyte ratio (NLR) in chronic hemodialysis patients. Ren Fail. 2014 Oct;36(9):1390-4. doi: 10.3109/0886022X.2014.945183. | Not relevant to the topic |
|  | Gibyeli Genek D, Tuncer Altay C, Unek T, Sifil A, Seçil M, Camsari T. Can primary failure of arteriovenous fistulas be anticipated? Hemodial Int. 2015 Apr;19(2):296-305. doi: 10.1111/hdi.12206. | Not relevant to the topic |
|  | Dageforde LA, Harms KA, Feurer ID, Shaffer D. Increased minimum vein diameter on preoperative mapping with duplex ultrasound is associated with arteriovenous fistula maturation and secondary patency. J Vasc Surg. 2015 Jan;61(1):170-6. doi: 10.1016/j.jvs.2014.06.092. | Not relevant to the topic |
|  | Lynch SK, Ahanchi SS, Dexter DJ, Glickman MH, Panneton JM. Patient compliance limits the efforts of quality improvement initiatives on arteriovenous fistula maturation. J Vasc Surg. 2015 Jan;61(1):184-91. doi: 10.1016/j.jvs.2014.05.095. | Not relevant to the topic |
|  | Neuen BL, Gunnarsson R, Baer RA, Tosenovsky P, Green SJ, Golledge J, Mantha ML. Factors associated with patency following angioplasty of hemodialysis fistulae. J Vasc Interv Radiol. 2014 Sep;25(9):1419-26. doi: 10.1016/j.jvir.2014.05.020. | Not relevant to the topic |
|  | Elramah M, Boujelbane L, Yevzlin AS, Wakeen M, Astor BC, Chan MR. Dialysis access venous stenosis: treatment with balloon angioplasty 30-second vs. 1-minute inflation times. Hemodial Int. 2015 Jan;19(1):108-14. doi: 10.1111/hdi.12183. | Not relevant to the topic |
|  | Gowda A, Pavan M, Babu K. Vascular access profile in maintenance hemodialysis patients. Iran J Kidney Dis. 2014 May;8(3):218-24. | Not relevant to the topic |
|  | Memetoğlu ME, Kehlіbar T, Yilmaz M, Kocaaslan C, Günay R, Arslan İY, Ketencі B, Demіrtas MM. Red blood cell distribution width is associated with early failure of arteriovenous fistula for haemodialysis access. Blood Coagul Fibrinolysis. 2015 Jan;26(1):32-5. doi: 10.1097/MBC.0000000000000142. | Not relevant to the topic |
|  | Stoumpos S, Stevens KK, Aitken E, Kingsmore DB, Clancy MJ, Fox JG, Geddes CC. Predictors of sustained arteriovenous access use for haemodialysis. Am J Nephrol. 2014;39(6):491-8. doi: 10.1159/000362744. | Not relevant to the topic |
|  | Wang HY, Wu CH, Chen CY, Lin BS. Novel noninvasive approach for detecting arteriovenous fistula stenosis. IEEE Trans Biomed Eng. 2014 Jun;61(6):1851-7. doi: 10.1109/TBME.2014.2308906. | Not relevant to the topic |
|  | Gibbons CP. Which accesses should be abandoned or revised? J Vasc Access. 2014;15 Suppl 7:S76-80. doi: 10.5301/jva.5000227. | Not relevant to the topic |
|  | Remuzzi A, Manini S. Computational model for prediction of fistula outcome. J Vasc Access. 2014;15 Suppl 7:S64-9. doi: 10.5301/jva.5000241. | Not relevant to the topic |
|  | Krivitski N. Why vascular access trials on flow surveillance failed. J Vasc Access. 2014;15 Suppl 7:S15-9. doi: 10.5301/jva.5000256. | Not relevant to the topic |
|  | Malovrh M. Postoperative assessment of vascular access. J Vasc Access. 2014;15 Suppl 7:S10-4. doi: 10.5301/jva.5000243. | Not relevant to the topic |
|  | Mandolfo S, Acconcia P, Bucci R, Corradi B, Farina M, Rizzo MA, Stucchi A. Hemodialysis tunneled central venous catheters: five-year outcome analysis. J Vasc Access. 2014 Nov-Dec;15(6):461-5. doi: 10.5301/jva.5000236. | Not relevant to the topic |
|  | Zhang C, Cui T, Zhao S, Wang S, Zhang X. [The incidence of aspirin resistance and relevant influencing factors in patients on maintenance hemodialysis]. Zhonghua Nei Ke Za Zhi. 2014 Mar;53(3):178-83. Chinese. | Not relevant to the topic |
|  | Zhu M, Zhang W, Zhou W, Zhou Y, Fang Y, Wang Y, Zhang H, Yan Y, Ni Z, Qian J. Initial hemodialysis with a temporary catheter is associated with complications of a later permanent vascular access. Blood Purif. 2014;37(2):131-7. doi: 10.1159/000360269. | Not relevant to the topic |
|  | Li M, Zhang Z, Yu Y, Chen H, Li X, Ma J, Dong Z. Clinical application of long-term Palindrome catheter in hemodialysis patients. Iran J Kidney Dis. 2014 Mar;8(2):123-9. | Not relevant to the topic |
|  | Drew DA, Lok CE. Strategies for planning the optimal dialysis access for an individual patient. Curr Opin Nephrol Hypertens. 2014 May;23(3):314-20. doi: 10.1097/01.mnh.0000444815.49755.d9. | Not relevant to the topic |
|  | Stolic RV, Trajkovic GZ, Kostic M, Stolic DZ, Miric DJ, Kisic BM, Pajovic SD, Peric VM. Predictive value of serum myeloperoxidase activity for thrombosis of arteriovenous fistulas. Hemodial Int. 2014 Jul;18(3):680-5. doi: 10.1111/hdi.12155. | Not relevant to the topic |
|  | Bilgic MA, Yilmaz H, Bozkurt A, Celik HT, Bilgic IC, Gurel OM, Kirbas I, Bavbek N, Akcay A. Relationship of late arteriovenous fistula stenosis with soluble E-selectin and soluble EPCR in chronic hemodialysis patients with arteriovenous fistula. Clin Exp Nephrol. 2015 Feb;19(1):133-9. doi: 10.1007/s10157-014-0955-4. | Not relevant to the topic |
|  | Yoo DW, Yoon M, Jun HJ. Successful Access Rate and Risk Factor of Vascular Access Surgery in Arm for Dialysis. Vasc Specialist Int. 2014 Mar;30(1):33-7. doi: 10.5758/vsi.2014.30.1.33. | Not relevant to the topic |
|  | Lee T, Somarathna M, Hura A, Wang Y, Campos B, Arend L, Munda R, Roy-Chaudhury P. Natural history of venous morphologic changes in dialysis access stenosis. J Vasc Access. 2014 Jul-Aug;15(4):298-305. doi: 10.5301/jva.5000212. | Not relevant to the topic |
|  | Aitken EL, Stevenson KS, Gingell-Littlejohn M, Aitken M, Clancy M, Kingsmore DB. The use of tunneled central venous catheters: inevitable or system failure? J Vasc Access. 2014 Sep-Oct;15(5):344-50. doi: 10.5301/jva.5000206. | Not relevant to the topic |
|  | Ferring M, Henderson J, Wilmink T. Accuracy of early postoperative clinical and ultrasound examination of arteriovenous fistulae to predict dialysis use. J Vasc Access. 2014 Jul-Aug;15(4):291-7. doi: 10.5301/jva.5000210. | Not relevant to the topic |
|  | Fila B, Lovčić V, Sonicki Z, Magaš S, Sudar-Magaš Z, Malovrh M. Vein diameter after intraoperative dilatation with vessel probes as a predictor of success of hemodialysis arteriovenous fistulas. Med Sci Monit. 2014 Feb 5;20:191-8. doi: 10.12659/MSM.890155. | Not relevant to the topic |
|  | Lin CL, Chen HY, Huang SC, Hsu SP, Pai MF, Peng YS, Chiu YL. Increased blood loss from access cannulation site during hemodialysis is associated with anemia and arteriovenous graft use. Ther Apher Dial. 2014 Feb;18(1):51-6. doi: 10.1111/1744-9987.12026. | Not relevant to the topic |
|  | Tessitore N, Bedogna V, Verlato G, Poli A. The rise and fall of access blood flow surveillance in arteriovenous fistulas. Semin Dial. 2014 Mar;27(2):108-18. doi: 10.1111/sdi.12187. | Not relevant to the topic |
|  | Hayes WN, Tennankore K, Battistella M, Chan CT. Vascular access-related infection in nocturnal home hemodialysis. Hemodial Int. 2014 Apr;18(2):481-7. doi: 10.1111/hdi.12140. | Not relevant to the topic |
|  | Sivananthan G, Menashe L, Halin NJ. Cephalic arch stenosis in dialysis patients: review of clinical relevance, anatomy, current theories on etiology and management. J Vasc Access. 2014 May-Jun;15(3):157-62. doi: 10.5301/jva.5000203. | Not relevant to the topic |
|  | Yan Y, Clark TW, Mondschein JI, Shlansky-Goldberg RD, Dagli MS, Soulen MC, Stavropoulos SW, Sudheendra D, Mantell MP, Cohen RD, Kobrin S, Chittams JL, Trerotola SO. Outcomes of percutaneous interventions in transposed hemodialysis fistulas compared with nontransposed fistulas and grafts. J Vasc Interv Radiol. 2013 Dec;24(12):1765-72; quiz 1773. doi: 10.1016/j.jvir.2013.08.025. | Not relevant to the topic |
|  | Hod T, Desilva RN, Patibandla BK, Vin Y, Brown RS, Goldfarb-Rumyantzev AS. Factors predicting failure of AV "fistula first" policy in the elderly. Hemodial Int. 2014 Apr;18(2):507-15. doi: 10.1111/hdi.12106. | Not relevant to the topic |
|  | Price AJ, Fidelman N, Wilson MW, Kerlan RK. Percutaneous interventions in failing "necklace" hemodialysis grafts: long-term outcomes. J Vasc Interv Radiol. 2014 Feb;25(2):199-205. doi: 10.1016/j.jvir.2013.09.011. | Not relevant to the topic |
|  | Roy-Chaudhury P, Lee TC, Munda R. Predicting dialysis vascular access blood flow and diameter: too much, too little, or just right. Kidney Int. 2013 Dec;84(6):1076-8. doi: 10.1038/ki.2013.307. | Conference abstracts,etc |
|  | Al-Jaishi AA, Oliver MJ, Thomas SM, Lok CE, Zhang JC, Garg AX, Kosa SD, Quinn RR, Moist LM. Patency rates of the arteriovenous fistula for hemodialysis: a systematic review and meta-analysis. Am J Kidney Dis. 2014 Mar;63(3):464-78. doi: 10.1053/j.ajkd.2013.08.023. | Not relevant to the topic |
|  | Dukkipati R, Molnar MZ, Park J, Jing J, Kovesdy CP, Kajani R, Kalantar-Zadeh K. Association of vascular access type with inflammatory marker levels in maintenance hemodialysis patients. Semin Dial. 2014 Jul-Aug;27(4):415-23. doi: 10.1111/sdi.12146. | Not relevant to the topic |
|  | Schier T, Göbel G, Bösmüller C, Gruber I, Tiefenthaler M. Incidence of arteriovenous fistula closure due to high-output cardiac failure in kidney-transplanted patients. Clin Transplant. 2013 Nov-Dec;27(6):858-65. doi: 10.1111/ctr.12248. | Not relevant to the topic |
|  | Wasse H, Huang R, Long Q, Zhao Y, Singapuri S, McKinnon W, Skardasis G, Tangpricha V. Very high-dose cholecalciferol and arteriovenous fistula maturation in ESRD: a randomized, double-blind, placebo-controlled pilot study. J Vasc Access. 2014 Mar-Apr;15(2):88-94. doi: 10.5301/jva.5000187. | Not relevant to the topic |
|  | Kim HR, Kim HK, Oh DJ. Serum osteoprotegerin level is associated with degree of arteriovenous fistula stenosis in patients with hemodialysis. Clin Nephrol. 2013 Nov;80(5):322-7. doi: 10.5414/CN107979. | Not relevant to the topic |
|  | Asano M, Thumma J, Oguchi K, Pisoni RL, Akizawa T, Akiba T, Fukuhara S, Kurokawa K, Ethier J, Saran R, Saito A; J-DOPPS Research Group. Vascular access care and treatment practices associated with outcomes of arteriovenous fistula: international comparisons from the Dialysis Outcomes and Practice Patterns Study. Nephron Clin Pract. 2013;124(1-2):23-30. doi: 10.1159/000353733. | Not relevant to the topic |
|  | Afsar B. The impact of inflammatory factors associated with primary arteriovenous failure. Int J Artif Organs. 2013 Oct;36(10):710-6. doi: 10.5301/ijao.5000235. | Not relevant to the topic |
|  | Field MA, McGrogan DG, Tullet K, Inston NG. Arteriovenous fistula aneurysms in patients with Alport's. J Vasc Access. 2013 Oct-Dec;14(4):397-9. doi: 10.5301/jva.5000167. | Not relevant to the topic |
|  | Jemcov TK. Morphologic and functional vessels characteristics assessed by ultrasonography for prediction of radiocephalic fistula maturation. J Vasc Access. 2013 Oct-Dec;14(4):356-63. doi: 10.5301/jva.5000163. | Not relevant to the topic |
|  | Lee J, Kim YS, Yoon SA, Kim YS, Won YD, Park SC, Jang EJ, Kim YO. Retrospective review of angiography before cannulation of newly created vascular accesses in hemodialysis patients. J Vasc Interv Radiol. 2013 Sep;24(9):1309-15. doi: 10.1016/j.jvir.2013.04.035. | Not relevant to the topic |
|  | Vrakas G, Defigueiredo F, Turner S, Jones C, Taylor J, Calder F. A comparison of the outcomes of one-stage and two-stage brachiobasilic arteriovenous fistulas. J Vasc Surg. 2013 Nov;58(5):1300-4. doi: 10.1016/j.jvs.2013.05.030. | Not relevant to the topic |
|  | Alhassan SU, Adamu B, Abdu A, Aji SA. Outcome and complications of permanent hemodialysis vascular access in Nigerians: a single centre experience. Ann Afr Med. 2013 Apr-Jun;12(2):127-30. doi: 10.4103/1596-3519.112410. | Not relevant to the topic |
|  | Cansu A, Soyturk M, Ozturk MH, Kul S, Pulathan Z, Dinc H. Diagnostic value of color Doppler ultrasonography and MDCT angiography in complications of hemodialysis fistulas and grafts. Eur J Radiol. 2013 Sep;82(9):1436-43. doi: 10.1016/j.ejrad.2013.03.015. | Not relevant to the topic |
|  | Borzumati M, Funaro L, Mancini E, Resentini V, Baroni A. Survival and complications of arteriovenous fistula dialysis access in an elderly population. J Vasc Access. 2013 Oct-Dec;14(4):330-4. doi: 10.5301/jva.5000143. | Not relevant to the topic |
|  | Kim YO, Choi YJ, Kim JI, Kim YS, Kim BS, Park CW, Song HC, Yoon SA, Chang YS, Bang BK. The impact of intima-media thickness of radial artery on early failure of radiocephalic arteriovenous fistula in hemodialysis patients. J Korean Med Sci. 2006 Apr;21(2):284-9. doi: 10.3346/jkms.2006.21.2.284. | Conference abstracts,etc |
|  | Han M, Kim JD, Bae JI, Lee JH, Oh CK, Ahn C, Won JH. Endovascular treatment for immature autogenous arteriovenous fistula. Clin Radiol. 2013 Jun;68(6):e309-15. doi: 10.1016/j.crad.2013.01.005. | Not relevant to the topic |
|  | Wang S, Almehmi A, Asif A. Surgical management of cephalic arch occlusive lesions: are there predictors for outcomes? Semin Dial. 2013 Jul-Aug;26(4):E33-41. doi: 10.1111/sdi.12085. | Not relevant to the topic |
|  | Salmela B, Hartman J, Peltonen S, Albäck A, Lassila R. Thrombophilia and arteriovenous fistula survival in ESRD. Clin J Am Soc Nephrol. 2013 Jun;8(6):962-8. doi: 10.2215/CJN.03860412. | Not relevant to the topic |
|  | Mortamais J, Papillard M, Girouin N, Boutier R, Cougnaud L, Martin X, Badet L, Juillard L, Rouvière O. Endovascular treatment of juxta-anastomotic venous stenoses of forearm radiocephalic fistulas: long-term results and prognostic factors. J Vasc Interv Radiol. 2013 Apr;24(4):558-64; quiz 565. doi: 10.1016/j.jvir.2012.12.004. | Not relevant to the topic |
|  | Caeiro F, Carvalho D, Cruz J, Ribeiro Santos J, Nolasco F. Efficacy of percutaneous transluminal angioplasty on dysfunctional fistulae because of inflow stenosis. J Vasc Access. 2013 Jul-Sep;14(3):231-8. doi: 10.5301/jva.5000129. | Not relevant to the topic |
|  | Steerman SN, Wagner J, Higgins JA, Kim C, Mirza A, Pavela J, Panneton JM, Glickman MH. Outcomes comparison of HeRO and lower extremity arteriovenous grafts in patients with long-standing renal failure. J Vasc Surg. 2013 Mar;57(3):776-83; discussion 782-3. doi: 10.1016/j.jvs.2012.09.040. | Not relevant to the topic |
|  | Peden EK, Leeser DB, Dixon BS, El-Khatib MT, Roy-Chaudhury P, Lawson JH, Menard MT, Dember LM, Glickman MH, Gustafson PN, Blair AT, Magill M, Franano FN, Burke SK. A multi-center, dose-escalation study of human type I pancreatic elastase (PRT-201) administered after arteriovenous fistula creation. J Vasc Access. 2013 Apr-Jun;14(2):143-51. doi: 10.5301/jva.5000125. | Not relevant to the topic |
|  | Rajput A, Rajan DK, Simons ME, Sniderman KW, Jaskolka JD, Beecroft JR, Kachura JR, Tan KT. Venous aneurysms in autogenous hemodialysis fistulas: is there an association with venous outflow stenosis. J Vasc Access. 2013 Apr-Jun;14(2):126-30. doi: 10.5301/jva.5000111. | Not relevant to the topic |
|  | Usta E, Elkrinawi R, Salehi-Gilani S, Adili S, Sonnentag T, Alscher M, Artunc F, Franke U. Risk factors predicting the successful function and use of autogenous arteriovenous fistulae for hemodialysis. Thorac Cardiovasc Surg. 2013 Aug;61(5):438-44. doi: 10.1055/s-0032-1321953. | Not relevant to the topic |
|  | Bolignano D, Rastelli S, Agarwal R, Fliser D, Massy Z, Ortiz A, Wiecek A, Martinez-Castelao A, Covic A, Goldsmith D, Suleymanlar G, Lindholm B, Parati G, Sicari R, Gargani L, Mallamaci F, London G, Zoccali C. Pulmonary hypertension in CKD. Am J Kidney Dis. 2013 Apr;61(4):612-22. doi: 10.1053/j.ajkd.2012.07.029. Epub 2012 Nov 17. Erratum in: Am J Kidney Dis. 2015 Mar;65(3):524. doi: 10.1053/j.ajkd.2014.12.004. | Not relevant to the topic |
|  | Veroux P, Giaquinta A, Tallarita T, Sinagra N, Virgilio C, Zerbo D, Gloviczki P, Veroux M. Primary balloon angioplasty of small (≤2 mm) cephalic veins improves primary patency of arteriovenous fistulae and decreases reintervention rates. J Vasc Surg. 2013 Jan;57(1):131-6. doi: 10.1016/j.jvs.2012.07.047. | Not relevant to the topic |
|  | Stolic R. Most important chronic complications of arteriovenous fistulas for hemodialysis. Med Princ Pract. 2013;22(3):220-8. doi: 10.1159/000343669. | Not relevant to the topic |
|  | Kroon W, Bosboom M, Huberts W, Tordoir J, van de Vosse F. Computational model for estimating the short- and long-term cardiac response to arteriovenous fistula creation for hemodialysis. Med Biol Eng Comput. 2012 Dec;50(12):1289-98. doi: 10.1007/s11517-012-0966-9. | Not relevant to the topic |
|  | Chandra A, Mix D, Varble N. Hemodynamic study of arteriovenous fistulas for hemodialysis access. Vascular. 2013 Feb;21(1):54-62. doi: 10.1258/vasc.2011.201204. | Not relevant to the topic |
|  | Lee JH, Won JH, Oh CK, Jung HA. Clinical significance of upper-arm cephalic vein patency in autogenous radial-cephalic wrist fistulas for hemodialysis. Eur J Vasc Endovasc Surg. 2012 Nov;44(5):514-20. doi: 10.1016/j.ejvs.2012.08.017. | Not relevant to the topic |
|  | Hanko J, Romann A, Taylor P, Copland M, Beaulieu M. Optimizing AVF creation prior to dialysis start: the role of predialysis renal replacement therapy choices. Nephrol Dial Transplant. 2012 Nov;27(11):4205-10. doi: 10.1093/ndt/gfs378. | Not relevant to the topic |
|  | Scholz H. Behandlung von Komplikationen nach Anlage von AV-Gefässzugängen [Treatment of complications after arteriovenous access surgery]. Chirurg. 2012 Sep;83(9):793-800. German. doi: 10.1007/s00104-012-2305-y. | Not relevant to the topic |
|  | Hollenbeck M, Niehuus A, Wozniak G, Hennigs S. Zentralvenöse Katheter als Zugang für die Akut- und Dauerdialyse [Central venous catheters as access for acute and long-term dialysis]. Chirurg. 2012 Sep;83(9):801-8. German. doi: 10.1007/s00104-012-2306-x. | Not relevant to the topic |
|  | Rajabi-Jagahrgh E, Krishnamoorthy MK, Roy-Chaudhury P, Succop P, Wang Y, Choe A, Banerjee RK. Longitudinal assessment of hemodynamic endpoints in predicting arteriovenous fistula maturation. Semin Dial. 2013 Mar-Apr;26(2):208-15. doi: 10.1111/j.1525-139X.2012.01112.x. | Not relevant to the topic |
|  | Wu JH, Zhang DW, Cheng XL, Shi H, Fan YP. Platelet glycoprotein IIb HPA-3 a/b polymorphism is associated with native arteriovenous fistula thrombosis in chronic hemodialysis patients. Ren Fail. 2012;34(8):960-3. doi: 10.3109/0886022X.2012.706865. | Not relevant to the topic |
|  | Kizilisik AT, Kim SB, Nylander WA, Shaffer D. Improvements in dialysis access survival with increasing use of arteriovenous fistulas in a Veterans Administration medical center. Am J Surg. 2004 Nov;188(5):614-6. doi: 10.1016/j.amjsurg. | Conference abstracts,etc |
|  | Alashek WA, McIntyre CW, Taal MW. Vascular access in patients receiving hemodialysis in Libya. J Vasc Access. 2012 Oct-Dec;13(4):468-74. doi: 10.5301/jva.5000089. | Not relevant to the topic |
|  | Shingarev R, Barker-Finkel J, Allon M. Association of hemodialysis central venous catheter use with ipsilateral arteriovenous vascular access survival. Am J Kidney Dis. 2012 Dec;60(6):983-9. doi: 10.1053/j.ajkd.2012.06.014. | Not relevant to the topic |
|  | Khavanin Zadeh M, Gholipour F, Hadipour R. The effect of hemoglobin level on arteriovenous fistula survival in Iranian hemodialysis patients. J Vasc Access. 2008 Apr-Jun;9(2):133-6. | Conference abstracts,etc |
|  | Katsanos K, Karnabatidis D, Kitrou P, Spiliopoulos S, Christeas N, Siablis D. Paclitaxel-coated balloon angioplasty vs. plain balloon dilation for the treatment of failing dialysis access: 6-month interim results from a prospective randomized controlled trial. J Endovasc Ther. 2012 Apr;19(2):263-72. doi: 10.1583/11-3690.1. | Not relevant to the topic |
|  | Fokou M, Teyang A, Ashuntantang G, Kaze F, Eyenga VC, Chichom Mefire A, Angwafo F 3rd. Complications of arteriovenous fistula for hemodialysis: an 8-year study. Ann Vasc Surg. 2012 Jul;26(5):680-4. doi: 10.1016/j.avsg.2011.09.014. | Not relevant to the topic |
|  | Twine CP, Haidermota M, Woolgar JD, Gibbons CP, Davies CG. A scoring system (DISTAL) for predicting failure of snuffbox arteriovenous fistulas. Eur J Vasc Endovasc Surg. 2012 Jul;44(1):88-91. doi: 10.1016/j.ejvs.2012.03.014. | Not relevant to the topic |
|  | Martínez-Gallardo R, Ferreira-Morong F, García-Pino G, Cerezo-Arias I, Hernández-Gallego R, Caravaca F. Congestive heart failure in patients with advanced chronic kidney disease: association with pre-emptive vascular access placement. Nefrologia. 2012;32(2):206-12. English, Spanish. doi: 10.3265/Nefrologia.pre2011.Dec.11223. | Not relevant to the topic |
|  | Poulikakos D, Theti D, Pau V, Banerjee D, Jones D. The impact of arteriovenous fistula creation in pulmonary hypertension: measurement of pulmonary pressures by right heart catheterization in a patient with respiratory failure following arteriovenous fistula creation. Hemodial Int. 2012 Oct;16(4):553-5. doi: 10.1111/j.1542-4758.2012.00674.x. | Conference abstracts,etc |
|  | Solak Y, Caymaz M, Tonbul HZ, Ozbek O, Turkmen K, Gormus N. Effects of secondary amyloidosis on arteriovenous hemodialysis fistula outcomes and intradialytic hypotension: a case-control study. Hemodial Int. 2012 Jul;16(3):401-6. doi: 10.1111/j.1542-4758.2012.00673.x. | Not relevant to the topic |
|  | Lilly MP, Lynch JR, Wish JB, Huff ED, Chen SC, Armistead NC, McClellan WM. Prevalence of arteriovenous fistulas in incident hemodialysis patients: correlation with patient factors that may be associated with maturation failure. Am J Kidney Dis. 2012 Apr;59(4):541-9. doi: 10.1053/j.ajkd.2011.11.038. | Not relevant to the topic |
|  | Suttie SA, Ponnuvelu G, Henderson N, Vint R, Ross R, Tootill R, Howd A, Nagy J, Griffiths GD. Natural history of upper limb arterio-venous fistulae for chronic hemodialysis. J Vasc Access. 2012 Jul-Sep;13(3):332-7. doi: 10.5301/jva.5000050. | Not relevant to the topic |
|  | Fokou M, Ashuntantang G, Teyang A, Kaze F, Chichom Mefire A, Halle MP, Angwafo F 3rd, Takongmo S, Sandmann W. Patients characteristics and outcome of 518 arteriovenous fistulas for hemodialysis in a sub-Saharan African setting. Ann Vasc Surg. 2012 Jul;26(5):674-9. doi: 10.1016/j.avsg.2011.07.019. | Not relevant to the topic |
|  | Fitzgerald JT, Schanzer A, Chin AI, McVicar JP, Perez RV, Troppmann C. Outcomes of upper arm arteriovenous fistulas for maintenance hemodialysis access. Arch Surg. 2004 Feb;139(2):201-8. doi: 10.1001/archsurg.139.2.201. | Conference abstracts,etc |
|  | Hayes WN, Watson AR, Callaghan N, Wright E, Stefanidis CJ; European Pediatric Dialysis Working Group. Vascular access: choice and complications in European paediatric haemodialysis units. Pediatr Nephrol. 2012 Jun;27(6):999-1004. | Not relevant to the topic |
|  | Marchi G. Ipotensione intradialitica? controlliamo anche la fistola! [Intradialytic hypotension? check the fistula!]. G Ital Nefrol. 2011 Nov-Dec;28(6):579. Italian. | Not relevant to the topic |
|  | Shah R, Bhatt UY, Cleef SV, Farley M, Davis A, Swope M, Agarwal AK. Vascular access thrombosis and interventions in patients missing hemodialysis sessions. Clin Nephrol. 2011 Dec;76(6):435-9. doi: 10.5414/cn107086. | Not relevant to the topic |
|  | Sato M, Io H, Tanimoto M, Shimizu Y, Fukui M, Hamada C, Horikoshi S, Tomino Y. Relationship between preoperative radial artery and postoperative arteriovenous fistula blood flow in hemodialysis patients. J Nephrol. 2012 Sep-Oct;25(5):726-31. doi: 10.5301/jn.5000050. | Not relevant to the topic |
|  | Mestres G, Fontseré N, García-Madrid C, Campelos P, Maduell F, Riambau V. Intra-operative factors predicting 1-month arteriovenous fistula thrombosis. J Vasc Access. 2012 Apr-Jun;13(2):193-7. doi: 10.5301/jva.5000021. | Not relevant to the topic |
|  | Wasse H, Huang R, Naqvi N, Smith E, Wang D, Husain A. Inflammation, oxidation and venous neointimal hyperplasia precede vascular injury from AVF creation in CKD patients. J Vasc Access. 2012 Apr-Jun;13(2):168-74. doi: 10.5301/jva.5000024. | Not relevant to the topic |
|  | Heye S, Maleux G, Vaninbroukx J, Claes K, Kuypers D, Oyen R. Factors influencing technical success and outcome of percutaneous balloon angioplasty in de novo native hemodialysis arteriovenous fistulas. Eur J Radiol. 2012 Sep;81(9):2298-303. doi: 10.1016/j.ejrad.2011.09.004. | Not relevant to the topic |
|  | Ayez N, Fioole B, Aarts RA, van den Dorpel MA, Akkersdijk GP, Dinkelman MK, de Smet AA. Secondary interventions in patients with autologous arteriovenous fistulas strongly improve patency rates. J Vasc Surg. 2011 Oct;54(4):1095-9. doi: 10.1016/j.jvs.2011.04.023. | Not relevant to the topic |
|  | Chang TI, Paik J, Greene T, Desai M, Bech F, Cheung AK, Chertow GM. Intradialytic hypotension and vascular access thrombosis. J Am Soc Nephrol. 2011 Aug;22(8):1526-33. doi: 10.1681/ASN.2010101119. | Not relevant to the topic |
|  | Schinstock CA, Albright RC, Williams AW, Dillon JJ, Bergstralh EJ, Jenson BM, McCarthy JT, Nath KA. Outcomes of arteriovenous fistula creation after the Fistula First Initiative. Clin J Am Soc Nephrol. 2011 Aug;6(8):1996-2002. doi: 10.2215/CJN.11251210. | Not relevant to the topic |
|  | Radoui A, Lyoussfi Z, Haddiya I, Skalli Z, El Idrissi R, Rhou H, Ezzaitouni F, Ouzeddoun N, El Mesnaoui A, Bayahia R, Benamar L. Survival of the first arteriovenous fistula in 96 patients on chronic hemodialysis. Ann Vasc Surg. 2011 Jul;25(5):630-3. doi: 10.1016/j.avsg.2010.08.011. | Not relevant to the topic |
|  | Kimball TA, Barz K, Dimond KR, Edwards JM, Nehler MR. Efficiency of the kidney disease outcomes quality initiative guidelines for preemptive vascular access in an academic setting. J Vasc Surg. 2011 Sep;54(3):760-5; discussion 765-6. doi: 10.1016/j.jvs.2011.03.006. | Not relevant to the topic |
|  | Field M, Van Dellen D, Mak D, Winter H, Hamsho A, Mellor S, Inston N. The brachiobasilic arteriovenous fistula: effect of patient variables. J Vasc Access. 2011 Oct-Dec;12(4):325-30. doi: 10.5301/JVA.2011.8349. | Not relevant to the topic |
|  | Afsar B, Elsurer R. The primary arteriovenous fistula failure-a comparison between diabetic and non-diabetic patients: glycemic control matters. Int Urol Nephrol. 2012 Apr;44(2):575-81. doi: 10.1007/s11255-011-9978-x. | Not relevant to the topic |
|  | Bonforte G, Pogliani D, Genovesi S. Sorveglianza della fistola arterovenosa: nuove risposte a un vecchio problema [Surveillance of arteriovenous fistula: new answers to an old problem]. G Ital Nefrol. 2011 Jan-Feb;28(1):48-56. Italian. | Not relevant to the topic |
|  | Jiang SH, Clayton PA, Maguire A, Talaulikar GS. Validation of the measurement of haemodialysis access flow using a haemoglobin dilution test. Blood Purif. 2011;32(1):48-52. doi: 10.1159/000323554. | Not relevant to the topic |
|  | Swindlehurst N, Swindlehurst A, Lumgair H, Rebollo Mesa I, Mamode N, Cacciola R, Macdougall I. Vascular access for hemodialysis in the elderly. J Vasc Surg. 2011 Apr;53(4):1039-43. doi: 10.1016/j.jvs.2010.09.068. | Not relevant to the topic |
|  | Anaya-Ayala JE, Bellows PH, Ismail N, Cheema ZF, Naoum JJ, Bismuth J, Lumsden AB, Reardon MJ, Davies MG, Peden EK. Surgical management of hemodialysis-related central venous occlusive disease: a treatment algorithm. Ann Vasc Surg. 2011 Jan;25(1):108-19. doi: 10.1016/j.avsg.2010.11.002. | Conference abstracts,etc |
|  | Lee T, Ullah A, Allon M, Succop P, El-Khatib M, Munda R, Roy-Chaudhury P. Decreased cumulative access survival in arteriovenous fistulas requiring interventions to promote maturation. Clin J Am Soc Nephrol. 2011 Mar;6(3):575-81. doi: 10.2215/CJN.06630810. | Not relevant to the topic |
|  | Gagliardi GM, Rossi S, Condino F, Mancuso D, Greco F, Tenuta R, Savino O, Bonofiglio R, Domma F, Latorre G. Malnutrition, infection and arteriovenous fistula failure: is there a link? J Vasc Access. 2011 Jan-Mar;12(1):57-62. doi: 10.5301/jva.2010.5831. | Not relevant to the topic |
|  | Paneni F, Gregori M, Ciavarella GM, Sciarretta S, De Biase L, Marino L, Tocci G, Principe F, Domenici A, Luciani R, Punzo G, Menè P, Volpe M. Right ventricular dysfunction in patients with end-stage renal disease. Am J Nephrol. 2010;32(5):432-8. doi: 10.1159/000320755. | Not relevant to the topic |
|  | Bonforte G, Rossi E, Auricchio S, Pogliani D, Mangano S, Mandolfo S, Galli F, Genovesi S. The middle-arm fistula as a valuable surgical approach in patients with end-stage renal disease. J Vasc Surg. 2010 Dec;52(6):1551-6. doi: 10.1016/j.jvs.2010.06.165. | Not relevant to the topic |
|  | Lee ES, Shen Q, Pitts RL, Guo M, Wu MH, Yuan SY. Vein tissue expression of matrix metalloproteinase as biomarker for hemodialysis arteriovenous fistula maturation. Vasc Endovascular Surg. 2010 Nov;44(8):674-9. doi: 10.1177/1538574410377021. | Not relevant to the topic |
|  | Hsiao JF, Chou HH, Hsu LA, Wu LS, Yang CW, Hsu TS, Chang CJ. Vascular changes at the puncture segments of arteriovenous fistula for hemodialysis access. J Vasc Surg. 2010 Sep;52(3):669-73. doi: 10.1016/j.jvs.2010.03.032. | Not relevant to the topic |
|  | Lin CC, Yang WC, Chung MY, Lee PC. Functional polymorphisms in matrix metalloproteinases-1, -3, -9 are associated with arteriovenous fistula patency in hemodialysis patients. Clin J Am Soc Nephrol. 2010 Oct;5(10):1805-14. doi: 10.2215/CJN.01500210. | Not relevant to the topic |
|  | Monroy-Cuadros M, Yilmaz S, Salazar-Bañuelos A, Doig C. Risk factors associated with patency loss of hemodialysis vascular access within 6 months. Clin J Am Soc Nephrol. 2010 Oct;5(10):1787-92. doi: 10.2215/CJN.09441209. | Not relevant to the topic |
|  | Briones L, Diaz Moreno A, Sierre S, Lopez L, Lipsich J, Adragna M. Permanent vascular access survival in children on long-term chronic hemodialysis. Pediatr Nephrol. 2010 Sep;25(9):1731-8. doi: 10.1007/s00467-010-1553-7. | Inconsistent with study population |
|  | Kim HS, Park JW, Chang JH, Yang J, Lee HH, Chung W, Park YH, Kim S. Early vascular access blood flow as a predictor of long-term vascular access patency in incident hemodialysis patients. J Korean Med Sci. 2010 May;25(5):728-33. doi: 10.3346/jkms.2010.25.5.728. | Not relevant to the topic |
|  | Stolić R, Mitrović S, Stolić D, Mitić N. [Early pathohistological changes in dysfunction of arteriovenous fistula for hemodialysis]. Vojnosanit Pregl. 2010 Jan;67(1):65-8. Serbian. doi: 10.2298/vsp1001065s. | Not relevant to the topic |
|  | Lioupis C, Mistry H, Junghans C, Haughey N, Freedman B, Tyrrell M, Valenti D. High brachial artery bifurcation is associated with failure of brachio-cephalic autologous arteriovenous fistulae. J Vasc Access. 2010 Apr-Jun;11(2):132-7. doi: 10.1177/112972981001100209. | Not relevant to the topic |
|  | Ascher E, Hingorani A, Marks N. Duplex scanning-derived access volume flow: novel predictor of success following endovascular repair of failing or nonmaturing arteriovenous fistulae for hemodialysis. Vascular. 2010 Jan-Feb;18(1):9-13. doi: 10.2310/6670.2009.00055. | Not relevant to the topic |
|  | Son HJ, Min SK, Min SI, Park YJ, Ha J, Kim SJ. Evaluation of the efficacy of the forearm basilic vein transposition arteriovenous fistula. J Vasc Surg. 2010 Mar;51(3):667-72. doi: 10.1016/j.jvs.2009.09.048. | Not relevant to the topic |
|  | Bonforte G, Pogliani D, Brenna S, Martinelli D, Bernardi LE, D'Amico M, Mangano S, Rossi E, Genovesi S, Grillo C. Validation of QB stress test as a useful tool in the detection of native arteriovenous fistula stenosis: results after 22 months of follow-up. Nephrol Dial Transplant. 2010 Jun;25(6):1943-9. doi: 10.1093/ndt/gfp725. | Not relevant to the topic |
|  | Bojakowski K, Gorczyca-Wiśniewska E, Szatkowski M, Walecki J, Andziak P. Preoperative ultrasonographic examination of the radial artery and the cephalic vein and risks of dialysis arterio-venous fistula dysfunction. Pol J Radiol. 2010 Jan;75(1):7-12 | Not relevant to the topic |
|  | Kukavica N, Resic H, Sahovic V. Comparison of complications and dialysis adequacy between temporary and permanent tunnelled catheter for haemodialysis. Bosn J Basic Med Sci. 2009 Nov;9(4):265-70. doi: 10.17305/bjbms.2009.2776. | Full text not available |
|  | Conte MS, Nugent HM, Gaccione P, Guleria I, Roy-Chaudhury P, Lawson JH. Multicenter phase I/II trial of the safety of allogeneic endothelial cell implants after the creation of arteriovenous access for hemodialysis use: the V-HEALTH study. J Vasc Surg. 2009 Dec;50(6):1359-68.e1. doi: 10.1016/j.jvs.2009.07.108 | Not relevant to the topic |
|  | Miguel SS, Chow J. Vascular dialysis access flow measurement: early intervention through early detection. J Ren Care. 2009 Dec;35(4):185-91. doi: 10.1111/j.1755-6686.2009.00114.x. | Not relevant to the topic |
|  | Tomilina NA, Gendlin GE, Zhidkova DA, Tronina OA, Fedorova ND. [Left ventricular myocardial hypertrophy after transplantation of the kidney: risk factors and possible regress]. Ter Arkh. 2009;81(8):42-8. Russian. | Not relevant to the topic |
|  | Woo K, Doros G, Ng T, Farber A. Comparison of the efficacy of upper arm transposed arteriovenous fistulae and upper arm prosthetic grafts. J Vasc Surg. 2009 Dec;50(6):1405-11.e1-2. doi: 10.1016/j.jvs.2009.07.090. | Not relevant to the topic |
|  | Malinzak EB, Gan TJ. Regional anesthesia for vascular access surgery. Anesth Analg. 2009 Sep;109(3):976-80. doi: 10.1213/ane.0b013e3181adc208. | Not relevant to the topic |
|  | Beigi AA, Sadeghi AM, Khosravi AR, Karami M, Masoudpour H. Effects of the arteriovenous fistula on pulmonary artery pressure and cardiac output in patients with chronic renal failure. J Vasc Access. 2009 Jul-Sep;10(3):160-6. doi: 10.1177/112972980901000305. | Not relevant to the topic |
|  | Akoh JA. Prosthetic arteriovenous grafts for hemodialysis. J Vasc Access. 2009 Jul-Sep;10(3):137-47. doi: 10.1177/112972980901000301. | Not relevant to the topic |
|  | Soni SS, Nagarik AP, Adikey GK, Raman A. Using continuous renal replacement therapy to manage patients of shock and acute renal failure. J Emerg Trauma Shock. 2009 Jan;2(1):19-22. doi: 10.4103/0974-2700.44678. | Not relevant to the topic |
|  | van Loon MM, Kessels AG, van der Sande FM, Tordoir JH. Cannulation practice patterns in haemodialysis vascular access: predictors for unsuccessful cannulation. J Ren Care. 2009 Jun;35(2):82-9. doi: 10.1111/j.1755-6686.2009.00092.x. | Full text not available |
|  | Malovrh M. Approach to patients with end-stage renal disease who need an arteriovenous fistula. Nephrol Dial Transplant. 2003 Jul;18 Suppl 5:v50-2. doi: 10.1093/ndt/gfg1047. | Full text not available |
|  | Heye S, Maleux G, Claes K, Kuypers D, Oyen R. Stenosis detection in native hemodialysis fistulas with MDCT angiography. AJR Am J Roentgenol. 2009 Apr;192(4):1079-84. doi: 10.2214/AJR.08.1620. | Not relevant to the topic |
|  | Bradbury BD, Chen F, Furniss A, Pisoni RL, Keen M, Mapes D, Krishnan M. Conversion of vascular access type among incident hemodialysis patients: description and association with mortality. Am J Kidney Dis. 2009 May;53(5):804-14. doi: 10.1053/j.ajkd.2008.11.031. | Not relevant to the topic |
|  | Wu CC, Wen SC, Yang CW, Pu SY, Tsai KC, Chen JW. Plasma ADMA predicts restenosis of arteriovenous fistula. J Am Soc Nephrol. 2009 Jan;20(1):213-22. doi: 10.1681/ASN.2008050476. | Not relevant to the topic |
|  | Rooijens PP, Serafino GP, Vroegindeweij D, Dammers R, Yo TI, De Smet AA, Tordoir JH. Multi-slice computed tomographic angiography for stenosis detection in forearm hemodialysis arteriovenous fistulas. J Vasc Access. 2008 Oct-Dec;9(4):278-84. | Not relevant to the topic |
|  | Costa E, Rocha S, Rocha-Pereira P, Castro E, Reis F, Teixeira F, Miranda V, Do Sameiro Faria M, Loureiro A, Quintanilha A, Belo L, Santos-Silva A. Cross-talk between inflammation,coagulation/fibrinolysis and vascular access in hemodialysis patients. J Vasc Access. 2008 Oct-Dec;9(4):248-53. | Not relevant to the topic |
|  | Berman SS, Mendoza B, Westerband A, Quick RC. Predicting arteriovenous fistula maturation with intraoperative blood flow measurements. J Vasc Access. 2008 Oct-Dec;9(4):241-7. | Not relevant to the topic |
|  | Schild AF, Perez E, Gillaspie E, Seaver C, Livingstone J, Thibonnier A. Arteriovenous fistulae vs. arteriovenous grafts: a retrospective review of 1,700 consecutive vascular access cases. J Vasc Access. 2008 Oct-Dec;9(4):231-5. | Not relevant to the topic |
|  | Righetti M, Ferrario G, Serbelloni P, Milani S, Tommasi A. Some old drugs improve late primary patency rate of native arteriovenous fistulas in hemodialysis patients. Ann Vasc Surg. 2009 Jul-Aug;23(4):491-7. doi: 10.1016/j.avsg.2008.08.033. | Not relevant to the topic |
|  | Koksoy C, Demirci RK, Balci D, Solak T, Köse SK. Brachiobasilic versus brachiocephalic arteriovenous fistula: a prospective randomized study. J Vasc Surg. 2009 Jan;49(1):171-177.e5. doi: 10.1016/j.jvs.2008.08.002. | Not relevant to the topic |
|  | Malik J, Tuka V, Kasalova Z, Chytilova E, Slavikova M, Clagett P, Davidson I, Dolmatch B, Nichols D, Gallieni M. Understanding the dialysis access steal syndrome. A review of the etiologies, diagnosis, prevention and treatment strategies. J Vasc Access. 2008 Jul-Sep;9(3):155-66. | Not relevant to the topic |
|  | López Gómez JM. Avances en hemodiálisis clínica: accesos vasculares y pacientes de edad avanzada [Updates in clinical hemodialysis: vascular accessess and elderly patients]. Nefrologia. 2008;28 Suppl 5:67-70. Spanish. | Not relevant to the topic |
|  | Diskin CJ, Stokes TJ, Dansby LM, Radcliff L, Carter TB. Understanding the pathophysiology of hemodialysis access problems as a prelude to developing innovative therapies. Nat Clin Pract Nephrol. 2008 Nov;4(11):628-38. doi: 10.1038/ncpneph0947. | Not relevant to the topic |
|  | Lazarides MK, Georgiadis GS, Papasideris CP, Trellopoulos G, Tzilalis VD. Transposed brachial-basilic arteriovenous fistulas versus prosthetic upper limb grafts: a meta-analysis. Eur J Vasc Endovasc Surg. 2008 Nov;36(5):597-601. doi: 10.1016/j.ejvs.2008.07.008. | Not relevant to the topic |
|  | Salimi J, Razeghi E, Karjalian H, Meysamie A, Dahhaz M, Dadmehr M. Predicting hemodialysis access failure with the measurement of dialysis access recirculation. Saudi J Kidney Dis Transpl. 2008 Sep;19(5):781-4. | Not relevant to the topic |
|  | Zohny SF, Abd el-Fattah M. Evaluation of circulating vascular endothelial growth factor and soluble adhesion molecules as reliable predictors of native arteriovenous fistula thrombosis in chronic hemodialysis patients. Clin Biochem. 2008 Oct;41(14-15):1175-80. doi: 10.1016/j.clinbiochem.2008.07.006. | Not relevant to the topic |
|  | Gorgi Y, Sfar I, Ben Aabdallah T, Aouadi H, Abderrahim E, Bardi R, Jendoubi-Ayed S, Ayed K. Human platelet antigens polymorphisms and susceptibility of thrombosis in hemodialysis patients. Hemodial Int. 2008 Jul;12(3):331-5. doi: 10.1111/j.1542-4758.2008.00277.x. | Not relevant to the topic |
|  | Frankenfield DL, Neu AM, Warady BA, Watkins SL, Friedman AL, Fivush BA. Adolescent hemodialysis: results of the 2000 ESRD Clinical Performance Measures Project. Pediatr Nephrol. 2002 Jan;17(1):10-5. doi: 10.1007/s004670200002. | Inconsistent with study population |
|  | Hasegawa T, Elder SJ, Bragg-Gresham JL, Pisoni RL, Yamazaki S, Akizawa T, Jadoul M, Hugh RC, Port FK, Fukuhara S. Consistent aspirin use associated with improved arteriovenous fistula survival among incident hemodialysis patients in the dialysis outcomes and practice patterns study. Clin J Am Soc Nephrol. 2008 Sep;3(5):1373-8. doi: 10.2215/CJN.00130108 | Not relevant to the topic |
|  | Kadiroglu AK, Sit D, Kayabasi H, Yilmaz Z, Yilmaz E. The association of demographic, clinical, and thrombophilic factors with the failure of arteriovenous fistula among hemodialysis patients. Saudi Med J. 2008 Jun;29(6):888-91. | Not relevant to the topic |
|  | Qasaimeh GR, El Qaderi S, Al Omari G, Al Badadweh M. Vascular access infection among hemodialysis patients in Northern Jordan: incidence and risk factors. South Med J. 2008 May;101(5):508-12. doi: 10.1097/SMJ.0b013e31816c0155. | Not relevant to the topic |
|  | Chan MR, Young HN, Becker YT, Yevzlin AS. Obesity as a predictor of vascular access outcomes: analysis of the USRDS DMMS Wave II study. Semin Dial. 2008 May-Jun;21(3):274-9. doi: 10.1111/j.1525-139X.2008.00434.x. | Not relevant to the topic |
|  | Kapun S, Zibar L. Kako je moguće predvidjeti trombozu arterijskovenske fistule? [How to predict thrombosis of arteriovenous fistule?]. Acta Med Croatica. 2008 Feb;62(1):9-13. | Not relevant to the topic |
|  | Huijbregts HJ, Bots ML, Wittens CH, Schrama YC, Moll FL, Blankestijn PJ; CIMINO study group. Hemodialysis arteriovenous fistula patency revisited: results of a prospective, multicenter initiative. Clin J Am Soc Nephrol. 2008 May;3(3):714-9. doi: 10.2215/CJN.02950707. | Not relevant to the topic |
|  | Gundevia Z, Whalley H, Ferring M, Claridge M, Smith S, Wilmink T. Effect of operating surgeon on outcome of arteriovenous fistula formation. Eur J Vasc Endovasc Surg. 2008 May;35(5):614-8. doi: 10.1016/j.ejvs.2007.11.018. | Not relevant to the topic |
|  | Campos RP, Chula DC, Perreto S, Riella MC, do Nascimento MM. Accuracy of physical examination and intra-access pressure in the detection of stenosis in hemodialysis arteriovenous fistula. Semin Dial. 2008 May-Jun;21(3):269-73. doi: 10.1111/j.1525-139X.2007.00419.x. | Not relevant to the topic |
|  | Budu-Grajdeanu P, Schugart RC, Friedman A, Valentine C, Agarwal AK, Rovin BH. A mathematical model of venous neointimal hyperplasia formation. Theor Biol Med Model. 2008 Jan 23;5:2. doi: 10.1186/1742-4682-5-2. | Not relevant to the topic |
|  | Kian K, Asif A. Cephalic arch stenosis. Semin Dial. 2008 Jan-Feb;21(1):78-82. doi: 10.1111/j.1525-139X.2007.00387.x. | Not relevant to the topic |
|  | Marcus RJ, Marcus DA, Sureshkumar KK, Hussain SM, McGill RL. Gender differences in vascular access in hemodialysis patients in the United States: developing strategies for improving access outcome. Gend Med. 2007 Sep;4(3):193-204. doi: 10.1016/s1550-8579(07)80040-4 | Not relevant to the topic |
|  | Chan MR, Sanchez RJ, Young HN, Yevzlin AS. Vascular access outcomes in the elderly hemodialysis population: A USRDS study. Semin Dial. 2007 Nov-Dec;20(6):606-10. doi: 10.1111/j.1525-139X.2007.00370.x. | Not relevant to the topic |
|  | Crikis S, Lee D, Brooks M, Power DA, Ierino FL, Levidiotis V. Predictors of early dialysis vascular-access failure after thrombolysis. Am J Nephrol. 2008;28(2):181-9. doi: 10.1159/000110086. | Not relevant to the topic |
|  | Basile C, Lomonte C, Vernaglione L, Casucci F, Antonelli M, Losurdo N. The relationship between the flow of arteriovenous fistula and cardiac output in haemodialysis patients. Nephrol Dial Transplant. 2008 Jan;23(1):282-7. doi: 10.1093/ndt/gfm549. | Not relevant to the topic |
|  | Liu JH, Lin PW, Liu YL, Lin HH, Huang CC. Comparison of classical and non-classical cardiovascular risk factors influencing the patency of native arteriovenous fistulas after percutaneous transluminal angioplasty therapy among haemodialysis patients. Postgrad Med J. 2007 Aug;83(982):547-51. doi: 10.1136/pgmj.2006.054908. | Not relevant to the topic |
|  | Ravani P, Marcelli D, Pecchini P, Malberti F. Early failure rates of arterovenous fistulas for hemodialysis: evaluation of six-year activity. J Vasc Access. 2001 Oct-Dec;2(4):154-60. doi: 10.1177/112972980100200405. | Not relevant to the topic |
|  | Bilgic A, Ozdemir FN, Bayraktar N, Karakus S, Sasak G, Arat Z, Sezer S, Haberal M. Soluble endothelial protein C receptor: influence on arteriovenous fistula thrombosis development in hemodialysis patients. Am J Nephrol. 2007;27(4):366-72. doi: 10.1159/000103911. | Not relevant to the topic |
|  | Serati AR, Roozbeh J, Sagheb MM. Serum LDL levels are a major prognostic factor for arteriovenous fistula thrombosis (AVFT) in hemodialysis patients. J Vasc Access. 2007 Apr-Jun;8(2):109-14. | Not relevant to the topic |
|  | Ravani P, Spergel LM, Asif A, Roy-Chaudhury P, Besarab A. Clinical epidemiology of arteriovenous fistula in 2007. J Nephrol. 2007 Mar-Apr;20(2):141-9. | Not relevant to the topic |
|  | Seyahi N, Altiparmak MR, Tascilar K, Pekpak M, Serdengecti K, Erek E. Ultrasonographic maturation of native arteriovenous fistulae: a follow-up study. Ren Fail. 2007;29(4):481-6. doi: 10.1080/08860220701278026. | Not relevant to the topic |
|  | Planken RN, Tordoir JH, Duijm LE, de Haan MW, Leiner T. Current techniques for assessment of upper extremity vasculature prior to hemodialysis vascular access creation. Eur Radiol. 2007 Nov;17(11):3001-11. doi: 10.1007/s00330-007-0662-6. | Not relevant to the topic |
|  | Huijbregts HJ, Bots ML, Moll FL, Blankestijn PJ; CIMINO members. Hospital specific aspects predominantly determine primary failure of hemodialysis arteriovenous fistulas. J Vasc Surg. 2007 May;45(5):962-7. doi: 10.1016/j.jvs.2007.01.014. | Not relevant to the topic |
|  | Moini M, Rasouli MR, Rayatzadeh H. Creation of permanent hemodialysis vascular access in patients with failed arteriovenous fistula. Saudi Med J. 2007 Feb;28(2):213-5. | Not relevant to the topic |
|  | Lazarides MK, Georgiadis GS, Antoniou GA, Staramos DN. A meta-analysis of dialysis access outcome in elderly patients. J Vasc Surg. 2007 Feb;45(2):420-426. doi: 10.1016/j.jvs.2006.10.035. | Not relevant to the topic |
|  | Wasse H, Speckman RA, Frankenfield DL, Rocco MV, McClellan WM. Predictors of delayed transition from central venous catheter use to permanent vascular access among ESRD patients. Am J Kidney Dis. 2007 Feb;49(2):276-83. doi: 10.1053/j.ajkd.2006.11.030. | Not relevant to the topic |
|  | Gruss E, Portolés J, Jiménez P, Hernández T, Rueda JA, del Cerro J, Lasala M, Tato A, Gago MC, Martínez S, Velayos P. Seguimiento prospectivo del acceso vascular en hemodialisis mediante un equipo multidisciplinar [Prospective monitoring of vascular access in hemodialysis by means of a multidisciplinary team]. Nefrologia. 2006;26(6):703-10. Spanish. | Not relevant to the topic |
|  | Jushinskis J, Bicans J, Suhorukov V, Trushkov S, Rozental R. Vascular access outcomes as a predictor of development of chronic allograft nephropathy. Transplant Proc. 2006 Oct;38(8):2657-8. doi: 10.1016/j.transproceed.2006.08.189. | Not relevant to the topic |
|  | Wali MA, Eid RA, Dewan M, Al-Homrany MA. Pre-existing histopathological changes in the cephalic vein of renal failure patients before arterio-venous fistula (AVF) construction. Ann Thorac Cardiovasc Surg. 2006 Oct;12(5):341-8. | Not relevant to the topic |
|  | Parmar J, Aslam M, Standfield N. Pre-operative radial arterial diameter predicts early failure of arteriovenous fistula (AVF) for haemodialysis. Eur J Vasc Endovasc Surg. 2007 Jan;33(1):113-5. doi: 10.1016/j.ejvs.2006.09.001. | Not relevant to the topic |
|  | Lok CE, Allon M, Moist L, Oliver MJ, Shah H, Zimmerman D. Risk equation determining unsuccessful cannulation events and failure to maturation in arteriovenous fistulas (REDUCE FTM I). J Am Soc Nephrol. 2006 Nov;17(11):3204-12. doi: 10.1681/ASN.2006030190. | Not relevant to the topic |
|  | Wijnen E, Planken N, Keuter X, Kooman JP, Tordoir JH, de Haan MW, Leunissen KM, van der Sande F. Impact of a quality improvement programme based on vascular access flow monitoring on costs, access occlusion and access failure. Nephrol Dial Transplant. 2006 Dec;21(12):3514-9. doi: 10.1093/ndt/gfl424. | Not relevant to the topic |
|  | Morena M, Bosc JY, Jaussent I, Dupuy AM, Terrier N, Leray-Moragues H, Flavier JL, Maurice F, Delcourt C, Cristol JP, Canaud B. The role of mineral metabolism and inflammation on dialysis vascular access failure. J Vasc Access. 2006 Apr-Jun;7(2):77-82. doi: 10.1177/112972980600700207. | Not relevant to the topic |
|  | Polkinghorne KR, Lau KK, Saunder A, Atkins RC, Kerr PG. Does monthly native arteriovenous fistula blood-flow surveillance detect significant stenosis--a randomized controlled trial. Nephrol Dial Transplant. 2006 Sep;21(9):2498-506. doi: 10.1093/ndt/gfl242 | Not relevant to the topic |
|  | Salahi H, Fazelzadeh A, Mehdizadeh A, Razmkon A, Malek-Hosseini SA. Complications of arteriovenous fistula in dialysis patients. Transplant Proc. 2006 Jun;38(5):1261-4. doi: 10.1016/j.transproceed.2006.02.066. | Not relevant to the topic |
|  | Tonelli M, Klarenbach S, Jindal K, Manns B; Alberta Kidney Disease Network. Economic implications of screening strategies in arteriovenous fistulae. Kidney Int. 2006 Jun;69(12):2219-26. doi: 10.1038/sj.ki.5000151. | Not relevant to the topic |
|  | Lee T, Barker J, Allon M. Needle infiltration of arteriovenous fistulae in hemodialysis: risk factors and consequences. Am J Kidney Dis. 2006 Jun;47(6):1020-6. doi: 10.1053/j.ajkd.2006.02.181. | Not relevant to the topic |
|  | van der Linden J, Lameris TW, van den Meiracker AH, de Smet AA, Blankestijn PJ, van den Dorpel MA. Forearm venous distensibility predicts successful arteriovenous fistula. Am J Kidney Dis. 2006 Jun;47(6):1013-9. doi: 10.1053/j.ajkd.2006.01.033. | Not relevant to the topic |
|  | Heard KA, Russell TA. Access thrombosis, hospitalization, and hematocrit level in hemodialysis patients. Nephrol Nurs J. 2000 Dec;27(6):607-11. | Not relevant to the topic |
|  | Ohira S, Kon T, Imura T. Evaluation of primary failure in native AV-fistulae (early fistula failure). Hemodial Int. 2006 Apr;10(2):173-9. doi: 10.1111/j.1542-4758.2006.00091.x. | Not relevant to the topic |
|  | Woo K, Farber A, Doros G, Killeen K, Kohanzadeh S. Evaluation of the efficacy of the transposed upper arm arteriovenous fistula: a single institutional review of 190 basilic and cephalic vein transposition procedures. J Vasc Surg. 2007 Jul;46(1):94-99; discussion 100. doi: 10.1016/j.jvs.2007.02.057. | Conference abstracts,etc |
|  | Planken RN, Keuter XH, Hoeks AP, Kooman JP, van der Sande FM, Kessels AG, Leiner T, Tordoir JH. Diameter measurements of the forearm cephalic vein prior to vascular access creation in end-stage renal disease patients: graduated pressure cuff versus tourniquet vessel dilatation. Nephrol Dial Transplant. 2006 Mar;21(3):802-6. doi: 10.1093/ndt/gfi340. | Not relevant to the topic |
|  | Huang HL, Chen CC, Chang SH, Hung KC, Hsieh IC, Chang HJ, Wen MS, Fang JT. Combination of duplex ultrasound-guided manual declotting and percutaneous transluminal angioplasty in thrombosed native dialysis fistulas. Ren Fail. 2005;27(6):713-9. doi: 10.1080/08860220500242967 | Not relevant to the topic |
|  | Ku YM, Kim YO, Kim JI, Choi YJ, Yoon SA, Kim YS, Song SW, Yang CW, Kim YS, Chang YS, Bang BK. Ultrasonographic measurement of intima-media thickness of radial artery in pre-dialysis uraemic patients: comparison with histological examination. Nephrol Dial Transplant. 2006 Mar;21(3):715-20. doi: 10.1093/ndt/gfi214. | Not relevant to the topic |
|  | Doelman C, Duijm LE, Liem YS, Froger CL, Tielbeek AV, Donkers-van Rossum AB, Cuypers PW, Douwes-Draaijer P, Buth J, van den Bosch HC. Stenosis detection in failing hemodialysis access fistulas and grafts: comparison of color Doppler ultrasonography, contrast-enhanced magnetic resonance angiography, and digital subtraction angiography. J Vasc Surg. 2005 Oct;42(4):739-46. doi: 10.1016/j.jvs.2005.06.006. | Conference abstracts,etc |
|  | Ozdemir FN, Akcay A, Bilgic A, Akgul A, Arat Z, Haberal M. Effects of smoking and blood eosinophil count on the development of arteriovenous fistulae thrombosis in hemodialysis patients. Transplant Proc. 2005 Sep;37(7):2918-21. doi: 10.1016/j.transproceed.2005.07.011. | Not relevant to the topic |
|  | Ernandez T, Saudan P, Berney T, Merminod T, Bednarkiewicz M, Martin PY. Risk factors for early failure of native arteriovenous fistulas. Nephron Clin Pract. 2005;101(1):c39-44. doi: 10.1159/000085710. | Not relevant to the topic |
|  | Treacy PJ, Ragg JL, Snelling P, Lawton P, Lammi H. Prediction of failure of native arteriovenous fistulas using 'on-line' fistula flow measurements. Nephrology (Carlton). 2005 Apr;10(2):136-41. doi: 10.1111/j.1440-1797.2005.00380.x. | Not relevant to the topic |
|  | Koseoglu K, Akar H, Cildag B, Ozsunar Y, Gayret P. Resistive index measurement of native hemodialysis arteriovenous fistula feeding artery as a predictor for fistula dysfunction. ASAIO J. 2004 Nov-Dec;50(6):577-80. doi: 10.1097/01.mat.0000145052.75525.77. | Not relevant to the topic |
|  | Paulson WD. Access monitoring does not really improve outcomes. Blood Purif. 2005;23(1):50-6. doi: 10.1159/000082011. | Not relevant to the topic |
|  | Froger CL, Duijm LE, Liem YS, Tielbeek AV, Donkers-van Rossum AB, Douwes-Draaijer P, Cuypers PW, Buth J, van den Bosch HC. Stenosis detection with MR angiography and digital subtraction angiography in dysfunctional hemodialysis access fistulas and grafts. Radiology. 2005 Jan;234(1):284-91. doi: 10.1148/radiol.2341031859. | Not relevant to the topic |
|  | Wu JX, Lin CH, Du CY, et al."Estimation of arteriovenous fistula stenosis by FPGA based Doppler flow imaging system," 2015 IEEE International Ultrasonics Symposium (IUS), Taipei, Taiwan, 2015, pp. 1-4, doi: 10.1109/ULTSYM.2015.0328. | Not relevant to the topic |
|  | Maya ID, Oser R, Saddekni S, Barker J, Allon M. Vascular access stenosis: comparison of arteriovenous grafts and fistulas. Am J Kidney Dis. 2004 Nov;44(5):859-65. | Not relevant to the topic |
|  | Schild AF, Prieto J, Glenn M, Livingstone J, Alfieri K, Raines J. Maturation and failure rates in a large series of arteriovenous dialysis access fistulas. Vasc Endovascular Surg. 2004 Sep-Oct;38(5):449-53. doi: 10.1177/153857440403800509. | Not relevant to the topic |
|  | Stein G, Fünfstück R, Schiel R. Diabetes mellitus and dialysis. Minerva Urol Nefrol. 2004 Sep;56(3):289-303. | Not relevant to the topic |
|  | Chang CF, Kuo BI, Chen TL, Yang WC, Lee SD, Lin CC. Infective endocarditis in maintenance hemodialysis patients: fifteen years' experience in one medical center. J Nephrol. 2004 Mar-Apr;17(2):228-35. | Not relevant to the topic |
|  | Tonelli M, Hirsch DJ, Chan CT, Marryatt J, Mossop P, Wile C, Jindal K. Factors associated with access blood flow in native vessel arteriovenous fistulae. Nephrol Dial Transplant. 2004 Oct;19(10):2559-63. doi: 10.1093/ndt/gfh406. | Not relevant to the topic |
|  | Lee PY, Ng W, Chen WH. Concomitant coronary and subclavian steal caused by ipsilateral subclavian artery stenosis and arteriovenous fistula in a hemodialysis patient. Catheter Cardiovasc Interv. 2004 Jun;62(2):244-8. doi: 10.1002/ccd.20055. | Conference abstracts,etc |
|  | Jamshid R, Reza SA, Abbas G, Raha A. Incidence of arteriovenous thrombosis and the role of anticardiolipin antibodies in hemodialysis patients. Int Urol Nephrol. 2003;35(2):275-82. doi: 10.1023/b:urol.0000020354.61227.40. | Not relevant to the topic |
|  | Basile C, Ruggieri G, Vernaglione L, Montanaro A, Giordano R. The natural history of autogenous radio-cephalic wrist arteriovenous fistulas of haemodialysis patients: a prospective observational study. Nephrol Dial Transplant. 2004 May;19(5):1231-6. doi: 10.1093/ndt/gfh073. | Not relevant to the topic |
|  | Sprouse LR 2nd, Lesar CJ, Meier GH 3rd, Parent FN, Demasi RJ, Gayle RG, Marcinzyck MJ, Glickman MH, Shah RM, McEnroe CS, Fogle MA, Stokes GK, Colonna JO. Percutaneous treatment of symptomatic central venous stenosis [corrected]. J Vasc Surg. 2004 Mar;39(3):578-82. doi: 10.1016/j.jvs.2003.09.034. | Not relevant to the topic |
|  | Abbott KC, Trespalacios FC, Agodoa LY. Arteriovenous fistula use and heart disease in long-term elderly hemodialysis patients: analysis of United States Renal Data System Dialysis Morbidity and Mortality Wave II. J Nephrol. 2003 Nov-Dec;16(6):822-30. | Not relevant to the topic |
|  | Ravani P, Brunori G, Mandolfo S, Cancarini G, Imbasciati E, Marcelli D, Malberti F. Cardiovascular comorbidity and late referral impact arteriovenous fistula survival: a prospective multicenter study. J Am Soc Nephrol. 2004 Jan;15(1):204-9. doi: 10.1097/01.asn.0000103870.31606.90. | Not relevant to the topic |
|  | Tonelli M, Jhangri GS, Hirsch DJ, Marryatt J, Mossop P, Wile C, Jindal KK. Best threshold for diagnosis of stenosis or thrombosis within six months of access flow measurement in arteriovenous fistulae. J Am Soc Nephrol. 2003 Dec;14(12):3264-9. doi: 10.1097/01.asn.0000099381.98940.2e. | Not relevant to the topic |
|  | Jüngling A, Bunge N, König M, Holzgreve A. Erleichtert die sonographische Planung die Anlage arteriovenöser Fisteln als Dialysezugang? [Impact of ultrasonographic vascular mapping on constructing autogenous arteriovenous fistulas for permanent hemodialysis access?]. Zentralbl Chir. 2003 Sep;128(9):762-7. German. doi: 10.1055/s-2003-42756. | Not relevant to the topic |
|  | Patel ST, Hughes J, Mills JL Sr. Failure of arteriovenous fistula maturation: an unintended consequence of exceeding dialysis outcome quality Initiative guidelines for hemodialysis access. J Vasc Surg. 2003 Sep;38(3):439-45; discussion 445. doi: 10.1016/s0741-5214(03)00732-8. | Not relevant to the topic |
|  | Tessitore N, Bedogna V, Gammaro L, Lipari G, Poli A, Baggio E, Firpo M, Morana G, Mansueto G, Maschio G. Diagnostic accuracy of ultrasound dilution access blood flow measurement in detecting stenosis and predicting thrombosis in native forearm arteriovenous fistulae for hemodialysis. Am J Kidney Dis. 2003 Aug;42(2):331-41. doi: 10.1016/s0272-6386(03)00659-0. | Not relevant to the topic |
|  | Grandaliano G, Teutonico A, Allegretti A, Losappio R, Mancini A, Gesualdo L, Schena FP, Pertosa G. The role of hyperparathyroidism, erythropoietin therapy, and CMV infection in the failure of arteriovenous fistula in hemodialysis. Kidney Int. 2003 Aug;64(2):715-9. doi: 10.1046/j.1523-1755.2003.00120.x. | Not relevant to the topic |
|  | Lok CE, Bhola C, Croxford R, Richardson RM. Reducing vascular access morbidity: a comparative trial of two vascular access monitoring strategies. Nephrol Dial Transplant. 2003 Jun;18(6):1174-80. doi: 10.1093/ndt/gfg122. | Not relevant to the topic |
|  | Fukasawa M, Matsushita K, Kamiyama M, Mikami Y, Araki I, Yamagata Z, Takeda M. The methylentetrahydrofolate reductase C677T point mutation is a risk factor for vascular access thrombosis in hemodialysis patients. Am J Kidney Dis. 2003 Mar;41(3):637-42. doi: 10.1053/ajkd.2003.50125. | Not relevant to the topic |
|  | Kim YO, Song HC, Yoon SA, Yang CW, Kim NI, Choi YJ, Lee EJ, Kim WY, Chang YS, Bang BK. Preexisting intimal hyperplasia of radial artery is associated with early failure of radiocephalic arteriovenous fistula in hemodialysis patients. Am J Kidney Dis. 2003 Feb;41(2):422-8. doi: 10.1053/ajkd.2003.50051. | Not relevant to the topic |
|  | Planken RN, Tordoir JH, Dammers R, de Haan MW, Oei TK, van der Sande FM, van Engelshoven JM, Leiner T. Stenosis detection in forearm hemodialysis arteriovenous fistulae by multiphase contrast-enhanced magnetic resonance angiography: preliminary experience. J Magn Reson Imaging. 2003 Jan;17(1):54-64. doi: 10.1002/jmri.10225. | Not relevant to the topic |
|  | Polkinghorne KR, Atkins RC, Kerr PG. Native arteriovenous fistula blood flow and resistance during hemodialysis. Am J Kidney Dis. 2003 Jan;41(1):132-9. doi: 10.1053/ajkd.2003.50032. | Not relevant to the topic |
|  | Patard JJ, Bensalah K, Lucas A, Rodriguez A, Manunta A, Rivalan J, Le Pogamp P, Lobel B, Guillé F. Management of vascular access for hemodialysis after successful kidney transplantation. Scand J Urol Nephrol. 2002;36(5):373-6. doi: 10.1080/003655902320783890. | Not relevant to the topic |
|  | Ravani P, Marcelli D, Malberti F. Vascular access surgery managed by renal physicians: the choice of native arteriovenous fistulas for hemodialysis. Am J Kidney Dis. 2002 Dec;40(6):1264-76. doi: 10.1053/ajkd.2002.36897. | Not relevant to the topic |
|  | Frinak S, Zasuwa G, Dunfee T, Besarab A, Yee J. Dynamic venous access pressure ratio test for hemodialysis access monitoring. Am J Kidney Dis. 2002 Oct;40(4):760-8. doi: 10.1053/ajkd.2002.35687. | Not relevant to the topic |
|  | Brimble KS, Rabbat ChG, Treleaven DJ, Ingram AJ. Utility of ultrasonographic venous assessment prior to forearm arteriovenous fistula creation. Clin Nephrol. 2002 Aug;58(2):122-7. doi: 10.5414/cnp58122. | Not relevant to the topic |
|  | Huber TS, Ozaki CK, Flynn TC, Lee WA, Berceli SA, Hirneise CM, Carlton LM, Carter JW, Ross EA, Seeger JM. Prospective validation of an algorithm to maximize native arteriovenous fistulae for chronic hemodialysis access. J Vasc Surg. 2002 Sep;36(3):452-9. doi: 10.1067/mva.2002.127342. | Not relevant to the topic |
|  | Oncevski A, Dejanov P, Gerasimovska V, Polenakovic MH. Approach to vascular access for hemodialysis: experiences from the Republic of Macedonia. Int J Artif Organs. 2002 May;25(5):354-64. doi: 10.1177/039139880202500503. | Not relevant to the topic |
|  | Hojs R, Gorenjak M, Ekart R, Dvorsak B, Pecovnik-Balon B. Homocysteine and vascular access thrombosis in hemodialysis patients. Ren Fail. 2002 Mar;24(2):215-22. doi: 10.1081/jdi-120004098. | Not relevant to the topic |
|  | Malovrh M. Native arteriovenous fistula: preoperative evaluation. Am J Kidney Dis. 2002 Jun;39(6):1218-25. doi: 10.1053/ajkd.2002.33394. | Not relevant to the topic |
|  | Zeebregts C, van den Dungen J, Bolt A, Franssen C, Verhoeven E, van Schilfgaarde R. Factors predictive of failure of Brescia-Cimino arteriovenous fistulas. Eur J Surg. 2002;168(1):29-36. doi: 10.1080/110241502317307544. | Not relevant to the topic |
|  | Clark TW, Hirsch DA, Jindal KJ, Veugelers PJ, LeBlanc J. Outcome and prognostic factors of restenosis after percutaneous treatment of native hemodialysis fistulas. J Vasc Interv Radiol. 2002 Jan;13(1):51-9. doi: 10.1016/s1051-0443(07)60009-8. | Not relevant to the topic |
|  | Brimble KS, Rabbat CG, Schiff D, Ingram AJ. The clinical utility of Doppler ultrasound prior to arteriovenous fistula creation. Semin Dial. 2001 Sep-Oct;14(5):314-7. doi: 10.1046/j.1525-139x.2001.00077.x. | Not relevant to the topic |
|  | Barril G, Besada E, Cirugeda A, Perpen AF, Selgas R. Hemodialysis vascular assessment by an ultrasound dilution method (transonic) in patients older than 65 years. Int Urol Nephrol. 2001;32(3):459-62. doi: 10.1023/a:1017534317535. | Not relevant to the topic |
|  | Kim YO, Yang CW, Yoon SA, Chun KA, Kim NI, Park JS, Kim BS, Kim YS, Chang YS, Bang BK. Access blood flow as a predictor of early failures of native arteriovenous fistulas in hemodialysis patients. Am J Nephrol. 2001 May-Jun;21(3):221-5. doi: 10.1159/000046251. | Not relevant to the topic |
|  | Goldstein SL, Allsteadt A. Ultrasound dilution evaluation of pediatric hemodialysis vascular access. Kidney Int. 2001 Jun;59(6):2357-60. doi: 10.1046/j.1523-1755.2001.00753.x. PMID: 11380840. | Not relevant to the topic |
|  | Konner K. Increasing the proportion of diabetics with AV fistulas. Semin Dial. 2001 Jan-Feb;14(1):1-4. doi: 10.1046/j.1525-139x.2001.00001.x. | Not relevant to the topic |
|  | Schwab SJ, Oliver MJ, Suhocki P, McCann R. Hemodialysis arteriovenous access: detection of stenosis and response to treatment by vascular access blood flow. Kidney Int. 2001 Jan;59(1):358-62. doi: 10.1046/j.1523-1755.2001.00498.x. | Not relevant to the topic |
|  | Goff CD, Sato DT, Bloch PH, DeMasi RJ, Gregory RT, Gayle RG, Parent FN, Meier GH, Wheeler JR. Steal syndrome complicating hemodialysis access procedures: can it be predicted? Ann Vasc Surg. 2000 Mar;14(2):138-44. doi: 10.1007/s100169910025. | Not relevant to the topic |
|  | Valeri A, Joseph R, Radhakrishnan J. A large prospective survey of anti-cardiolipin antibodies in chronic hemodialysis patients. Clin Nephrol. 1999 Feb;51(2):116-21. | Not relevant to the topic |
|  | Song IS, Yang WS, Kim SB, Lee JH, Kwon TW, Park JS. Association of plasma fibrinogen concentration with vascular access failure in hemodialysis patients. Nephrol Dial Transplant. 1999 Jan;14(1):137-41. doi: 10.1093/ndt/14.1.137. | Not relevant to the topic |
|  | Obialo CI, Robinson T, Brathwaite M. Hemodialysis vascular access: variable thrombus-free survival in three subpopulations of black patients. Am J Kidney Dis. 1998 Feb;31(2):250-6. doi: 10.1053/ajkd.1998.v31.pm9469495. | Inconsistent with study population |
|  | Hodges TC, Fillinger MF, Zwolak RM, Walsh DB, Bech F, Cronenwett JL. Longitudinal comparison of dialysis access methods: risk factors for failure. J Vasc Surg. 1997 Dec;26(6):1009-19. doi: 10.1016/s0741-5214(97)70014-4. | Not relevant to the topic |
|  | May RE, Himmelfarb J, Yenicesu M, Knights S, Ikizler TA, Schulman G, Hernanz-Schulman M, Shyr Y, Hakim RM. Predictive measures of vascular access thrombosis: a prospective study. Kidney Int. 1997 Dec;52(6):1656-62. doi: 10.1038/ki.1997.499. | Inconsistent with study population |
|  | Culp K, Taylor L, Hulme PA. Geriatric hemodialysis patients: a comparative study of vascular access. ANNA J. 1996 Dec;23(6):583-90, 622; discussion 591-2. | Not relevant to the topic |
|  | White RA, Donayre CE, Walot I, Kopchok GE, Wilson EP, Buwalda R, de Virgilio C, Ayres B, Zalewski M, Mehringer CM. Preliminary clinical outcome and imaging criterion for endovascular prosthesis development in high-risk patients who have aortoiliac and traumatic arterial lesions. J Vasc Surg. 1996 Oct;24(4):556-69; discussion 569-71. doi: 10.1016/s0741-5214(96)70071-x. | Inconsistent with study population |
|  | Erdem Y, Haznedaroglu IC, Celik I, Yalcin AU, Yasavul U, Turgan C, Caglar S. Coagulation, fibrinolysis and fibrinolysis inhibitors in haemodialysis patients: contribution of arteriovenous fistula. Nephrol Dial Transplant. 1996 Jul;11(7):1299-305. | Not relevant to the topic |
|  | Prischl FC, Kirchgatterer A, Brandstätter E, Wallner M, Baldinger C, Roithinger FX, Kramar R. Parameters of prognostic relevance to the patency of vascular access in hemodialysis patients. J Am Soc Nephrol. 1995 Dec;6(6):1613-8. doi: 10.1681/ASN.V661613. | Not relevant to the topic |
|  | Grote J, Lufft V, Nikutta P, van der Lieth H, Bahlmann J, Daniel WG. Transesophageal echocardiographic assessment of superior vena cava thrombosis in patients with long-term central venous hemodialysis catheters. Clin Nephrol. 1994 Sep;42(3):183-8. | Not relevant to the topic |
|  | Windus DW. Permanent vascular access: a nephrologist's view. Am J Kidney Dis. 1993 May;21(5):457-71. doi: 10.1016/s0272-6386(12)80391-x. | Not relevant to the topic |
|  | Wong V, Ward R, Taylor J, Selvakumar S, How TV, Bakran A. Factors associated with early failure of arteriovenous fistulae for haemodialysis access. Eur J Vasc Endovasc Surg. 1996 Aug;12(2):207-13. doi: 10.1016/s1078-5884(96)80108-0. | Full text not available |
|  | Wetzig GA, Gough IR, Furnival CM. One hundred cases of arteriovenous fistula for haemodialysis access: the effect of cigarette smoking on patency. Aust N Z J Surg. 1985 Dec;55(6):551-4. doi: 10.1111/j.1445-2197.1985.tb00943.x. | Full text not available |
|  | Gani JS, Fowler PR, Steinberg AW, Wlodarczyk JH, Nanra RS, Hibberd AD. Use of the fistula assessment monitor to detect stenoses in access fistulae. Am J Kidney Dis. 1991 Mar;17(3):303-6. doi: 10.1016/s0272-6386(12)80478-1. | Full text not available |
|  | Thijssen S, Wystrychowski G, Usvyat L, Kotanko P, Levin NW. Determinants of serum albumin concentration analyzed in a large cohort of patients on maintenance hemodialysis. J Ren Nutr. 2007 Jan;17(1):70-4. doi: 10.1053/j.jrn.2006.10.011. | Full text not available |
|  | Strauch BS, O'Connell RS, Geoly KL, Grundlehner M, Yakub YN, Tietjen DP. Forecasting thrombosis of vascular access with Doppler color flow imaging. Am J Kidney Dis. 1992 Jun;19(6):554-7. doi: 10.1016/s0272-6386(12)80834-1. | Full text not available |
|  | De Marchi S, Falleti E, Giacomello R, Stel G, Cecchin E, Sepiacci G, Bortolotti N, Zanello F, Gonano F, Bartoli E. Risk factors for vascular disease and arteriovenous fistula dysfunction in hemodialysis patients. J Am Soc Nephrol. 1996 Aug;7(8):1169-77. doi: 10.1681/ASN.V781169. | Full text not available |
|  | Hirth RA, Turenne MN, Woods JD, Young EW, Port FK, Pauly MV, Held PJ. Predictors of type of vascular access in hemodialysis patients. JAMA. 1996 Oct 23-30;276(16):1303-8. | Not relevant to the topic |
|  | Mickley V, Görich J, Rilinger N, Storck M, Abendroth D. Stenting of central venous stenoses in hemodialysis patients: long-term results. Kidney Int. 1997 Jan;51(1):277-80. doi: 10.1038/ki.1997.33. | Not relevant to the topic |
|  | M A Yerdel, M Kesenci, K M Yazicioglu, Z Döşeyen, A G Türkçapar, E Anadol, Effect of haemodynamic variables on surgically created arteriovenous fistula flow., *Nephrology Dialysis Transplantation*, Volume 12, Issue 8, Aug 1997, Pages 1684–1688, <https://doi.org/10.1093/ndt/12.8.1684> | Not relevant to the topic |
|  | Lin SL, Chen HS, Huang CH, Yen TS. Predicting the outcome of hemodialysis arteriovenous fistulae using duplex ultrasonography. J Formos Med Assoc. 1997 Nov;96(11):864-8. | Not relevant to the topic |
|  | Oakes DD, Sherck JP, Cobb LF. Surgical salvage of failed radiocephalic arteriovenous fistulae: techniques and results in 29 patients. Kidney Int. 1998 Feb;53(2):480-7. doi: 10.1046/j.1523-1755.1998.00769.x. | Not relevant to the topic |
|  | Ifudu O, Mayers JD, Matthew JJ, Fowler A, Friedman EA. Haemodialysis dose is independent of type of surgically-created vascular access. Nephrol Dial Transplant. 1998 Sep;13(9):2311-6. doi: 10.1093/ndt/13.9.2311. | Not relevant to the topic |
|  | Locatelli F, Del Vecchio L, Manzoni C. Morbidity and mortality on maintenance haemodialysis. Nephron. 1998 Dec;80(4):380-400. doi: 10.1159/000045210. | Not relevant to the topic |
|  | Manns BJ, Burgess ED, Parsons HG, Schaefer JP, Hyndman ME, Scott-Douglas NW. Hyperhomocysteinemia, anticardiolipin antibody status, and risk for vascular access thrombosis in hemodialysis patients. Kidney Int. 1999 Jan;55(1):315-20. doi: 10.1046/j.1523-1755.1999.00258.x. | Full text not available |
|  | Woods JD, Port FK, Orzol S, Buoncristiani U, Young E, Wolfe RA, Held PJ. Clinical and biochemical correlates of starting "daily" hemodialysis. Kidney Int. 1999 Jun;55(6):2467-76. doi: 10.1046/j.1523-1755.1999.00493.x. | Full text not available |
|  | Miller PE, Tolwani A, Luscy CP, Deierhoi MH, Bailey R, Redden DT, Allon M. Predictors of adequacy of arteriovenous fistulas in hemodialysis patients. Kidney Int. 1999 Jul;56(1):275-80. doi: 10.1046/j.1523-1755.1999.00515.x. | Full text not available |
|  | Ifudu O, Macey LJ, Homel P, Hyppolite JC, Hong J, Sumrani N, Distant D, Sommer BG, Friedman EA. Determinants of type of initial hemodialysis vascular access. Am J Nephrol. 1997;17(5):425-7. doi: 10.1159/000169135. | Full text not available |
|  | Tonelli M, Muirhead N. Access type as a predictor of dialysis adequacy in chronic hemodialysis patients. ASAIO J. 2000 May-Jun;46(3):279-82. doi: 10.1097/00002480-200005000-00007. | Full text not available |
|  | Allon M, Ornt DB, Schwab SJ, Rasmussen C, Delmez JA, Greene T, Kusek JW, Martin AA, Minda S. Factors associated with the prevalence of arteriovenous fistulas in hemodialysis patients in the HEMO study. Hemodialysis (HEMO) Study Group. Kidney Int. 2000 Nov;58(5):2178-85. doi: 10.1111/j.1523-1755.2000.00391.x. | Full text not available |
|  | Shemesh D, Olsha O, Mabjeesh NJ, Abramowitz HB. Dialysis access induced limb ischemia corrected using quantitative duplex ultrasound. Pediatr Nephrol. 2001 May;16(5):409-11. doi: 10.1007/s004670100585. | Not relevant to the topic |
|  | Dhingra RK, Young EW, Hulbert-Shearon TE, Leavey SF, Port FK. Type of vascular access and mortality in U.S. hemodialysis patients. Kidney Int. 2001 Oct;60(4):1443-51. doi: 10.1046/j.1523-1755.2001.00947.x. | Not relevant to the topic |
|  | Little MA, O'Riordan A, Lucey B, Farrell M, Lee M, Conlon PJ, Walshe JJ. Transplant. 2001 Nov;16(11):2194-200. doi: 10.1093/ndt/16.11.2194. | Not relevant to the topic |
|  | Allon M, Lockhart ME, Lilly RZ, Gallichio MH, Young CJ, Barker J, Deierhoi MH, Robbin ML. Effect of preoperative sonographic mapping on vascular access outcomes in hemodialysis patients. Kidney Int. 2001 Nov;60(5):2013-20. doi: 10.1046/j.1523-1755.2001.00031.x. | Not relevant to the topic |
|  | Turmel-Rodrigues L, Mouton A, Birmelé B, Billaux L, Ammar N, Grézard O, Hauss S, Pengloan J. Salvage of immature forearm fistulas for haemodialysis by interventional radiology. Nephrol Dial Transplant. 2001 Dec;16(12):2365-71. doi: 10.1093/ndt/16.12.2365. | Not relevant to the topic |
|  | He C, Charoenkul V, Kahn T, Langhoff E, Uribarri J, Sedlacek M. Impact of the surgeon on the prevalence of arteriovenous fistulas. ASAIO J. 2002 Jan-Feb;48(1):39-40. doi: 10.1097/00002480-200201000-00009. | Not relevant to the topic |
|  | Pisoni RL, Young EW, Dykstra DM, Greenwood RN, Hecking E, Gillespie B, Wolfe RA, Goodkin DA, Held PJ. Vascular access use in Europe and the United States: results from the DOPPS. Kidney Int. 2002 Jan;61(1):305-16. doi: 10.1046/j.1523-1755.2002.00117.x. | Not relevant to the topic |
|  | Puskar D, Pasini J, Savić I, Bedalov G, Sonicki Z. Survival of primary arteriovenous fistula in 463 patients on chronic hemodialysis. Croat Med J. 2002 Jun;43(3):306-11. | Not relevant to the topic |
|  | Fan SL, Naqvi R, Ahmad R, Thuraisingham R, Raftery MJ, Rudge CJ, Sobeh M, Yaqoob MM. Haemodialysis access: a single centre UK Experience. J Vasc Access. 2002 Jul-Sep;3(3):101-7. doi: 10.1177/112972980200300304. | Not relevant to the topic |
|  | Reddan D, Klassen P, Frankenfield DL, Szczech L, Schwab S, Coladonato J, Rocco M, Lowrie EG, Owen WF Jr; National ESRD CPM Work Group. National profile of practice patterns for hemodialysis vascular access in the United States. J Am Soc Nephrol. 2002 Aug;13(8):2117-24. doi: 10.1097/01.asn.0000022422.79790.a8. | Not relevant to the topic |
|  | Valentine RJ, Bouch CW, Scott DJ, Li S, Jackson MR, Modrall JG, Clagett GP. Do preoperative finger pressures predict early arterial steal in hemodialysis access patients? A prospective analysis. J Vasc Surg. 2002 Aug;36(2):351-6. doi: 10.1067/mva.2002.125848. | Not relevant to the topic |
|  | Lee H, Manns B, Taub K, Ghali WA, Dean S, Johnson D, Donaldson C. Cost analysis of ongoing care of patients with end-stage renal disease: the impact of dialysis modality and dialysis access. Am J Kidney Dis. 2002 Sep;40(3):611-22. doi: 10.1053/ajkd.2002.34924. | Not relevant to the topic |
|  | Chand DH, Poe SA, Strife CF. Venous pressure monitoring does not accurately predict access failure in children. Pediatr Nephrol. 2002 Sep;17(9):765-9. doi: 10.1007/s00467-002-0934-y. | Inconsistent with study population |
|  | Robbin ML, Chamberlain NE, Lockhart ME, Gallichio MH, Young CJ, Deierhoi MH, Allon M. Hemodialysis arteriovenous fistula maturity: US evaluation. Radiology. 2002 Oct;225(1):59-64. doi: 10.1148/radiol.2251011367. | Not relevant to the topic |
|  | Gulati S, Sahu KM, Avula S, Sharma RK, Ayyagiri A, Pandey CM. Role of vascular access as a risk factor for infections in hemodialysis. Ren Fail. 2003 Nov;25(6):967-73. doi: 10.1081/jdi-120026031 | Not relevant to the topic |
|  | Baumann M, Niebel W, Kribben A, Philipp T, Heemann U. Primary failure of arteriovenous fistulae in auto-immune disease. Kidney Blood Press Res. 2003;26(5-6):362-7. doi: 10.1159/000073943. | Not relevant to the topic |
|  | Miller CD, Robbin ML, Allon M. Gender differences in outcomes of arteriovenous fistulas in hemodialysis patients. Kidney Int. 2003 Jan;63(1):346-52. doi: 10.1046/j.1523-1755.2003.00740.x. | Not relevant to the topic |
|  | Punzi M, Ferro F, Petrosino F, Masiello P, Villari V, Sica V, Cavaliere G. Use of an intra-aortic Tesio catheter as vascular access for haemodialysis. Nephrol Dial Transplant. 2003 Apr;18(4):830-2. doi: 10.1093/ndt/gfg025. | Not relevant to the topic |
|  | Lok CE, Oliver MJ. Overcoming barriers to arteriovenous fistula creation and use. Semin Dial. 2003 May-Jun;16(3):189-96. doi: 10.1046/j.1525-139x.2003.16038.x. | Not relevant to the topic |
|  | Obialo CI, Tagoe AT, Martin PC, Asche-Crowe PE. Adequacy and survival of autogenous arteriovenous fistula in African American hemodialysis patients. ASAIO J. 2003 Jul-Aug;49(4):435-9. | Not relevant to the topic |
|  | Shenoy S, Miller A, Petersen F, Kirsch WM, Konkin T, Kim P, Dickson C, Schild AF, Stewart L, Reyes M, Anton L, Woodward RS. A multicenter study of permanent hemodialysis access patency: beneficial effect of clipped vascular anastomotic technique. J Vasc Surg. 2003 Aug;38(2):229-35. doi: 10.1016/s0741-5214(03)00412-9. | Not relevant to the topic |
|  | Doulton T, Sabharwal N, Cairns HS, Schelenz S, Eykyn S, O'Donnell P, Chambers J, Austen C, Goldsmith DJ. Infective endocarditis in dialysis patients: new challenges and old. Kidney Int. 2003 Aug;64(2):720-7. doi: 10.1046/j.1523-1755.2003.00136.x. | Not relevant to the topic |
|  | O'shea SI, Lawson JH, Reddan D, Murphy M, Ortel TL. Hypercoagulable states and antithrombotic strategies in recurrent vascular access site thrombosis. J Vasc Surg. 2003 Sep;38(3):541-8. doi: 10.1016/s0741-5214(03)00321-5. | Not relevant to the topic |
|  | Muyshondt I, Lateur L, Van Roost G, Maes B. Osteolysis induced by AV-fistula in idiopathic carpotarsal osteolysis. Nephrol Dial Transplant. 2003 Oct;18(10):2185-8. doi: 10.1093/ndt/gfg331. | Not relevant to the topic |
|  | Heine GH, Ulrich C, Sester U, Sester M, Köhler H, Girndt M. Transforming growth factor beta1 genotype polymorphisms determine AV fistula patency in hemodialysis patients. Kidney Int. 2003 Sep;64(3):1101-7. doi: 10.1046/j.1523-1755.2003.00176.x. | Not relevant to the topic |
|  | Barama AA. Evaluating the impact of an aggressive strategy to create wrist arterio-venous fistula in patients on hemodialysis. J Vasc Access. 2003 Oct-Dec;4(4):140-5. | Full text not available |
|  | Polkinghorne KR, McDonald SP, Atkins RC, Kerr PG. Epidemiology of vascular access in the Australian hemodialysis population. Kidney Int. 2003 Nov;64(5):1893-902. doi: 10.1046/j.1523-1755.2003.00277.x. | Not relevant to the topic |
|  | Tonelli M, Jhangri GS, Hirsch DJ, Marryatt J, Mossop P, Wile C, Jindal KK. Best threshold for diagnosis of stenosis or thrombosis within six months of access flow measurement in arteriovenous fistulae. J Am Soc Nephrol. 2003 Dec;14(12):3264-9. doi: 10.1097/01.asn.0000099381.98940.2e. | Not relevant to the topic |
|  | Krueger K, Bendel M, Zaehringer M, Reinicke G, Lackner K. Centered endovascular irradiation to prevent postangioplasty restenosis of arteriovenous fistula in hemodialysis patients; Results of a feasibility study. Cardiovasc Radiat Med. 2004 Jan-Mar;5(1):1-8. doi: 10.1016/j.carrad.2004.02.005. | Not relevant to the topic |
|  | Saran R, Pisoni RL, Weitzel WF. Epidemiology of vascular access for hemodialysis and related practice patterns. Contrib Nephrol. 2004;142:14-28. doi: 10.1159/000074876. | Not relevant to the topic |
|  | Roy-Chaudhury P, Kelly BS, Melhem M, Zhang J, Li J, Desai P, Munda R, Heffelfinger SC. Vascular access in hemodialysis: issues, management, and emerging concepts. Cardiol Clin. 2005 Aug;23(3):249-73. doi: 10.1016/j.ccl.2005.04.004. | Not relevant to the topic |
|  | Berardinelli L. Arteriovenous fistulas: different types and surgical techniques. Contrib Nephrol. 2004;142:47-72. doi: 10.1159/000074866. | Not relevant to the topic |
|  | Warnock DG, Tolwani AJ, Gallichio M, Allon M. Vascular grafts for hemodialysis: types, sites and techniques. Contrib Nephrol. 2004;142:73-93. doi: 10.1159/000074867. | Not relevant to the topic |
|  | Newmann JM. The vascular access: a long-term patient's considerations and reflections. Contrib Nephrol. 2004;142:363-75. doi: 10.1159/000074852. | Not relevant to the topic |
|  | Sprouse LR 2nd, Lesar CJ, Meier GH 3rd, Parent FN, Demasi RJ, Gayle RG, Marcinzyck MJ, Glickman MH, Shah RM, McEnroe CS, Fogle MA, Stokes GK, Colonna JO. Percutaneous treatment of symptomatic central venous stenosis [corrected]. J Vasc Surg. 2004 Mar;39(3):578-82. doi: 10.1016/j.jvs.2003.09.034. | Not relevant to the topic |
|  | Vassalotti JA, Falk A, Teodorescu V, Uribarri J. The multidisciplinary approach to hemodialysis vascular access at the Mount Sinai Hospital. Mt Sinai J Med. 2004 Mar;71(2):94-102. | Full text not available |
|  | Polkinghorne KR, McDonald SP, Marshall MR, Atkins RC, Kerr PG. Vascular access practice patterns in the New Zealand hemodialysis population. Am J Kidney Dis. 2004 Apr;43(4):696-704. doi: 10.1053/j.ajkd.2003.11.023. | Not relevant to the topic |
|  | Tessitore N, Bedogna V, Poli A. The role of surveillance in mature arteriovenous fistula management. J Vasc Access. 2004 Apr-Jun;5(2):57-61. doi: 10.1177/112972980400500203. | Not relevant to the topic |
|  | Lorenzo V, Martn M, Rufino M, Hernández D, Torres A, Ayus JC. Predialysis nephrologic care and a functioning arteriovenous fistula at entry are associated with better survival in incident hemodialysis patients: an observational cohort study. Am J Kidney Dis. 2004 Jun;43(6):999-1007. doi: 10.1053/j.ajkd.2004.02.012. | Not relevant to the topic |
|  | Huber TS, Hirneise CM, Lee WA, Flynn TC, Seeger JM. Outcome after autogenous brachial-axillary translocated superficial femoropopliteal vein hemodialysis access. J Vasc Surg. 2004 Aug;40(2):311-8. doi: 10.1016/j.jvs.2004.04.018. | Not relevant to the topic |
|  | Polkinghorne KR, Atkins RC, Kerr PG. Determinants of native arteriovenous fistula blood flow. Nephrology (Carlton). 2004 Aug;9(4):205-11. doi: 10.1111/j.1440-1797.2004.00257.x. | Not relevant to the topic |
|  | Strott KL, Rodgers DJ, Karp SK, Woodruff SD, Wright LD. Increasing the use of arteriovenous fistulas (AVF): a network QI project. Nephrol News Issues. 2004 Aug;18(9):49-53. | Full text not available |
|  | Dember LM, Kaufman JS, Beck GJ, Dixon BS, Gassman JJ, Greene T, Himmelfarb J, Hunsicker LG, Kusek JW, Lawson JH, Middleton JP, Radeva M, Schwab SJ, Whiting JF, Feldman HI; DAC Study Group. Design of the Dialysis Access Consortium (DAC) Clopidogrel Prevention of Early AV Fistula Thrombosis Trial. Clin Trials. 2005;2(5):413-22. doi: 10.1191/1740774505cn118oa. | Not relevant to the topic |
|  | Lomonte C, Petronelli S, Antonelli M, Prudenzano R, Giammaria B, Marchio G, Losurdo N, Basile C. Embolization of haemodialysis arteriovenous fistulas complicated by venous hypertension: a feasibility study. Nephrol Dial Transplant. 2005 Jan;20(1):199-202. doi: 10.1093/ndt/gfh551. | Not relevant to the topic |
|  | Cipleu CD, Cherla GV, Merrill D, Asif A. Can blood flow surveillance and pre-emptive repair of subclinical stenosis prolong the useful life of arteriovenous fistulae? A randomized controlled study. Nephrol Dial Transplant. 2005 Jan;20(1):241-2; author reply 242-3. doi: 10.1093/ndt/gfh600. | Not relevant to the topic |
|  | Yildirim S, Nursal TZ, Yildirim T, Tarim A, Caliskan K. Brachial artery pseudoaneurysm: a rare complication after haemodialysis therapy. Acta Chir Belg. 2005 Apr;105(2):190-3. | Full text not available |
|  | Mallamaci F, Bonanno G, Seminara G, Rapisarda F, Fatuzzo P, Candela V, Scudo P, Spoto B, Testa A, Tripepi G, Tech S, Zoccali C. Hyperhomocysteinemia and arteriovenous fistula thrombosis in hemodialysis patients. Am J Kidney Dis. 2005 Apr;45(4):702-7. doi: 10.1053/j.ajkd.2005.01.004. | Not relevant to the topic |
|  | Lok CE, Oliver MJ, Su J, Bhola C, Hannigan N, Jassal SV. Arteriovenous fistula outcomes in the era of the elderly dialysis population. Kidney Int. 2005 Jun;67(6):2462-9. doi: 10.1111/j.1523-1755.2005.00355.x. | Not relevant to the topic |
|  | Shin SW, Do YS, Choo SW, Lieu WC, Choo IW. Salvage of immature arteriovenous fistulas with percutaneous transluminal angioplasty. Cardiovasc Intervent Radiol. 2005 Jul-Aug;28(4):434-8. doi: 10.1007/s00270-003-0211-x. | Not relevant to the topic |
|  | Elsharawy MA, Moghazy KM. Pre-operative evaluation of hemodialysis access fistula. A multidisciplinary approach. Acta Chir Belg. 2005 Aug;105(4):355-8. doi: 10.1080/00015458.2005.11679735. | Not relevant to the topic |
|  | Zeebregts CJ, Tielliu IF, Hulsebos RG, de Bruin C, Verhoeven EL, Huisman RM, van den Dungen JJ. Determinants of failure of brachiocephalic elbow fistulas for haemodialysis. Eur J Vasc Endovasc Surg. 2005 Aug;30(2):209-14. doi: 10.1016/j.ejvs.2005.04.009. | Not relevant to the topic |
|  | Katzman HE, Glickman MH, Schild AF, Fujitani RM, Lawson JH. Multicenter evaluation of the bovine mesenteric vein bioprostheses for hemodialysis access in patients with an earlier failed prosthetic graft. J Am Coll Surg. 2005 Aug;201(2):223-30. doi: 10.1016/j.jamcollsurg.2005.03.040. | Not relevant to the topic |
|  | Ko SF, Huang CC, Ng SH, Lee TY, Hsieh MJ, Lee FY, Chen MC, Sheen-Chen SM, Lee CH. MDCT angiography for evaluation of the complete vascular tree of hemodialysis fistulas. AJR Am J Roentgenol. 2005 Nov;185(5):1268-74. doi: 10.2214/AJR.04.1553. | Not relevant to the topic |
|  | Toussaint ND, Lau KK, Polkinghorne KR, Kerr PG. Measurement of vascular calcification using CT fistulograms. Nephrol Dial Transplant. 2007 Feb;22(2):484-90. doi: 10.1093/ndt/gfl621. | Not relevant to the topic |

**Table S2. Excluded full text articles (87)**

| No. | Reference | Reason of exclusion |
| --- | --- | --- |
| 1. | Jin XY,Li JS,Wu FU,et al. Construction of risk prediction model for thrombosis in autogenous arteriovenous fistula in maintenance hemodialysis patients [J]. Chinese Journal of Blood Purification, 2024, 23 (03): 209-213.Chinese.doi: 10.3969/j.issn.1671-4091.2024.03.012 | comprehensive predictive models for various AVF complications |
| 2. | Li HB.Analysis of risk factors and construction of a risk prediction model for the complications of autologous arteriovenous fistula in maintenance hemodialysis patients[J].Chinese Journal of Blood Purification,2023,22(11):861-865.Chinese.doi: 10.3969/j.issn.1671-4091.2023.11.014 | comprehensive predictive models for various AVF complications |
| 3. | Heindel P, Dey T, Fitzgibbon JJ, et al. Predicting recurrent interventions after radiocephalic arteriovenous fistula creation with machine learning and the PREDICT-AVF web app. J Vasc Access. 2023;24(23).doi:10.1177/11297298231203356 | Only analyzing influencing factors |
| 4. | Liao XY,Liu HY,Liao ZB. Investigation on related factors of autogenous arteriovenous fistula dysfunction by COX model[J].Pharmaceutical Sciences of China,2021,11(08):145-148.Chinese.DOI：10.3969/j.issn.2095-0616.2021.08.040 | Only analyzing influencing factors |
| 5. | Tang RY. Risk factors of arteriovenous fistula failure in elderly hemodialysis patients : Application of competing risk model[D].Jinan University,2020.  DOI:10.27167/d.cnki.gjinu.2020.001535. | Only analyzing influencing factors |
| 6. | Liang YF,Chen YP,Tu SZ, et al.A nomogram to predict the maturity of arteriovenous fistulas for hemodialysis[J].Chinese Journal of Blood Purification,2019,18(02):110-114.Chinese.  doi: 10.3969/j.issn.1671-4091.2019.02.008 | comprehensive predictive models for various AVF complications |
| 7. | Wei XY,Zhang QF,Wang MR.Construction of an Early Warning Model for Thrombosis Risk of AutologousArteriovenous Fistula in Patients on Long-term Hemodialysis[J].Shanghai Nursing,2023,23(10):29-32.Chinese.  DOI：10.3969/j.issn.1009-8399.2023.10.006 | comprehensive predictive models for various AVF complications |
| 8. | Zheng S,Mei YY,Wang ZH,et al.Application of random forest model and logistic regression model inpredicting arteriovenous fistula dysfunction in maintenance hemodialysispatients[J].China Hospital Statistics,2021,28(06):485-490.Chinese.  DOI:10.3969 /j. issn. 1006-5253. 2021.06. 002 | model comparison study |
| 9. | Zhang BC. Analysis of risk factors for arteriovenous fistula dysfunction in maintenance hemodialysis patients[D].Jilin University. 2018. | Only analyzing influencing factors |
| 10. | Liuqian. Risk factors of loss of arteriovenous fistula in maintenance hemodialysis patients[D]. Qinghai University; 2023. doi:10.27740/d.cnki.gqhdx.2023.000662 | Only analyzing influencing factors |
| 11. | Ma LJ,Zhao SM,Sun F,et al.Value of combined atherogenic index of plasma and non-HDL-cholesterol in predicting vascular access failure in hemodialysis patients[J].Chinese Journal of Blood Purification,2023,22(5):384-388.DOI:10.3969/j.issn.1671-4091.2023.05.015. | Only analyzing influencing factors |
| 12. | Zhang Q. Autogenous Arteriovenous Fistula in Patients Receiving Hemodialysis Pathway Dysfunction of Blood Flow of Risk Assessment and Factor Analysis[D]. Dali University; 2024. doi:10.27811/d.cnki.gdixy.2023.000455 | Only analyzing influencing factors |
| 13. | Zhang F, Yu J, Li G, et al.The risk factors for arteriovenous fistula dysfunction in maintenance hemodialysis patients: A cross-sectional study. Hemodial Int. 2024 Apr;28(2):170-177. doi: 10.1111/hdi.13145. | Only analyzing influencing factors |
| 14. | Wen M, Li Z, Li J, et al.Risk Factors for Primary Arteriovenous Fistula Dysfunction in Hemodialysis Patients: A Retrospective Survival Analysis in Multiple Medical Centers. Blood Purif. 2019;48(3):276-282. doi: 10.1159/000500045. | Only analyzing influencing factors |
| 15. | Zhang F, Li J, Yu J, et al. Risk factors for arteriovenous fistula dysfunction in hemodialysis patients: a retrospective study. Sci Rep. 2023 Dec 3;13(1):21325. doi: 10.1038/s41598-023-48691-4. | Only analyzing influencing factors |
| 16. | Rotmans JI; Dutch Vascular Access Study Group. Arteriovenous Fistula Maturation Failure in a Large Cohort of Hemodialysis Patients in the Netherlands. World J Surg. 2018 Jun;42(6):1895-1903. doi: 10.1007/s00268-017-4382-z. | comprehensive predictive models for various AVF complications |
| 17. | Song L, Quan ZL, Zhao LY,et al. Impact of pulmonary hypertension on arteriovenous fistula failure of hemodialysis patients: A 10 years follow-up cohort study. J Vasc Access. 2023 Mar;24(2):261-270. doi: 10.1177/11297298211027408. | Only analyzing influencing factors |
| 18. | Akin D, Ozmen S, Kaya R. A novel factor for primary arteriovenous fistula failure: hyperinsulinism. Ren Fail. 2016 Sep;38(8):1206-9. doi: 10.1080/0886022X.2016.1209061. | Only analyzing influencing factors |
| 19. | Jankovic A, Damjanovic T, Djuric Z, et al. Calcification in arteriovenous fistula blood vessels may predict arteriovenous fistula failure: a 5-year follow-up study. Int Urol Nephrol. 2017 May;49(5):881-887. doi: 10.1007/s11255-017-1515-0. | Only analyzing influencing factors |
| 20. | HS, Song YR, Kim HJ, et al. Leptin, pre-existing vascular disease, and increased arteriovenous fistula maturation failure in dialysis patients. J Vasc Surg. 2016 Aug;64(2):402-410.e1. doi: 10.1016/j.jvs.2016.03.011. | Only analyzing influencing factors |
| 21. | Rezapour M, Khavanin Zadeh M, et al. Implementation of predictive data mining techniques for identifying risk factors of early AVF failure in hemodialysis patients. Comput Math Methods Med. 2013;2013:830745. doi: 10.1155/2013/830745. | Only analyzing influencing factors |
| 22. | Lamprou A, de Bruin C, van Roon A,et al. Patient-related factors influencing patency of autogenous brachiocephalic haemodialysis fistulas. J Vasc Access. 2017 Mar 6;18(Suppl. 1):104-109. doi: 10.5301/jva.5000675. | Only analyzing influencing factors |
| 23. | Premuzic V, Hudolin T, Pasini J, et al. Hypoproteinemia as a prognostic risk factor for arteriovenous fistula failure. Hemodial Int. 2018 Jan;22(1):37-44. doi: 10.1111/hdi.12538. | Only analyzing influencing factors |
| 24. | Wang T, Cheng Y, He Z,et al. Analysis of high-risk factors for failure of autologous arteriovenous fistula in hemodialysis patients. Zhonghua Wei Zhong Bing Ji Jiu Yi Xue. 2023 Dec;35(12):1281-1285. Chinese. doi: 10.3760/cma.j.cn121430-20230829-00711. | Only analyzing influencing factors |
| 25. | Yang XL,Zhuang F,Xian SL,et al. The influencing factors for early failure of arteriovenous fistula in maintenance hemodialysis patients[J].Chinese Journal of Blood Purification,2023,22(11): 871-875.Chian.doi: 10.3969/j.issn.1671-4091.2023.11.016 | Only analyzing influencing factors |
| 26. | Shi CE. Analysis of influencing factors for arteriovenous fistula dysfunctionin maintenance hemodialysis patients.[D]. Jilin University; 2024. doi:10.27162/d.cnki.gjlin.2023.006477 | Only analyzing influencing factors |
| 27. | Xu Y, Xu Y, Hao DD, et al. Analysis of influencing factors of autogenous arteriovenous fistula dysfunction in maintenance hemodialysis patients[J]. Chinese Journal of Blood Purification, 2020, 19(06): 393-397.Chian.doi: 10.3969/j.issn.1671-4091.2020.06.009 | Only analyzing influencing factors |
| 28. | Wang Tao,CHEN Huan,HE Zhaoxia,et al.The value of C-reactive protein combined with platelet lymphocyte ratio in predicting arteriovenous endovascular fistula loss[J]. Journal of Sichuan North Medical College,2024,39(03):389-392.DOI: 10.3969/j.issn.1005-3697.2024.03.024 | Only analyzing influencing factors |
| 29. | Sun JJ. Risk factors for dysfunction of arteriovenous endovascular fistulae in elderly haemodialysis patients. Chinese Journal of Gerontology. 2022;42(17):4232-4235.Chian.doi: 10. 3969 / j. issn. 1005-9202. 2022. 17. 028 | Only analyzing influencing factors |
| 30. | Xie XT,Liu H,Tu Y,et al. Factors influencing dysfunction of arteriovenous endovascular fistulae in maintenance haemodialysis patients[J].Journal of Nephrology and Dialysis Renal Transplantation,2018,27(05):435-439.Chinese.DOI:1.3969/j.issn.1006-298X.2018.05.007 | Only analyzing influencing factors |
| 31. | Xiong LW, Zhang XG.A study of chronic risk factors for failure of mature autologous arteriovenous endovascular fistulae.Chinese Journal of Gerontology. 2018;17(5):329-334.Chinese.doi:10.3969/j.issn.1671-4091.2018.05.010 | Only analyzing influencing factors |
| 32. | Li L.Logistic regression model based analysis of risk factors affecting arteriovenous endovascular fistula malfunction in elderly maintenance haemodialysis patients[J].Practical Chinese and Western Medicine Clinical,2023,23(05):21-23.Chinese.DOI:10.13638/j.issn.1671-4040.2023.05.006. | Only analyzing influencing factors |
| 33. | Yao Z,Sun LY,Zhao ZJ,et al. Influencing factors, risk prediction and Chinese medicine evidence distribution of autologous arteriovenous endovascular fistulae failure in maintenance haemodialysis patients[J]. Journal of Modern Integrative Chinese and Western Medicine,2022,31(15):2058-2063.Chinese.doi:10.3969/j.issn.1008－ 8849.2022.15.003 | Only analyzing influencing factors |
| 34. | Kong RX, Ge YF, XuXQ, et al. Risk factors of autologous arteriovenous fistula nonfunction in long-term hemodialysis patients. Journal of Clinical Nephrology. 2023;23(11):889-896.Chinese.DOI：10.3969/j.issn.1671-2390.2023.11.002 | Only analyzing influencing factors |
| 35. | Jin X, Fan Y, Li J, et al. Construction of Risk-Prediction Models for Autogenous Arteriovenous Fistula Thrombosis in Patients on Maintenance Hemodialysis. Blood Purif. 2024;53(10):813-823. doi: 10.1159/000540543. | comprehensive predictive models for various AVF complications |
| 36. | Wu LY，Fan PQ，Guo GL， et al. The influencing factors of arteriovenous fistula dysfunction in non-diabetic maintenance hemodialysis patients[J]. Chinese Journal of Blood Purification, 2022, 21(07): 530-535.Chinese.doi: 10.3969/j.issn.1671-4091.2022.07.015 | Only analyzing influencing factors |
| 37. | Engin M, As AK. Triglyceride glucose index as a marker of primary patency rate of arteriovenous fistula in diabetic hemodialysis patients. Eur Rev Med Pharmacol Sci. 2024 May;28(10):3615-3620. doi: 10.26355/eurrev_202405_36298. | Only analyzing influencing factors |
| 38. | Kim MJ, Ko H, Kim SM. Predicting factors for early failure of vascular access in hemodialysis patients. Ann Surg Treat Res. 2024 May;106(5):255-262. doi: 10.4174/astr.2024.106.5.255. | Only analyzing influencing factors |
| 39. | Long J, Chen H, Huang Q, et al. Analysis of risk factors for late arteriovenous fistula failure and patency rates after angioplasty in hemodialysis patients: a retrospective cohort study. Transl Androl Urol. 2024 Feb 29;13(2):209-217. doi: 10.21037/tau-23-431. | Only analyzing influencing factors |
| 40. | Hashmi SA, Hudar SA, Stephen E, et al. Factors Affecting the Early Maturation of Arteriovenous Fistulae Created at a Tertiary Centre in Oman. Sultan Qaboos Univ Med J. 2024 Feb;24(1):37-43. doi: 10.18295/squmj.9.2023.050. | Only analyzing influencing factors |
| 41. | de Winter EP, Wilschut D, Plasmans K, Eefting D, van der Steenhoven T, Putter H, Rotmans J, van der Bogt K. Intraoperative transit time flow measurement predicts maturation of radiocephalic arteriovenous fistulas. J Vasc Surg. 2024 Jul;80(1):232-239. doi: 10.1016/j.jvs.2024.02.028. | Only analyzing influencing factors |
| 42. | Tian Y, Shi K, Zhang Y, et al. Logistic regression analysis of risk factors for hematoma after autologous arteriovenous fistula in hemodialysis patients. Medicine (Baltimore). 2024 Jan 12;103(2):e36890. doi: 10.1097/MD.0000000000036890. | Only analyzing influencing factors |
| 43. | Wang Y, Qin Y, Huang X, Liu W. MSCTA imaging analysis of autologous arteriovenous fistula dysfunction in maintenance hemodialysis patients. Am J Transl Res. 2024 Mar 15;16(3):955-963. doi: 10.62347/YEGN9292. | Only analyzing influencing factors |
| 44. | Zhang YM, Chen W, Wei HL, et al. Analysis of predictive factors of thrombosis in autogenous arteriovenous fistula. J Vasc Access. 2024 Jul;25(4):1134-1139. doi: 10.1177/11297298221151135. | Only analyzing influencing factors |
| 45. | Baek J, Lee H, Yang T, et al. Plasma Interleukin-6 Level Predicts the Risk of Arteriovenous Fistula Dysfunction in Patients Undergoing Maintenance Hemodialysis. J Pers Med. 2023 Jan 12;13(1):151. doi: 10.3390/jpm13010151. | Only analyzing influencing factors |
| 46. | Bodington R, Hazara AM, Lamplugh A, et al. Reassessing the utility of access recirculation and Kt/V for the prediction of arteriovenous fistula failure using online clearance monitoring: the SHUNT STUDY. J Nephrol. 2023 Apr;36(3):677-686. doi: 10.1007/s40620-022-01525-4. | Only analyzing influencing factors |
| 47. | Raksasuk S, Naweera W, Rojwatcharapibarn S, et al. Comparing non-invasive diagnostic methods for arteriovenous fistula stenosis: a prospective study. J Ultrasound. 2023 Sep;26(3):687-693. doi: 10.1007/s40477-022-00731-x. | Only analyzing influencing factors |
| 48. | Kaller R, Arbănași EM, Mureșan AV, Voidăzan S, Arbănași EM, Horváth E, Suciu BA, Hosu I, Halmaciu I, Brinzaniuc K, Russu E. The Predictive Value of Systemic Inflammatory Markers, the Prognostic Nutritional Index, and Measured Vessels' Diameters in Arteriovenous Fistula Maturation Failure. Life (Basel). 2022 Sep 18;12(9):1447. doi: 10.3390/life12091447. | Only analyzing influencing factors |
| 49. | Shindo M, Morino J, Minato S, et al. Risk Factors and Utility of Intraoperative Arteriovenous Fistula Blood Flow Level as a Surrogate Marker of Arteriovenous Fistula Failure in Patients with End-stage Renal Disease. Saudi J Kidney Dis Transpl. 2022 Aug;33(Supplement):S147-S158. doi: 10.4103/1319-2442.384187. | Only analyzing influencing factors |
| 50. | Alturkistani HM, Alsergani AH, Alasqah MI, et al. Predictors of recurrent arteriovenous fistula stenosis in Saudi patients undergoing hemodialysis. Saudi Med J. 2022 Jun;43(6):592-598. doi: 10.15537/smj.2022.43.6.20220192. | Only analyzing influencing factors |
| 51. | Faaborg-Andersen CC, Ramos CR, Minton K, et al. Pre-existing Systolic Dysfunction is the Most Powerful Predictor of Failed Arteriovenous Fistula Maturation. Ann Vasc Surg. 2022 Nov;87:174-180. doi: 10.1016/j.avsg.2022.01.020. | Only analyzing influencing factors |
| 52. | Tsai HC, Ou SM, Wu CC,et al. Pentraxin 3 Predicts Arteriovenous Fistula Functional Patency Loss and Mortality in Chronic Hemodialysis Patients. Am J Nephrol. 2022;53(2-3):148-156. doi: 10.1159/000522049. | Only analyzing influencing factors |
| 53. | Abreu R. New hemodynamic variables as predictors of arteriovenous fistula maturation. Semin Dial. 2022 Jul;35(4):358-362. doi: 10.1111/sdi.13062. | Only analyzing influencing factors |
| 54. | Astor BC, Hirschman K, Kennedy J, et al. Development and validation of a risk score to prioritize patients for evaluation of access stenosis. Semin Dial. 2022 May;35(3):236-244. doi: 10.1111/sdi.13026. | Only analyzing influencing factors |
| 55. | Chen CH, Tao TH, Chou YH, et al. Arteriovenous Fistula Flow Dysfunction Surveillance: Early Detection Using Pulse Radar Sensor and Machine Learning Classification. Biosensors (Basel). 2021 Aug 26;11(9):297. doi: 10.3390/bios11090297. | Only analyzing influencing factors |
| 56. | Chen MC, Weng MJ, Chao LH, et al. Quantitative physical examination indicators to detect patients with stenosis at a high risk of thrombosis at hemodialysis vascular access sites: A retrospective case-control study. J Vasc Access. 2023 Jul;24(4):639-645. doi: 10.1177/11297298211045505. | Only analyzing influencing factors |
| 57. | Bulbul E, Enc N. Factors influencing arteriovenous fistulas: A multicenter study. Ther Apher Dial. 2022 Apr;26(2):441-449. doi: 10.1111/1744-9987.13708. | Only analyzing influencing factors |
| 58. | Chen J, Zhou M, Zeng K, et al. The risk factors of autogenous arteriovenous fistula dysfunction in maintenance hemodialysis patients and the curative effect of personalized nursing. Am J Transl Res. 2021 May 15;13(5):5107-5116. | Only analyzing influencing factors |
| 59. | Venkat Ramanan S, Prabhu RA, Rao IR, Chawla A, et al. Outcomes and predictors of failure of arteriovenous fistulae for hemodialysis. Int Urol Nephrol. 2022 Jan;54(1):185-192. doi: 10.1007/s11255-021-02908-5. | Only analyzing influencing factors |
| 60. | Li Y, Cui W, Wang J, et al. Factors associated with dysfunction of autogenous arteriovenous fistula in patients with maintenance hemodialysis: a retrospective study. Ann Palliat Med. 2021 Apr;10(4):4047-4054. doi: 10.21037/apm-20-2196. | Only analyzing influencing factors |
| 61. | Sun CY, Zhou LF, Song L, et al. How to Reduce the Risk of Arteriovenous Fistula Dysfunction by Observing Prepump Arterial Pressure during Hemodialysis: A Multicenter Retrospective Study. Blood Purif. 2021;50(6):800-807. doi: 10.1159/000512352. | Only analyzing influencing factors |
| 62. | Wärme A, Hadimeri H, Nasic S, et al. The association of erythropoietin-stimulating agents and increased risk for AV-fistula dysfunction in hemodialysis patients. A retrospective analysis. BMC Nephrol. 2021 Jan 18;22(1):30. doi: 10.1186/s12882-020-02209-6. | Only analyzing influencing factors |
| 63. | Ong LM, Paul-Brent PA, Kerr PG, et al. Predictors of Arteriovenous Fistula Failure: A Post Hoc Analysis of the FAVOURED Study. Kidney360. 2020 Sep 14;1(11):1259-1269. doi: 10.34067/KID.0002732020. | Only analyzing influencing factors |
| 64. | Lau B. Selecting important predictors for arteriovenous fistula maturation in older hemodialysis patients by using random survival forests. Semin Dial. 2020 Mar;33(2):148-155. doi: 10.1111/sdi.12866. | Only analyzing influencing factors |
| 65. | He ZJ, Cao JQ, Wu JY, et al. Predictors of primary patency after percutaneous balloon angioplasty for stenosis of Brescia-Cimino hemodialysis arteriovenous fistula. Br J Radiol. 2020 May 1;93(1109):20190505. doi: 10.1259/bjr.20190505. | Only analyzing influencing factors |
| 66. | Du J, Kong X, Liang L,et al. Plasma D-Dimer Level and the Failure of Forearm Autologous Arteriovenous Fistula in Patients With End-Stage Renal Disease. Ther Apher Dial. 2020 Aug;24(4):400-407. doi: 10.1111/1744-9987.13454. | Only analyzing influencing factors |
| 67. | Kumar JS, Sajeev Kumar KS, Arun Thomas ET,et al. Prediction model for successful radiocephalic arteriovenous fistula creation in patients with diabetic nephropathy. Saudi J Kidney Dis Transpl. 2019 Sep-Oct;30(5):1058-1064. doi: 10.4103/1319-2442.270261. | Only analyzing influencing factors |
| 68. | Martinez-Mier G, Camargo-Diaz C, Urbina-Velazquez MA, et al. Predictive Factors for Unsuccessful Use of Arteriovenous Fistula in a Population of End-Stage Renal Disease Patients in Southeastern Mexico. Ann Vasc Surg. 2020 Jan;62:304-309. doi: 10.1016/j.avsg.2019.06.034. | Only analyzing influencing factors |
| 69. | Sankhwar S. The effects of preoperative blood pressure on early failure rate of distal arteriovenous fistulas for hemodialysis access. Hemodial Int. 2019 Jul;23(3):314-318. doi: 10.1111/hdi.12745. | Only analyzing influencing factors |
| 70. | Mok MYM, Chan CT. Risk factors affecting arteriovenous access patency in nocturnal home hemodialysis patients. Clin Nephrol. 2019 May;91(5):284-293. doi: 10.5414/CN109572. | Only analyzing influencing factors |
| 71. | Abreu R, Rioja S, Vallespin J, et al.Predictors of early failure and secondary patency in native arteriovenous fistulas for hemodialysis. Int Angiol. 2018 Aug;37(4):310-314. doi: 10.23736/S0392-9590. | Only analyzing influencing factors |
| 72. | Yap YS, Ting KT, Chi WC, et al. Aortic Arch Calcification as a Predictor of Repeated Arteriovenous Fistula Failure within 1-Year in Hemodialysis Patients. Biomed Res Int. 2017;2017:6728437. doi: 10.1155/2017/6728437. | Only analyzing influencing factors |
| 73. | Jeong HY, Ko EJ, Kim SH, et al.Administration of a High-Dose Erythropoietin-Stimulating Agent in Hemodialysis Patients is Associated with Late Arteriovenous Fistula Failure. Yonsei Med J. 2017 Jul;58(4):793-799. doi: 10.3349/ymj.2017.58.4.793. | Only analyzing influencing factors |
| 74. | Bashar K, Conlon PJ, Kheirelseid EA, et al. Arteriovenous fistula in dialysis patients: Factors implicated in early and late AVF maturation failure. Surgeon. 2016 Oct;14(5):294-300. doi: 10.1016/j.surge.2016.02.001. | Only analyzing influencing factors |
| 75. | Lin CH, Liu YC. Risk Factors for Early Failure of Arteriovenous Vascular Access Among Patients With Type 2 Diabetes Mellitus. Ther Apher Dial. 2016 Apr;20(2):112-7. doi: 10.1111/1744-9987.12383. | Only analyzing influencing factors |
| 76. | Lin CF, Chiou HY, Chang YH, et al. Risk of arteriovenous fistula failure associated with hypnotic use in hemodialysis patients: a nested case-control study. Pharmacoepidemiol Drug Saf. 2016 Aug;25(8):889-97. doi: 10.1002/pds.3963. | Only analyzing influencing factors |
| 77. | Romann A, Beaulieu MC, Rhéaume P, Clement J, Sidhu R, Kiaii M. Risk factors associated with arteriovenous fistula failure after first radiologic intervention. J Vasc Access. 2016 Mar-Apr;17(2):167-74. doi: 10.5301/jva.5000459. | Only analyzing influencing factors |
| 78. | Cheng Q, Zhao YJ. The reasons for the failure of the primary arteriovenous fistula surgery in patients with end-stage renal disease. J Vasc Access. 2015 Nov;16 Suppl 10:S74-7. doi: 10.5301/jva.5000424. Epub 2015 Oct 19. Erratum in: J Vasc Access. 2016 Jan-Feb;17(1):101. doi: 10.5301/JVA.2016.15410. | Only analyzing influencing factors |
| 79. | Yap YS, Chuang HY, Wu CH, et al. Preoperative and Intraoperative Factors for Early Failure of Native Arteriovenous Fistulas. Ther Apher Dial. 2015 Dec;19(6):590-7. doi: 10.1111/1744-9987.12323. | Only analyzing influencing factors |
| 80. | Choi SJ, Yoon HE, Kim YS, et al. Pre-existing Arterial Micro-Calcification Predicts Primary Unassisted Arteriovenous Fistula Failure in Incident Hemodialysis Patients. Semin Dial. 2015 Nov-Dec;28(6):665-9. doi: 10.1111/sdi.12365. | Only analyzing influencing factors |
| 81. | Hadhri S, Rejeb MB, Belarbia A, et al. Hemodialysis duration, human platelet antigen HPA-3 and IgA isotype of anti-β2glycoprotein I antibodies are associated with native arteriovenous fistula failure in Tunisian hemodialysis patients. Thromb Res. 2013 May;131(5):e202-9. doi: 10.1016/j.thromres.2013.03.003. | Only analyzing influencing factors |
| 82. | Gagliardi GM, Mancuso D, Falbo E, et al. Anthropometric parameters of nutritional assessment as predictive factors of arteriovenous fistula malfunction in patients undergoing hemodialysis. J Vasc Access. 2012 Oct-Dec;13(4):475-81. doi: 10.5301/jva.5000096. | Only analyzing influencing factors |
| 83. | Söderberg-Nauclér C, Religa P. A high red blood cell distribution width predicts failure of arteriovenous fistula. PLoS One. 2012;7(5):e36482. doi: 10.1371/journal.pone.0036482. | Only analyzing influencing factors |
| 84. | Bahadi A, Hamzi MA, Farouki MR, Montasser D, Zajjari Y, Arache W, Hassani K, El Amrani M, Alayoud A, Hassani M, Benyahia M, Elallam M, Elkabbaj D, Oualim Z. Predictors of early vascular-access failure in patients on hemodialysis. Saudi J Kidney Dis Transpl. 2012 Jan;23(1):83-7. | Only analyzing influencing factors |
| 85. | Xu YK,Zhang WY,Duan QQ,et al. Study on early failure of autologous arteriovenous fistula due to artery stenosis at anastomosis site[J].Chinese Journal of Blood Purification , 2016, 15(09): 494-497.Chinese.doi: 10.3969/j.issn.1671-4091.2016.09.013 | Only analyzing influencing factors |
| 86. | Doneda M, Poloni S, Bozzetto M, et al. Surgical planning of arteriovenous fistulae in routine clinical practice: A machine learning predictive tool. J Vasc Access. Published online February 10, 2023.doi:10.1177/11297298221147968 | Only analyzing influencing factors |
| 87. | Poushpas S, Normahani P, Kisil I, et al. Tensor decomposition and machine learning for the detection of arteriovenous fistula stenosis: An initial evaluation. Bhattacharjee D, ed. PLOS One. 2023;18(7):e0286952. doi:10.1371/journal.pone.0286952 | Only analyzing influencing factors |

**Table S3 Studies included in synthesis(11)**

| No. | Reference |
| --- | --- |
|  | Weng X Y, Wu H M, Chen Y H, et al. Prognostic factors of late loss of function in arteriovenous fistula based on randomised survival forest. Chinese Journal of Practical Internal Medicine. 2024;44(3):235-242. doi:10.19538/j.nk2024030111 |
|  | Gong C C, Wang T T, Ma Q, et al. Individualised prediction of the risk of dysfunction after arteriovenous endovascular fistula surgery in haemodialysis patients. China Blood Purification. 2024;23(3):214-218. doi：10.3969/j.issn.1671-4091.2024.03.013 |
|  | Wang L, Yang Y, Zhao Q. Retrospective analysis of predictive factors for AVF dysfunction in patients undergoing MHD. Medicine (Baltimore). 2024;103(16):e37737. doi:10.1097/MD.0000000000037737 |
|  | Liang Y, Wang Y Y, Wu S X, et al. Construction and Evaluation of a Predictive Model for Autologous Arteriovenous Endovascular Fistula Failure in Maintenance Haemodialysis Patients. Journal of Nurse Advancement. 2022;37(23):2191-2195. doi:10.16821/j.cnki.hsjx.2022.23.016 |
|  | Che X, Zhang M. Establishment and evaluation of a nomogram prediction model for the failure risk of arteriovenous fistula. Chinese Journal of Vascular Surgery. 2022;7(4):256-260. doi:10.3760/cma.j.cn101411-20220324-00030 |
|  | Peralta R, Garbelli M, Bellocchio F, et al. Development and Validation of a Machine Learning Model Predicting Arteriovenous Fistula Failure in a Large Network of Dialysis Clinics. Int J Environ Res Public Health. 2021;18(23):12355-12367. doi:10.3390/ijerph182312355 |
|  | Wang, G H. Establishment of a prediction model for arteriovenous endovascular fistula loss of function and prognostic analysis of interventional treatment. Master's thesis. People's Liberation Army Navy Medical University of China; 2021. doi:10.26998/d.cnki.gjuyu.2020.000118 |
|  | Wongmahisorn Y. Development and validation of a clinical score to predict 1-year survival of arteriovenous fistula access: a diagnostic study. Ann Surg Treat Res. 2020;98(1):44. doi:10.4174/astr.2020.98.1.44 |
|  | Qian JZ, McAdams-DeMarco MA, Ng D, et al. Validation of a Risk Equation Predicting Hemodialysis Arteriovenous Fistula Primary Failure in Elderly. Am J Nephrol. 2020;51(1):17-23. doi:10.1159/000504466 |
|  | Eslami MH, Zhu CK, Rybin D, et al. Simple Predictive Model of Early Failure among Patients Undergoing First-Time Arteriovenous Fistula Creation. Ann Vasc Surg. 2016;35:46-52. doi:10.1016/j.avsg.2016.01.023 |
|  | Masengu A, Maxwell AP, Hanko JB. Investigating clinical predictors of arteriovenous fistula functional patency in a European cohort. Clin Kidney J. 2016;9(1):142-147. doi:10.1093/ckj/sfv131 |
